# Implementing a climate-resilient and inclusive WASH guidance in Indonesia: A mixed-methods case study

**DOI:** 10.1371/journal.pone.0359266

**Published:** 2026-09-28

**Authors:** Silvia Landa, Fahrunnisa, Sarah Lebu, Novika Noerdiyanti, Musa Manga

**Affiliations:** 1 Department of Environmental Sciences and Engineering, Gillings School of Public Health, University of North Carolina at Chapel Hill, Chapel Hill, North Carolina, United States of America; 2 University of Technology Sumbawa, Sumbawa, Indonesia; 3 The Water Institute at UNC, Department of Environmental Sciences and Engineering, Gillings School of Public Health, University of North Carolina at Chapel Hill, Chapel Hill, North Carolina, United States of America; 4 Yayasan Plan International Indonesia, Jakarta, Indonesia; 5 Department of Construction Economics and Management, College of Engineering, Design, Art and Technology, Makerere University, Kampala, Uganda; National Research and Innovation Agency, INDONESIA

## Abstract

Climate change increasingly disrupts water, sanitation, and hygiene (WASH) services, disproportionately affecting women, children, people with disabilities, and groups with limited adaptive resources. Evidence remains limited on how WASH programs integrate climate resilience, equity, and social inclusion and on the conditions that enable benefits to reach households. Researchers examined how Climate Change Response for Inclusive WASH (CCRIW) guidance was implemented and adapted within routine programming in Indonesia, how implementation outcomes related to post-implementation household access, and which contextual factors enabled or constrained such access. Researchers conducted a mixed-methods study using program data collected between August 2023 and October 2024. Data included key informant interviews (n = 29) and a group discussion (n = 10). Quantitative data included village workshop records (n = 91) and household surveys and observations (n = 1,243) from 12 villages affected by floods, landslides, and drought. Researchers used descriptive statistics and logistic regression and triangulated the findings with deductive qualitative analysis informed by an implementation science framework. Results indicated that CCRIW guidance was perceived as relevant but was adapted during implementation rather than adopted as a complete stand-alone protocol. These adaptations appeared to improve short-term practicality while reducing consistent application, depth of learning, and recall of CCRIW as a distinct approach. Communities primarily adopted lower-cost actions aligned with immediate needs, available resources, and perceived feasibility. Household modifications supported more resilient and inclusive access but could not replace system-level investments under severe hazards. Regression models showed district-level associations for several facility-specific outcomes, limited associations with multiple-hazard exposure, and no consistent associations with reported household characteristics. The findings suggest that CCRIW could connect lived hazard experience with inclusive WASH assessment and local planning but should be embedded within sequenced programming that provides a WASH inclusion foundation and coordinated institutional support to address risks beyond household and village capacity.

## Introduction

Climate change is intensifying pressures on water, sanitation, and hygiene (WASH) services. Extreme weather events, including intense rainfall, flooding, and drought, are becoming more frequent and may occur concurrently within the same geographic areas [1]. These hazards can damage WASH facilities, reduce water availability, degrade water quality, and create adverse consequences for public health [2–5].

Their effects are not evenly distributed, as vulnerable populations often face greater exposure and have fewer resources to respond. Existing inequalities, conflict, and broader development constraints can further intensify climate-related vulnerability, particularly in rural communities whose livelihoods depend heavily on climate-sensitive sectors [1]. The JMP 2025 report highlights that WASH service levels vary by road access, household type, ethnic and indigenous groups, and disability status. For example, the 2022 Papua New Guinea Socio-Demographic and Economic Survey found that households with functional disabilities had 10 percentage points (pp) lower coverage of improved water sources (56%) compared to those without disabilities (66%) and were less likely to have water available on premises [6]. In rural areas, women bear heavier burdens from household water and sanitation tasks, low-income families lack resources to adapt, and people with disabilities face systemic barriers to accessing services. They also face disproportionate impacts due to inadequate water and sanitation, with heightened vulnerability and limited coping capacity, particularly during climate-related hazards such as droughts and flooding [7–9]. Although more than one billion people worldwide live with disabilities and face disproportionate exposure to climate-related risks, the intersection of disability and climate change remains insufficiently addressed in policies, programs, and research. Advancing climate justice therefore requires urgent action to integrate disability inclusion across climate interventions [10], including in the WASH sector.

The experiences of vulnerable groups in WASH contexts are shaped by interdependent dimensions, physical infrastructure, management practices, social capitals, institutional arrangements, and individual capacities, which collectively may influence both hazard exposure and coping potential [2,11–13]. A 14-month study involving 2,353 households and 685 water sources across Bangladesh, Nepal, and Tanzania also demonstrates that physical infrastructure alone does not determine service resilience [14]. Drawing on vulnerability perspective, these dimensions together may influence both exposure and coping potential [15]. For instance, although South Asian women often suffer disproportionately from climate-induced service disruptions, securing land and water rights and inclusive governance forums enables them to exercise significant adaptive agency and drive locally appropriate adaptation measures [16,17]. Similarly, in Micronesia, robust social capital and heightened risk awareness have been shown to strengthen rural communities’ resilience to environmental shocks [18]. These cases demonstrate that, despite systemic disadvantages, marginalized actors can become catalysts for effective adaptation and resilience when critical social and institutional support is in place. Therefore, ensuring the meaningful inclusion of these groups in WASH programs is essential for strengthening overall service resilience.

Although a growing number of WASH programs integrate climate resilience and social inclusion to improve vulnerable groups’ access to and participation in services, the academic literature largely treats these concerns separately [19]. A global review of 110 studies on WASH resilience found no research addressing social inclusion beyond gender [20]. Similarly, a recent meta-review of climate-resilient WASH research identified only one disability-focused study [21]. In the Blue Pacific context, a study synthesized 23 studies on climate–gender partnerships, revealing only two inclusion themes: efforts to enhance diversity within collective action and the incorporation of feminist perspectives in dialogue [22]. Another study mapped 463 intervention studies and showed that 78 percent lacked any Gender Equality and Social Inclusion (GESI) mainstreaming components; of those that did, most targeted women and girls, with few considering other social groups or intersectional dynamics [23]. Although evidence on WASH inequities affecting people with disabilities has grown, a scoping review of 22 studies in low- and middle-income countries found that only two evaluated climate-resilient WASH interventions, neither of which considered disability. Only two studies included evidence on the experiences of people with disabilities [24]. More broadly, although recent studies have begun to examine how climate considerations can be incorporated into existing WASH programming [25,26], two important evidence gaps remain: (1) limited evaluation of interventions that explicitly integrate climate resilience, equity, and social inclusion across WASH services; and (2) limited implementation-focused evidence explaining how implementation guidance is adapted within broader WASH programming and how these adaptations affect outcomes.

Evaluating interventions that integrate resilience and inclusion is necessary to understand how they operate in practice and the conditions under which intended benefits emerge. Such evaluations can reveal how programs are implemented and adapted, how communities experience them, and which contextual enablers and barriers shape household-level outcomes. Without this evidence, practitioners and policymakers have limited guidance on how to sustain WASH access during climate shocks while advancing equity for marginalized groups. Drawing on a case study of an inclusive, climate-resilient WASH intervention in Indonesia, this study addressed three research questions: (1) how was the CCRIW approach implemented and adapted in routine programming? (2) How were implementation outcomes, including relevance, adoption, feasibility, cost, fidelity, and sustainability, associated with post-implementation patterns of household access to climate-resilient and inclusive WASH services? and (3) Which contextual factors enabled or constrained such access at the household level? By integrating implementation evidence with household monitoring data and qualitative findings, this study advances understanding of how inclusive, climate-resilient WASH guidance can be translated from program design into practical action in climate-affected communities.

## Methods

### Study context

The Climate Change Response for Inclusive WASH (CCRIW) guidance was developed jointly by Plan International Indonesia and the Institute for Sustainable Futures at the University of Technology Sydney. It originated from action research conducted in Manggarai District in 2019 and was subsequently refined and expanded by Plan Indonesia [7]. Its conceptual framework examines climate impacts on WASH infrastructure, water resources, ecosystems, livelihoods, access to resources, and psychosocial conditions [12]. Following pilot testing in Manggarai and Sumbawa in 2022, the guidance was revised to include four activities: climate-sensitive community mapping, assessment of climate impacts on WASH accessibility, analysis of gender equality in community-based sanitation, and identification of community resources and future visions [27]. The activities, as detailed in Fig 1, were implemented from July 2023 to December 2024 through Plan Indonesia’s Climate-Resilient and Inclusive WASH (CERIA) project, building on the earlier WASH and Beyond project implemented from 2018 to 2022 focusing on GESI [28].

**Fig 1:**
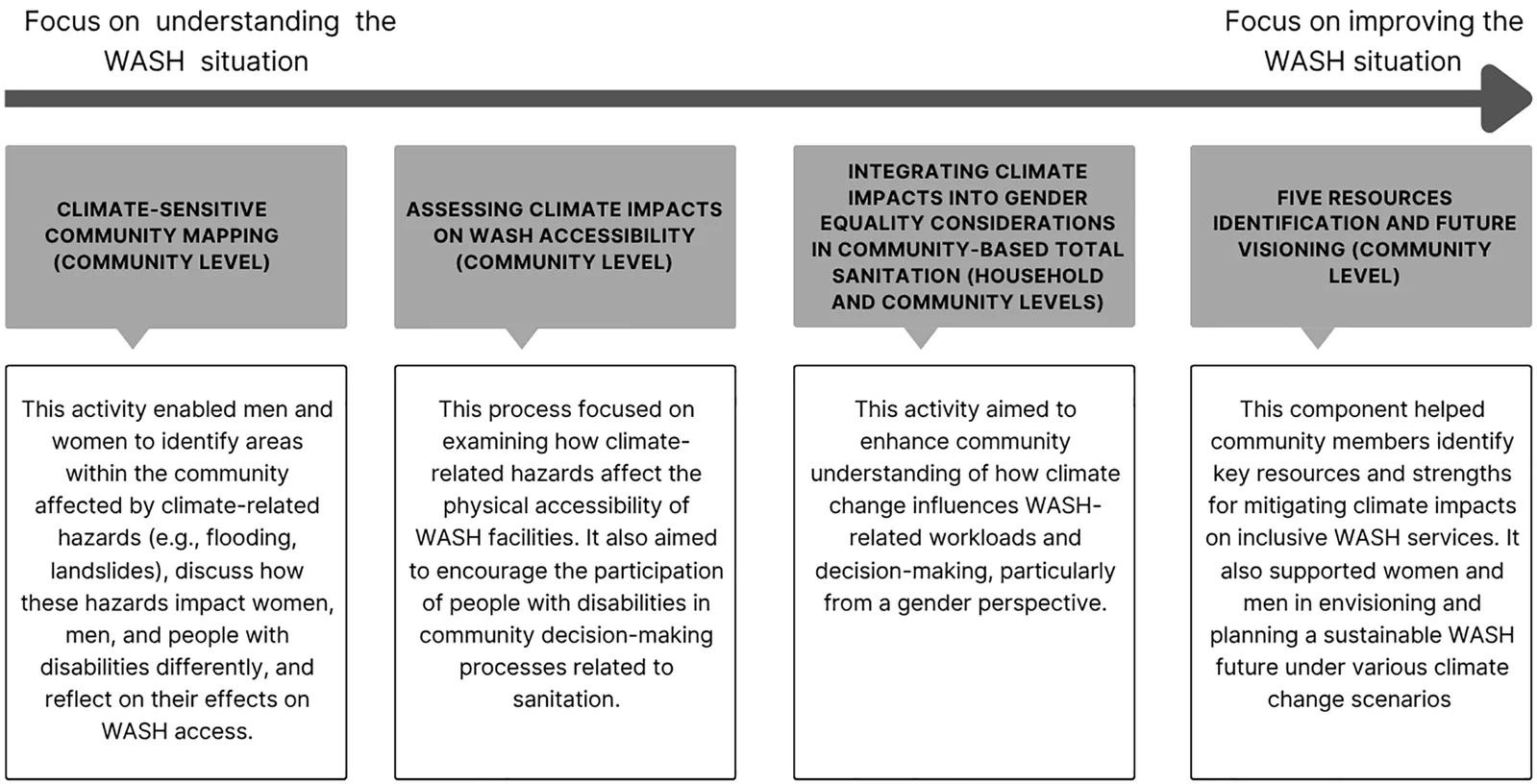
Main activities carried out during CCRIW workshop [29].

After the CCRIW workshop, follow-up activities focused on translating agreed actions into practice through household- and village-level monitoring, complemented by district-level advocacy through Integrated Water Management (IWM) forum. Action plans developed for households, communities, and local governments were advanced through structured facilitation and routine monitoring, with WASH Team supporting communities to pursue locally feasible sanitation improvements using available resources. WASH Team members were trained volunteers operating at the village and sub-district levels, typically supported through village funds or transportation allowances from district health agencies, and sometimes by NGOs such as Plan Indonesia when programs are active in the area.

Plan Indonesia developed and distributed a guidance booklet showcasing inclusive and climate-resilient toilet (upper structures) options to provide practical examples of simple facility modifications and to serve as a reference for implementation in villages, health centers, and schools [30]. To strengthen technical understanding and execution, WASH Team conducted follow-up discussions and provided hands-on support to village governments, while sub-district teams monitored progress at household and village levels through periodic observations and check-ins. Although household-level monitoring was not conducted uniformly throughout implementation, a broader household assessment was carried out at the end of the project to document outcomes. Monitoring results were documented, validated through household and public data, and communicated back to village and district authorities to inform follow-up actions. In parallel, CCRIW action plans were brought to district-level IWM forums to align community priorities with local government planning and to mobilize additional institutional support, including private-sector and corporate social responsibility resources.

### Conceptual framework

Researchers developed a conceptual framework using a simplified program implementation logic model comprising intervention activities, influencing mechanisms or factors, and outcomes, as shown in Fig 2 [31]. The framework was adapted from implementation science approaches used in previous WASH program evaluations in similar contexts [32]. Its initial components were derived from studies examining factors that influence implementation outcomes in community-based inclusive WASH interventions [2,12,13,33]. These components were then refined to reflect the project context, including local environmental conditions, cultural factors, and challenges faced by marginalized groups, including women, youth, and persons with disabilities, in the target areas. The detailed framework, including its components and hypothesized links to project outcomes, is provided in S1 Appendix.

**Fig 2:**
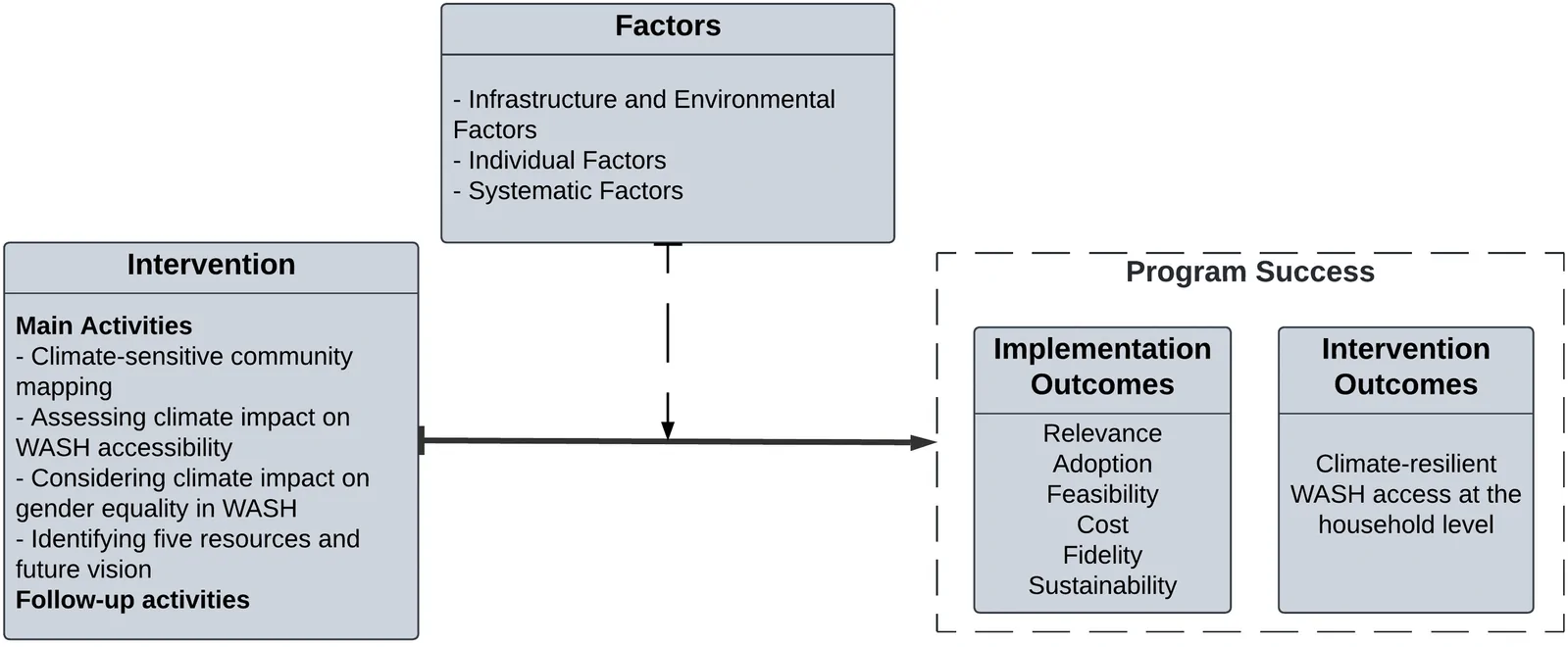
Conceptual framework linking implementation activities, influencing factors, and program outcomes.

Building on the normative definition of climate-resilient WASH services as those that anticipate, recover from, adapt to, or transform in response to climate-related events while maintaining equitable access [34], researchers adopted a narrower operational definition suited to the CCRIW context and household-level monitoring design. Researchers defined climate-resilient access as the ability to access and use WASH facilities during or after climate-related disasters, including floods, landslides, and droughts. This definition captures the dimensions of resilience most directly addressed in the CCRIW guidance and observable in household monitoring data. Researchers defined inclusive access as WASH facilities that are accessible and usable by all people, including people with disabilities, consistent with the CCRIW emphasis on accessibility and usability in service delivery and monitoring [29]

### Study design

Researchers conducted a mixed methods [35] case study. The study combined quantitative project monitoring data with qualitative evidence from key informant interviews and one focus group discussion. Researchers used quantitative data to describe post-implementation patterns of household and facility outcomes and to estimate associations between household characteristics, hazard type, district location, and WASH outcomes. Researchers used qualitative data to interpret implementation processes, contextual enablers and barriers, and mechanisms that could not be captured through monitoring data alone.

#### Quantitative data collection.

This study used secondary quantitative data collected by Plan Indonesia through two existing project monitoring systems. The first dataset comprised implementation data collected during CCRIW workshop activities (S7 Appendix), whereas the second comprised post-implementation household WASH monitoring data (S8 Appendix). The quantitative instruments were developed for project implementation and monitoring rather than specifically for the present analysis.

For the initial implementation analysis, researchers used records generated through workshop activities based on the 2020 CCRIW guidance [29]. The guidance’s research and conceptual foundations are described elsewhere [7,12]. This study focused specifically on Activities 1, 2, and 3 from the guidance, as refined in the 2023 version [27]. The workshop tools and monitoring criteria were adapted for the project context, pretested before implementation, and administered by enumerators trained by the Plan Indonesia monitoring team.

The initial implementation data (S7 Appendix) was collected in August – September 2023 during CCRIW workshop activities in all 91 participating villages, including 32 villages in Sumbawa District and 59 in Manggarai District. Because data collection was integrated into the core CCRIW activities, it covered all project villages rather than a sampled subset. Village-level data were gathered from focus group discussions attended by an average of 26 participants per village. The village, rather than each workshop participant, was treated as the unit of observation in the quantitative village-level dataset.

For household monitoring, researchers used data collected with Plan Indonesia’s structured checklist (S2 Appendix). The project developed the checklist through internal review by WASH, climate, and gender equality, disability, and social inclusion specialists, as well as testing by project field officers. The project monitoring team trained enumerators before administration. Because the checklist was developed for project monitoring, no formal psychometric or measurement-validation study was undertaken. In particular, the reliability of facility classifications and their criterion validity against an external reference standard were not independently assessed.

Household WASH monitoring (S8 Appendix) was conducted between May and June 2024 in 12 villages, with villages purposively selected by Plan Indonesia from each district based on their recent exposure to floods, landslides, or droughts. The selection was intended to represent different hazards and topographic contexts. It was not intended to produce a probability sample representative of all 91 CCRIW villages. Enumerators surveyed 1,291 households. Within each selected village, households were selected through the following procedure: enumerators coordinated with village heads to purposively identify households reflecting relevant variation, including households with members with disabilities and households located in areas exposed to the village’s principal hazards, such as floods, droughts, or landslides. Enumerators assessed household WASH facilities using the structured checklist provided in S2 Appendix. Table 1 summarizes the operational criteria used to construct the climate-resilient and inclusive climate-resilient facilities. They also recorded modifications made by households to improve climate resilience and accessibility.

**Table 1:** Operational criteria for classifying WASH facilities and households.

Classification	Operational criteria
Climate-resilient facility	A completed modification or rehabilitation was documented for the corresponding facility type, and the facility was assessed as structurally sturdy and usable during rain, flooding, and the dry season.
Inclusive and climate-resilient facility	The facility met the climate-resilience criteria, remained accessible during or after rain or flooding, and could be accessed independently by all users, including people with disabilities.
Climate-resilient household	At least one drinking-water, toilet, handwashing, or greywater facility met the climate-resilient facility criteria.
Inclusive and climate-resilient household	At least one of these facilities met both the climate-resilience and accessibility criteria.

Note: Classification did not indicate compliance with engineering standards, technical certification, or continued functionality during repeated climate events. “Inclusive” referred only to physical accessibility and independent use, excluding affordability, safety, privacy, and social acceptability.

Within Plan Indonesia’s programming framework, WASH facilities were classified as drinking water, sanitation, or handwashing facilities [36]. Drinking water facilities included infrastructure used to supply and safely store water for human consumption, such as boreholes, protected wells, piped systems, elevated tanks, cisterns, and sealed containers. Sanitation facilities included blackwater/toilet and greywater systems. Blackwater facilities or toilets comprised infrastructure for containing and managing human excreta, including the superstructure, user interface, and containment system, such as a pit latrine, septic tank, or sewer connection. Greywater facilities included systems for managing wastewater from bathing, laundry, and dishwashing, such as soak pits, lined drains, and pipes that conveyed wastewater away from living areas. Handwashing facilities were defined as designated fixed or mobile facilities with both water and soap available. These included sinks, taps, buckets with taps, tippy taps, and containers fitted with spigots.

#### Qualitative data collection.

Qualitative data were collected through key informant interviews (KIIs) in CCRIW implementation villages in Sumbawa and Manggarai and an online focus group discussion (FGD) with Plan Indonesia CERIA project staff. Between July and October 2024, Fahrunnisa (F), a co-author, conducted the KIIs in person, while Silvia Landa (SAL), the lead author, facilitated the FGD through Zoom. In-person interviews supported rapport and in-depth probing during field visits. The FGD was conducted online because participants were geographically dispersed across villages and districts, reducing travel and scheduling barriers. Written informed consent was obtained before each KII, while FGD participants completed electronic consent forms before participating.

Researchers developed the semi-structured KII and FGD guides based on the initial literature-informed conceptual framework presented in Fig 2 and detailed in S1 Appendix. The framework domains were translated into open-ended questions and follow-up probes addressing implementation experiences, household and community responses to climate hazards, and contextual enablers and barriers. The guides were tailored to household and community participants, village officials, WASH Team members, and project staff. The guides are provided in S3 Appendix. Researchers piloted the qualitative guides with five informants whose characteristics were similar to those of the intended participants and excluded the pilot interviews from the analysis. The pilot assessed question clarity and alignment with the study concepts. Researchers then refined the wording of questions and follow-up probes based on the pilot findings.

The qualitative component used purposive sampling. Plan Indonesia staff identified potential KII participants from relevant stakeholder groups, with selection designed to capture variation in participant roles, gender, disability status, topographic settings, and exposure to climate hazards. The sample size reflected the planned representation of these groups, participant availability, and fieldwork constraints.

The online FGD included 10 members of Plan Indonesia’s CERIA project field team, comprising six men and four women. The study included KIIs with 29 participants: 13 village officials, four WASH Team members, and 12 household or community members. Three participants identified as people with disabilities. Participants were recruited from villages representing highland, lowland, and mixed-topography settings affected by floods, droughts, or landslides. Because sampling was purposive, subgroup sizes were not intended to be equal or statistically representative. Instead, recruitment sought to capture diverse perspectives and reflected local stakeholder composition, availability, and willingness to participate. Participants with disabilities were fewer because they represented a smaller proportion of the population and had more limited availability during data collection. Table 2 presents the characteristics of the 29 KII participants.

**Table 2:** Characteristics of KII Participants, n = 29 participants.

Category	N	%
*Topography of residence*		
Lowland	10	34%
Highland	11	38%
Mixed Lowland and Highland	8	28%
*Most frequently experienced climate hazard*		
Floods	6	21%
Droughts	17	59%
Landslides	6	21%
*Participant role*		
Village Heads/Officials (or staff)	13	45%
WASH Team	4	14%
Households	12	41%
*Gender and disability status*		
Men without disabilities	16	55%
Women without disabilities	10	34%
Men with disabilities	2	7%
Women with disabilities	1	3%
TOTAL	29	

The monitoring instruments, KII, and FGD were designed as program-monitoring and qualitative inquiry tools rather than psychometric scales. Content validity was supported through alignment with the CCRIW conceptual framework and review by authors who are researchers and implementers with expertise in WASH, climate resilient, and disability inclusion. Face validity and comprehensibility were assessed through tools pilot testing informants whose characteristics were similar to those of the intended participants. Based on the pilot interviews, the research team refined the wording of questions and follow-up probes. The complete monitoring protocol for household surveys is provided in S2 Appendix, and the KII and FGD protocols are provided in S3 Appendix.

Community participants were asked about household and community responses to floods, droughts, and landslides, including household decision-making, facility modifications, collective action, and community planning. WASH Team and village or urban village officials were asked about community processes and CCRIW implementation. The FGD and KII sessions were conducted in Indonesian and lasted approximately one hour. With participants’ consent, the sessions were audio-recorded and transcribed in Indonesian for analysis.

#### Analysis method.

**Quantitative analysis.** Of the 1,291 household records, 1,243 contained valid household-composition data and were included in the quantitative analysis. The remaining 48 records were excluded because their household-composition data were missing or invalid. Researchers first calculated descriptive statistics for household characteristics and WASH facility outcomes. Researchers then estimated logistic regression models for two binary household-level outcomes: whether the household had at least one climate-resilient WASH facility and whether it had at least one facility that was both inclusive and climate-resilient. Researchers also estimated separate models for drinking water, toilet, greywater, and handwashing facilities. Following prior WASH research that accounted for household clustering at the village level [37], researchers calculated village-clustered CR2 standard errors. Because the study included only 12 villages, confidence intervals and hypothesis tests used coefficient-specific Satterthwaite-adjusted degrees of freedom, following recommended small-sample procedures for cluster-robust inference [38,39].

The predictors included household size, the presence of children, the presence of household members with disabilities, the proportions of female household members, climate hazard type, and district. Household composition measures were interpreted as household-level characteristics rather than respondent-level demographics. Researchers reported adjusted odds ratios, 95% confidence intervals, and p-values. Because the data were cross-sectional and derived from program monitoring rather than random assignment, the results were interpreted as associations rather than causal effects. All quantitative analyses were conducted in R, as detailed in R code with Markdown in S9 Appendix.

**Qualitative analysis.** Researchers analyzed the KII and FGD data using a primarily deductive thematic approach with inductive refinement using NVivo 15. SAL and F transcribed the recordings and coded the transcripts in Indonesian to preserve their original meaning. SAL developed the initial coding framework with input from the co-authors, drawing on the conceptual framework presented in Fig 2 and relevant WASH resilience research [3,11] to enrich each component’s conceptual depth. SAL and F initially applied the codebook to six interview transcripts. They divided the six transcripts between them and reviewed the resulting coding together. The research team then reviewed the preliminary findings, refined existing codes, and added codes for emergent themes until consensus was reached. SAL and F subsequently coded the full dataset using the finalized codebook, which is provided in S1 Appendix. Coding disagreements were resolved through discussion between SAL and F, with unresolved cases reviewed by other co-authors. Relevant findings and selected quotations were translated into English during manuscript preparation.

Researchers integrated the quantitative and qualitative findings during analysis using the conceptual framework to align themes across both strands. For each theme, researchers compared patterns in the household monitoring data with explanations from the qualitative interviews and discussions. The qualitative findings were used to interpret quantitative results, identify potential mechanisms, explain contextual constraints, and clarify variation across settings.

The research team comprised five individuals with varying degrees of affiliation with the implementing organization. Two team members were affiliated with the implementer, including the lead author (SAL), who previously worked with the organization, and a co-author (NN) who served as the current project manager. This affiliation strengthened contextual understanding but may have influenced interpretation. Three co-authors were unaffiliated with the implementing organization, including one local independent researcher (F) and two university-based experts from outside Indonesia (SL, MM), and they contributed analytical distance. The team managed these risks through role separation where feasible, triangulation across data sources and actor groups, and an audit trail documenting codebook revisions and interpretive decisions.

### Ethics

Ethical approval for KII and FGD data collection and the use of program data for this study was granted by the Ethics Committee of Undana University, Kupang, Indonesia (No. 002218/KEP FKM UNDANA/2024). The Institutional Review Board of the University of North Carolina at Chapel Hill determined that the secondary analysis of all coded program data was exempt from further review (No. 25–3028). Researchers also obtained permission to conduct data collection from the Manggarai and Sumbawa district governments through facilitation by the Plan Indonesia program team.

## Results

### Post-implementation household WASH outcomes

Informants frequently referred to WASH GESI activities or the broader WASH program when discussing CCRIW and often required prompts to provide specific climate-related examples. Many participants also had limited recall of the content covered during CCRIW sessions. Researchers interpreted the observed outcomes as post-implementation patterns and their associated factors within the broader package of interventions implemented at CERIA project sites, including WASH GESI and IWM activities. Among the 1,243 households surveyed across 12 villages, 179 (14.4%) had at least one WASH facility modified to address climate hazards, including rainfall, floods, and droughts. These facilities were classified as climate-resilient within the project context (Table 3). A further 167 (13.4%) of households had any facilities that were both climate-resilient and inclusive, meaning resilient facilities were also accessible to all household members. The difference between the two outcomes was small, ranging from 0.3 to 1.2 pp across facility types. Drinking water and toilet facilities had the highest reported levels of both climate-resilient and inclusive-resilient outcomes, followed by handwashing and greywater facilities.

**Table 3:** Number of climate-resilient and inclusive WASH facilities at household level, n = 1,243.

Type of Facility	Climate-resilient facilities	Inclusive and climate-resilient facilities	Difference between inclusive and climate-resilient vs climate-resilient only
	n (%)	(n,%)	% points
Drinking water	161 (13.0%)	146 (11.7%)	1.2
Sanitation/ wastewater			
- Toilet (black water)	125 (10.1%)	116 (9.3%)	0.7
- Greywater	75 (6.0%)	70 (5.6%)	0.4
Handwashing	107 (8.6%)	103 (8.3%)	0.3
Total Households	179 (14.4%)	167 (13.4%)	1

Across facilities, households most often used low-cost, incremental modifications to keep WASH services usable during shocks and safer for vulnerable users. For drinking water, households primarily expanded storage and, less often, collected rainwater, relied on piped distribution when it functioned, and shifted to wells or boreholes when piped water became turbid or supply failed. For sanitation, households combined hazard protection with accessibility by upgrading or constructing toilets, strengthening toilet superstructures and foundations, adding roofs, and installing handrails or improving access paths. Greywater adaptations focused on drainage channels and simple infiltration wells or holes to reduce pooling, while handwashing practices were maintained under intermittent water availability through stored water (for example, jerry cans) and, in some cases, taps or dedicated handwashing facilities. However, informants also noted threshold effects, whereby severe floods exceeded what incremental upgrades could address and made larger measures, such as embankments or relocation, more relevant but often unaffordable.

Interview evidence suggests that inclusive and climate-resilient features may co-occur because households and village actors pursue “usability under constraints,” meaning practical efforts to keep WASH facilities usable despite climate hazards, limited resources, and accessibility barriers, rather than treating resilience and inclusion as two separate objectives. One respondent captured this overlap across handwashing, toilet design, storage, and disability access.

*“During landslide seasons, we try to locate toilets on higher ground and provide larger water-storage facilities. [...] It is a preparedness measure. During landslides, water becomes discolored and turbid and cannot be used. We therefore prepare larger storage containers. Before a landslide occurs, OPAM (community-based water committee) staff also inspect the pipes. [...] As I mentioned, two years ago the village government distributed taps to every household for handwashing facilities. We use jerry cans specifically for handwashing water and place basins beneath them. We do not waste the used water. We use it to water plants around our houses. [...] Yes. They apply the practices used in my household, such as spreading sand on paths to toilets. They also install supporting handrails near toilets. [...] People with disabilities have responded positively and use these practices. We no longer focus only on households that currently include a person with a disability. We do not know what may happen to us in the future, so we prepare accessible paths to toilets and handwashing facilities now.”* – Household KII [14]

### CCRIW intervention outcomes

#### Relevance.

Participants considered CCRIW relevant to local needs, particularly when it was integrated into the broader WASH program and used to address climate-related hazards. They described changes in community awareness and practices, such as elevating toilets to maintain their usability during floods. Because CCRIW workshop emphasized behavior change for households, households action plans generally prioritized low-cost adaptations over major investments.

*“I am satisfied. The benefit I feel is the change in people’s mindsets concerning the effects of climate change. That is knowledge. The village government and the community have gained that knowledge. We provide information and immediately put it into practice through mapping and other activities. People say, “Oh, so this is how it works.” That is the first effect. Our next task is to keep the knowledge in their minds by supporting it through programs. In an area affected by severe flooding, for example, we consider what type of toilet should be built. We need to elevate it. We ask the community how high the water rises. They show us, and we raise the toilet slightly above that level. That is how it works.”* – Village Official KII [04]

#### Adoption.

None of the villages institutionalized CCRIW as an independent program. The interviews did not reveal one primary explanation for this pattern. Instead, Village Officials generally emphasized practical measures, including modifying facilities, planting trees, and organizing communal work. FGD participants similarly indicated that village leaders primarily engaged with CCRIW by developing action plans and carrying out activities, rather than implementing its structured stages as a separate program.

“*The complete CCRIW method has not been adopted. However, some results from the steps discussed in CCRIW have been adopted and followed up. The full set of activities based on the existing module has not been adopted. Based on our experience in Manggarai, CCRIW activities using the module take a relatively long time. We felt that the approach did not fully suit participants’ backgrounds. If possible, it might be condensed or modified because, in our view, the duration used thus far has been quite long*” – FGD participants [10]

#### Feasibility.

CCRIW implementation at the village and subdistrict levels appeared to strengthen community understanding, reflecting patterns observed in the broader WASH program. Participants indicated that the activities improved awareness of planning for equitable WASH access and of the environmental consequences associated with routine WASH practices. They also reported increased recognition of water conservation measures, such as planting trees and maintaining water sources, as important forms of climate adaptation. Because their accounts frequently combined several program components, these perceived changes could not be attributed to CCRIW alone. Some participants nevertheless emphasized that further training was needed to support a more comprehensive and consistent understanding of effective CCRIW implementation.

*“Yes. Without continued training and specific guidance, the program may gradually fade from community attention. If people regard it as a completed project, implementation may stop. It would be better for the program and support to continue so that communities can sustain what has been learned.”* – WASH Team KII [18]

#### Cost.

Village funding and community contributions through communal work were essential for implementing CCRIW action plans and could support the program’s future adoption. Participants explained that some villages financed sanitation activities through village funds without depending on external assistance from district governments or nongovernmental organizations, whereas urban villages relied more heavily on subdistrict funding. They also noted that voluntary labor and materials supplied by community members helped reduce implementation costs beyond the support provided through formal budgets.

*“We can use simple materials available in the environment. We can make handrails from rope or bamboo along the path. To reduce slipperiness, we can place small stones or gravel on the path. [...] Yes. Handrails were installed, and gravel or small stones were placed on the paths.”* – WASH Team KII [17]

*“The government provides funding for clean-water facilities, while the community contributes labor through mutual cooperation, such as digging wells and pipeline trenches. Construction funding comes from the government, and the labor is a community contribution.”* – WASH Team KII [21]

#### Fidelity.

Some facilitators indicated that implementation generally followed the main CCRIW stages, although some components were not applied consistently. For instance, facilitators did not always use supplementary guidance, including suggested facilitator tips and probing questions for discussions with participants. They also reported that limited time constrained the depth of discussion and opportunities for follow-up. Although the guidance did not prescribe a specific duration, all CCRIW stages were completed within one day. Many informants considered this insufficient because of the complexity of the material and the time needed to develop a thorough understanding. The compressed schedule also limited participants’ ability to learn unfamiliar terminology, suggesting that additional training time could strengthen the quality and consistency of field implementation.

*“We only had one day of training, but the material was supposed to guide us for a whole year. Honestly, one day just wasn’t enough, especially because this was all new to us. Some of the terms were familiar, but many were not. I think it would be much better if the training lasted several days.”* – WASH Team KII [17]

“*There were already quite a lot of activities, and it took a lot of time. In fact, the triggering process was originally planned for two days, but we engaged the community until the afternoon so that it could be completed in a single day*.” – WASH Team KII [21]

#### Sustainability.

Informants indicated that sustaining CCRIW at the village level would require coordinated support across multiple areas, including assistance from district and subdistrict governments, enabling regulations, reliable funding, ongoing training, and systematic monitoring. Participants particularly stressed the need for more frequent monitoring to consolidate progress and maintain program outcomes over time. They also identified stronger regulatory support and more predictable district-level financing as important for embedding CCRIW within regular village programming and ensuring its long-term continuity.

*“We hope the state will remain present and support communities in facing climate-related situations. [...] across all sectors, and that government at every level will support programs that reach the regions […] Without continued training and specific guidance, the program may gradually fade from community attention. If people regard it as a completed project, implementation may stop. It would be better for the program and support to continue so that communities can sustain what has been learned.”*– Village Official KII [26]

### Influencing factors

#### Infrastructure and environmental factors.

**Environmental characteristics**. In the beginning of CCRIW implementation, participants indicated that the most common climate hazards experienced in the study sites over the past 5–10 years were drought (68.1% of 91 villages) and floods or landslides (58.2%), while sea level rise and cyclones or storms were uncommon (<10%), as shown in Fig 3. These hazards were linked to disruptions to WASH services. Nearly all villages (91.2%) reported disruption to access routes to facilities, and reduced comfort and safety of access, such as steep or muddy paths (70.3%) as shown in Fig 4.

**Fig 3:**
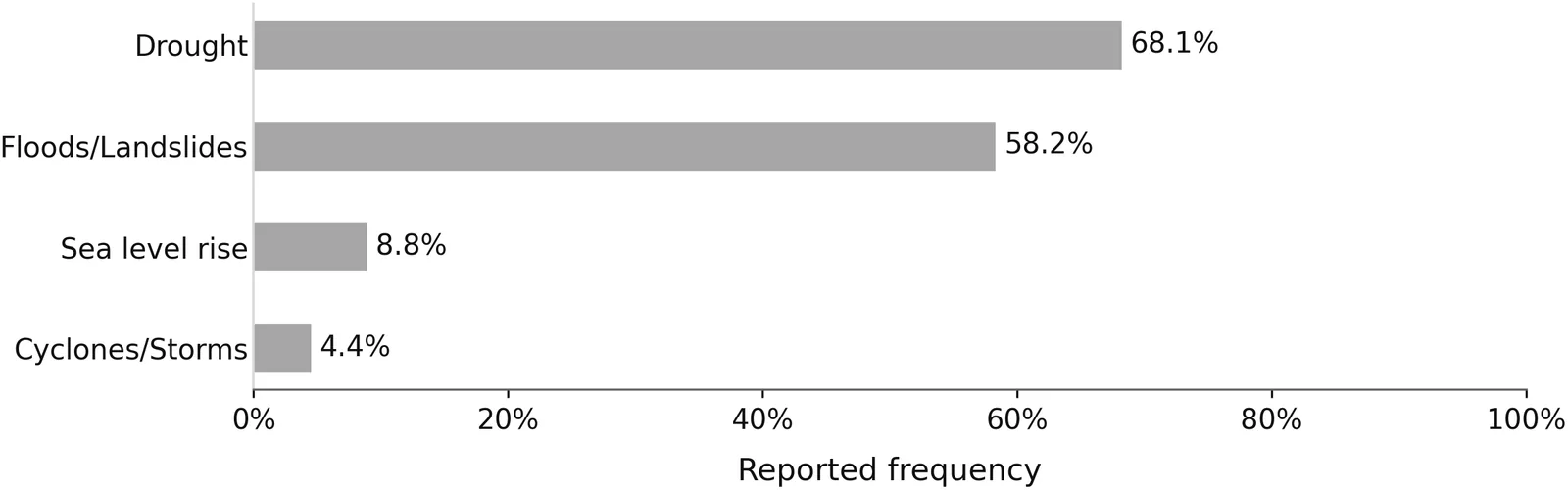
Types of climate-related hazards experienced over the past 5 to 10 years (n = 91 village workshops).

**Fig 4:**
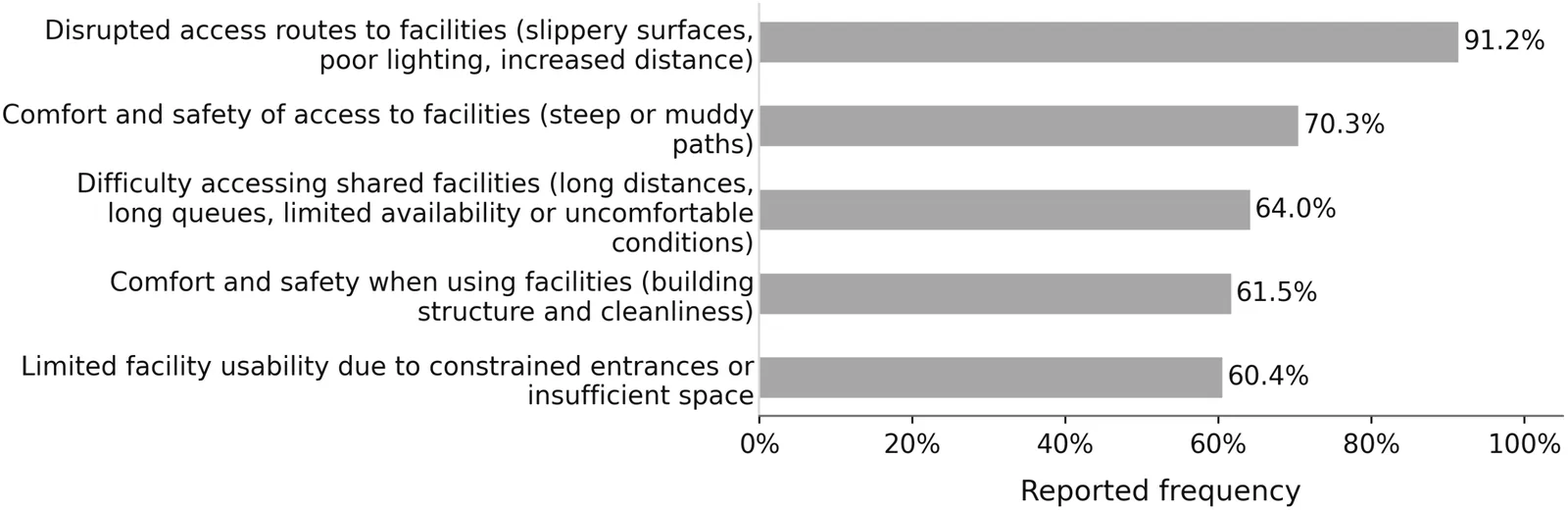
Impacts of climate-related hazards on WASH facility access and use (n = 91 village workshops).

Informants emphasized that these hazards were not purely ‘natural’ events but were amplified by human activity and local geography. In Sumbawa, deforestation linked to cornfield expansion in upstream areas reduced infiltration and accelerated runoff, intensifying floods and landslides and increasing contamination risks and sanitation challenges. In Manggarai, groundwater extraction for commercial uses compounded drought stress, while steep topography created a spatially uneven burden: highland villages faced more acute water shortages, whereas lower-elevation settlements experienced flooding from hillside runoff.

**Infrastructure conditions.** Households relied on simple modifications and pragmatic infrastructure choices to manage climate hazards and maintain accessibility for all household members. Here, “modification” refers to any structural change to WASH facilities intended to reduce hazard exposure (for example: flooding, drought) and or improve inclusive access. Among the 1,243 households included in the quantitative analysis, 264 modification records were documented. After excluding 20 solid-waste-management records, Table 4 summarizes 244 modifications concerning the four WASH facility outcomes. Researchers grouped the standardized modification classifications into the categories presented in Table 4 and used qualitative quotations to illustrate the corresponding practices.

**Table 4:** Facility modifications.

Facility	Modification	Frequency (n, %)	Quotes
Drinking Water	Water distribution through piped system	6 (2.5%)	“*One solution is a managed clean-water system for which users pay monthly fees. Water comes from a source here and is distributed through pipes. PAMSIMAS (community-based water committee) water can be used for purposes other than drinking*.” – Village Officials KII [01]
	Construction/addition of water storage and Rainwater collection	129 (52.9%)	*“In several [local hamlet] areas, some households collect rainwater, although not all households do so”* – WASH Team KII [21]
	Water source protection from flooding	1 (0.4%)	“*Residents in flood-prone areas generally seal drilled wells with concrete so that floodwater does not contaminate drinking and clean water*.” – Household KII [25]
Toilet	Roofing installation/repair	4 (1.6%)	“*If a facility owner has a disability and the toilet is outside, we might recommend adding a roof or temporary shelter during the rainy season, so the facility remains usable*.” – WASH Team KII [23]
	Installation/repair of septic tanks	2 (0.8%)	“*Regarding the septic tank, we considered the proper flow and constructed it permanently with concrete so it would remain strong despite weather changes*.” – Household KII [19]
	Installation or repair of toilet lighting, flooring, and doors	5 (2.0%)	“*For slippery floors, we also suggest replacing them or adding a surface so that they are at least not slippery*.” – WASH Team KII [23]
	Adding water storage in toilet	4 (1.6%)	*“Not only water tanks. In several villages, for example, community members began constructing water-storage basins inside their toilets and bathrooms.”* – FGD participants [10]
	Construction or upgrading of toilets, including permanent superstructures and raised foundations	17 (7.0%)	“*Newly built toilets are elevated because flooding occurs frequently. Households whose bathrooms often flooded renovated them*.” – Village Officials KII [02]
	Adding/repair of access path to toilets and handrails installation inside the toilet	19 (7.8%)	*“They apply the practices used in my household, such as spreading sand on paths to toilets. They also install supporting handrails near toilets. [...] People with disabilities have responded positively and use these practices.”* – Household KII [14]
Greywater	Construction of wastewater channels to drain	18 (7.4%)	“*We constructed a wastewater drainage channel. [...] Water does not stagnate, particularly wastewater from washing dishes or clothes*.” – Household KII [15]
	Construction of simple infiltration well/hole	8 (3.3%)	“*For household wastewater, however, we constructed a soak pit, so the water does not flow outside*.” – Household KII [07]
Handwashing	Construction or modification of handwashing facilities, including taps, water storage, and height adjustments	31 (12.7%)	“*For handwashing, if there is no water coming from the tap, we use stored water in jerry cans*.” – Household KII [14]

Note: Frequencies represent modification records among the 1,243 households included in the quantitative analysis. Percentages were calculated using the 244 modifications concerning drinking water, toilets, greywater, and handwashing as the denominator.

Across facility types, the common strategy was incremental, low-cost adjustments that households could implement quickly, especially when formal services were disrupted. For drinking water, the most common resilience strategy reported was expanding storage and rainwater collection (n = 129, 52.9%), which households described as essential when heavy rainfall damaged pipes and interrupted supply. A smaller share reported reliance on piped distribution (n = 6, 2.5%), often framed as a way to move water from a source through pipes for household use. The study found only one occurrence of protecting water sources from flooding by sealing boreholes with concrete to prevent contamination during flood events (0.4%). These patterns indicate that households perceived piped systems as useful when functional but treated storage and source protection as the more controllable options when shocks disrupted service reliability.

However, when piped water became turbid or pipelines were damaged, households often reverted to wells or boreholes, as their upper structures could be more easily raised or protected from flood waters. However, when hazards reached a certain severity, simpler modifications were often insufficient.

*“That is difficult because floodwater can reach approximately half the height of a house, or about two and a half meters. [...] It may be more realistic to focus on post-flood recovery. If we discuss elevating a bathroom so that water does not enter it, the actual flood level can be as high as two and a half meters and can submerge houses along the riverbank […] A more feasible measure may be constructing a protective embankment, as has been done in another area, to prevent river overflow during the rainy season.”* – Village Official KII [22]

For toilets, households reported a wider range of modifications that combined hazard protection and accessibility. Common changes included constructing or upgrading toilets, including permanent superstructures and raised foundations (n = 17, 7.0%), and improving access paths and installing handrails (n = 19, 7.8%), reflecting the dual need to keep facilities usable during heavy rain and safer for people with disabilities, older adults, and pregnant women. Several households also added water storage inside toilets (n = 4, 1.6%) as part of preparedness for hazard periods. A smaller share reported roofing installation or repair (n = 4, 1.6%) to reduce rain exposure, and installation or repair of septic tanks (n = 2, 0.8%), often emphasizing more durable construction such as permanent concrete to withstand weather changes. Taken together, the toilet results suggest that households prioritized modifications that protect usability during floods and landslides while also addressing mobility and safety constraints for vulnerable users.

During severe floods, for example, relocating toilets or building barriers near rivers were viable options, but these measures required resources beyond what households and communities could afford.

“*Some have been raised. Many residents cannot afford to raise them, so we requested a retaining embankment, although it has not yet been provided. We submitted the request first. We also received district special-allocation funding for 80 toilets across [study village]. The recently constructed toilets are elevated and are unlikely to be inundated by tidal flooding*.” – Household KII [11]

For greywater, households most often reported constructing wastewater channels to drain flow away from the home environment (n = 18, 7.4%) and building simple infiltration wells or holes to prevent wastewater from flowing out and creating unsafe conditions (n = 8, 3.3%). For handwashing, households reported constructing or modifying facilities, including taps, water storage, and occasional height adjustment (n = 31, 12.7%), commonly using stored water in jerry cans when tap water was unavailable.

#### Individual factors.

**Vulnerability and resources**. Participants reported that vulnerable groups, including pregnant women, children, people with disabilities, and older adults, often encountered difficulties accessing WASH facilities during extreme weather. These challenges were especially linked to safety concerns, such as slippery or hard-to-navigate pathways. However, the logistic regression models using CR2 village-clustered standard errors and Satterthwaite-adjusted degrees of freedom showed that none of the measured household characteristics were statistically significantly associated with the resilient or inclusive-resilient WASH outcomes (S4 Appendix). Although the estimated associations with child presence were generally positive, all confidence intervals included the null value. Child presence was not significantly associated with reporting at least one inclusive-resilient WASH facility (OR = 1.615, 95% CI: 0.716, 3.640; p = 0.211), an inclusive-resilient drinking water facility (OR = 1.724, 95% CI: 0.630, 4.717; p = 0.247), a climate-resilient greywater facility (OR = 2.122, 95% CI: 0.662, 6.808; p = 0.162), or an inclusive-resilient greywater facility (OR = 2.119, 95% CI: 0.557, 8.064; p = 0.215). Household size, household disability status, and the proportion of female household members were also not significantly associated with any modeled outcome.

Regarding hazard exposure, the estimated odds ratios for drought were generally greater than 1, but none remained statistically significant after accounting for village-level clustering. Confidence intervals were wide, reflecting the limited number of villages and the small amount of independent village-level information available to estimate hazard and district associations. Multiple-hazard exposure was also not significantly associated with the household-level, drinking water, or toilet outcomes. Although the handwashing and greywater models produced extremely small estimates for multiple-hazard exposure, the coefficient-specific degrees of freedom were approximately 1, indicating potential sparse-data or separation problems. These unstable estimates therefore provided only weak evidence of an association between multiple-hazard exposure and the outcomes.

District was the only predictor with statistically significant and interpretable associations. Compared with households in Manggarai, households in Sumbawa had significantly lower odds of reporting climate-resilient and inclusive-resilient toilet facilities (OR = 0.046, 95% CI: 0.007, 0.304; p = 0.012; and OR = 0.046, 95% CI: 0.007, 0.301; p = 0.012, respectively), handwashing facilities (OR = 0.053, 95% CI: 0.005, 0.565; p = 0.031; and OR = 0.061, 95% CI: 0.005, 0.728; p = 0.039), and greywater facilities (OR = 0.056, 95% CI: 0.005, 0.583; p = 0.032; and OR = 0.066, 95% CI: 0.006, 0.772; p = 0.041). District differences were not statistically significant for the overall household-level or drinking water outcomes. Overall, the models suggest that post-implementation patterns of resilient and inclusive-resilient WASH access were more strongly associated with district location, only weakly associated with multiple-hazard exposure, and not consistently associated with the measured household characteristics.

KII findings indicated that differences in vulnerability and access to resources influenced households’ ability to sustain WASH access. Participants viewed households living in hazard-prone locations, particularly along riverbanks and in coastal areas frequently affected by flooding, as more vulnerable. They also attributed variation in adaptive capacity to differences in knowledge, financial resources, and social support. Awareness of simple measures, such as elevating toilet foundations to reduce flood damage, enabled some households to take protective action. However, limited financial capacity constrained others, particularly those required to purchase water during droughts or pay for sanitation repairs themselves. In one village, households reported spending as much as IDR 250,000, approximately USD 16, per water tank each week, equivalent to around IDR 1 million per month. This expenditure could account for up to half of the average income of a rural Indonesian household.

Participants emphasized that external support, including government assistance, communal labor, and help from family or neighbors, was often essential to cope with these constraints. For example, government-provided clean water was viewed as particularly valuable during droughts, although insufficient in some areas, while community solidarity, such as sharing private wells or jointly purchasing water tanks, was described as critical for households affected by droughts and floods.

The regression findings were consistent with these accounts, as district location was the only statistically significant variable. Compared with households in Manggarai, households in Sumbawa had substantially lower odds of reporting climate-resilient and inclusive-resilient toilet, handwashing, and greywater facilities (S4 Appendix). These differences may reflect variation in hazard severity and the scale of adaptation required. In Sumbawa, 98.5% of monitored households were located in villages affected by floods or landslides, whereas in Manggarai, 65.9% of households were primarily exposed to drought. In Manggarai, households could adopt relatively small and observable measures, such as adding water-storage containers, bathroom reservoirs, and drainage channels. In Sumbawa, however, floods could reach approximately 2.5 meters, making household-level modifications impractical and requiring larger collective infrastructure. The district association may therefore reflect differences in adaptation feasibility, financing, and scale rather than district location alone.

Overall, vulnerability in maintaining WASH access during climate hazards reflected the interaction of individual functional limitations, hazard-prone environments, and unequal access to adaptive resources, with external support often determining whether households could cope when financial capacity was limited.

“*We, as the government, have mainly provided outreach and education to the community. Adopting the approach would require funding [...] but if we are talking about flood-resistant modifications, I do not think we can implement them yet [...] At a minimum, we would need a protective embankment to prevent the river from overflowing during the rainy season*” – Village Official KII [22]

“*Much of [study urban village] lies along a riverbank. Of its 11 community units, seven are located along the river, so they are associated with flood risk […] Some riverbank households have raised their toilets. Others have not because they cannot yet afford it. We continue educating residents about protecting defecation and clean-water facilities. [...] It depends on the weather. In recent years, floodwater usually did not reach residential areas, so the facilities remained safe. When water* *rises beyond its usual level and enters the neighborhood, some toilets are flooded while elevated ones remain unaffected.”.* – Household KII [25]

*“If piped water does not flow, a neighbor has a well where we can collect water. [...] Yes, several nearby neighbors have wells, so it is not far.”* – Household Disability KII [15]

**Participation in activities and decision-making power.** Fig 5 highlights a clear imbalance between workload burden and decision-making power among middle-aged women. Middle-aged women were the group most frequently reported as experiencing increased workload after climate-related hazards, at 64% of 91 villages, compared with 41% for middle-aged men and 20% for young women. However, they were identified as decision-makers in only 32% of villages, while middle-aged men were identified at 78%. This pattern suggests that middle-aged women carried a disproportionate share of disaster-related household and WASH responsibilities, but they did not hold equivalent authority over household decisions during climate-related disruptions. The result points to a gendered division in which women, especially middle-aged women, bore much of the practical burden of coping and recovery, while decision-making power remained concentrated among men.

**Fig 5:**
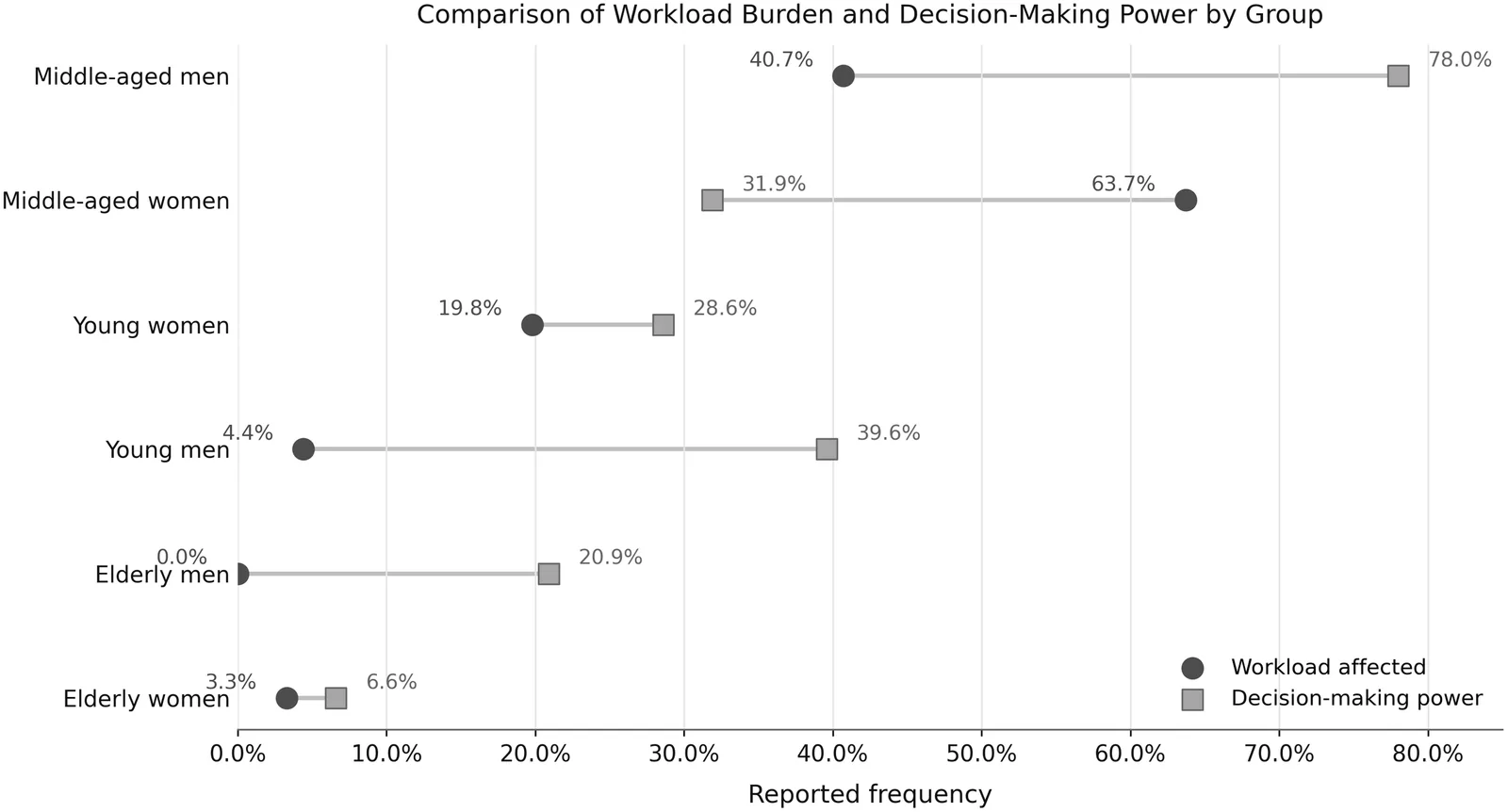
Comparison of workload burden and decision-making power after related hazards (n = 91 village workshops).

Interview findings indicated that village-level participation became more inclusive following CCRIW implementation and subsequent activities, although this change could not be attributed to CCRIW alone. Women, older adults, and people with disabilities were increasingly invited to participate in village programs, although they were generally involved as attendees or regular members rather than as decision-makers. At the household level, women were still seldom recognized as the primary decision-makers, despite carrying greater WASH-related responsibilities during climate-related disasters. Decisions concerning modifications to water and sanitation facilities remained largely controlled by men, suggesting limited change from the initial patterns presented in Fig 5. Some respondents explained that men typically made major decisions but consulted women beforehand. Only a few informants reported more collaborative decision-making, particularly following training that promoted greater equality in household roles.

WASH GESI activities conducted before CCRIW may have contributed to broader awareness of gender equality and inclusive participation in decision-making. However, the interview findings did not provide clear evidence that CCRIW itself changed how disaster-related decisions were made. Women and people with disabilities were increasingly invited to participate, but men continued to hold greater influence over important decisions, particularly within households. Thus, participation expanded in terms of representation, while meaningful equality in decision-making remained limited, especially for responses to climate-related disruptions in WASH services.

*“ “Decision-making is approximately 40 percent women and 60 percent men. […] I am not certain. Male dominance remains relatively strong.”.” –* Household KII [13]

“*Men generally make those decisions because they earn the household income, but they seek women’s opinions. Women also influence household consumption decisions. Physical mutual-cooperation work around the house is generally performed by men. […] In my view, women may bear the greater workload because we must clean the house and think about preparing food for the family, although men perform the heavier physical work*.” *–* Household KII [25]

**Knowledge, experience, and risk perception**. Community understandings of climate adaptation differed from those of facilitator teams and village officials. Knowledge within communities was uneven and generally developed through direct experience and observation of local environmental conditions. Even when residents were unfamiliar with terms such as “climate disaster,” they still adopted practical responses to specific hazards. For instance, households often followed adaptations that had worked for their neighbors, such as elevating toilet foundations to reduce flood exposure during the rainy season. Participants also observed that traditional environmental knowledge, including customary practices and rituals intended to protect natural resources, was becoming less common, particularly in urban areas. Facilitators and village leaders who connected climate change with local development priorities were viewed as more capable of explaining climate hazards and their implications for WASH resilience. Institutional training helped shape the understanding of these actors, whereas changes in community practices appeared to arise more from accumulated experience and repeated engagement in project activities.

Perceptions of climate risk were influenced by the frequency of hazards and by the resources available to respond, including socioeconomic capacity and knowledge of adaptation options. Communities with greater resources and a clearer understanding of practical adaptation measures often felt better prepared, even when disasters occurred frequently. By contrast, communities with fewer resources were more likely to view climate threats with resignation and accept them as part of everyday life. Participants commonly expressed concern about health, food security, and personal safety, particularly among vulnerable groups. Agricultural households were considered especially vulnerable because extreme weather could damage crops, reduce income, and threaten food availability. Under such conditions, immediate concerns about livelihoods and food security often took priority over disruptions to WASH access.

Overall, knowledge, experience, and risk perception interacted to shape how communities responded to climate disasters and their effects on WASH access. Where households understood available resources and feasible adaptation options, they described greater capacity to maintain WASH access during shocks. This pattern suggests that practical knowledge of what can be done, and with which resources, was a key contributor to perceived and enacted resilience.

*“ Until now, we have generally built structures by following customary practice and copying what other people do.”* – Village Officials KII [08]

“*Some residents had already acted on their own before Plan (Plan Indonesia) arrived because they had experienced flooding at several locations. Others made changes after WASH facilitators and the village government provided guidance*.” – Household KII [29]

### Systemic factors

**Governance and community management.** WASH governance involved several actors, including the regional water utility, village WASH teams, community-based water committees, and residents participating through village development planning meetings and communal work. The regional water utility (PDAM) operated through a centralized management structure, but its services were sometimes disrupted during extreme weather, resulting in lower water flow and poorer water quality. In response, some households relied on private wells or stored water as alternative sources.

Community-managed water systems provided residents with more direct involvement in operating and maintaining facilities. However, their effectiveness depended on the community’s capacity to organize management, conduct maintenance, and collect user fees. Responses to disaster-related damage also varied across settings. Informants reported that damaged pipes were generally reported to the management unit responsible, although residents sometimes completed minor repairs themselves or worked collectively to construct and improve village facilities.

These findings indicate that neither centralized PDAM management nor community-based management guarantee resilient and inclusive WASH services. Rather, outcomes depended largely on the quality of management, particularly the regularity of maintenance and the speed of repairs.

*“ No, it has never explained that. When we go to pay the [water utility] bill, we always complain that the water is not flowing.”* – Household KII [17]

*“PAMSIMAS has a management group in [local hamlet], but the group in another hamlet does not function very well”* – Village Official KII [01]

**Institutional support.** Regression analyses showed that post-implementation patterns of resilient and inclusive-resilient WASH access were most strongly associated with district location. Qualitative findings further indicated that institutional factors influenced whether community plans developed through CCRIW workshops could be translated into sustained action. First, financial support allowed village governments and other relevant institutions to carry out measures that households and communities could not afford independently, such as constructing embankments, drilling groundwater wells, and installing pipelines. Village funds also provided an important starting point by supplying initial resources for basic infrastructure and encouraging communities to participate.

Second, district policies and regulatory support helped formally recognize CCRIW and establish opportunities for continued funding. Advocacy related to budgeting and regulation supported the inclusion of CCRIW activities within district-level village financing mechanisms. Cooperation with the Village Community Empowerment Office (PMD) further reinforced this process by promoting sanitation and climate resilience as priorities for the use of village funds.

Third, CCRIW implementation built on the institutional groundwork created by earlier projects. Prior WASH GESI project strengthened attention to gender and social inclusion, which aligned with CCRIW’s focus on inclusive resilience. In parallel, IWM expanded collaboration with district-level institutions and supported partnerships with churches and the private sector, which in turn reinforced village conservation and climate resilience activities. These networks, spanning village government, traditional institutions, and community organizations, supported coordination beyond a single project cycle.

Moreover, monitoring showed whether early gains were maintained, especially for infrastructure maintenance. Although monitoring occurred, it was often irregular and rarely included timely follow-up after CCRIW workshops, which limited opportunities to troubleshoot implementation challenges.

Taken together, CCRIW outcomes depended on a package of enabling conditions: adequate funding, supportive district policies, sustained stakeholder collaboration, and regular monitoring. This also implies that CCRIW’s implementation success could not be separated from the earlier WASH GESI and IWM investments that built capacity and relationships, or from the broader institutional support required to finance and sustain follow-up actions.

*“ CCRIW encouraged activities through the PSDAT (IWM) forum. Many activities were conducted, […] Financial support came from several regional government offices, while seedlings were obtained free of charge from the relevant production and forest-management units. They were planted at several locations. For example, on Environment Day, trees were planted around a small reservoir.”* – FGD participants [10]

## Discussion

This study shows that the CCRIW guidance functioned primarily as an implementation tool embedded within a broader program bundle, rather than as a stand-alone intervention. When asked about CCRIW, informants often referred instead to WASH GESI activities or the broader WASH program. They sometimes needed additional prompts to relate CCRIW to climate-related disasters, and some could not clearly remember what had been discussed during the CCRIW sessions. These patterns suggest that CCRIW knowledge and practices were absorbed into existing routines and messages, rather than forming a distinct program that communities recalled independently. Researchers therefore interpret CCRIW-specific evidence through its identifiable workshop processes and climate-risk and adaptation-planning functions, while interpreting household and institutional patterns primarily within the broader WASH program, including WASH GESI and IWM activities. This matters for interpretation because implementation research distinguishes implementation outcomes from service outcomes and warns that when interventions are not well differentiated in practice, attribution becomes difficult and apparent effects may reflect the broader delivery system rather than the named tool itself [33].

### How was CCRIW implemented and adapted in practice?

CCRIW was implemented in ways that improved short-term practicality but likely reduced the depth of learning, consistency of application, and distinct recall of the approach. The clearest adaptation was the compression of CCRIW stages into a one-day workshop, despite the complexity of the material and participants’ limited familiarity with several key terms. Facilitators also reported that some guidance elements, including special facilitator tips and probing questions, were not applied consistently. From an implementation science perspective, these delivery choices should be interpreted as adaptations with consequences for fidelity and downstream outcomes, rather than as minor procedural deviations. Evidence from school-based WASH programs similarly shows that inadequate training and incomplete delivery of program components can weaken behavioral and health outcomes, even when the intervention itself is relevant [40]. In our case, limited adherence to the intended CCRIW delivery process plausibly contributed to weaker community recall of CCRIW as a distinct intervention. Similar implementation pattern was observed in a process evaluation of Community-Led Total Sanitation in Malawi, which identified variable implementation fidelity, limited household contact, and low recall of hygiene campaigns [41].

At the same time, these adaptations also suggest that CCRIW was used less as a tightly scripted package and more as a practical planning catalyst. Village leaders focused more on practical and visible follow-up actions, including modifying facilities, planting trees, and organizing communal work, than on formally adopting CCRIW as a separate step-by-step process. No village appeared to adopt CCRIW as an independent protocol, but several communities used ideas from the process to guide locally feasible actions. This suggests that CCRIW’s main contribution was not full procedural adoption, but the translation of climate and inclusion concepts into actions that communities could recognize, prioritize, and implement. This interpretation is consistent with evidence from integrated WASH and nutrition interventions in Kenya, where grounding new activities in established community practices improved feasibility and sustained engagement [42].

### How did implementation outcomes relate to household-level climate-resilient and inclusive WASH access?

The implementation outcomes help explain why household-level climate-resilient and inclusive WASH access emerged as a partial but meaningful post-implementation pattern. CCRIW was perceived as relevant because communities were already experiencing droughts, floods, landslides, and disruptions to WASH access. However, limited fidelity and the absence of formal stand-alone adoption meant that household outcomes reflected selective uptake of practical actions rather than full implementation of the CCRIW sequence. In other words, communities did not necessarily adopt CCRIW as a complete protocol, but they did adopt some of the practical ideas that aligned with their immediate needs.

Feasibility and cost strongly shaped which actions households could undertake. Low-cost modifications, such as water storage, raised toilet foundations, drainage improvements, and handrails, were more likely to be adopted because they could be implemented with household resources, village support, or communal labor. By contrast, larger measures such as relocation, embankments, or major infrastructure upgrades remained beyond household capacity. This distinction matters because it shows that household-level inclusive and climate-resilient WASH access was not simply the result of exposure to CCRIW. Rather, it reflected the interaction between CCRIW delivery, feasible household adaptation, local financing, institutional support, and existing community practices. This interpretation aligns with Iyer and Kohlitz’s case studies in Burkina Faso, Bangladesh, and Lao PDR, which show that climate-related sanitation outcomes depend on household resources, livelihoods, social conditions, existing service levels, and program support. They further argue that responsibility for progressive adaptation cannot rest solely with households [25].

Sustainability also depended on conditions outside the household. Continued monitoring, village funding, district-level policy support, and reinforcement through WASH GESI and IWM shaped whether practical actions could be sustained. This finding suggests that CCRIW may contribute to household and village-level adaptation, but only when it is embedded in a wider enabling environment. Without repeated learning cycles, and financing pathways CCRIW risks remain a one-time training activity rather than a sustained implementation process.

### What contextual factors shaped CCRIW outcomes?

Our findings suggested that CCRIW outcomes, interpreted as post-implementation patterns, were shaped by factors at many levels: climate hazards created the main pressure, while institutional support determined whether communities could respond effectively. Initial data showed that drought and floods or landslides were common across sites, and nearly all communities reported negative impacts on WASH facilities, with 91% of villages reporting disrupted access routes and 70.3% reporting reduced comfort and safety of access. Informants also described how land use change and local resource governance amplified risks, including deforestation that intensified runoff and groundwater extraction that compounded drought stress. These results align with evidence that resilience to floods and droughts is shaped not only by infrastructure, but also by environmental setting, management, governance, and institutional support [43].

Individual and social factors shaped uptake and the equity content of implementation. Vulnerable groups experienced heightened access barriers during extreme weather, and households relied on social support networks and government assistance to cope with droughts and flooding. Program participation became more inclusive in invitations to village activities, but decision making on adaptation to climate hazards remained unequal, with men continuing to dominate key household decisions about WASH facility modifications even when women carried greater disaster-related workload. Our case provides an implementation-grounded example in which inclusion can improve attendance but does not necessarily shift decision-making authority. This pattern is consistent with evidence on participation and empowerment gaps in other WASH programs, including Indonesia and Fiji [44]. Related evidence from Bangladesh suggests that empowerment effects may depend on intensive intervention and leadership mainstreaming at the institutional level [45]. With similar leadership- and institution-focused interventions, internal reports from the Plan Indonesia WASH GESI project found increased women’s household-level decision-making authority [46]. However, researchers did not observe comparable evidence specifically for climate-hazard-related decisions in this study, highlighting the need for further research on how and why routine WASH decision-making may differ from hazard-response decision-making.

Infrastructure conditions and household modifications created practical pathways to resilience and inclusion but did not substitute for system-level solutions under severe hazards. Respondents described low-cost modifications such as raising toilets and handwashing facilities, using water storage, and installing assistive features like bamboo handrails that helped people with disabilities and pregnant women navigate slippery paths during heavy rain. However, our results also showed clear limits: when floods were severe, larger measures such as relocation or river embankments were viewed as necessary but often unaffordable for households. This pattern is also supported by the regression results, which suggest that household characteristics mattered less than the district where the household was located. This supports the conclusion that household ingenuity enabled partial resilience, while binding financial and institutional constraints shaped the feasibility and sustainability of CCRIW follow-up actions. This aligns with evidence from the Philippines that autonomous household responses can provide short-term resilience benefits but are unsustainable without institutional coordination [47].

System-level factors most clearly explained why CCRIW was relevant and feasible but not adopted as a stand-alone program. Participants consistently described CCRIW as relevant to local needs and credited it with mindset shifts and practical adaptations, yet formal adoption of CCRIW as an independent process did not occur at village level. Our evidence points to institutional design rather than community rejection: implementation depended on village funds, sub-district funding in urban villages, and community contributions through communal work, and informants emphasized the need for district-level policy and regular monitoring to sustain progress. Moreover, our regression results showed that household characteristics were less strongly associated with resilient and inclusive-resilient WASH access than district location. This is consistent with a conceptual review of community-based adaptation, which argues that projects often fail to sustain outcomes because of multi-level governance gaps and rigid institutional structures rather than lack of community interest, and that effective adaptation requires linking local participation to higher-level policy and finance systems [48].

### Key learning: The role and limits of CCRIW within sequenced WASH programming

The study’s main contribution to knowledge is an empirically informed explanation of how guidance integrating climate resilience and disability-inclusive WASH operates within broader WASH programming. The findings position CCRIW as a complementary planning component rather than a stand-alone intervention. Its most plausible distinctive function was to connect lived hazard experience with inclusive WASH assessment and adaptation planning. Prior WASH GESI programming provided an inclusion foundation, while IWM activities and institutional partners supported coordination, financing, monitoring, and system-level action.

This function builds on the reality that communities often adapt without using “climate change” language. In the study sites, community knowledge was frequently expressed through lived experience and observation rather than technical terminology, and residents described copying practices that seemed to work for neighbors (for example, raising toilet foundations before the rainy season). This fits ethnographic evidence that communities often undertake “everyday adaptation” through experience and social learning without relying on formal climate terminology [49]. It also aligns with evidence on autonomous adaptation, where households adopt risk-reducing measures in response to climate variability and shocks, shaped by local constraints and opportunities rather than formal planning sequences, as documented in rural Ghana [50]. The mechanism indicated by the data is social and experiential learning: people observe what works, test small modifications, and diffuse practices through local networks. This is consistent with evidence on adaptation programming in international development practice [51]. However, as noted earlier, household modifications supported practical gains in resilience and inclusion but did not substitute for system-level solutions under severe hazards. CCRIW may therefore be most useful for helping communities assess, organize, and prioritize locally grounded practices through climate-risk and inclusion perspectives, rather than for introducing adaptation as an entirely new activity.

Household monitoring also indicated that resilience and physical accessibility could co-occur. Across twelve villages, 14.4% of households had climate-resilient WASH facilities and 13.4% had facilities that were both climate-resilient and inclusive, yielding a small gap of about 1 pp. Interview evidence suggests that this co-occurrence reflects households’ and village actors’ practical efforts to keep WASH facilities functional, accessible, and safe under climate-related disruptions and resource limitations, rather than deliberate efforts to achieve resilience and inclusion as two separate objectives. This interpretation aligns with survey patterns showing resilience and inclusion emerging as bundled, low-cost design choices rather than competing priorities. However, this co-occurrence does not imply that all resilience features improve accessibility. Some adaptations enhance resilience but may create new barriers depending on the type of disability. For example, raising toilets can reduce flood damage but may hinder access for wheelchair users. The observed co-occurrence therefore reflects the accumulation of multiple modifications addressing resilience and accessibility in parallel, rather than single features with dual functions. This distinction is important because, although inclusivity is often discussed as supportive of resilience, there is limited empirical evidence on whether inclusive household facility design strengthens resilience to climate hazards, or whether households prioritizing resilience also intentionally consider inclusion and why. These questions remain underexplored and warrant further empirical investigation.

The findings also identify an important capacity boundary, which aligns with resilience-focused WASH research emphasizing that climate resilience depends on institutional support, governance, and wider environmental management that shape disruption during droughts and floods [43], as well as work noting complementarities between everyday adaptation and formal programs [52]. Households and villages could implement lower-cost modifications within their technical and financial capacity, whereas severe or recurrent hazards required infrastructure, financing, and coordination beyond that capacity. CCRIW should therefore be positioned as one component of a sequenced program platform rather than as a stand-alone package. Based on program design and participants’ accounts, researchers interpret prior WASH GESI programming as establishing the inclusion foundation; CCRIW as connecting local hazard experience with inclusive assessment and adaptation planning; and IWM activities and institutional partners as supporting coordination, financing, monitoring, and system-level responses. The study advances implementation knowledge by specifying both the capacity boundary of community-level guidance and the complementary program sequence needed to translate such guidance into action. Integrated guidance can help communities identify and prioritize feasible adaptations, but sustained and equitable WASH resilience requires foundational inclusion programming and institutional support capable of addressing risks beyond household and village capacity.

### Limitations

This study intentionally evaluated CCRIW as it was implemented in routine programming, so the first constraint is limited attribution. Because CCRIW was delivered within a broader platform that included prior WASH GESI programming, IWM, and institutional support, observed changes in household practices, inclusion, or facility access should be interpreted as platform-aligned effects rather than as CCRIW-only causal effects. Second, household monitoring data were collected only after implementation and did not include a pre-intervention baseline from the same households. Researchers therefore describe post-implementation patterns rather than before-after change. Third, the monitoring dataset was collected as part of project monitoring rather than through a statistically designed sampling strategy. As a result, the sample was not designed to ensure representativeness, statistical power, or generalizability beyond the monitored villages. Researchers therefore describe post-implementation patterns that are conditional on the 12 monitored hazard-affected villages, cannot be generalized to all 91 CCRIW villages or lower-risk settings, and may be affected by selection bias. Fourth, the study used descriptive monitoring from purposively selected hazard-affected villages, which may overstate the salience of climate shocks relative to lower-risk settings. Researchers address this by treating estimates as descriptive and triangulating them with qualitative evidence across sites. Finally, researchers relied on program operational definitions of climate-resilient and inclusive-resilient WASH facilities, which were not formally validated; therefore, differences in interpretation across enumerators or households cannot be excluded. The monitoring data may also miss seasonal variability or longer-run performance; researchers addressed this by pairing the monitoring data with informant descriptions of facility access under recent hazard conditions and by interpreting the measures as indicators of practical adaptations rather than full performance tests. Because inclusion was operationalized as physical accessibility and independent usability, the findings do not represent all dimensions of inclusive WASH. These limitations clarify the scope of the claim made in this study.

## Conclusion

Climate hazards disrupted household WASH services and physical accessibility across the study settings. The study’s main contribution is implementation-focused evidence explaining how guidance that integrates climate resilience and disability-inclusive WASH can function as a complementary component of WASH sequenced programming. Such guidance can structure locally grounded planning for actions within household and village capacity, but prior inclusion-focused WASH programming and coordinated institutional, financial, and infrastructure support are needed to address risks that exceed that capacity. CCRIW was relevant to communities experiencing climate-related WASH disruptions, but it was not adopted as a complete stand-alone protocol. Adapted delivery made implementation more practical in the short term, but likely weakened learning depth, consistent use, and community recognition of CCRIW as a distinct approach. Post-implementation household patterns reflected the selective uptake of feasible, lower-cost modifications aligned with immediate needs and available resources. These modifications offered practical pathways toward climate resilience and physical accessibility but could not substitute for infrastructure and institutional responses to severe or recurrent hazards. The findings therefore identify an important capacity boundary: guidance can help households and villages prioritize actions within their financial, technical, and organizational capacities, but it cannot overcome risks that exceed those capacities. Regression findings also highlighted the importance of hazard and district context, although associations varied across outcomes and most measured household characteristics showed limited associations.

Practitioners and policymakers should therefore account for implementation barriers and enablers and position guidance such as CCRIW as a sequenced and reinforced component within an enabling program platform, with sufficient time for facilitator training, community workshops, routine follow-up, and stronger monitoring. Future research should focus on two priorities: first, strengthening sampling strategies and quantitatively assessing the causal pathways through which infrastructure, environmental, individual, and system-level factors shape climate-resilient and inclusive WASH access at the household level; and second, identifying which adaptations to CCRIW delivery improve practicality without undermining fidelity, adoption, and sustainability.

## Supporting information

S1 Appendix Framework and codebook. (DOCX)

S2 Appendix Monitoring protocol. (DOCX)

S3 Appendix KII and FGD protocols. (DOCX)

S4 Appendix Regression results. (DOCX)

S5 Appendix KII and FGD informant characteristics. (XLSX)

S6 Appendix Transcripts. (ZIP)

S7 Appendix CCRIW’s initial implementation data. (XLSX)

S8 Appendix CCRIW’s end of project monitoring data on household WASH access. (XLSX)

S9 Appendix R Markdown Codes. (DOCX)

## References

[1] IPCC. Climate Change 2022: Impacts, Adaptation and Vulnerability Working Group II Contribution to the Sixth Assessment Report of the Intergovernmental Panel on Climate Change. Cambridge, UK and New York, NY, USA: Cambridge University Press. 2022.

[2] Howard G, Calow R, Macdonald A, Bartram J. Climate change and water and sanitation: likely impacts and emerging trends for action. Annu Rev Environ Resour. 2016;41(1):253–76.

[3] Lebu S, Gyimah R, Nandoya E, Brown J, Salzberg A, Manga M. Assessment of sanitation infrastructure resilience to extreme rainfall and flooding: evidence from an informal settlement in Kenya. J Environ Manage. 2024;354:120264. 10.1016/j.jenvman.2024.120264 38354609

[4] Sherpa AM, Koottatep T, Zurbrügg C, Cissé G. Vulnerability and adaptability of sanitation systems to climate change. J Water and Climate Change. 2014;5(4):487–95. doi: 10.2166/wcc.2014.003

[5] Fleming L, Anthonj C, Thakkar MB, Tikoisuva WM, Manga M, Howard G, et al. Urban and rural sanitation in the Solomon Islands: how resilient are these to extreme weather events? Sci Total Environ. 2019;683:331–40. 10.1016/j.scitotenv.2019.05.253 31132712

[6] JMP. Progress on household drinking water, sanitation and hygiene 2000-2024: special focus on inequalities. Geneva: World Health Organization (WHO) and the United Nations Children’s Fund (UNICEF). 2025.

[7] Jeremy Kohlitz JK, Tamara Megaw TM, Anna Gero AG, Silvia Landa SL, Angelo Ximenes AX, Caitlin Leahy CL, et al. Assessing climate impacts on gender and socially inclusive WASH: lessons from a research-practice project. Waterlines. 2022;41(3):1–11. doi: 10.3362/1756-3488.21-00062

[8] Iyer R, Pare Toe L. Impact of climate hazards on rural sanitation and hygiene practices in Burkina Faso. The Sanitation Learning Hub, Institute of Development Studies. 2022.

[9] Dickin S, Segnestam L, Sou Dakouré M. Women’s vulnerability to climate-related risks to household water security in Centre-East, Burkina Faso. Climate and Development. 2020;13(5):443–53. doi: 10.1080/17565529.2020.1790335

[10] Stein PJS, Stein MA, Groce N, Kett M, Akyeampong EK, Alford WP, et al. Advancing disability-inclusive climate research and action, climate justice, and climate-resilient development. The Lancet Planetary Health. 2024;8(4):e242–55. doi: 10.1016/s2542-5196(24)00024-x 38580426

[11] Kohlitz J, Chong J, Willetts J. Rural drinking water safety under climate change: The importance of addressing physical, social, and environmental dimensions. Resources. 2020;9(6):77.

[12] Megaw T, Kohlitz J, Gero A, Chong J. Understanding and responding to climate change impacts in inclusive WASH programs: A conceptual framework. Sydney: ISF-UTS. 2020.

[13] Apatinga GA, Schuster‐Wallace CJ, Dickson‐Anderson SE. A conceptual framework for gender and climate mainstreaming to mitigate water inaccessibility in ruralsub‐SaharanAfrica. WIREs Water. 2022;9(4). doi: 10.1002/wat2.1591

[14] Charles KJ, Howard G, Villalobos Prats E, Gruber J, Alam S, Alamgir ASM. Infrastructure alone cannot ensure resilience to weather events in drinking water supplies. Sci Total Environ. 2022;813:151876. 10.1016/j.scitotenv.2021.151876 34826465

[15] Watts MJ, Bohle HG. The space of vulnerability: the causal structure of hunger and famine. Prog Hum Geogr. 1993;17(1):43–67.

[16] Agarwal B. Gender and forest conservation: The impact of women’s participation in community forest governance. Ecological Economics. 2009;68(11):2785–99. doi: 10.1016/j.ecolecon.2009.04.025

[17] Yadav SS, Lal R. Vulnerability of women to climate change in arid and semi-arid regions: The case of India and South Asia. Journal of Arid Environments. 2018;149:4–17. doi: 10.1016/j.jaridenv.2017.08.001

[18] Hagedoorn LC, Brander LM, van Beukering PJH, Dijkstra HM, Franco C, Hughes L, et al. Community-based adaptation to climate change in small island developing states: an analysis of the role of social capital. Climate and Development. 2019;11(8):723–34. doi: 10.1080/17565529.2018.1562869

[19] Water for Women. Knowledge and Practice Gaps in Climate Resilient Inclusive WASH. Water for Women. 2022.

[20] Tshuma M, Belle JA, Ncube A, Nyam YS, Orimoloye IR. Building resilience to hazards in the water, sanitation, and hygiene (WASH) systems: a global review. Int J Environ Health Res. 2022;34(1):466–78. doi: 10.1080/09603123.2022.2153809 36519285

[21] The University of Leeds, University of Technology Sydney, The University of Bristol, Oxford University. Indicators, measures and methods for monitoring climate resilient WASH – discussion paper. The University of Leeds. 2025. https://eprints.whiterose.ac.uk/id/eprint/225769/1/132_20250422_cr-wash-discussion-paper.pdf

[22] Grant M, Willetts J. Locally led opportunities for water, sanitation and hygiene, climate change and gender equality partnerships in the blue pacific. Water. 2024;16(6):872. doi: 10.3390/w16060872

[23] Macura B, Foggitt E, Liera C, Soto A, Orlando A, Del Duca L, et al. Systematic mapping of gender equality and social inclusion in WASH interventions: knowledge clusters and gaps. BMJ Glob Health. 2023;8(1). 10.1136/bmjgh-2022-010850 PMC9884933 36693669

[24] Wilbur J, Ruuska D, Nawaz S, Natukunda J. Climate risks to water, sanitation and hygiene services and evidence of inclusive and effective interventions in low and middle-income countries: a scoping review. medRxiv. 2024. 10.1371/journal.pgph.0003676 PMC12463209 40997027

[25] Iyer R, Kohlitz J. Climate impacts on rural sanitation: evidence from Burkina Faso, Bangladesh and Lao PDR. Front Water. 2024;6.

[26] Ang R, Cumming-Bruce A, Grant M, Chhaing M, So L, Kim H, et al. Strengthening systems for inclusive climate resilient water, sanitation and hygiene services in Cambodia. H2Open Journal. 2026;9(3):100003. doi: 10.1016/j.htopen.2026.100003

[27] Kohlitz J, Megaw T, Noerdiyanti N, Napope SA, Talan JF, Jatmoko AB. Climate Change Adaptation Strategies for Inclusive WASH: Guidance Note. ISF-UTS. 2023.

[28] Water for Women Fund. Climate-Resilient and Inclusive WASH (CERIA). 2022. https://www.waterforwomenfund.org/en/project/water-for-women---Indonesia.aspx Accessed 2024 June 21.

[29] Kohlitz J, Megaw T, Chong J, Sugi F, Palaipeni P, Emanuel Y. Climate Change Response for Inclusive WASH: A guidance note for Plan International Indonesia. ISF-UTS. 2020.

[30] Plan I. Climate resilient inclusive toilet construction. Plan Indonesia. 2023.

[31] Smith JD, Li DH, Rafferty MR. The implementation research logic model: a method for planning, executing, reporting, and synthesizing implementation projects. Implementation Science. 2020;15(1–2). 10.1186/s13012-020-01041-8 PMC7523057 32988389

[32] Anderson DM, Gupta AK, Birken S, Sakas Z, Freeman MC. Successes, challenges, and support for men versus women implementers in water, sanitation, and hygiene programs: A qualitative study in rural Nepal. Int J Hyg Environ Health. 2021;236:113792. doi: 10.1016/j.ijheh.2021.113792 PMC8325630 34144357

[33] Proctor E, Silmere H, Raghavan R, Hovmand P, Aarons G, Bunger A, et al. Outcomes for implementation research: conceptual distinctions, measurement challenges, and research agenda. Adm Policy Ment Health. 2010;38(2):65–76. doi: 10.1007/s10488-010-0319-7 PMC3068522 20957426

[34] Sanitation and Water for All. Definition of climate-resilient water, sanitation and hygiene services. Sanitation and Water for All. 2025.

[35] Creswell JW, Plano Clark VL. Designing and Conducting Mixed Methods Research. 3rd ed. Los Angeles: SAGE Publications, Inc. 2017.

[36] Plan Indonesia. Training module: Implementation of WASH that is nutrition-sensitive, gender-equal, and inclusive. 2020.

[37] Davies K, Panulo M, MacLeod C, Wilbur J, Morse T, Chidziwisano K, et al. Inclusion of persons living with disabilities in a district-wide sanitation programme: A cross-sectional study in rural Malawi. PLOS Glob Public Health. 2024;4(8):e0003005. doi: 10.1371/journal.pgph.0003005 PMC11361574 39208144

[38] Pustejovsky JE, Tipton E. Small-sample methods for cluster-robust variance estimation and hypothesis testing in fixed effects models. J Business & Economic Statistics. 2017;36(4):672–83. doi: 10.1080/07350015.2016.1247004

[39] Huang FL, Zhang B, Li X. Using robust standard errors for the analysis of binary outcomes with a small number of clusters. J Res Educ Eff. 2023;16(2):213–45.

[40] McMichael C. Water, Sanitation and Hygiene (WASH) in schools in low-income countries: A review of evidence of impact. Int J Environ Res Public Health. 2019;16(3). 10.3390/ijerph16030359 PMC6388361 30696023

[41] Panulo M, Chidziwisano K, MacLeod C, Kapazga T, Dreibelbis R, Beattie TK, et al. Community-Led Total Sanitation implementation in Malawi: Process evaluation of a sanitation and hygiene intervention. Global Public Health. 2026;21(1). doi: 10.1080/17441692.2026.2638018 41764035

[42] Jacob Arriola KR, Ellis A, Webb-Girard A, Ogutu EA, McClintic E, Caruso B, et al. Designing integrated interventions to improve nutrition and WASH behaviors in Kenya. Pilot Feasibility Stud. 2020;6(1). doi: 10.1186/s40814-020-0555-x PMC6998333 32042436

[43] Howard G, Nijhawan A, Flint A, Baidya M, Pregnolato M, Ghimire A, et al. The how tough is WASH framework for assessing the climate resilience of water and sanitation. npj Clean Water. 2021;4(1). doi: 10.1038/s41545-021-00130-5

[44] Mink TL, Salinger AP, Francis N, Batagol B, Burge K, Ilhamsyah N, et al. Who participates in ‘participatory design’ of WASH infrastructure: A mixed-methods process evaluation. PLOS Glob Public Health. 2025;5(6):e0003430. doi: 10.1371/journal.pgph.0003430 PMC12165399 40512713

[45] Lima MH, Salehin M, Chowdhury M d A, Hasan M d H, Hossain MJ, Bala K. From participation to empowerment: the case of women in community-based water management in hydrologically diverse southwest coastal Bangladesh. Environ Dev Sustain. 2025.

[46] Helmi A, Noerdiyanti N, Triwahyudi W. Wash and beyond – transforming lives in eastern Indonesia: final evaluation of Yayasan Plan International Indonesia’s Water for Women Fund project in NTT and NTB. Yayasan Plan International Indonesia and Plan International Australia. 2022.

[47] Maquiling KSM, De La Sala S, Rabé P. Urban resilience in the aftermath of tropical storm Washi in the Philippines: The role of autonomous household responses. Environ Plan B: Urban Analytics and City Sci. 2021;48(5):1025–41. doi: 10.1177/2399808321998693

[48] Dodman D, Mitlin D. Challenges for community-based adaptation: discovering the potential for transformation. J Int Dev. 2013;25(5):640–59.

[49] Castro B, Sen R. Everyday adaptation: Theorizing climate change adaptation in daily life. Global Environ Change. 2022;75:102555. doi: 10.1016/j.gloenvcha.2022.102555

[50] Bawakyillenuo S, Yaro JA, Teye J. Exploring the autonomous adaptation strategies to climate change and climate variability in selected villages in the rural northern savannah zone of Ghana. Local Environment. 2015;21(3):i–ii. doi: 10.1080/13549839.2015.1020020

[51] Ensor J, Harvey B. Social learning and climate change adaptation: Evidence for international development practice. WIREs Climate Change. 2015;6(5):509–22. doi: 10.1002/wcc.348

[52] Lindegaard L, Sen L. Everyday adaptation, interrupted agency and beyond: Examining the interplay between formal and everyday climate change adaptations. Ecosys Soc. 2022;27(4).

