# Supplementary material for: Implementing a climate-resilient and inclusive WASH guidance in Indonesia: A mixed-methods case study
Source: PLoS One. 2026 Sep 28;21(9):e0359266. doi: 10.1371/journal.pone.0359266 (PMC13618932; doi:10.1371/journal.pone.0359266)
Supplement: Supplementary file 1 [file pone.0359266.s001.docx]

## **Key Influencing Factors**

Below are several key frameworks that guided the development of the main components representing factors influencing implementation outcomes.

[Howard et al. (2021)](https://sciwheel.com/work/citation?ids=13542930&pre=&suf=&sa=0&dbf=0)

Six key domains that influence resilience to floods and droughts

- Water Supply and Sanitation Infrastructure: Infrastructure that continues to deliver services effectively even after climate-related events.
- Environmental Setting (Catchment): Availability of catchment protection activities that reduce flood occurrence, increase water infiltration during droughts and prevent landslides.
- Water and Sanitation Management: Adaptive management includes representative WASH structures, sustainable financing, skilled operators, and transparent decision-making, allowing for a quick response to environmental threats.
- Community Governance and Engagement: Communities with established, responsive, and representative civic structures are more resilient, effectively addressing decision-making processes and power dynamics.
- Institutional Support: Support from local or national governments to WASH committees and managers ensures more effective management and operation.

The framework proposed by [Howard et al. (2021)](https://sciwheel.com/work/citation?ids=13542930&pre=&suf=&sa=0&dbf=0) was selected for its specific focus on rural communities and small towns in low- and middle-income countries, making it highly suitable for the study context. Additionally, its robustness is underscored by its development through a comprehensive literature review, expert consultations, and limited field assessments in two countries.

[Apatinga et al. (2022)](https://sciwheel.com/work/citation?ids=16578405&pre=&suf=&sa=0&dbf=0)

The framework by [Apatinga et al. (2022)](https://sciwheel.com/work/citation?ids=16578405&pre=&suf=&sa=0&dbf=0) was chosen because it aims to identify drivers of water (in)accessibility in rural Sub-Saharan Africa (SSA), with a particular emphasis on the intersection between gender and climate change. It employs a rigorous methodology involving a thematic analysis of literature based on a systematic search.

[Megaw et al. (2020)](https://sciwheel.com/work/citation?ids=16547863&pre=&suf=&sa=0&dbf=0)

The framework [Megaw et al. (2020)](https://sciwheel.com/work/citation?ids=16547863&pre=&suf=&sa=0&dbf=0) developed was selected due to its initial development for the Climate Change Resilience in WASH (CCRIW) guidance implemented by Plan Indonesia. It draws upon the foundation of scholarly work on climate change adaptation and resilience, Water, Sanitation, and Hygiene (WASH), and Gender and Social Inclusion (GSI).

*Combining the frameworks for the key influencing factors.*

We adopted three primary components—environmental, systemic, and individual factors—from [Apatinga et al. (2022)](https://sciwheel.com/work/citation?ids=16578405&pre=&suf=&sa=0&dbf=0) to organize the key elements of the framework. Elements from other relevant frameworks were subsequently grouped under these components. To better align with the research objectives, we integrated specific elements from [Apatinga et al. (2022)](https://sciwheel.com/work/citation?ids=16578405&pre=&suf=&sa=0&dbf=0) and Megaw et al. (2020), particularly those addressing gender equity and disability inclusion. However, not all components from [Apatinga et al. (2022)](https://sciwheel.com/work/citation?ids=16578405&pre=&suf=&sa=0&dbf=0) were included, as some were deemed too general for the purposes of this study.

Recurring themes identified during the coding pilot informed the inclusion of environmental, infrastructure, and community management factors from [Howard et al. (2021)](https://sciwheel.com/work/citation?ids=13542930&pre=&suf=&sa=0&dbf=0). The individual-level factors were developed by synthesizing relevant aspects from both [Megaw et al. (2020)](https://sciwheel.com/work/citation?ids=16547863&pre=&suf=&sa=0&dbf=0) and [Apatinga et al. (2022)](https://sciwheel.com/work/citation?ids=16578405&pre=&suf=&sa=0&dbf=0). Finally, the subthemes within the environmental, individual, and systemic categories were shaped by the three core references and refined through inductive analysis of the transcript data.

## **Codebook**

We adopted the implementation outcome categories from [Proctor et al. (2011)](https://sciwheel.com/work/citation?ids=650981&pre=&suf=&sa=0&dbf=0) due to their widespread use in implementation research. However, the outcome category reach was excluded, as it was not relevant to the implementation strategy of the CCRIW program. The definitions of the remaining components—acceptability, adoption, appropriateness, cost, feasibility, fidelity, and sustainability—as well as other themes and sub-themes in the codebook, were refined through initial inductive analysis to ensure their relevance and alignment with the study context.

| Theme/Sub-theme | Definition |
| --- | --- |
| Influencing factors | |
| Infrastructure and Environmental Factors: | |
| Infrastructure Conditions | Types and modifications of WASH infrastructure that are resilient to climate-related disasters and accessible to all household members—including persons with disabilities, the elderly, and pregnant women—are influenced by the design and maintenance of these facilities. |
| Environmental Characteristics | Features such as frequent climate hazards, topography, and land use patterns that shape disaster risk exposure in communities. |
| Systematic factors: | |
| Governance and Community Management | Community-level systems for planning, resource allocation, coordination, and decision-making that are essential to ensuring inclusive and sustainable WASH access, as well as preparedness and response to climate-related disasters. |
| Institutional support | Regulatory and financial support from local government, multisectoral collaboration, and oversight mechanisms that affect local-level implementation and service delivery. |
| Individual Factors: | |
| Vulnerability and Resources | Vulnerability: The degree to which individuals are unable to cope with, respond to, and recover from the adverse impacts of climate-related disasters. This may be shaped by both individual characteristics and environmental factors, including personal attributes such as age, gender, and disability status.  Resources: Assets, capabilities, or strengths individuals can draw upon to reduce their vulnerability. |
| Participation in Activities and Decision-Making Power | The extent to which individuals actively engage in climate-resilient WASH activities and are equitably involved in decision-making processes that affect them, including participation of vulnerable and marginalized groups. |
| Knowledge, Experience, and Risk Perception | Knowledge: Awareness or understanding of risks, including their causes, consequences, and mitigation strategies, including needs and solutions for vulnerable groups.  Experience: Individual or collective past experiences dealing with climate hazards that shape preparedness and response.  Risk Perception: How individuals interpret the severity and likelihood of risks, influencing their preventive or adaptive actions. |
| Implementation Outcomes | |
| Acceptability | The degree to which the CCRIW tool implementation is perceived as appropriate and aligned with local needs. |
| Adoption | Actions taken to trial or implement the CCRIW program. |
| Cost | Financial resources allocated for implementing CCRIW activities or associated action plans. |
| Feasibility | The likelihood that the CCRIW program can be successfully implemented within the given context. |
| Fidelity | The extent to which CCRIW implementation adheres to established guidelines and protocols. |
| Sustainability | The capacity of the CCRIW program to be maintained over time and generate long-term benefits. |
| Intervention Outcomes | |
| Household-Level Climate-Resilient WASH Access | Households gain access to inclusive and resilient WASH services. |

# **Bibliography**

[Apatinga, G. A., Schuster‐Wallace, C. J., & Dickson‐Anderson, S. E. (2022). A conceptual framework for gender and climate mainstreaming to mitigate water inaccessibility in ruralsub‐Saharan Africa. *Wiley Interdisciplinary Reviews: Water*, *9*(4). https://doi.org/10.1002/wat2.1591](https://sciwheel.com/work/bibliography/16578405)

[Howard, G., Nijhawan, A., Flint, A., Baidya, M., Pregnolato, M., Ghimire, A., Poudel, M., Lo, E., Sharma, S., Mengustu, B., Ayele, D. M., Geremew, A., & Wondim, T. (2021). The how tough is WASH framework for assessing the climate resilience of water and sanitation. *Npj Clean Water*, *4*(1), 39. https://doi.org/10.1038/s41545-021-00130-5](https://sciwheel.com/work/bibliography/13542930)

[Megaw, T., Kohlitz, J., Gero, A., & Chong, J. (2020). *Understanding and Responding to ClimateChange Impacts in Inclusive WASH Programs: A Conceptual Framework*. ISF-UTS.](https://sciwheel.com/work/bibliography/16547863)

[Proctor, E. K., Silmere, H., Raghavan, R., Hovmand, P., Aarons, G., Bunger, A., Griffey, R., & Hensley, M. (2011). Outcomes for implementation research: conceptual distinctions, measurement challenges, and research agenda. *Administration and Policy in Mental Health*, *38*(2), 65–76. https://doi.org/10.1007/s10488-010-0319-7](https://sciwheel.com/work/bibliography/650981)
