# Supplementary material for: Implementing a climate-resilient and inclusive WASH guidance in Indonesia: A mixed-methods case study
Source: PLoS One. 2026 Sep 28;21(9):e0359266. doi: 10.1371/journal.pone.0359266 (PMC13618932; doi:10.1371/journal.pone.0359266)
Supplement: Supplementary file 2 [file pone.0359266.s002.docx]

**Monitoring Protocol**

**Inclusive and Climate-Resilient Household Water and Blackwater/Toilet Facilities**

| District/Regency (Kabupaten) |  |
| --- | --- |
| Subdistrict (Kecamatan) |  |
| Village/Sub-village (Desa/Kelurahan) |  |
| Hamlet (Dusun) |  |
| Household Head Name (Nama KK) |  |
| Date (Tanggal) |  |
| Monitoring and Evaluation Officer Name (Nama Petugas Monev) |  |

Number of household members (Jumlah anggota keluarga)

| Number of household members (fill in counts) | | | | | | | | |
| --- | --- | --- | --- | --- | --- | --- | --- | --- |
|  | Without disability (Non-dis) | | | | With disability (Dengan dis) | | | |
|  | Children | | Adults | | Children | | Adults | |
|  | M | F | M | F | M | F | M | F |

1. Identification of Facilities and Facility Modifications

Use the question guide sheet (Appendix) to see examples and explanations.

1.1 Type of facility built/rehabilitated/modified

| Water | Blackwater/Toilet | Handwashing with Soap | Greywater |
| --- | --- | --- | --- |
|  |  |  |  |
|  |  |  |  |
|  |  |  |  |

1.2 What design/modification was made to make this facility more inclusive and/or climate resilient?

| Water | Blackwater/Toilet | Handwashing with Soap | Greywater |
| --- | --- | --- | --- |
|  |  |  |  |
|  |  |  |  |
|  |  |  |  |

2. Checklist for assessing the route to the facility

Tick the appropriate answer and add notes if needed. Use the question guide sheet (Appendix) for examples.

| Question | Water | | Blackwater/Toilet | | Handwashing | | Greywater | | Notes |
| --- | --- | --- | --- | --- | --- | --- | --- | --- | --- |
|  | Yes | No | Yes | No | Yes | No | Yes | No |  |
| 2.1 Route is safe and easy to use. |  |  |  |  |  |  |  |  |  |
| 2.2 Adequate lighting on the route. |  |  |  |  |  |  |  |  |  |
| 2.3 Route has guides/handrails/wayfinding aids. |  |  |  |  |  |  |  |  |  |
| 2.4 Facility is within 5 m of the user's home/main public facility. |  |  |  |  |  |  |  |  |  |
| 2.5 Facility accessible during rain/flooding/after flooding. |  |  |  |  |  |  |  |  |  |
| 2.6 All users (including persons with disabilities) can access independently. |  |  |  |  |  |  |  |  |  |
| 2.7 Similar facility within about 500 m if the main facility is damaged/unusable. |  |  |  |  |  |  |  |  |  |

3. Checklist for assessing facility use

| Question | Water | | Blackwater/Toilet | | Handwashing | | Greywater | | Notes |
| --- | --- | --- | --- | --- | --- | --- | --- | --- | --- |
|  | Yes | No | Yes | No | Yes | No | Yes | No |  |
| 3.1 Facility has adequate access for all users to enter. |  |  |  |  |  |  |  |  |  |
| 3.2 Supporting features allow comfortable use. |  |  |  |  |  |  |  |  |  |
| 3.3 Adequate safety and security features are in place. |  |  |  |  |  |  |  |  |  |
| 3.4 Facility space is sufficient for all users. |  |  |  |  |  |  |  |  |  |
| 3.5 Lighting is sufficient in the facility. |  |  |  |  |  |  |  |  |  |
| 3.6 Facility is structurally sound and withstands climate hazards. |  |  |  |  |  |  |  |  |  |
| 3.7 Facility can be used during rain/flooding/dry season. |  |  |  |  |  |  |  |  |  |
| 3.8 Cleanliness is maintained throughout seasons. |  |  |  |  |  |  |  |  |  |
| 3.9 Liquid waste connects to a drain leading to a soak pit/infiltration pit. |  |  |  |  |  |  |  |  |  |

Appendix: Question Guide

This explanation serves as guidance/examples. Many other measures can be taken to build climate resilience or make facilities accessible using local resources and knowledge that are not yet captured here.

| Q | Explanation |
| --- | --- |
| 1.1 | Water facilities include any water infrastructure used for drinking needs, such as: - Water sources (e.g., bore well, dug well, piped supply) - Water storage (e.g., tank, water basin) - Drinking water facilities  Blackwater/Toilet facilities support defecation/urination activities and may include: - Superstructure (e.g., toilet building, including walls and roof) - Middle structure (e.g., toilet pan) - Substructure (e.g., all parts of the septic tank)  Handwashing with Soap facilities may include: - Sink with running water and soap - Bucket with dipper and soap - Container fitted with a tap and soap - Tippy tap, or other handwashing facilities with running water and soap  Greywater management facilities may include: - Soak pit/infiltration pit - Greywater drainage channel  - Ditch/drain/culvert |
| 1.2 | In general, modifications can include:  Climate resilience: a. Adding safety equipment/facilities (e.g., building embankments, raising the facility elevation to avoid disasters) b. Changing facility dimensions; selecting hazard-resistant materials; etc.  Accessibility: c. Changing the facility structure (e.g., toilet/tap height, door width, door swing direction) d. Adding safety/access features (e.g., ramps, handrails, rough-textured flooring, direction signs) e. Relocating facilities to areas that are not disaster-prone  Examples of modifications for water facilities (not limited to): Climate resilience: a. Increasing water storage capacity during water scarcity b. Providing drinking water for students during the dry season c. etc. Accessibility: d. Changing the stand/height of drinking-water storage so it can be accessed by everyone e. etc.  Examples of modifications for Blackwater/Toilet facilities (not limited to): Climate resilience: a. Relocating the toilet to a safer location away from disaster risks (e.g., landslides, floods, strong winds) b. Upgrading the toilet substructure from an unlined pit (cubluk) to a septic tank sealed on all four sides c. Raising the septic-tank vent pipe to prevent floodwater from entering d. Raising the toilet elevation above average flood levels e. etc. Accessibility: f. Installing guidance features to the toilet (e.g., guiding blocks, handrails, or guide ropes) to help persons with disabilities/older adults reach the toilet g. Installing a ramp (sloped surface) to the toilet for wheelchair users h. Installing safety features in the toilet such as grab bars i. Widening the toilet door so wheelchair users can enter j. etc.  Examples of modifications for CTPS facilities (not limited to): Climate resilience: a. Relocating the CTPS station to a safer location away from disaster risks (e.g., landslides, floods, strong winds) b. etc. Accessibility: c. Installing lever-handle taps instead of twist taps d. Installing guidance features to the CTPS station (e.g., guiding blocks, handrails, or guide ropes) to help persons with disabilities/older adults reach the station e. Installing a ramp (sloped surface) for wheelchair users f. Installing safety features such as grab bars g. etc.  Examples of modifications for Greywater management facilities (not limited to): Climate resilience: a. Building soak pits/infiltration wells b. Maintaining and cleaning drains/culverts/ditches Accessibility: c. etc. |

***Additional guidance for checklist questions***

2.1. Route is safe and easy to use: no obstacles (e.g., large rocks, large holes, many steep steps) that could cause falls or injuries. The route is not steep and not slippery.

2.2. Adequate lighting is available along the route, especially at night.

2.3. Route includes guidance for persons with disabilities (e.g., handrails, guide ropes, guiding blocks) and is wide enough for wheelchair users.

2.4. Distance from water, sanitation, and CTPS facilities to the user's home/main public facility is within 5 m.

2.5. Facility remains usable during rain/flooding or after flooding: the route is not slippery, not muddy, and not waterlogged.

2.6. All users (including persons with disabilities) can access independently without assistance.

2.7. Another similar water and Blackwater/Toiletfacility exists within about 500 m if the main facility is damaged and cannot be used.

3.1. Adequate access for all users: doorways wide enough for wheelchairs, door swing does not obstruct, and there is a level platform in front of the door.

3.2. Supporting features: appropriate tap height, lever-type tap handles, suitable toilet height/design, menstrual hygiene management (MKM) facilities, etc.

3.3. Safety and security: rough-textured flooring, lockable doors, grab bars, no openings that allow easy viewing from outside, covers on waste bins/Greywater drains, etc.

3.4. Sufficient space for all users, including space for wheelchair users to turn.

3.5. Sufficient lighting, either natural or electric.

3.6. Structurally sound and able to withstand climate hazards (not easily collapsed; no rotted walls/roof).

3.7. Usable during rain/flooding:

Water: source not contaminated by floodwater/sewage/waste/drain water; source not damaged.
Wastewater:

- Toilet (blackwater): toilet usable and not inundated; toilet not damaged.
- Greywater: Greywater remains contained and does not overflow.

Handwashing: station usable, not inundated, water and soap available.
3.8. Cleanliness maintained across seasons: no unpleasant smell in dry season; taps still have water; toilets can be flushed and are not smelly; CTPS stations remain usable.
