# Supplementary material for: Implementing a climate-resilient and inclusive WASH guidance in Indonesia: A mixed-methods case study
Source: PLoS One. 2026 Sep 28;21(9):e0359266. doi: 10.1371/journal.pone.0359266 (PMC13618932; doi:10.1371/journal.pone.0359266)
Supplement: Supplementary file 3 [file pone.0359266.s003.docx]

**Key Informant Interview and Focus Group Discussion Protocols**

# 1. Overview

| **Method** | **Target participants** | **Approx. duration** | **Recording and platform** |
| --- | --- | --- | --- |
| KII | Household members; Village Heads/Officials; WASH Team members | About 60 minutes | Audio recording |
| FGD | Plan International Indonesia Water for Women field staff | About 60 minutes | Video recording; Zoom and Miro |

The protocols use the following terms consistently:

- Climate hazards include floods, droughts, landslides, and sea-level rise. Inclusive WASH facilities and services are those that can be accessed and used by everyone.
- The five Community Based WASH pillars refer to ending open defecation, handwashing with soap, household drinking-water and food management, solid-waste management, and domestic-wastewater management.

Notes:

- Ask the main questions in the order presented. Use probes only when clarification or additional detail is needed.
- Adapt phrasing to the participant's role and local terminology without changing the intended meaning.
- At the indicated points, show the visual reminder covering WASH/Community Based WASH, climate hazards, and inclusion.
- Offer any reasonable accommodation needed for a participant to take part comfortably.

# Part I. Combined Key Informant Interview Protocol

**Target participants:** Household members, Village Heads/Officials, and WASH Team members.

## 2. KII administration

| **Field** | **Record** |
| --- | --- |
| **Interviewer** | ____________________________________________ |
| **Date** | ____________________________________________ |
| **District/municipality** | ____________________________________________ |
| **Village** | ____________________________________________ |
| **Participant group** | ☐ Household member ☐ Village Head/Official ☐ WASH Team |
| **Participant gender** | ____________________________________________ |
| **Participant reports a disability** | ☐ Yes ☐ No ☐ Prefer not to answer |
| **Type of disability, if disclosed** | ____________________________________________ |
| **Job title, if applicable** | ____________________________________________ |
| **Years in current position, if applicable** | ____________________________________________ |
| **Years working in the sector, if applicable** | ____________________________________________ |

## 2.1 Opening script

My name is __________, and I am a researcher from __________. This study examines the factors that influence climate-change responses in the implementation of inclusive water, sanitation, and hygiene programs in Indonesia. The study will help us understand the factors that enable or hinder climate-resilient and inclusive WASH services and village-level program decision-making.

Today, we would like to learn about your experiences with climate-resilient and inclusive WASH. In this discussion, WASH includes water access and the five Community Based WASH pillars, including solid-waste management, household drinking-water and food hygiene, and domestic-wastewater management. Please keep all of these components in mind when answering.

During this interview, I may use the terms extreme weather event and climate hazard. Examples include floods, landslides, droughts, and sea-level rise. Inclusive WASH facilities are facilities that everyone can access and use.

We are interested in your experiences and perceptions. This interview will take approximately one hour and, with your permission, will be audio-recorded. The recording will be stored securely, transcribed, retained for one year, and then destroyed.

Your participation is entirely voluntary. You may decline to participate, skip any question, or stop the interview at any time without penalty. The information you provide will be kept confidential. If you need any support or accommodation to participate comfortably, please let us know.

We appreciate honest answers because they will help us develop better recommendations about the program. Your responses will not affect whether your village receives grants or other programs in the future. Please read and sign the consent form before we begin.

**KII-C1. Do you have any questions about the interview?**

**Interviewer action:** Confirm consent, then state: “I will begin the recording now.”

## 3. Section 1: Participant background and CCRIW participation

**KII-1.1 Please describe your role in the village.**

**KII-1.2 How long have you lived in this village?**

**KII-1.3 Have you participated in any activities related to climate-resilient and inclusive WASH?**

**Probe:** Do you remember the one-day climate-change workshop conducted by Plan International Indonesia, which included mapping, a transect walk, and group discussion?

**Probe:** What was your role in the workshop?

**Probe:** What was your overall impression of the workshop?

## 4. Section 2: Experiences and perceptions of climate-resilient and inclusive WASH

**Facilitator prompt:** Show the visual reminder covering WASH/Community Based WASH, climate hazards, and inclusion. Continue to remind participants of these concepts when necessary.

### 4.1 Environmental and technical factors

**KII-2.1 What are the main challenges to implementing the five Community Based WASH pillars and ensuring water access in this village?**

**KII-2.2 Which extreme weather events or climate hazards commonly affect the village?**

**Probe:** Floods, droughts, landslides, or sea-level rise.

**KII-2.3 How do the challenges of providing water access and implementing the five Community Based WASH pillars change during extreme weather events or climate hazards?**

**Probe:** Are water and sanitation facilities damaged, disrupted, or able to remain functional during these events?

**Probe:** Which design features help maintain water and Community Based WASH services during climate hazards?

**Probe:** How does infrastructure design account for local topography, land use, and the hazards that occur most often?

**Probe:** Examples may include raised and covered wells or toilets in flood-prone areas, reuse of washing water for toilet flushing in dry areas, or protective embankments near WASH facilities in landslide-prone areas.

**KII-2.4 How does the community respond to these challenges?**

**Probe:** Which operation, management, and maintenance practices are most effective for keeping WASH facilities usable during climate hazards?

### 4.2 WASH management

**KII-2.5 Please describe the current organizational structure of the community water-management group and the WASH Team.**

**KII-2.6 How are water-management and WASH Team roles and responsibilities distributed among community members?**

**Probe:** Do women and persons with disabilities participate in management and leadership roles? Why or why not?

**KII-2.7 How do the community water-management group and WASH Team respond to climate hazards?**

**Probe:** How quickly do they repair damaged facilities or take preventive action, and what explains the speed of their response?

### 4.3 Governance and community engagement

**KII-2.8 How would you describe community involvement in WASH-related decision-making?**

**Probe:** Do the decision-making process and the people making decisions change after a climate hazard? How?

**Probe:** Who has the greatest influence on community decisions after a climate hazard, and why?

**Probe:** Do social or cultural norms influence these changes? If so, how?

**KII-2.9 Please provide an example of an effective community process, such as communication or coordination, that addressed WASH problems during a climate hazard while maintaining equitable access.**

**KII-2.10 What does the community do to ensure equitable participation in decisions about climate-resilient and inclusive WASH?**

### 4.4 Institutional support

**KII-2.11 What government support has the community received to maintain access to WASH services during climate hazards, including access for persons with disabilities?**

**Probe:** Please describe a successful collaboration between the community and government that helped maintain inclusive WASH access during a climate hazard.

**KII-2.12 How does the community communicate and coordinate with government officials about climate-resilient and inclusive WASH?**

**Probe:** What are the main challenges to obtaining government support?

**Facilitator prompt:** Show the visual reminder covering WASH/Community Based WASH, climate hazards, and inclusion.

### 4.5 Agency, power, and participation

**KII-2.13 Which characteristics, such as capacity, gender, disability, age, or ethnicity, influence participation in village WASH activities?**

**Probe:** What barriers prevent people from participating in community decision-making?

**KII-2.14 Who are the key people or groups with decision-making power over WASH services that should be accessible to everyone?**

**Probe:** Do imbalances in roles or power affect the effectiveness of inclusive WASH services?

**Probe:** How do these imbalances affect access for persons with disabilities and the community's response to climate-related WASH impacts?

### 4.6 Vulnerability and inclusion

**KII-2.15 How do factors such as disability, gender, age, and poverty affect individual access to inclusive WASH services?**

**Probe:** What specific WASH challenges are experienced by women, persons with disabilities, older people, children, or households experiencing poverty?

**Probe:** What measures support people who may be especially vulnerable during climate-related events?

**Probe:** Do social norms and cultural practices reduce or increase these groups' vulnerability? How?

### 4.7 Knowledge and practical capacity

**KII-2.16 How would you describe general awareness of climate-change impacts in this village?**

**KII-2.17 What local or traditional knowledge and practices help reduce climate-hazard risks to WASH access?**

**KII-2.18 What practical capacities do people in the community have to respond to climate impacts and support inclusive sanitation access?**

**Probe:** What prevents individuals or groups from acting on these capacities?

**Probe:** How do these barriers affect the community's overall capacity to respond to climate change and maintain inclusive sanitation access?

**KII-2.19 *[Household members]* How have you applied your knowledge to make household WASH services more climate-resilient and accessible to all household members, including older people, pregnant women, and children?**

### 4.8 Risk perceptions and concerns

**KII-2.20 *[Village Heads/Officials and WASH Team members]* How would you describe community attitudes toward the risks that climate hazards pose to WASH services?**

**Probe:** How do risk attitudes affect decisions and actions related to climate adaptation and mitigation, including actions affecting groups that may be especially vulnerable?

**Probe:** What concerns among community members or village leaders may limit their ability to act to reduce climate-related damage?

**KII-2.21 *[Household members]* How serious is the threat that climate hazards pose to your household's access to WASH services?**

**Probe:** Please describe a personal experience in which a climate hazard affected your WASH access.

**Probe:** How did this experience affect the actions you took to reduce climate-related damage at home?

**Probe:** What concerns may limit your ability to take protective action?

## 5. Section 3: Implementation outcomes

**Routing:** Ask this section only of Village Heads/Officials and WASH Team members. Ask fidelity questions only of participants who facilitated CCRIW activities.

### 5.1 Acceptability

**KII-3.1 How satisfied are you with the implementation of CCRIW in this community? Why?**

**Probe:** Were any aspects unacceptable or unsatisfactory? If so, why?

### 5.2 Adoption

**KII-3.2 Has the village incorporated CCRIW activities into village programs?**

**Probe:** Were all recommended activities adopted, only some, or none? Consider Community Based WASH mapping, inclusive sanitation audits, gender-equality monitoring, and future planning based on available capacity.

**Probe:** What initial actions were needed for adoption? If useful, compare this process with another activity previously incorporated into village programming.

**Probe:** Which strategies were most effective in encouraging government adoption of CCRIW?

### 5.3 Appropriateness and feasibility

**KII-3.3 How well does CCRIW fit the village's needs for WASH access that remains inclusive during climate hazards? Why?**

**Probe:** What modifications would make CCRIW more appropriate or feasible for this village? Why?

### 5.4 Cost

**KII-3.4 If the village adopted CCRIW activities, what were the financial implications for the community?**

**Probe:** What could make CCRIW implementation more cost-effective?

### 5.5 Fidelity

**KII-3.5 *[CCRIW facilitators]* Please describe what you learned during the CCRIW training.**

**Probe:** How well did you understand the training provided by Plan International Indonesia?

**Probe:** Which parts of the training or implementation should be revised or improved?

**Probe:** What challenges did you experience when facilitating workshops as trained, and what improvements would you recommend?

### 5.6 Sustainability

**KII-3.6 What approaches could help sustain CCRIW activities in this village?**

**Probe:** What support is needed to improve sustainability?

**Probe:** If funding is mentioned, what other support is needed in addition to available village funds?

## 6. Section 4: Intervention outcomes and alternative explanations

**Facilitator prompt:** Show the visual reminder covering WASH/Community Based WASH, climate hazards, and inclusion.

### 6.1 WASH access and facility changes

**KII-4.1 *[Village Heads/Officials and WASH Team members]* How has CCRIW influenced community access to public WASH services that are climate-resilient and inclusive?**

**KII-4.2 *[Household members]* After participating in the CCRIW workshop, did you experience more climate-resilient and inclusive WASH access? Why or why not?**

**KII-4.3 *[Village Heads/Officials]* After participating in the CCRIW workshop, did the village modify existing WASH infrastructure or construct new infrastructure to improve climate resilience? Why or why not?**

**Probe:** What was modified or constructed, and why?

**Probe:** Did these changes help address subsequent climate hazards? Please explain using a relevant example.

**KII-4.4 *[Household members]* After participating in the CCRIW workshop, did your household modify existing WASH facilities or construct new facilities to improve climate resilience? Why or why not?**

**Probe:** What did you modify or construct, and why?

**Probe:** Did these changes help during a subsequent climate hazard? Please explain using a relevant example.

**KII-4.5 Apart from CCRIW, what other factors influenced changes in climate-resilient and inclusive WASH access?**

**Probe:** What was the specific contribution of community outreach before CCRIW activities and advocacy after the activities?

**Probe:** Compared with CCRIW, which activities conducted by Plan International Indonesia, government, or other organizations had the greatest influence?

**Probe:** What was the single most important factor contributing to improved climate-resilient and inclusive WASH facilities?

### 6.2 Village WASH programming

**KII-4.6 *[Village Heads/Officials and WASH Team members]* How has CCRIW influenced village-level planning and implementation of climate-resilient and inclusive WASH programs?**

**Probe:** Apart from CCRIW, what other factors influenced the village's WASH programming?

**Probe:** What was the specific contribution of outreach before CCRIW activities and advocacy afterward?

**Probe:** Compared with CCRIW, which activities conducted by Plan International Indonesia, government, or other organizations had the greatest influence?

**Probe:** What was the single most important factor contributing to improvements in village WASH programming?

### 6.3 Inclusive decision-making

**KII-4.7 What did CCRIW help you understand about who should participate in WASH-related decisions during climate hazards?**

**Probe:** Did this understanding change after the activity? Please provide an example, particularly regarding the involvement of women and persons with disabilities.

**KII-4.8 Did CCRIW influence WASH and climate-resilience decision-making within households or the wider community?**

**Probe:** If yes, how did the process change?

**Probe:** Please provide a specific example.

**KII-4.9 Apart from CCRIW, what other factors influenced awareness of inclusive and climate-resilient decision-making?**

**Probe:** What was the specific contribution of outreach before CCRIW activities and advocacy afterward?

**Probe:** Compared with CCRIW, which activities conducted by Plan International Indonesia, government, or other organizations most affected community awareness?

**Probe:** What was the most influential reason for the change in community awareness?

## 7. Section 5: Closing

**KII-5.1 Is there anything else you would like to share about your experience with CCRIW?**

**Interviewer action:** Thank the participant for their time and stop the recording.

# Part II. Focus Group Discussion Protocol

**Target participants:** Plan International Indonesia Water for Women (CERIA Project) field staff.

## 8. FGD administration

| **Field** | **Record** |
| --- | --- |
| **Facilitator** | ____________________________________________ |
| **Date** | ____________________________________________ |
| **Location or online platform** | ____________________________________________ |
| **Village or program area** | ____________________________________________ |
| **Number of participants** | ____________________________________________ |
| **Miro board link** | ____________________________________________ |

## 8.1 Opening script

My name is __________, and I am a researcher from __________. This study examines the factors that influence climate-change responses in the implementation of inclusive WASH programs in Indonesia. It will help us understand what enables or hinders climate-resilient and inclusive WASH services and village-level decision-making.

Today, we would like to learn from your experiences with climate-resilient and inclusive WASH, including solid-waste management, household drinking-water and food management, and domestic-wastewater management. Please keep these components in mind throughout the discussion.

The discussion will take approximately one hour and, with your permission, will be video-recorded. The recording will be stored securely, transcribed, retained for one year, and then destroyed.

Participation is entirely voluntary. You may decline to participate, skip any question, or leave the discussion at any time without penalty. The information you provide will be kept confidential. If you need any support or accommodation to participate comfortably, please let us know.

We appreciate honest responses because they will help us develop better recommendations about program procedures. Your feedback will not affect whether a village receives assistance or other programs in the future. Please read and sign the consent form before we begin.

**FGD-C1. Do you have any questions about the discussion?**

**Facilitator action:** Confirm consent, then state: “I will begin the recording now.”

## 9. Miro-based activity

Today, we will use Miro as a collaborative platform. Please open the Miro link shared in the Zoom chat and sign in using your email address. To confirm that everyone can use the platform, create a sticky note at the bottom of the board, write your name on it, and indicate that you have joined.

We will use an Outcome Harvesting approach to identify major outcomes and the factors that contributed to them. Please enter your reflections on sticky notes and place each contribution in the relevant box.

**Identify outcomes**: Individually reflect on the outcomes you observed. Write each outcome on a separate sticky note and include your name before each response, for example, “Silvia: [outcome].”

**Group outcomes thematically**: After everyone has contributed, organize the outcomes into emerging themes.

**Identify contributing reasons or factors**: For each outcome, add the reasons or factors that contributed to it. Use arrows to connect each factor to the relevant outcome and label the arrow with your name.

**Group reasons thematically**: Organize the reasons into themes and common factors.

**Sequence the outcomes**: Examine relationships among the outcomes, distinguish intermediate outcomes from final outcomes, and arrange them in a logical sequence.

**Identify supporting evidence**: Identify potential individual or location-based case studies that support the outcomes and contributing factors. Discuss the types of information and general evidence themes that the evaluation should collect.

*Figure S2.1. Example Miro board layout used for the Outcome Harvesting activity.*

## 10. General discussion

### 10.1 Intervention outcomes and alternative explanations

**FGD-1.1 Which outcomes hypothesized in the initial framework were not raised during the Outcome Harvesting activity, and why might they not have been mentioned?**

**FGD-1.2 What were the specific effects of community outreach before CCRIW activities and advocacy after the activities?**

**Probe:** How did pre-activity outreach and post-activity advocacy influence climate-resilient and inclusive WASH access?

**Probe:** What factors outside CCRIW also influenced WASH access?

**Probe:** Compared with CCRIW, which other activities conducted by Plan International Indonesia or government had the greatest influence on climate-resilient WASH access?

**Probe:** What was the most influential reason for changes in climate-resilient and inclusive WASH access?

### 10.2 Implementation outcomes

**FGD-2.1 How satisfied were you with CCRIW implementation in your program area? Why?**

**Probe:** How did acceptability affect the outcomes? What should be changed or improved?

**FGD-2.2 How well did CCRIW fit community needs for WASH access that remains inclusive during climate hazards? Why?**

**Probe:** How did program fit affect the outcomes? What should be changed?

**FGD-2.3 Did participating villages incorporate CCRIW activities into village programs?**

**Probe:** Did villages adopt all recommended activities, only some, or none? Why?

**Probe:** What were the main reasons behind these decisions?

**FGD-2.4 Did cost influence implementation or adoption decisions? Why?**

**FGD-2.5 Which parts of the training or workshops should be revised or improved?**

**Probe:** What changes would be most likely to improve the outcomes?

**FGD-2.6 What approaches could help Village Heads/Officials sustain CCRIW activities?**

**Probe:** What support is needed to improve sustainability?

## 11. FGD closing

**FGD-3.1 Is there anything else you would like to add about CCRIW implementation or outcomes?**

**Facilitator action:** Thank participants, confirm that their Miro contributions have been saved, and stop the recording.
