# Supplementary material for: Implementing a climate-resilient and inclusive WASH guidance in Indonesia: A mixed-methods case study
Source: PLoS One. 2026 Sep 28;21(9):e0359266. doi: 10.1371/journal.pone.0359266 (PMC13618932; doi:10.1371/journal.pone.0359266)
Supplement: Supplementary file 4 [file pone.0359266.s004.docx]

**S4 Appendix. Logistic regression results with village-clustered standard errors**

**Table S4.1. Summary statistics for outcomes and model predictors, n = 1,243**

| **Variable** | **Category or statistic** | **Overall** |
| --- | --- | --- |
| **Panel A. Outcome variables** |  |  |
| Household has at least one climate-resilient WASH facility | No | 1,064 (85.6) |
|  | Yes | 179 (14.4) |
| Household has at least one inclusive-resilient WASH facility | No | 1,076 (86.6) |
|  | Yes | 167 (13.4) |
| Drinking water facility is climate-resilient | No | 1,082 (87.0) |
|  | Yes | 161 (13.0) |
| Drinking water facility is inclusive-resilient | No | 1,097 (88.3) |
|  | Yes | 146 (11.7) |
| Toilet facility is climate-resilient | No | 1,118 (89.9) |
|  | Yes | 125 (10.1) |
| Toilet facility is inclusive-resilient | No | 1,127 (90.7) |
|  | Yes | 116 (9.3) |
| Handwashing facility is climate-resilient | No | 1,136 (91.4) |
|  | Yes | 107 (8.6) |
| Handwashing facility is inclusive-resilient | No | 1,140 (91.7) |
|  | Yes | 103 (8.3) |
| Greywater facility is climate-resilient | No | 1,168 (94.0) |
|  | Yes | 75 (6.0) |
| Greywater facility is inclusive-resilient | No | 1,173 (94.4) |
|  | Yes | 70 (5.6) |
| **Panel B. Household and contextual predictors** |  |  |
| Hazard type | Floods or landslides | 777 (62.5) |
|  | Drought | 350 (28.2) |
|  | Multiple hazards | 116 (9.3) |
| Household size | Mean (SD) | 3.75 (1.86) |
| Household size, standardized | Mean (SD) | 0.07 (0.95) |
| Household has at least one member with disability | No | 1,195 (96.1) |
|  | Yes | 48 (3.9) |
| Household has at least one child | No | 529 (42.6) |
|  | Yes | 714 (57.4) |
| Share of household members who are female | Mean (SD) | 0.51 (0.23) |
| District | Manggarai | 531 (42.7) |
|  | Sumbawa | 712 (57.3) |

*Note.* Values are n (%) for categorical variables and mean (standard deviation) for continuous variables. WASH refers to water, sanitation, and hygiene. The standardized household-size variable was used in the regression models.

**Table S4.2. Distribution of the analysis sample across village clusters**

| **District** | **Village** | **Households, n** | **Analysis sample, %** |
| --- | --- | --- | --- |
| Manggarai | 1 | 76 | 6.1 |
| Manggarai | 2 | 102 | 8.2 |
| Manggarai | 3 | 80 | 6.4 |
| Manggarai | 4 | 46 | 3.7 |
| Manggarai | 5 | 57 | 4.6 |
| Manggarai | 6 | 48 | 3.9 |
| Manggarai | 7 | 72 | 5.8 |
| Manggarai | 8 | 50 | 4.0 |
| Sumbawa | 9 | 189 | 15.2 |
| Sumbawa | 10 | 250 | 20.1 |
| Sumbawa | 11 | 162 | 13.0 |
| Sumbawa | 12 | 111 | 8.9 |
| **Total** |  | **1,243** | **100.0** |

*Note.* The analysis included 12 villages: eight in Manggarai and four in Sumbawa. Village cluster sizes ranged from 46 to 250 households. Village was the clustering unit for CR2 standard errors.

**Table S4.3. Adjusted logistic regression results using village-clustered CR2 standard errors**

| **Predictor** | **β** | **CR2 SE** | **OR (95% CI)** | **Satt. df** | **p value** |
| --- | --- | --- | --- | --- | --- |
| **Household: at least one climate-resilient WASH facility** | | | | | |
| Household size (per 1 SD) | -0.075 | 0.095 | 0.928 (0.740 to 1.163) | 6.93 | 0.458 |
| Any household member with disability | -0.234 | 0.421 | 0.791 (0.205 to 3.057) | 2.95 | 0.617 |
| Any child in household | 0.325 | 0.291 | 1.383 (0.707 to 2.706) | 7.95 | 0.297 |
| Female household share (per 1 SD) | -0.038 | 0.093 | 0.963 (0.771 to 1.203) | 6.51 | 0.696 |
| Drought vs floods/landslides | 0.976 | 2.685 | 2.655 (6.25e-09 to 1.13e+09) | 1.31 | 0.766 |
| Multiple hazards vs floods/landslides | 1.118 | 3.043 | 3.059 (6.02e-07 to 1.55e+07) | 1.71 | 0.754 |
| Sumbawa vs Manggarai | -2.598 | 2.741 | 0.074 (7.62e-06 to 727.285) | 2.75 | 0.419 |
| **Household: at least one inclusive-resilient WASH facility** | | | | | |
| Household size (per 1 SD) | -0.114 | 0.099 | 0.892 (0.707 to 1.126) | 7.09 | 0.286 |
| Any household member with disability | -0.113 | 0.444 | 0.893 (0.216 to 3.685) | 2.98 | 0.815 |
| Any child in household | 0.479 | 0.352 | 1.615 (0.716 to 3.640) | 8.01 | 0.211 |
| Female household share (per 1 SD) | -0.015 | 0.088 | 0.985 (0.797 to 1.217) | 6.53 | 0.867 |
| Drought vs floods/landslides | 0.890 | 2.905 | 2.434 (1.36e-09 to 4.37e+09) | 1.32 | 0.801 |
| Multiple hazards vs floods/landslides | 0.940 | 3.205 | 2.561 (3.25e-07 to 2.01e+07) | 1.75 | 0.800 |
| Sumbawa vs Manggarai | -2.554 | 2.966 | 0.078 (3.98e-06 to 1.52e+03) | 2.78 | 0.457 |
| **Drinking water: climate-resilient** | | | | | |
| Household size (per 1 SD) | -0.113 | 0.097 | 0.893 (0.711 to 1.122) | 7.17 | 0.280 |
| Any household member with disability | -0.077 | 0.483 | 0.926 (0.196 to 4.364) | 2.95 | 0.883 |
| Any child in household | 0.390 | 0.315 | 1.477 (0.715 to 3.050) | 8.01 | 0.250 |
| Female household share (per 1 SD) | -0.063 | 0.084 | 0.939 (0.766 to 1.150) | 6.48 | 0.482 |
| Drought vs floods/landslides | 0.862 | 3.199 | 2.369 (1.46e-10 to 3.85e+10) | 1.32 | 0.824 |
| Multiple hazards vs floods/landslides | 0.745 | 3.434 | 2.106 (1.22e-07 to 3.62e+07) | 1.78 | 0.851 |
| Sumbawa vs Manggarai | -2.567 | 3.253 | 0.077 (1.66e-06 to 3.56e+03) | 2.82 | 0.491 |
| **Drinking water: inclusive-resilient** | | | | | |
| Household size (per 1 SD) | -0.187 | 0.134 | 0.830 (0.605 to 1.138) | 7.23 | 0.206 |
| Any household member with disability | 0.075 | 0.521 | 1.078 (0.203 to 5.734) | 2.96 | 0.895 |
| Any child in household | 0.545 | 0.435 | 1.724 (0.630 to 4.717) | 7.88 | 0.247 |
| Female household share (per 1 SD) | -0.075 | 0.077 | 0.928 (0.770 to 1.119) | 6.29 | 0.369 |
| Drought vs floods/landslides | 0.856 | 3.642 | 2.354 (4.02e-12 to 1.38e+12) | 1.31 | 0.846 |
| Multiple hazards vs floods/landslides | 0.622 | 3.834 | 1.862 (2.08e-08 to 1.66e+08) | 1.81 | 0.887 |
| Sumbawa vs Manggarai | -2.546 | 3.712 | 0.078 (4.22e-07 to 1.46e+04) | 2.87 | 0.544 |
| **Toilet: climate-resilient** | | | | | |
| Household size (per 1 SD) | -0.019 | 0.093 | 0.981 (0.785 to 1.225) | 6.51 | 0.842 |
| Any household member with disability | -0.730 | 0.848 | 0.482 (0.023 to 10.164) | 2.48 | 0.464 |
| Any child in household | 0.429 | 0.291 | 1.536 (0.765 to 3.087) | 6.54 | 0.186 |
| Female household share (per 1 SD) | -0.042 | 0.097 | 0.959 (0.750 to 1.226) | 5.16 | 0.680 |
| Drought vs floods/landslides | 1.151 | 0.447 | 3.161 (0.085 to 116.885) | 1.25 | 0.194 |
| Multiple hazards vs floods/landslides | -0.117 | 1.159 | 0.890 (0.006 to 129.032) | 2.00 | 0.929 |
| Sumbawa vs Manggarai | -3.088 | 0.652 | 0.046 (0.007 to 0.304) | 3.58 | 0.012 |

Table S4.4. Continued

| **Predictor** | **β** | **CR2 SE** | **OR (95% CI)** | **Satt. df** | **p value** |
| --- | --- | --- | --- | --- | --- |
| **Toilet: inclusive-resilient** | | | | | |
| Household size (per 1 SD) | -0.047 | 0.094 | 0.954 (0.761 to 1.197) | 6.36 | 0.636 |
| Any household member with disability | -0.621 | 0.868 | 0.538 (0.024 to 12.293) | 2.47 | 0.536 |
| Any child in household | 0.399 | 0.317 | 1.490 (0.695 to 3.194) | 6.43 | 0.252 |
| Female household share (per 1 SD) | 0.015 | 0.116 | 1.015 (0.754 to 1.365) | 5.07 | 0.903 |
| Drought vs floods/landslides | 1.048 | 0.467 | 2.853 (0.069 to 117.272) | 1.26 | 0.224 |
| Multiple hazards vs floods/landslides | -0.323 | 1.157 | 0.724 (0.005 to 98.565) | 2.03 | 0.806 |
| Sumbawa vs Manggarai | -3.088 | 0.650 | 0.046 (0.007 to 0.301) | 3.60 | 0.012 |
| **Handwashing: climate-resilient** | | | | | |
| Household size (per 1 SD) | -0.130 | 0.112 | 0.878 (0.655 to 1.177) | 4.74 | 0.302 |
| Any household member with disability | -0.370 | 0.695 | 0.691 (0.050 to 9.603) | 2.31 | 0.641 |
| Any child in household | 0.560 | 0.302 | 1.751 (0.808 to 3.793) | 5.08 | 0.122 |
| Female household share (per 1 SD) | -0.081 | 0.088 | 0.922 (0.722 to 1.178) | 3.96 | 0.411 |
| Drought vs floods/landslides | 1.191 | 0.455 | 3.289 (0.026 to 408.742) | 1.08 | 0.217 |
| Multiple hazards vs floods/landslides | -16.256 | 0.047 | 8.71e-08 (4.87e-08 to 1.56e-07) | 1.01 | 0.002 |
| Sumbawa vs Manggarai | -2.931 | 0.644 | 0.053 (0.005 to 0.565) | 2.42 | 0.031 |
| **Handwashing: inclusive-resilient** | | | | | |
| Household size (per 1 SD) | -0.157 | 0.099 | 0.854 (0.659 to 1.108) | 4.71 | 0.178 |
| Any household member with disability | -0.320 | 0.719 | 0.726 (0.047 to 11.156) | 2.31 | 0.694 |
| Any child in household | 0.526 | 0.309 | 1.691 (0.765 to 3.738) | 5.04 | 0.149 |
| Female household share (per 1 SD) | -0.078 | 0.084 | 0.925 (0.731 to 1.171) | 3.91 | 0.409 |
| Drought vs floods/landslides | 1.292 | 0.465 | 3.641 (0.024 to 545.658) | 1.08 | 0.206 |
| Multiple hazards vs floods/landslides | -16.112 | 0.049 | 1.01e-07 (5.46e-08 to 1.85e-07) | 1.01 | 0.002 |
| Sumbawa vs Manggarai | -2.795 | 0.644 | 0.061 (0.005 to 0.728) | 2.27 | 0.039 |
| **Greywater: climate-resilient** | | | | | |
| Household size (per 1 SD) | -0.116 | 0.126 | 0.890 (0.645 to 1.229) | 5.05 | 0.398 |
| Any household member with disability | -0.515 | 0.628 | 0.597 (0.059 to 6.096) | 2.39 | 0.486 |
| Any child in household | 0.753 | 0.468 | 2.122 (0.662 to 6.808) | 5.58 | 0.162 |
| Female household share (per 1 SD) | 0.026 | 0.148 | 1.027 (0.691 to 1.526) | 4.39 | 0.867 |
| Drought vs floods/landslides | 0.673 | 0.652 | 1.960 (0.003 to 1.33e+03) | 1.11 | 0.476 |
| Multiple hazards vs floods/landslides | -16.210 | 0.074 | 9.12e-08 (3.67e-08 to 2.27e-07) | 1.01 | 0.003 |
| Sumbawa vs Manggarai | -2.877 | 0.640 | 0.056 (0.005 to 0.583) | 2.43 | 0.032 |
| **Greywater: inclusive-resilient** | | | | | |
| Household size (per 1 SD) | -0.093 | 0.137 | 0.911 (0.641 to 1.295) | 5.08 | 0.527 |
| Any household member with disability | -0.434 | 0.625 | 0.648 (0.064 to 6.555) | 2.39 | 0.549 |
| Any child in household | 0.751 | 0.537 | 2.119 (0.557 to 8.064) | 5.62 | 0.215 |
| Female household share (per 1 SD) | 0.071 | 0.125 | 1.073 (0.769 to 1.497) | 4.44 | 0.598 |
| Drought vs floods/landslides | 0.739 | 0.616 | 2.093 (0.004 to 1.08e+03) | 1.11 | 0.427 |
| Multiple hazards vs floods/landslides | -16.055 | 0.097 | 1.07e-07 (3.19e-08 to 3.56e-07) | 1.01 | 0.004 |
| Sumbawa vs Manggarai | -2.718 | 0.643 | 0.066 (0.006 to 0.772) | 2.29 | 0.041 |

Note. β = log-odds coefficient; CR2 SE = village-clustered CR2 standard error; OR = adjusted odds ratio; CI = confidence interval; Satt. df = coefficient-specific Satterthwaite degrees of freedom. Each model included 1,243 households clustered within 12 villages and adjusted for all predictors listed in the table. Reference categories were floods or landslides for hazard exposure and Manggarai for district. Household size and female household share were standardized, so their estimates represent a one-standard-deviation increase.

**Table S4.4. Model information and fit statistics**

| **Outcome** | **N** | **Events** | **Clusters** | **Log likelihood** | **AIC** |
| --- | --- | --- | --- | --- | --- |
| Household: at least one climate-resilient WASH facility | 1,243 | 179 | 12 | -377.62 | 771.23 |
| Household: at least one inclusive-resilient WASH facility | 1,243 | 167 | 12 | -368.49 | 752.99 |
| Drinking water: climate-resilient | 1,243 | 161 | 12 | -364.10 | 744.19 |
| Drinking water: inclusive-resilient | 1,243 | 146 | 12 | -344.82 | 705.63 |
| Toilet: climate-resilient | 1,243 | 125 | 12 | -294.67 | 605.34 |
| Toilet: inclusive-resilient | 1,243 | 116 | 12 | -284.46 | 584.93 |
| Handwashing: climate-resilient | 1,243 | 107 | 12 | -252.28 | 520.55 |
| Handwashing: inclusive-resilient | 1,243 | 103 | 12 | -247.41 | 510.83 |
| Greywater: climate-resilient | 1,243 | 75 | 12 | -212.12 | 440.23 |
| Greywater: inclusive-resilient | 1,243 | 70 | 12 | -203.92 | 423.84 |

Note. Each ordinary logistic regression model included seven predictors plus an intercept. Log likelihood, residual deviance, and Akaike information criterion (AIC) describe the maximum-likelihood model fit.

**Table S4.5. Distribution of household hazard exposure by district**

| **District** | **Hazard exposure** | **District sample, N** | **Households, n** | **Within-district, %** |
| --- | --- | --- | --- | --- |
| Manggarai | Floods or landslides | 531 | 76 | 14.3 |
| Manggarai | Drought | 531 | 350 | 65.9 |
| Manggarai | Multiple hazards | 531 | 105 | 19.8 |
| Sumbawa | Floods or landslides | 712 | 701 | 98.5 |
| Sumbawa | Drought | 712 | 0 | 0 |
| Sumbawa | Multiple hazards | 712 | 11 | 1.5 |
