# Supplementary material for: Implementing a climate-resilient and inclusive WASH guidance in Indonesia: A mixed-methods case study
Source: PLoS One. 2026 Sep 28;21(9):e0359266. doi: 10.1371/journal.pone.0359266 (PMC13618932; doi:10.1371/journal.pone.0359266)
Supplement: Supplementary file 5 [file pone.0359266.s009.docx]

**S9 Appendix R Markdown Codes**

````
# Packages

```{r packages}
library(readxl)
library(writexl)
library(dplyr)
library(tidyr)
library(purrr)
library(tibble)
library(knitr)
library(clubSandwich)
```

# Data preparation

## Read and validate the analysis fields

```{r read-data}
data_survey <- read_excel("data_survey.xlsx")

demographic_fields <- paste0("1.4.", 1:8)

required_fields <- c(
  "district", "village", "hazard", "1.7", "A.5",
  demographic_fields,
  "2.A.5", "2.A.6", "2.B.5", "2.B.6",
  "2.C.5", "2.C.6", "2.E.5", "2.E.6",
  "3.A.6", "3.B.6", "3.C.6", "3.E.6"
)

stopifnot(all(required_fields %in% names(data_survey)))
```

## Construct outcomes and household covariates


```{r construct-variables}
outcome_vars <- c(
  "HH_res", "HH_inres",
  "water_res", "water_inres",
  "san_res", "san_inres",
  "HW_res", "HW_inres",
  "wastewtr_res", "wastewtr_inres"
)

cleaned_HH <- data_survey %>%
  mutate(
    action_implemented = `1.7` %in% c("Sudah dilakukan", "Keduanya"),
    modification_documented = !is.na(A.5),
    water_res = as.integer(
      action_implemented & modification_documented & `3.A.6` == 1
    ),
    san_res = as.integer(
      action_implemented & modification_documented & `3.B.6` == 1
    ),
    HW_res = as.integer(
      action_implemented & modification_documented & `3.C.6` == 1
    ),
    wastewtr_res = as.integer(
      action_implemented & modification_documented & `3.E.6` == 1
    ),
    water_inres = as.integer(
      water_res == 1 & (`2.A.5` == 1 | `2.A.6` == 1)
    ),
    san_inres = as.integer(
      san_res == 1 & (`2.B.5` == 1 | `2.B.6` == 1)
    ),
    HW_inres = as.integer(
      HW_res == 1 & (`2.C.5` == 1 | `2.C.6` == 1)
    ),
    wastewtr_inres = as.integer(
      wastewtr_res == 1 & (`2.E.5` == 1 | `2.E.6` == 1)
    ),
    HH_res = as.integer(
      rowSums(across(c(water_res, san_res, HW_res, wastewtr_res))) > 0
    ),
    HH_inres = as.integer(
      rowSums(
        across(c(water_inres, san_inres, HW_inres, wastewtr_inres))
      ) > 0
    )
  ) %>%
  mutate(across(all_of(demographic_fields), ~ replace_na(as.numeric(.x), 0))) %>%
  mutate(
    child_total = `1.4.1` + `1.4.2` + `1.4.5` + `1.4.6`,
    adult_total = `1.4.3` + `1.4.4` + `1.4.7` + `1.4.8`,
    female_total = `1.4.2` + `1.4.4` + `1.4.6` + `1.4.8`,
    disability_total = `1.4.5` + `1.4.6` + `1.4.7` + `1.4.8`,
    hh_size = child_total + adult_total,
    any_child = as.integer(child_total > 0),
    any_disability = as.integer(disability_total > 0),
    female_share = if_else(
      hh_size > 0,
      female_total / hh_size,
      NA_real_
    ),
    disability_share = if_else(
      hh_size > 0,
      disability_total / hh_size,
      NA_real_
    ),
    valid_household_demographics = hh_size > 0,
    district = factor(
      district,
      levels = c("Manggarai", "Sumbawa")
    ),
    hazard = factor(
      as.character(hazard),
      levels = c("1", "2", "3"),
      labels = c(
        "Floods/Landslides",
        "Drought",
        "Multiple hazards"
      )
    ),
    village = factor(village)
  )

model_covariates <- c(
  "hh_size", "any_disability", "any_child",
  "female_share", "hazard", "district", "village"
)

analysis_data <- cleaned_HH %>%
  filter(valid_household_demographics) %>%
  drop_na(all_of(c(outcome_vars, model_covariates))) %>%
  mutate(
    hh_size_z = as.numeric(scale(hh_size)),
    female_share_z = as.numeric(scale(female_share))
  ) %>%
  droplevels()
```

```{r labels, include=FALSE}
outcome_labels <- tribble(
  ~outcome_variable, ~outcome_label, ~outcome_order,
  "HH_res", "Household: at least one climate-resilient WASH facility", 1,
  "HH_inres", "Household: at least one inclusive-resilient WASH facility", 2,
  "water_res", "Drinking water: climate-resilient", 3,
  "water_inres", "Drinking water: inclusive-resilient", 4,
  "san_res", "Toilet: climate-resilient", 5,
  "san_inres", "Toilet: inclusive-resilient", 6,
  "HW_res", "Handwashing: climate-resilient", 7,
  "HW_inres", "Handwashing: inclusive-resilient", 8,
  "wastewtr_res", "Greywater: climate-resilient", 9,
  "wastewtr_inres", "Greywater: inclusive-resilient", 10
)

facility_specs <- tribble(
  ~facility_type, ~resilient_variable, ~inclusive_variable,
  "Drinking Water", "water_res", "water_inres",
  "Toilet", "san_res", "san_inres",
  "Greywater", "wastewtr_res", "wastewtr_inres",
  "Handwashing", "HW_res", "HW_inres"
)
```

## Analysis sample

```{r sample-flow}
valid_composition_n <- sum(cleaned_HH$valid_household_demographics)

sample_flow <- tibble(
  stage = c(
    "Household records imported",
    "Records with valid household composition",
    "Complete records used in every adjusted model"
  ),
  records_remaining = c(
    nrow(data_survey),
    valid_composition_n,
    nrow(analysis_data)
  ),
  records_excluded_at_stage = c(
    0,
    nrow(data_survey) - valid_composition_n,
    valid_composition_n - nrow(analysis_data)
  )
)

kable(sample_flow, caption = "Analysis sample flow")
```

# Descriptive results

## Outcome prevalence

```{r outcome-prevalence}
outcome_prevalence <- analysis_data %>%
  summarise(
    n = n(),
    across(
      all_of(outcome_vars),
      list(
        events = ~ sum(.x == 1),
        prevalence = ~ mean(.x == 1)
      ),
      .names = "{.col}__{.fn}"
    )
  ) %>%
  pivot_longer(
    cols = -n,
    names_to = c("outcome_variable", ".value"),
    names_pattern = "^(.*)__(events|prevalence)$"
  ) %>%
  mutate(
    non_events = n - events,
    percent = 100 * prevalence
  ) %>%
  left_join(
    outcome_labels,
    by = "outcome_variable"
  ) %>%
  arrange(outcome_order) %>%
  select(
    outcome_variable,
    outcome_label,
    n,
    events,
    non_events,
    prevalence,
    percent
  )

outcome_prevalence %>%
  select(
    outcome_label,
    n,
    events,
    non_events,
    percent
  ) %>%
  kable(
    digits = 1,
    col.names = c(
      "Outcome",
      "N",
      "Events",
      "Non-events",
      "Percent"
    ),
    caption = "Prevalence of household and facility outcomes"
  )
```

## Climate hazard distribution by district

```{r district-hazard-distribution}
district_hazard_distribution <- analysis_data %>%
  count(
    district,
    hazard,
    name = "n_households",
    .drop = FALSE
  ) %>%
  group_by(district) %>%
  mutate(
    district_n = sum(n_households),
    percent_within_district =
      100 * n_households / district_n
  ) %>%
  ungroup() %>%
  arrange(
    district,
    hazard
  ) %>%
  select(
    district,
    hazard,
    district_n,
    n_households,
    percent_within_district
  )

district_hazard_distribution %>%
  kable(
    digits = 1,
    col.names = c(
      "District",
      "Hazard",
      "District N",
      "Households",
      "Percent within district"
    ),
    caption = "Climate hazard distribution in Manggarai and Sumbawa"
  )
```

## Aggregate facility table

```{r facility-table}
facility_table <- tibble(
  facility_type = c(
    facility_specs$facility_type,
    "Total Household"
  ),
  resilient_count = colSums(
    analysis_data[
      c(
        facility_specs$resilient_variable,
        "HH_res"
      )
    ]
  ),
  inclusive_resilient_count = colSums(
    analysis_data[
      c(
        facility_specs$inclusive_variable,
        "HH_inres"
      )
    ]
  )
) %>%
  mutate(
    n = nrow(analysis_data),
    resilient_percent = 100 * resilient_count / n,
    inclusive_resilient_percent =
      100 * inclusive_resilient_count / n
  )

kable(
  facility_table,
  digits = 1,
  caption = "Climate-resilient and inclusive-resilient WASH outcomes"
)
```

## Village cluster diagnostics
```{r cluster-diagnostics}
village_cluster_sizes <- analysis_data %>%
  count(
    district,
    village,
    name = "n_households"
  ) %>%
  arrange(
    district,
    as.integer(as.character(village))
  )

village_cluster_size_summary <- village_cluster_sizes %>%
  summarise(
    total_households = sum(n_households),
    n_villages = n(),
    minimum_households = min(n_households),
    median_households = median(n_households),
    maximum_households = max(n_households)
  )

village_outcome_detail <- analysis_data %>%
  select(
    district,
    village,
    all_of(outcome_vars)
  ) %>%
  pivot_longer(
    cols = all_of(outcome_vars),
    names_to = "outcome_variable",
    values_to = "outcome"
  ) %>%
  group_by(
    district,
    village,
    outcome_variable
  ) %>%
  summarise(
    n = n(),
    events = sum(outcome == 1),
    non_events = sum(outcome == 0),
    prevalence = mean(outcome == 1),
    percent = 100 * prevalence,
    .groups = "drop"
  ) %>%
  left_join(
    outcome_labels,
    by = "outcome_variable"
  ) %>%
  arrange(
    district,
    village,
    outcome_order
  )

village_outcome_summary <- village_outcome_detail %>%
  group_by(
    outcome_variable,
    outcome_label,
    outcome_order
  ) %>%
  summarise(
    n = sum(n),
    events = sum(events),
    non_events = sum(non_events),
    villages_with_events = sum(events > 0),
    villages_without_events = sum(events == 0),
    villages_with_only_events = sum(non_events == 0),
    .groups = "drop"
  ) %>%
  arrange(outcome_order)

kable(
  village_cluster_sizes,
  digits = 1,
  caption = "Households per village cluster"
)

kable(
  village_cluster_size_summary,
  digits = 1,
  caption = "Village cluster-size summary"
)

village_outcome_summary %>%
  select(
    outcome_label,
    n,
    events,
    non_events,
    villages_with_events,
    villages_without_events,
    villages_with_only_events
  ) %>%
  kable(
    caption = "Outcome variation across village clusters"
  )
```

## Household demographic summaries

```{r demographic-summaries}
make_demographic_summary <- function(data, outcome_var) {
  data %>%
    group_by(
      outcome_status = factor(
        .data[[outcome_var]],
        levels = c(0, 1),
        labels = c("No", "Yes")
      )
    ) %>%
    summarise(
      n = n(),
      mean_hh_size = mean(hh_size),
      median_hh_size = median(hh_size),
      mean_children = mean(child_total),
      mean_adults = mean(adult_total),
      percent_with_child = 100 * mean(any_child == 1),
      percent_with_disability = 100 * mean(any_disability == 1),
      mean_female_percent = 100 * mean(female_share),
      mean_disability_percent = 100 * mean(disability_share),
      .groups = "drop"
    ) %>%
    mutate(outcome_variable = outcome_var, .before = 1)
}

demographic_summary <- map_dfr(
  c("HH_res", "HH_inres"),
  ~ make_demographic_summary(analysis_data, .x)
) %>%
  left_join(outcome_labels, by = "outcome_variable") %>%
  arrange(outcome_order, outcome_status)

demographic_summary %>%
  select(
    outcome_label, outcome_status, n, mean_hh_size,
    percent_with_child, percent_with_disability,
    mean_female_percent, mean_disability_percent
  ) %>%
  kable(
    digits = 1,
    caption = "Household characteristics by household-level outcome"
  )
```

## Facility outcomes by child and disability status

```{r facility-breakdowns}
make_facility_data <- function(
  data,
  facility_name,
  resilient_variable,
  inclusive_variable
) {
  data %>%
    transmute(
      facility_type = facility_name,
      resilient = .data[[resilient_variable]],
      inclusive_resilient = .data[[inclusive_variable]],
      any_child,
      any_disability
    )
}

facility_long <- bind_rows(
  make_facility_data(
    analysis_data,
    "Drinking Water",
    "water_res",
    "water_inres"
  ),
  make_facility_data(
    analysis_data,
    "Toilet",
    "san_res",
    "san_inres"
  ),
  make_facility_data(
    analysis_data,
    "Greywater",
    "wastewtr_res",
    "wastewtr_inres"
  ),
  make_facility_data(
    analysis_data,
    "Handwashing",
    "HW_res",
    "HW_inres"
  )
)

summarise_facility_by <- function(data, group_var) {
  data %>%
    group_by(
      facility_type,
      group_value = .data[[group_var]]
    ) %>%
    summarise(
      n = n(),
      resilient_count = sum(resilient),
      resilient_percent = 100 * mean(resilient == 1),
      inclusive_resilient_count = sum(inclusive_resilient),
      inclusive_resilient_percent =
        100 * mean(inclusive_resilient == 1),
      .groups = "drop"
    ) %>%
    mutate(
      group_variable = group_var,
      group_value = factor(
        group_value,
        levels = c(0, 1),
        labels = c("No", "Yes")
      ),
      .before = 1
    )
}

facility_by_disability <- summarise_facility_by(
  facility_long,
  "any_disability"
)

facility_by_child <- summarise_facility_by(
  facility_long,
  "any_child"
)

kable(
  facility_by_disability,
  digits = 1,
  caption = "Facility outcomes by household disability status"
)

kable(
  facility_by_child,
  digits = 1,
  caption = "Facility outcomes by household child status"
)
```

## Regression summary statistics

```{r regression-summary}
binary_variable_labels <- bind_rows(
  outcome_labels %>%
    transmute(
      variable = outcome_variable,
      label = outcome_label,
      type = "Outcome",
      display_order = outcome_order
    ),
  tribble(
    ~variable, ~label, ~type, ~display_order,
    "any_disability", "Household has at least one member with disability",
      "Predictor", 11,
    "any_child", "Household has at least one child",
      "Predictor", 12
  )
)

binary_summary <- analysis_data %>%
  select(all_of(binary_variable_labels$variable)) %>%
  pivot_longer(
    everything(),
    names_to = "variable",
    values_to = "category_code"
  ) %>%
  mutate(
    category = factor(
      category_code,
      levels = c(0, 1),
      labels = c("No", "Yes")
    )
  ) %>%
  count(variable, category, .drop = FALSE) %>%
  group_by(variable) %>%
  mutate(percent = 100 * n / sum(n)) %>%
  ungroup() %>%
  left_join(binary_variable_labels, by = "variable") %>%
  transmute(
    type,
    variable,
    label,
    category = as.character(category),
    n,
    percent,
    mean = NA_real_,
    sd = NA_real_,
    min = NA_real_,
    max = NA_real_,
    display_order
  )

summarise_factor <- function(data, variable, label, display_order) {
  data %>%
    count(category = .data[[variable]], .drop = FALSE) %>%
    mutate(
      type = "Predictor",
      variable = variable,
      label = label,
      percent = 100 * n / sum(n),
      mean = NA_real_,
      sd = NA_real_,
      min = NA_real_,
      max = NA_real_,
      display_order = display_order
    ) %>%
    transmute(
      type,
      variable,
      label,
      category = as.character(category),
      n,
      percent,
      mean,
      sd,
      min,
      max,
      display_order
    )
}

factor_summary <- bind_rows(
  summarise_factor(
    analysis_data,
    "district",
    "District",
    15
  ),
  summarise_factor(
    analysis_data,
    "hazard",
    "Climate hazard",
    16
  )
)

continuous_specs <- tribble(
  ~variable, ~label, ~display_order,
  "hh_size_z", "Household size, standardized", 13,
  "female_share", "Share of household members who are female", 14
)

continuous_summary <- map_dfr(
  seq_len(nrow(continuous_specs)),
  function(i) {
    variable <- continuous_specs$variable[[i]]
    values <- analysis_data[[variable]]

    tibble(
      type = "Predictor",
      variable = variable,
      label = continuous_specs$label[[i]],
      category = "Mean (SD); range",
      n = sum(!is.na(values)),
      percent = NA_real_,
      mean = mean(values, na.rm = TRUE),
      sd = sd(values, na.rm = TRUE),
      min = min(values, na.rm = TRUE),
      max = max(values, na.rm = TRUE),
      display_order = continuous_specs$display_order[[i]]
    )
  }
)

summary_stats <- bind_rows(
  binary_summary,
  continuous_summary,
  factor_summary
) %>%
  arrange(display_order, category)

summary_stats_display <- summary_stats %>%
  mutate(
    summary = case_when(
      !is.na(percent) ~ sprintf("%s (%.1f%%)", n, percent),
      TRUE ~ sprintf(
        "%.2f (%.2f); %.2f to %.2f",
        mean, sd, min, max
      )
    )
  ) %>%
  select(type, label, category, summary)

kable(
  summary_stats_display,
  col.names = c("Type", "Variable", "Category", "Summary"),
  caption = "Summary statistics for modeled outcomes and predictors"
)
```

# Primary adjusted analysis

## Ordinary logistic regression with village-clustered CR2 inference


```{r fit-cr2}
fixed_terms <- c(
  "hh_size_z",
  "any_disability",
  "any_child",
  "female_share_z",
  "hazard",
  "district"
)

term_labels <- tribble(
  ~term, ~term_label, ~term_order,
  "hh_size_z", "Household size (per 1 SD)", 1,
  "any_disability", "Any household member with disability", 2,
  "any_child", "Any child in household", 3,
  "female_share_z", "Female household share (per 1 SD)", 4,
  "hazardDrought", "Drought vs floods/landslides", 5,
  "hazardMultiple hazards", "Multiple hazards vs floods/landslides", 6,
  "districtSumbawa", "Sumbawa vs Manggarai", 7
)

glm_formula <- function(outcome_var) {
  as.formula(
    paste(
      outcome_var,
      "~",
      paste(fixed_terms, collapse = " + ")
    )
  )
}

fit_village_cr2 <- function(outcome_var) {
  model <- glm(
    formula = glm_formula(outcome_var),
    data = analysis_data,
    family = binomial(link = "logit")
  )

  cr2_covariance <- vcovCR(
    model,
    cluster = analysis_data$village,
    type = "CR2"
  )

  cr2_inference <- conf_int(
    model,
    vcov = cr2_covariance,
    level = 0.95,
    test = "Satterthwaite",
    p_values = TRUE
  )

  fixed_effects <- as_tibble(cr2_inference) %>%
    transmute(
      outcome_variable = outcome_var,
      term = as.character(.data$Coef),
      log_odds = .data$beta,
      estimate = exp(.data$beta),
      conf.low = exp(.data$CI_L),
      conf.high = exp(.data$CI_U),
      std.error = .data$SE,
      degrees_of_freedom = .data$df,
      p.value = .data$p_val,
      n = nobs(model),
      events = sum(analysis_data[[outcome_var]] == 1),
      non_events = sum(analysis_data[[outcome_var]] == 0),
      n_clusters = n_distinct(analysis_data$village),
      model_type = paste(
        "Ordinary logistic regression with",
        "village-clustered CR2 inference"
      )
    )

  list(
    model = model,
    fixed_effects = fixed_effects
  )
}

cr2_objects <- map(outcome_vars, fit_village_cr2) %>%
  set_names(outcome_vars)

cr2_results <- map_dfr(
  cr2_objects,
  ~ .x$fixed_effects
) %>%
  filter(term != "(Intercept)") %>%
  left_join(outcome_labels, by = "outcome_variable") %>%
  left_join(term_labels, by = "term") %>%
  arrange(outcome_order, term_order)
```

```{r display-cr2}
cr2_results %>%
  select(
    outcome_label,
    term_label,
    estimate,
    conf.low,
    conf.high,
    degrees_of_freedom,
    p.value,
    n,
    events,
    n_clusters
  ) %>%
  kable(
    digits = 3,
    col.names = c(
      "Outcome",
      "Predictor",
      "Adjusted OR",
      "95% CI low",
      "95% CI high",
      "Satterthwaite df",
      "p value",
      "N",
      "Events",
      "Villages"
    ),
    caption = paste(
      "Ordinary logistic regression with village-clustered CR2",
      "standard errors and Satterthwaite corrections"
    )
  )
```

# Export results

```{r export-results}
excel_ready <- function(data) {
  data %>%
    mutate(across(where(is.factor), as.character))
}

results_to_export <- list(
  sample_flow = sample_flow,
  outcome_prevalence = outcome_prevalence,
  district_hazard_distribution = district_hazard_distribution,
  facility_table = facility_table,
  HH_demo_summary = demographic_summary,
  regression_summary_stats = summary_stats,
  facility_by_disability = facility_by_disability,
  facility_by_child = facility_by_child,
  village_cluster_sizes = village_cluster_sizes,
  village_cluster_summary = village_cluster_size_summary,
  village_outcome_detail = village_outcome_detail,
  village_outcome_summary = village_outcome_summary,
  CR2_adjusted_ORs = cr2_results
)

write_xlsx(
  map(results_to_export, excel_ready),
  "HH_facility_demographic_CR2.xlsx"
)
```
````
