# Supplementary material for: Implementing a climate-resilient and inclusive WASH guidance in Indonesia: A mixed-methods case study
Source: PLoS One. 2026 Sep 28;21(9):e0359266. doi: 10.1371/journal.pone.0359266 (PMC13618932; doi:10.1371/journal.pone.0359266)
Supplement: Supplementary file 6 [file pone.0359266.s006.zip › S6 Appendix Transcripts/FGD-01_Deidentified_English.docx]

FGD

Moderator: Yes, and during the process, I would like to ask FGD Participant 06 to take notes on this discussion so that it will be easier for us to summarize. Before we begin, I have shared a link. Can everyone access Miro?

FGD Participant 01: Miro?

Moderator: The link I shared in the chat. It resembles a game board, although I do not think that game board is used anymore.

Moderator: (...) Is it open?

Moderator: What about everyone other than FGD Participant 01?

Moderator: (...) Have you been able to enter?

Moderator: (...) Does anyone need assistance?

FGD Participant 02: We are trying to enter. We can see a dot near FGD Participant 01 but we want to know exactly where to go.

FGD Participant 02: FGD Participant 03 can enter.

Moderator: Okay, we will wait until everyone has entered.

Moderator: I will explain it now. I can see that FGD Participant 02 and FGD Participant 03 have entered.

Moderator: Okay, I will share my screen.

Moderator: Did FGD Participant 02 enter directly using an email address?

FGD Participant 02: Yes, I used my email.

Moderator: Who has not entered? During the discussion, you can type here, delete text, and connect this arrow to that item. When you write a name, for example, you can enlarge it if it is too small so we know who wrote it. You can make the letters smaller or larger here. Okay, who has not entered?

Moderator: Oh, do you have to install Miro? Just use this one. If FGD Participant 06 cannot enter, that is all right, as long as the Plan colleagues can. FGD Participant 07 and FGD Participant 02 have entered, along with the others.

FGD Participant 04: I will check and try to enter using my email, but I have forgotten my password.

Moderator: Your email password? (laughing)

Moderator: FGD Participant 07 FGD Participant 03 and FGD Participant 02 did you enter directly with a password or without one?

FGD Participant 02: I clicked the Google link, and it opened directly. At the upper-right side, where our Gmail account appears, there is a question asking whether to use Miro. I clicked there, and it opened.

Moderator: Okay, so it uses Gmail if you have a Gmail account.

FGD Participant 03: A six-digit code was also sent here, and we had to enter it.

(...)

Moderator: What about the others? FGD Participant 01 FGD Participant 08 and FGD Participant 05 have you entered?

Moderator: Okay, (...) everyone is here. Has anyone not entered? FGD Participant 01 has entered. Okay.

Moderator: FGD Participant 03 is here. FGD Participant 02 FGD Participant 08 FGD Participant 03 and FGD Participant 04 are all here, so everyone has entered.

Moderator: For attendance and practice, please use the existing sticky notes. Copy one from the left using Control-C, paste it using Control-V, and replace the text with your own name. You can use the yellow note below to practice typing and moving it.

Moderator: For attendance, use the note beside my name. Click mine, press Control-C, click anywhere, and press Control-V. FGD Participant 05 and FGD Participant 03 have already done it.

Moderator: "This is fun; we can see them moving." You can click mine, or, as FGD Participant 07 has done, copy it, double-click in the middle, and replace the name.

(...)

Moderator: Excellent, FGD Participant 03. Welcome to the group, [FGD participant].

Moderator: FGD Participant 07 FGD Participant 02 FGD Participant 05 FGD Participant 04 and FGD Participant 01.

Moderator: Correct, correct.

FGD Participant 03: Because (???) is not included in the lunch arrangement later.

Moderator: (laughing)

Moderator: This also serves as attendance.

(...)

Moderator: Where is FGD Participant 01's name?

FGD Participant 01: Which one?

Moderator: Click it, [FGD participant].

Moderator: Click this one. For example, FGD Participant 05 can press Control-C and paste it here, then click and replace the name. Who owns this green one? I am sharing my screen below. Can you see it? This is only practice so everyone knows how it works. FGD Participant 04 has already dragged this one here.

Moderator: FGD Participant 01 write your name below.

FGD Participant 01: Why will it not move down?

Moderator: Use the drag function.

FGD Participant 01: Done, done.

FGD Participant 01: So I copy the one from the box?

Moderator: You can also move it, or copy any one of them.

FGD Participant 01: It can be another person's name; that is all right.

Moderator: Yes. FGD Participant 05 can do the same. Okay.

Moderator: Everyone has now recorded attendance. The area below is actually...

Moderator: ...only for practice, so the exercise is easier. We will work above later. To change the size, double-click and drag it larger, like this. To create an arrow, draw it from here toward the other item. You can also move or modify the green note.

Moderator: To zoom, use a function similar to a Zoom meeting. For example, double-click the green note and drag it to the left. Then copy and paste it. To write, double-click and type directly inside. Let us try it first.

Moderator: If anyone has questions, we can address them as we proceed.

Moderator: The method is outcome harvesting. As program implementers, we will explore what you believe the CCRIW program has achieved. The results will provide a basis for my field verification, where I will examine whether the outcomes you expect, predict, or perceive are supported by evidence.

Moderator: We will not strictly use a theory of change or logical framework because this program did not have a logical framework at the beginning. Outcome harvesting is one method we can use for such an evaluation. We will begin with the results and then proceed through the discussion. Everyone may write as many outcomes as possible, and we will group them afterward.

Moderator: We will identify the main outcome themes. Please write directly in the outcome area inside the box. I will help group them, and we will discuss the groupings at the end. Do not draw arrows yet. For example, if I have an idea about an outcome in this section, I write my name and click here. You may write freely.

Moderator: Anyone may begin writing. The first question is...

Moderator: In your view, what has changed as a result of implementing the CCRIW program, whether in the community, among village heads, or...

Moderator: ...within Community Based Total Sanitationeams? Who changed, and what change occurred?

Moderator: Please write your answers here.

(...)

Moderator: Thank you, FGD Participant 02. FGD Participant 01 has also begun. To protect the integrity of the process, may I ask FGD Participant 09 not to participate, so everyone can express opinions freely without being monitored by the project manager? May FGD Participant 09 leave? Okay, thank you, Project Manager.

Moderator: The project manager has left, so please write your responses directly.

Moderator: If you need help, write in the chat. FGD Participant 06 and I can assist. If it is difficult to write directly in Miro, place your response in the chat, and we will transfer it.

Moderator: FGD Participant 06 can you also enter Miro?

Moderator: Okay. There are many purple notes above. We do not need different colors. Take a purple note from above and enlarge it slightly.

Moderator: FGD Participant 06 can you enter Miro?

FGD Participant 06Study area : Yes, I can.

Moderator: Thank you. You may type outside the box first and drag the note into it later. Remember to write your name. Use a purple note. If it is too large, make the letters smaller by double-clicking and changing the size here, so we have enough space.

Moderator: We will allow about five minutes for everyone to write. If you need help, place the text in the chat, and I will transfer it to Miro.

Moderator: FGD Participant 10 has just joined. Welcome. Good morning in Studiy area .

FGD Participant 10: Good morning. Sorry I joined late.

Moderator: That is all right. We have already begun. FGD Participant 10 please join Miro. I have shared the link again in the chat.

FGD Participant 10: It is clear.

Moderator: I will explain once you have entered. We are in the first section, discussing what outcomes the CCRIW program has produced.

Moderator: Sorry, FGD Participant 06. Please only monitor and do not type. FGD Participant 10 has entered and can do what FGD Participant 01 has already done.

Moderator: We will allow another three or four minutes for everyone to write about CCRIW activities before we discuss them. FGD Participant 10 double-click here and press Enter. If you need help, type your response in the chat, and FGD Participant 06 or I will enter it for you.

Moderator: FGD Participant 02 please make yours smaller.

Moderator: Who wrote this item? Please add a name. I will drag it upward.

Moderator: FGD Participant 07 please add your name. FGD Participant 04 used a blue note. Please do not use blue; use purple and write here.

Moderator: For example, this one.

FGD Participant 02: [FGD participant], where do I reduce the font size?

Moderator: Double-click here, and then use the font-size control in this section.

Moderator: Press Control-A while editing, and then you can change it. Once you finish editing, the data should be visible.

Moderator: Done. Okay, excellent.

(...)

FGD Participant 02: Okay, excellent.

FGD Participant 02: [FGD participant], may I enlarge this? I want to add an outcome for schools.

Moderator: Let us focus only on the community and village levels. Schools and health centers are outside the present scope.

FGD Participant 02: Okay.

Moderator: They are outside the scope.

FGD Participant 02: Okay, understood. Then I am finished.

Moderator: FGD Participant 10 please also focus only on this area.

Moderator: (???) and facilities at the village level.

FGD Participant 02: Understood.

Moderator: Or actors at the village level.

Moderator: Most people have finished. Does anyone still want to add something? Remember to write your name. FGD Participant 10 please add your name so we know who expressed each opinion.

Moderator: FGD Participant 04 are you finished?

FGD Participant 10: Should I put my name in parentheses, or how?

Moderator: Follow the format used by FGD Participant 07.

Moderator: Write \"FGD Participant 10:\" and then your answer, so your name appears at the beginning.

FGD Participant 10: Okay. (NOISE)

Moderator: I moved this one. Who wrote \"OKY\"? FGD Participant 03?

FGD Participant 03: That was FGD Participant 03, and [FGD participant].

Moderator: Oh, understood. That is why the page suddenly became so large. Why do FGD Participant 10's and [FGD participant]'s notes look like this?

Moderator: Okay, has everyone finished?

Moderator: We have not yet reached the green section concerning evidence.

Moderator: FGD Participant 10 please wait before entering evidence and reasons. For now, focus on outcomes and use purple. Everyone should remain focused on the upper section.

Moderator: Do not use the lower section yet. We will use it later when finalizing the results. Has everyone finished? In the outcomes column, write only outcomes, meaning changes that occurred as a result of CCRIW activities.

FGD Participant 10: So the focus is only the village, excluding schools and health centers?

Moderator: Yes.

FGD Participant 10: Okay.

Moderator: Change the color. Apparently, we can also add emojis.

Moderator: We will address reasons and other information later.

Moderator: FGD Participant 03 are you finished?

FGD Participant 03: Yes. Okay, I am arranging it so that it can (???).

Moderator: Everyone has finished. We will now discuss how to group the outcomes. For the initial grouping, I will use this color.

Moderator: We can create one grouping for community-level outcomes and another for village-government outcomes. Beginning with FGD Participant 10's item...

Moderator: Village budgets in 32 villages and urban village have produced...

Moderator: ...or covered CCRIW outcomes. I interpret this as village funds accommodating CCRIW action plans. Is that correct, FGD Participant 10?

FGD Participant 10: Yes, correct, [FGD participant].

Moderator: The action plan. I will bold the items already placed in this group. FGD Participant 02's item also concerns village and urban village governments and village funds, correct? It includes construction of ramps for persons with disabilities and replanting.

Moderator: I will retain FGD Participant 02's example, but because its theme is the same as FGD Participant 10's, I will group them together. We can discuss changes to the grouping later.

Moderator: At the district level, there was collaboration and required cost-sharing for reforestation. FGD Participant 02 could you explain that briefly?

Moderator: Specifically, the district-level cost-sharing.

FGD Participant 02: Certainly. This was also an outcome of work by [FGD participant] and colleagues. CCRIW encouraged activities through the Water Resource Management Forum forum. Many activities were conducted, although the funds did not come from Plan. Financial support came from several regional government offices, while seedlings were obtained free of charge from the relevant production and forest-management units. They were planted at several locations. For example, on Environment Day, trees were planted around a small reservoir.

Moderator: Thank you. So there was also a district-level outcome. FGD Participant 10 was there something similar in Study Area?

FGD Participant 10: Yes, [FGD participant]. The Water Resource Management Forum forum is quite active in conservation. Through the private company, it regularly conducts mangrove planting. Another program initiated by the Water resource Management Forum forum with a university involved planting mangroves as part of the CCRIW action plan in coral village . Approximately 1,000 seedlings were planted there. The water resource management forum forum also signed a memorandum of understanding with the provincial watershed-management agency to provide 10,000 seedlings per year. About 1,500 seedlings were planted in uppper village g as part of the action plan. Thus, village CCRIW action plans were integrated with the water resource management forum's work program. The annual 10,000-seedling activity will continue. Activities have already been conducted with privat comapny , with persons with disabilities inupper village , and with university students in coral village .

(...)

(NOISE)

Moderator: FGD Participant 07's item is similar to FGD Participant 10's. It says that subdistrict Community Based Total Sanitation teams better understand WASH and climate-change issues. FGD Participant 01 also proposed a government-related outcome...

Moderator: ...increased knowledge among village CCRIW/STBMCommunity Based Total Sanitation and subdistrict teams. Beyond village Community Based Total Sanitationteams, has there also been a change among village heads?

FGD Participant 01: God willing, yes, [FGD participant]. The change is visible in budgeting. Village governments are beginning to integrate CCRIW action-plan issues into their budgets.

FGD Participant 01: So both Community Based Total Sanitation teams and village heads have changed in relation to CCRIW.

Moderator: Okay, those are government-side changes. Now let us consider community-side changes.

Moderator: There are still many items that have not been...

Moderator: Keep them here under the community category.

FGD Participant 10: Perhaps increased knowledge among village governments, Community Based Total Sanitationteams, and communities could be combined because the CCRIW process involved many community members, including persons with disabilities. Monitoring results may help justify an outcome of increased community knowledge as well.

Moderator: Thank you. So we have increased knowledge through CCRIW. FGD Participant 07 wrote that communities recognize resources and potential that support climate-change adaptation. We could phrase this as increased community understanding of resources and potential.

(...)

Moderator: FGD Participant 06 please help take notes on this discussion. I will write the short points here, and you can prepare the narrative. The outcome is that communities understand resources and potential for climate adaptation and GEDSI-responsiveCommunity Based Total Sanitation. Is that correct, FGD Participant 07?

Moderator: FGD Participant 04 wrote about increased youth involvement and community contributions from Community Based Total Sanitation teams to modify ramps and toilet handrails. This represents more inclusive community participation in CCRIW-related activities. Community Based Total Sanitation teams also contributed resources to modify ramps and toilet handrails. FGD Participant 04 could you explain that briefly?

FGD Participant 04: Certainly.

FGD Participant 04: regarding modifications to toilet handrails and

ramps, after the triggering activity, um, I tried to follow up again regarding [???] with the urban village office government. At that time I met with the head of urban village . The head of urban village said there were no funds left. At that time, through discussions with the urban village office , [???] several members of the Community Based Total Sanitationteam [???]

FGD Participant 04: from the STBM colleagues, members of the STBM team who were able and willing made toilet handrails and ramps. This happened in only a few urban village office .

Moderator: Thank you. Was this in the studi area, FGD Participant 04?

FGD Participant 04: Yes.

Moderator: In other areas,

Moderator: were there also volunteers from the Community Based Total Sanitation teams who contributed independently?

[…]

Moderator: Hello, hello.

Moderator: Apart from studi area , were there other areas where Community Based Total Sanitation team members volunteered? If not, I will not write this as something equivalent across all areas. It will be recorded as a special note for [Study Area 3]. Was there anything elsewhere? Was this common, or only… go ahead… go ahead…

FGD Participant 03: In studi area , the village Community Based Total Sanitation team members also, what is the term, informed village colleagues about locations for toilets that were safe and easy to use. Then the village head also, um, allocated the village funds. There were also Village Fund Allocations, or ADD, that could be used to renovate toilets.

Moderator: Thank you. So this was advocacy.

FGD Participant 03: Yes, advocacy, but they did not build the facilities themselves as in study area ?

In study area , the village constructed the toilets itself using its ADD, and at the same time procured water storage tanks.

Moderator: Okay, okay… Now, the CCRIW results… okay, this one first.

Moderator: We have covered everything except these two. “Communities better understand the importance of maintaining cleanliness, particularly in relation to the five Community Based Total Sanitationpillars.” Could you briefly explain how CCRIW influenced this, FGD Participant 01?

Did this understanding already exist before CCRIW, or did CCRIW improve community understanding? Could you explain this part briefly?

[…]

Moderator: Hello, FGD Participant 01… this part.

FGD Participant 01: Hello, hello, [FGD participant].

Moderator: Did CCRIW improveCommunity Based Total Sanitationbehavior, or did Community Based Total Sanitation instead encourage CCRIW-related behavior?

FGD Participant 01: Um… yes… because during CCRIW, there was also, what is it called, education included. For example, during CCRIW in the village, there was education related to the five pillars, in addition to education for the government. I think the community also received that education.

Moderator: So it reinforced the understanding.

FGD Participant 01: Yes. For example, waste disposal is also included in the five Community Based Total Sanitationpillars and in climate-change issues. There were also community action plans, not only government action plans.

Moderator: Mm-hmm.

FGD Participant 01: Those emerged at the end of CCRIW.

Moderator: All right, thank you. Finally, FGD Participant 10 wrote, “CCRIW results were aligned with Regional Medium-Term Development Plan targets.” [???] Did the Regional Medium-Term Development Plan change because of CCRIW results? Is that what you meant, FGD Participant 10?

FGD Participant 10: All right, [FGD participant]. Regarding why I discussed integration and said that they were aligned, it was because…

Moderator: I am sharing my screen, FGD Participant 10. We are in the results section. The question is this: You wrote that CCRIW results were aligned with Regional Medium-Term Development Plan targets at the district level. Was this a reason, or did you actually observe that the Regional Medium-Term Development Plan accommodated CCRIW results?

FGD Participant 10: Regarding the RPJMDRegional Medium-Term Development Plan, it was already being implemented during the period that began before the first phase of WfW ended, [FGD participant]. So at that time, um, the process… In the Regional Medium-Term Development Plandocument that had already been prepared, the fifth mission was “Resilient and Sustainable [Study Area 2].”

It was still closely related to climate-change issues. This was one reason why, after the CCRIW results were completed in 32 villages, the advocacy process at the district level proceeded relatively easily. The Regional Medium-Term Development Plan document was aligned with the program. One mission addressed climate change, while Mission One, “Healthy for All,” includedRegional Medium-Term Development Plan, so the two were combined across the five missions. In the governor's regulation on Regional Revenue and Expenditure Budget preparation, this was also identified as one of the priorities for village budgets. Therefore, when we conducted advocacy concerning the CCRIW results, we compiled all of them and discussed them at the district level. We invited all villages, including the subdistrict heads, and asked the head of the Village Community Empowerment Office to explain why it was important to integrate CCRIW issues into village budgets. This occurred before discussions of the 2024 APBDRegional Revenue and Expenditure Budget, so it was last year. I used the term “aligned” because this facilitated the advocacy process, including the earlier point that CCRIW was accommodated through village budgets. At the district policy level, both the Regional Medium-Term Development Plan document and the governor's regulation providing village-budget guidance already mandated that this issue be prioritized. That is why I said it was aligned. The [Study Area 2] Regional Medium-Term Development Plan had existed before the CCRIW process began. So the appropriate language is that the two were aligned. That was the justification, [FGD participant].

Moderator: All right. Thank you, FGD Participant 10. So this was actually the reason the village budgets accommodated CCRIW results, because of this alignment.

FGD Participant 10: Yes. Because it was a priority in the Regional Medium-Term Development Plannd in the governor's regulation guiding village-budget preparation, which is essentially the village heads' “bible” for preparing their budgets, it became one of the priorities. Therefore, the multilayered advocacy process last year was greatly assisted by the higher-level policies.

Moderator: Okay, thank you. May I move this to the “reasons” section because it is a cause? This is the cause that leads here. Okay, we may discuss it in greater detail. FGD Participant 06 please remember to record FGD Participant 10's explanation about the advocacy process. All right, FGD Participant 06?

FGD Participant 06 study area : All right, [FGD participant].

Moderator: Thank you. FGD Participant 06 please record in detail the explanations provided by the other participants.

Oh yes, we have already covered the integration of the CCRIW plan. This… it belongs here. The water resource management forum item often held back activities by the district water resource management forum office. Please look at the shared screen. Have we covered all the items in this color? Is anything still missing?

If nothing is missing, I will copy the pink items.

FGD Participant 10: Perhaps one result was improved infrastructure, [FGD participant]. Okay.

For example, during the current program period, action plans have already been implemented through village budgets. Several were implemented, including repairs to network pipes, while others involved, perhaps, village heads and the distribution of seedlings funded by village budgets, among other activities. Therefore, improved infrastructure or similar changes could also be considered a result at the community level.

Moderator: At the household level or the village level?

FGD Participant 10: There were two types last year, [FGD participant]. Some were at the village public-facility level, such as general repairs to clean-water facilities. In upper village Village, however, a toilet specifically for people with disabilities was constructed. For example, that was at the household level. So, there were two types of upgrades, at both the village and household levels.

Moderator: Mm-hmm… In study area have there been changes at the household level? Were they only for people with disabilities, or were they for the general population?

FGD Participant 03 earlier mentioned the purchase of storage tanks. Were those household water tanks, FGD Participant 03?

FGD Participant 03: Yes, yes. Not only water tanks. In several villages, for example, community members began constructing water-storage basins inside their toilets and bathrooms. These had never been constructed before.

Moderator: Thank you very much.

FGD Participant 02: May I add something about study area ? They only recently included the purchase in the 2025 plan and budget. As you know, kelurahan do not have specific dedicated budgets, but they entered the purchase directly into their budget utilization listfor water-storage tanks and the installation of rain gutters. At the community level, people focused more on cleaning their dug-well reservoirs.

Moderator: FGD Participant 06 please help me write FGD Participant 02's response here as a special note for [Study Area 3]. FGD Participant 06…

FGD Participant 02: Because the approach in study area was different. In [Study Area 3] itself, there was already a target for CCRIW.

FGD Participant 06 study area : Just a moment.

FGD Participant 02: We only conducted training for witnesses in study area City last year, but over time, after we met, it was eventually added.

FGD Participant 02: study area was added to increase our progress figures.

Moderator: Was there no baseline in study area previously, or was there one?

FGD Participant 02: The baseline was in study area .

Moderator: Which…

FGD Participant 02: I may be forgetting, [participant]. However, during the baseline, they examined matters such as water quality. Initially, [Study Area 3] was targeted only for training. Over time, however, we also monitored CCRIW. We began mobilizing follow-up activities from the practice conducted in study area City. At that time, [FGD participant] and FGD Participant 03 and the others helped. Afterward, we stopped because we felt this was not included in the target, so several community self-help contributions were used for the construction. For example, handrails and ramps in several urban village were financed using their own funds. Whether the funds came from community self-help contributions or colleagues concerned about waste, they combined their resources for those activities.

Moderator: Thank you very much, FGD Participant 02.

FGD Participant 06 study area : Yes, yes.

Moderator: We can write it here.

FGD Participant 06 study area : I was focusing on the laptop earlier.

Moderator: All right.

FGD Participant 06 study area : FGD Participant 02's description is already here.

Moderator: So that it does not later enter the data…

Later, when we ask for help [???]. Okay, so we have completed all of this.

When everything is complete, I will move it downward, and this will remain here as the main item. Now, this is Stage One. Stage Two… In Stage Two, please move to the “reasons” column. Earlier, we grouped the items into increased knowledge,urban village funding that accommodated CCRIW action plans, and so forth. FGD Participant 10 has already explained some of the reasons, and others have also written some. Please add the reasons, specify where they apply, and draw arrows. For example, “CCRIW was aligned with the RPJMD, resulting in shared funding,” only as an example, and write it here. Whoever draws an arrow, for example FGD Participant 10 should write their name beside it. If the letters are too large, make them smaller. Please look at my shared screen. Okay, like this. I will delete this example. Please write the reasons and draw arrows. Does anyone need further explanation? I will give you ten minutes to work on the reasons.

FGD Participant 01: These are the reasons for the results we discussed earlier, correct?

Moderator: Correct. This allows us to see the reason for each argument. I will arrange it like this to make it easier to draw the arrows. We are summarizing the items, so it is not… this…

This can also be enlarged. Write first, and then we can adjust it. This is the arrow… Please proceed.

I will check again at 10:30. Thank you.

[…]

It is enjoyable to watch people's items moving, especially those with photographs, such as FGD Participant 03 FGD Participant 10 and FGD Participant 01. Their pictures are moving around.

[…]

Remember to double-click each arrow and write your name on it. I am showing an example on the shared screen. When we create an arrow, double-click in the middle and write your name so we know who proposed each connection. Please double-click the arrow and enter your name.

[…]

Excellent, many arrows have already been created. Remember to write your names. Great… great.

This one. Is there a name…

[.]

The shape is okay. For this one, change the line type so it is easier to see. I am showing on my shared screen how to change it. Do not use the elbow-shaped type.

[…]

Do not worry if one result is related to another. For example, if village funding leads to improved infrastructure, you can also draw an arrow. If village or urban village funds accommodate CCRIW and you want to connect that to the red item, “improved village infrastructure,” please do so. There can be causal relationships among results at different stages. I will give you five more minutes, until 10:45, and then we can continue discussing the grouping. This means we have been working from around nine, ten, eleven… almost one hour and forty-five minutes. Okay.

Moderator: This is done. Wow… the arrows are very complicated.

[…]

Moderator: Okay, we will begin. I will make a backup below first. Stage Three is clear. Is there anything else, everyone, or may we continue to the next stage?

Hello, hello…

Approximately how many more minutes do you need?

Okay, I can copy this below. I will no longer… Even if something is added here, it will not be included again in… I will title it “hold outside.”

Okay, we will begin with the results. Among these results, are any connected to each other through cause and effect?

Okay, hello… or we can begin with the existing connections. “The availability of CCRIW guidance tools added to the body of knowledge.” Okay, I will start by focusing on increased CCRIW knowledge. I will begin with FGD Participant 10's item and copy it to the right. Please look at the shared screen. I am moving it to the right to make it easier. I will bold this one. Next, whose item is this? “The Integral Ecology Year of [study city/institution].” Okay, I also happen to know about this. Could the team briefly explain the Integral Ecology Year of [study city/institution]?

FGD Participant 03: The chair of the KBG. Hello… In [study city/institution], there is a theme each year based on the bishop's pastoral letter. This year, the selected theme is integral ecology, which concerns the environment. It includes life and human beings, and everyone is expected to participate. The issues raised during this Integral Ecology Year focus on how people can care for the Earth as our common home. Examples include water issues and efforts by members of the diocese to protect water sources, as well as conservation efforts by the congregation. From the results of

the Integral Ecology Year, the strength lies in basic faith communities or categorical groups at the smallest local level. [???] These are called Basic Ecclesial Communities. Each consists of approximately 30 households. They conduct activities called socialization, or catechesis in church terminology. The sessions explain what members of the congregation can do to protect the environment.

The socialization is followed by activities. These include making waste bins in each KBG and efforts to protect water sources, such as planting trees. There is also cooperation with colleagues from the Natural Resources Conservation Agency,, and the Forest Management Unit,, which provide seedlings. After Mass at each church, members of the congregation can receive the seedlings free of charge. Although the seedlings are free, the church urges recipients to plant them in their yards or gardens. That is how it works.

Moderator: All right, thank you very much. Did Plan colleagues make any effort to integrate an external factor such as this into CCRIW, or did it occur organically?

FGD Participant 03: I think field activities inevitably involved others. Colleagues from church groups were involved only in several of our activities. Field colleagues informally communicated, for example, with parish priests. One example was communication with colleagues at [local parish] to conduct a joint tree-planting activity. Church colleagues and relegion organisation , or Catholic youth, at the church participated and supported the tree planting. The activity was also a collaboration among Plan, the Integrated Resource Empowerment forum in [study district], and church groups at [local parish]. However, CCRIW activities conducted specifically in villages did not involve church groups. They focused more on older adults, Plan staff, and facilitators. There was a special case at [local parish] in [local subdistrict], where a sanitarian served as a CCRIW facilitator and was also an OMK leader. Therefore,

the information he received during CCRIW was also conveyed to his OMK colleagues. In [local subdistrict], the participating villages were within the parish service area, and most participants were Catholic youth. Therefore, a connection or integration with church groups could be identified. They were also encouraged to participate in efforts related to [???] integral ecology, an issue promoted by [study city/institution].

Moderator: Thank you. So the connection existed because there was a facilitator and also through water resource management forum , correct? Was the reforestation actually a water resource management forum activity rather than a CCRIW activity?

FGD Participant 03: The initial idea came from the water resource management forum forum. We wanted to communicate it to the regegion organisation colleagues because, when they participated in CCRIW, they had already received information that was later reinforced by our planned reforestation activity. Although the activity was initially initiated by water resouce management forum , participants had already received information from the CCRIW team, which made them more willing to join. The tree-planting site was in an green open space area. In forest management unit terminology, it was one of their supported areas. It was a forestry area that the general public could not enter freely, so participation had to occur with water resource management forum or forest management unit colleagues. They provided the activity site, while participants came from the general public.

Yes, that is the story.

Moderator: Thank you very much. There are already many stories that can be directly verified. Later, we may select which evidence we agree to include in our report. There is already a great deal of evidence. From [FGD participant], I forgot to mark the CCRIW resource-mobilization action plan. I will combine it with FGD Participant 10's answer about the existence of guidance for resource-sharing and exploration.

Now, the action plan and resource mobilization, and the item from [FGD participant] earlier, where is it? This first section contains only one or two items. I will group them. We will move to village funding. “The CCRIW action plan was integrated into Regional Revenue and Expenditure Budget and Village Revenue and Expenditure Budgetthrough advocacy

conducted up to the district level.” Was this advocacy conducted by Plan staff or by the Community Based Total Sanitation team?

[NOISE]

FGD Participant 10: It was initiated by Plan, [FGD participant]. This is what I explained earlier. After compiling the action plans, we discussed them with the Village Community Empowerment Office,. We then gathered all village heads as the primary targets. We also gathered subdistrict heads in their capacity as chairs of village-fund verification teams, and we asked the head of the Village Community Empowerment Office office to serve as a resource person. The purpose was to ensure that climate-change issues, including CCRIW action plans, were accommodated in Village Revenue and Expenditure Budgetby explaining the regulations and policies at the district level. At the time, we also asked the official to compile the 2023 village budgets that had accommodated sanitation and climate-change issues and present the total amount. If I remember correctly, it was about three billion rupiah in 2023. The official spoke directly to the village heads to ensure that CCRIW results were genuinely accommodated in Village Revenue and Expenditure Budget.

Moderator: Okay. FGD Participant 10 this was advocacy by Plan. I have deleted the duplicates. There is still a backup above, correct? May I delete it so the board is not cluttered? This goes here. Second, what is this? Okay, “There is a connection between the SDGs and CCRIW issues contained in the Regional Long-Term Development Plan.” Oh, it is the Regional Long-Term Development Plan rather than the Regional Medium-Term Development Plan, meaning the long-term plan. FGD Participant 10 also said it was included in the Regional Long-Term Development Plan.

FGD Participant 10: That may refer to theRegional Long-Term Development Plan, the 20-year long-term plan. In Studi Area , environmental issues are also among the priorities in the Regional Long-Term Development Plan.

Moderator: There is an issue. Okay, this is regulatory support. There is a regent's regulation concerning village funds, correct? Does the regent's regulation for village funds address climate change?

FGD Participant 10: It is called the Regent's Regulation on Guidelines for Preparing village budget , and it is issued every year.

Moderator: Thank you. In study area , is climate change also included in a regent's regulation concerning village funds, [FGD participant]?

FGD Participant 02: Hello. There is not yet a written regent's regulation like that. However, theRegional Long-Term Development Planalready includes inclusivity, Community Based Total Sanitation , and environmental issues. Study area does not have a regent's regulation on village funds comparable to the one in Study area a.

Moderator: In Study area , was there actually an activity comparable to what FGD Participant 10 described [???]

FGD Participant 02: There was no activity at all in which, because a forum still existed for CCRIW, we held a special meeting for that purpose.

Moderator: So it occurred only in study area . We can examine the effects. FGD Participant 10 could you later share this regulatory document?

FGD Participant 10: One of the documents I sent earlier was the regent's regulation on village budget preparation guidelines, [FGD participant].

Moderator: Okay… This is another item from FGD Participant 10: “Involving the appropriate stakeholders and using the application were important factors.” The appropriate stakeholders were the village and subdistrict heads involved in the review, correct, FGD Participant 10?

FGD Participant 10: At the time, we considered the timing. Discussions and preparation of village budget would occur, for example, in December or January. Therefore, we selected a time before the village budget discussions or finalization had been completed. We gathered village heads as the primary targets because they needed to include the items. Subdistrict heads also had to be gathered because they chair the village-fund verification teams. The village empowerment office office also had to be a primary stakeholder and communicate the message. Stakeholder mapping was therefore one of the keys to the advocacy process we conducted. In addition, timing was important. Even when everything else is ready, the opportunity can be lost if the moment has passed. Timing is therefore very important.

Moderator: All right, thank you. I have combined all of these into one item. Okay, next. These links are very close together. We…

for participants two and three…

Okay, this one is complete.

The item in the RPJPD earlier.

Moderator: Okay. Who wrote the next one?

FGD Participant 01 please write your name here.

What is written above? “CCRIW action plans supported the urgency of WASH and climate-change issues because action plans existed, were compiled, and were used for advocacy.”

Whose item is this, FGD Participant 07? “The subdistrict head and Community Based Total Sanitation team monitored the action plan.” Could you briefly explain the monitoring of CCRIW actions, FGD Participant 07? What did the monitoring activity involve?

FGD Participant 07: Um, this was still related to the action plans developed during CCRIW. The subdistrict water and community sanitation team happened to serve as facilitators at that time. The subdistrict head followed up on the action plans to ensure that they were included in village budget. That was the monitoring approach. It checked whether the commitments made during the CCRIW action-planning process were actually included in village budget .

Moderator: Okay, good. So the monitoring concerned village budget , not monitoring [???]

We already have this item here.

FGD Participant 07: It also concerned households, [FGD participant], including whether there had been changes within households after the CCRIW activity. The monitoring was conducted before the meeting last year [???], or?

This item is indeed connected as well.

Moderator: So the monitoring was intended to examine… okay. Was it conducted immediately after the CCRIW activity, or only recently?

FGD Participant 07: It was conducted recently. A budget happened to be available, and the subdistrict routinely conducts monitoring. Last year, however, the team went directly to households. At that time, 30 households were monitored in each village.

Moderator: Together with Plan?

FGD Participant 10: Perhaps I can add something, [FGD participant]. Following the advocacy process at the district level, villages committed to accommodating CCRIW plans. The APBDes approval process is multilayered, beginning with village submission, verification at the subdistrict level, and then review at the district level. The subdistrict-head monitoring mentioned by FGD Participant 07 refers to the subdistrict head's role as chair of the village-fund verification team. The monitoring ensured that commitments made at the district level continued to be safeguarded through the APBDes approval process and did not end with the district-level meeting. Second, CCRIW and Community Based Total Sanitation monitoring was conducted several months ago to determine whether, first, CCRIW plans remained active and, second, the five Community Based Total Sanitation pillars were still being practiced. Monitoring also covered locations affected by floods or droughts to determine whether behaviors were maintained and whether constructed infrastructure had been preserved. It also checked whether additional infrastructure had been built to accommodate the CCRIW results. The Health Office conducted this work.

Moderator: Why was monitoring conducted only after one year?

FGD Participant 10: Regarding that…

Moderator: Was the purpose to observe results or to bring about change?

FGD Participant 10: Both, [FGD participant]. We used two tools. One tool monitored the five pillars, while the other examined additions to infrastructure. The two tools were used simultaneously during monitoring.

Moderator: So the purpose was to observe changes rather than to, um, remind people again. In the Community Based Total Sanitation triggering sequence, there is triggering followed by action to follow up on the triggering and the social contract. Did CCRIW not have a similar follow-up activity?

FGD Participant 10: The CCRIW process was conducted in 2023.

Moderator: Mm-hmm, mm-hmm.

FGD Participant 10: So indeed… yes, what were you asking, [FGD participant]?

Moderator: There was no immediate follow-up activity afterward to examine the commitments, correct?

FGD Participant 10: That follow-up was the safeguarding process. After the CCRIW action plans were prepared, we compiled them and processed their integration into village budget through the district-level meeting I described earlier.

Moderator: So the direct follow-up was conducted with the village government, not with the community?

FGD Participant 10: Yes, correct.

Moderator: [???] The Community Based Total Sanitation practice we conducted was different from that of people [???]

FGD Participant 10: Correct. The monitoring concerned the commitment to ensure that the plan was accommodated in village budget , and that process continued. The monitoring conducted last year, however, concerned implementation.

Moderator: Thank you. We will move to…

FGD Participant 02: [FGD participant]…

Moderator: Please go ahead.

FGD Participant 02: Excuse me. Our study area group was disconnected.

Moderator: All right.

Moderator: FGD Participant 03 is still joining… one of the others is here. Oh, I will contact the study area participants myself later. Has FGD Participant 03 joined? All right. FGD Participant 03's team has… oh, they still cannot speak. We will wait. [FGD participant] informant … Hello… hello… adjust it… Okay, has FGD Participant 03 joined?

FGD Participant 03: Yes, yes, I am ready.

Moderator: All right. We will continue.

(Communication in English) Um… okay, so… (unclear/self-dialogue)… Hello…

FGD Participant: Yes, is that right?

Moderator: Can you hear [FGD participant] infromant chatting?… (unclear)

Okay, now FGD Participant 07. “Village or urban village CCRIW action plans were produced.” So that is complete, correct? It was already captured in the statement about CCRIW action plans. Mm-hmm. Could someone explain why the advocacy activity occurred only in study area and why the approach in study area was different? Was this due to the project design, or something else? Why was there no comparable advocacy activity in [Study Area 1]? FGD Participant 03? [FGD participant]?

FGD Participant 03: Hello.

Moderator: Hello, yes.

FGD Participant 03: Were you asking us a question?

Moderator: Yes. Why was there no… I am not judging anyone. I want to explore the difference so I can conduct a comparative analysis across districts. Why was there no village-fund advocacy for CCRIW in [Study Area 1]?

FGD Participant 03: Hello. All right. In [study district], advocacy activities continued, but the form was different. In [study district], the PJMT team and members of the water resource management forum forum visited the 12 pilot subdistricts to advocate for village funds to support CCRIW implementation. This activity occurred after we completed the CCRIW assessment at the village level. It was intended to reinforce our advocacy. During the subdistrict-level village-fund advocacy activities, resource persons came from the [???] district. They included colleagues from regional planning agencyt , village empowermnet office representatives, the district family walfare and empowerment , and members of the water resource management forum forum.

Participants in the village-fund advocacy activities included village heads and village operators from all of our pilot villages. Village facilitators also participated, as did members of the subdistrict Community Based Total Sanitation eams who focused on disability issues. Therefore, it would be incorrect to say there was no advocacy. Advocacy was conducted, but it differed from the approach used by colleagues in study area . We did not… (unclear) all pilot villages and their village heads in one place. Instead, we visited each subdistrict. At those meetings, we presented the follow-up plans resulting from CCRIW so that the subdistrict heads and village facilitators would understand the plans and help ensure that the issues raised through CCRIW were supported in the village follow-up plans. That was the approach.

Moderator: All right, thank you very much. So both locations conducted advocacy, but the format differed. The other difference was that study area had a regent's regulation concerning village funds and climate change, whereas study area did not. Is that correct?

FGD Participant 03: Regarding the specific regulation in Study area , it sounds as though there was an activity to support a regent's regulation through a meeting. We did not have a special activity like that in study area i. However, at the regulatory level, our long term development plan does exist. The Regional Long-Term Development Plan includes inclusivity, climate change, and environmental issues.

Moderator: All right. We will move the item on funding shared by government offices. Thank you very much, [FGD participant]. We will move to the next item: activity funding shared by district-level offices and TSK. The first item, guidance tools, has already been placed there. Okay. So these are actually similar. I will arrange them like this. First, from FGD Participant 02 there are long term developmnet planning and sanitation strategy regulations that cover GEDSI and climate resilience. Does study area not include climate-change issues?

FGD Participant 10: It does, [FGD participant], but the process is still ongoing. [district sanitation strategy document] covers the 2022–2026 period, so the process is still underway. The sanitation strategyhad not yet been signed. There was a national policy requiring it to become a regent's regulation and to include climate change, stunting, and disaster issues. This is still being processed. We discussed it with [a government official] and the consultant. We did not need to include the issues in the EHRA study document because the current document had already been completed. We only needed to collect secondary data to incorporate the three issues: stunting, climate change, and disasters. That is in process. The regent's regulation is also why I was late this morning. We discussed the draft regulation with the working group and submitted it to the legal division for further review. The process is therefore still ongoing.

Moderator: Thank you. Second, regarding water resource managemnet forum , how was water resource management forum integrated with CCRIW? What was the answer for [Study Area 1]? Oh, [FGD participant] discussed that earlier… First…

FGD Participant 03: Hello, hello. Yes. In study area , during village-fund advocacy at the subdistrict level, we involved colleagues from the water resource management forum forum. When village follow-up plans included reforestation or water-resource conservation activities, members of the water resource management forum forum could provide solutions during the subdistrict-level advocacy activities. For example, if villages had difficulty obtaining information or support for tree seedlings, the forum could assist. Second, the forum could provide education about how to… (unclear), and so forth. That is the form of collaboration we used in study area . We described the activities as integrated in that way. Colleagues from regional agency palnning also assisted, not by financing villages, but by communicating these issues to regional government agencies so that conservation and other environmental activities could be included in the dikstict budgeting of the relevant regional agencies. During village-fund advocacy, these colleagues were also members of the water resource management forum, so issues discussed in the forum were passed on to villages. This made the process easier. Many colleagues assisted with the advocacy.

Moderator: Mm-hmm, okay. Thank you, [FGD participant]. FGD Participant 10 how was natural-resource management integrated with CCRIW in area study ?

FGD Participant 10: You mean study area ? Hello. In study location , we consistently involved the water resource management forum forum, the disability forum, traditional institutions, and related stakeholders in various meetings. We did not omit them from activities such as WASH FIT or the village budget advocacy activity discussed earlier. We always invited their representatives to participate. When we discussed the work program of the water resource management forum forum, which was also initiated by Plan, water resource management forumcolleagues understood the needs and outputs identified through CCRIW. Although they could not accommodate every action plan, several work programs could be aligned with CCRIW plans. One example was the need for a tree-planting work program. One reason village was selected was that it is an upstream area and a source of water for people in study location and the study location action plan in village also included this. The same was true in village , where the location was… (unclear), which was one reason tree planting was focused there. We deliberately and routinely involved these parties, including the water resource management forum forum, so they understood the program. water resource management forum colleagues were involved even in CCRIW training. They participated in learning how to facilitate CCRIW and understood the expected outputs. We deliberately involved them from the beginning, not only after results had been produced.

Moderator: Thank you very much. Perhaps we can return to this if anyone remembers more. We will move on. This one is complete, so we will delete it. Now, “Increased knowledge among village and subdistrict Community Based Total Sanitation teams and village governments regarding CCRIW.” FGD Participant 02 could you explain how existing regulations influenced the knowledge ofCommunity Based Total Sanitationteams and village heads?

FGD Participant 02: All right. When we met with village or urban village officials and asked them to allocate funding, we had to explain that the request did not originate only from us. It was part of a regional target. This made it easier to advocate for what we wanted.

FGD Participant 02: That is what we tried to do in our locations, especially in study City, where the difference was most apparent. Study location was not like study location , where we had a long incubation period. Work in [Study Area 3] was new, and our interaction with them did not cover two full years. We strengthened the effort by explaining that regulations existed. We also supported the inclusion of these three issues in the EHRA study and the sanitation strategy document . This strengthened our advocacy.

Moderator: Thank you. Does this Ssanitation strategy document refer to study location City and its climate-change executive process, or to study location ?

FGD Participant 02: Both. This one concerns [Study Area 1], but in study location City… Actually, it applies to both. [Study Area 1]'s SSK already includes climate-resilient Community Based Total Sanitation in the document. In study location , we supported the inclusion of the three issues in the EHRA study so they would strengthen the sanitation strategy document to be prepared in 2025.

Moderator: Okay, thank you. The next part could take a long time. Perhaps after this, we should focus only on evidence. I will select the evidence, and no more than one additional hour should be needed. We have already exceeded the planned time by 30 minutes. Would it be acceptable to finish at 12:45? Or perhaps 12:30, so we can order lunch and eat immediately afterward. Could we finish at 12:30? Here, the ride-hailing cost is higher than the cost of the food. “Selection of training participants that involved vulnerable groups.” This concerns the community. I want to confirm with FGD Participant 10 based on the shared screen: Did participant selection increase the knowledge of the Community Based Total Sanitation team, or did it increase community knowledge?

FGD Participant 10: It was one of the factors. Why did I focus on participants? CCRIW focused on communities affected by drought and flooding. Therefore, building the capacity of vulnerable community members in those areas, including people with disabilities, was important because they were vulnerable to climate-change impacts. It was important not only to build the capacity of stakeholders, but also to involve and strengthen vulnerable groups… (unclear), because they would be most affected by those issues. In the previous CCRIW activity, participants were selected together with the MKGS team and focused on youth, middle-aged people, older adults, and others. However, the focus remained on areas affected by the two hazards, flooding and drought. That was the main point of CCRIW.

Moderator: Okay, so it concerned the community. I only wanted to confirm.

FGD Participant 10: Yes, the community. In several villages, the village government and village head were also involved in the process, but the primary focus at that time was civil society.

Moderator: All right, thank you. I will revise the item above. We already discussed improved village infrastructure. We will discuss this item later. Okay. I will use a different color for the community-related items. “Communities understand resources and potential climate changes.” This concerns increased CCRIW knowledge. These items are actually similar, so I will move this one upward. The other one… yes, it is the same. I will delete it and combine it here. Then there is “Inclusivity in community activities related to CCRIW,” “understanding of action plans,” and, um, “inclusivity in community activities related to CCRIW.” These are also connected.

FGD Participant 10: The inclusivity item is also relevant to what I said about participants, [FGD participant], from…

Moderator: Mm-hmm, yes, this one. I will use this one because it concerns increased understanding. So these items are connected: action plans and concern. Yes, I will place this… to make it easier. The action plan is actually related to this. Now I want to ask briefly about governance issues, improved infrastructure at the village and community levels, and inclusivity. I will move this item upward. Imagine that the orange items are the higher-level results than… (unclear) what colleagues mentioned earlier.

Please look at the shared screen. I will zoom in. Would you agree that, at the community level, increased knowledge and inclusivity support these two results, and then support the results over here? Is this the relationship between increased knowledge and community inclusivity in CCRIW, or does anyone have another suggestion? We are nearing the end, so I will delete this item. Okay, we will clean this up. Does anyone have suggestions about these relationships? Or are they acceptable? Involvement… I have been looking in different places. While I revise this, please begin preparing the strongest evidence. Think about [Study Area 2] and [Study Area 1]. If we write a story, which areas should be highlighted? Select no more than three in [Study Area 2] and three in [Study Area 1]. Please send private messages in the group chat and discuss within each district group which evidence should be highlighted

at the higher level…

FGD Participant 10: Do you mean the blue points should be highlighted, [FGD participant]?

Moderator: The red points.

FGD Participant 10: Why are they red?

Moderator: Select stories with strong processes and results. Which should be highlighted? It will be helpful when we compile and communicate the findings. This is the form that remains after the discussion.

FGD Participant 02: [FGD participant]…

Moderator: Okay, please go ahead.

FGD Participant 02: Instudy location , there are two locations. Should the three stories include two from study location and one from study location , or three from study location and one or two from study location ?

Moderator: Yes, we can propose one or two from study location . That would also be useful because…

FGD Participant 02: Okay, so for study location do we have four in total, three from study location and one from study location ?

Moderator: Yes.

FGD Participant 02: Okay, we will discuss it in the group.

Moderator: Yes, while we wait. Now the community section is complete and the government section is complete. Let us discuss this section: village funds accommodated the action plans, activity funding was shared, and the knowledge of village teams and village heads increased. May I propose the following relationship? Increased knowledge led to village-fund allocations. Village funding then led to improved infrastructure at the village level. Funding shared by district offices also led to improved infrastructure and perhaps activities, not only infrastructure. Reforestation is not infrastructure, correct? Would this arrangement be acceptable?

FGD Participant 03: Yes, that is acceptable.

Moderator: All right, thank you. Excellent. Later, FGD Participant 06 and I will organize it neatly so it can be translated into English or downloaded. Now we will identify evidence. I apologize that [local health worker's] item and the school items are not included. We have already addressed the district budget study. Now, which evidence should be highlighted? Is there something related to village funds? There is evidence at the village level and household level, including community self-help contributions. Finally, there is evidence related to inclusion. What happened regarding disability inclusion in study location ? We can select examples later. The information should include… this can also be homework… or we can discuss the location, person's name, and story to highlight. Okay. At the village level, was the result a program or physical infrastructure? Reforestation and so forth. We already have a community-level example. Inclusion should be here. We can discuss the details later and identify whom FGD Participant 06 and [FGD participant] needs to interview. I need to use the restroom. While waiting for FGD Participant 02 I want to ask briefly about… CCRIW included an initial discussion about workloads and similar matters. How did you see… okay, before I go there, should the evidence be homework, or should it be submitted today? Or should you first discuss it with your teams?

Hello, hello. Would you…

FGD Participant 10: In my opinion, we should discuss it today. There is only one more item, correct?

Moderator: Yes. Please discuss it. I still have another question. Can this be submitted today after the discussion? Do you have suggestions besides these three items? If not, we can enter a more focused discussion session. Oh, FGD Participant 02 has returned. During the next 30 minutes… I want to ask several questions about the program. Are there any suggestions regarding the green items? If not, please send the data to [FGD participant] later, and [FGD participant] can compile it. Or should [FGD participant] give the draft to FGD Participant 02? Only provide the key points for the story to be highlighted. Later,

our consultant team will write the story. Is that acceptable? Once finished, everyone may leave Miro. I can save the Miro board and stop editing. Now participants cannot edit… oh, it can still be edited… manage access… Okay, to prevent further changes. My first clarification question about these results is this: How strongly did programs outside CCRIW influence the results you observed? Honestly, the CCRIW community activity lasted only one day. Did larger programs outside CCRIW, such as water resource management forum the established Community Based Total Sanitation program, water and sanitation GEDSI , or another local-government program, help make the targets or outcomes possible, including improvements in village and community infrastructure or programs? Could you explain how strongly Plan's existing five-year Community Based Total Sanitation with GEDSI program influenced these achievements? Did it have no influence? What about water resource management forum ? Please share your views.

FGD Participant 02: Hello. To explain simply, these were closely connected. The fiveCommunity Based Total Sanitation pillars include ensuring water availability, which addresses Pillar Three. This gave us a strong foundation for advocacy. We could explain that if we did not act now, communities in study location would experience the consequences in future years. This was especially true in study location . In study location City, when we reviewed the training results and went directly to implement activities at the locations, we asked what changes had occurred in study location city City's water conditions over the previous five or ten years. Participants described the severe drought that [Study Area 3] experienced at that time. This gave us stronger leverage for advocacy with several regional government agencies.

Several agencies were also highly concerned about CCRIW-related issues. However, they were constrained by funding, and their knowledge was still limited. Their educational approach also differed greatly from ours. They tended to use a face-to-face approach in which the person who considered themself most knowledgeable spoke until finished. Our approach instead elicited extensive information from participants. We used these approaches in study location , which is why our waste-related issue progressed so rapidly. That is what we experienced in study location City. That is my view. Perhaps others have additional opinions.

Moderator: Please go ahead, colleagues from study location . How did other Plan programs or programs outside Plan influence CCRIW's success? The CCRIW activity lasted only one day, although advocacy and socialization also occurred. Did other programs contribute, and how did they influence achievements at the community and village levels?

FGD Participant 10: In my view, CCRIW served as the beginning of a process to map priorities based on existing conditions and climate-change impacts in communities, and to identify action plans proposed by communities to reduce those impacts or… Although the process lasted only one day and involved a small number of people who were expected to represent affected communities or areas, it was important because it enabled participants to discuss possible steps together. The process then became an entry point for further action. To be honest, several plans emerged directly during each stage of the CCRIW process. The village-level compilation of action plans generated through the CCRIW guidance tool actually contained far more ideas than the summary results viewed by village. This may be because the one-day process was facilitated by UF teams in each area, [FGD participant]. We were trained together in provincial close study location with colleagues from university and then applied the approach with subdistrict teams. The subdistrict teams implemented it directly in each village. In that sequential process, I believe we did not fully implement every stage of CCRIW. When we conducted a simulation in one village, if we examined the guidance in detail, ideas actually emerged at each stage, [FGD participant]. From mapping through the final discussion of the desired future, numerous possible action plans emerged. However, when CCRIW teams facilitated implementation, not all of those ideas appeared fully. Even so, the ideas that emerged described shared needs.

FGD Participant 10: In my view, the facilitation process requires improvement. Personally, I cannot guarantee with complete certainty that CCRIW facilitation was delivered exactly according to the guidance. That is the first point. At a minimum, however, everyone participated in the process and learned, including members of the BP team. The results agreed upon collectively became an entry point for us to seek support from stakeholders, including APBDes, the closest funding source accessible to communities through village heads, as well as other sources. Other sources cannot be ignored because government agencies such as the public works and environmental offices also have substantial budgets. The challenge is ensuring that advocacy reaches our program locations. Regarding whether CCRIW should be adopted by local government, we deliberately invited members of the water reseource management forum. The person who attended the full training and practical sessions was a division head at Regional Disaster Management Agency and a member of the destructive-power division of the water reseource management forum. He explained that the CCRIW process was relevant to and very similar to disaster-related Regional Disaster Management Agencyprocesses, although it included slight modifications. The resulting processes need to be collaboratively integrated. In study location , for example, one institution mentioned earlier was the study location traditional institution. This newly established institution, inaugurated by the sultan, focuses on environmental issues, including climate change. Have we completed every action plan produced? In my view, no. Much work remains. This is one reason for extending the program period by two years. Two years is very short for realizing the large ambition that all action plans will be achieved. Considerable work remains. It would be very beneficial if local government adopted the approach, but the question is how urgently they view the process. CCRIW takes nearly a full day, often from morning until three or four in the afternoon. Local governments may not find such a long participatory process attractive, even though it is essential for mapping conditions and identifying actual needs. Honestly, our work was not completed in two years. Some action plans were accommodated in village budget through various advocacy methods, while others were not. At the community level, as [FGD participant] noted, only a small number of people participated in CCRIW. Whether knowledge reached everyone remains a question. Additional efforts are needed, perhaps through mass media or socialization, to increase awareness of the plans and local conditions. CCRIW cannot stand alone, and a one-day process cannot solve everything. Nevertheless, in my personal view, that one-day process was an essential starting point for identifying community needs, particularly among people living in areas affected by climate-related disasters. That is my view, [FGD participant].

Moderator: Thank you, [FGD participant]. We will now focus broadly on enabling and inhibiting factors. We have discussed how previous disasters were used as enabling factors, funding constraints, implementation constraints, and the difficulty of making the adoption process attractive. Has CCRIW been adopted in any area, village, or subdistrict?

FGD Participant 10: Not yet in study location , [FGD participant].

Moderator: What about study location ?

FGD Participant 03: Hello. The complete CCRIW method has not been adopted. However, some results from the steps discussed in CCRIW have been adopted and followed up. The full set of activities based on the existing module has not been adopted. Based on our experience in study location , CCRIW activities using the module take a relatively long time. We felt that the approach did not fully suit participants' backgrounds. If possible, it might be condensed or modified because, in our view, the duration used thus far has been quite long.

Moderator: Thank you very much, [FGD participant]. My understanding is that the guide was originally intended to be implemented through separate activities on different days. FGD Participant 03 was one of the initial authors of the guide with [program specialist]. When we piloted it, the activities took place on different days, correct, FGD Participant 03?

FGD Participant 03: When it was piloted in [pilot locality], the activities did occur on different days. In field implementation, however, they were completed in one day.

Moderator: Mm-hmm. So, as discussed earlier, the guide and Plan colleagues' implementation were not fully aligned, as FGD Participant 10 noted.

FGD Participant 10: Hello. We revised the guide together with [program specialist]. We discussed each stage and revised parts of the implementation methodology. To assess the effectiveness of implementation, each region piloted the agreed guide. The study location team piloted it in one hamlet in the Village. We found that the outputs of several stages could be achieved in one day. The important issue was the quality of facilitation at each stage, which was necessary to ensure that each stage produced its intended outputs. If implementation diverged from the guide, the stage-specific action plans did not emerge clearly. In study location , different people took responsibility for facilitating different stages, so it functioned as a learning center. If the guide is read carefully, the process can be effective because facilitation at each stage is crucial. The quality of the process must be maintained.

Moderator: My question is this. When all four activities are compressed into one day, they appear to constitute a single product. But what if local governments were offered the four activities as separate products and a village could adopt only one? Which one would be most feasible and appropriate to promote? In reality, when everything was delivered in one day, even Plan's training for subdistrict Community Based Total Sanitation teams did not produce optimal results because four components were forced into one day. If the components were separated and offered individually, which one or two of the four stages would you promote, and why? The four activities included MKGS, climate change, triggering and mapping, resources, and future visioning. As FGD Participant 10 said, the complete one-day package may not be sufficiently attractive for advocacy and adoption.

FGD Participant 03: Hello.

Moderator: There is a transect walk. Mm-hmm. Please go ahead.

FGD Participant 03: We from study location would like to provide some information.

Moderator: Okay.

FGD Participant 03: When we look at village activities and participants' enthusiasm across the four components, they were very enthusiastic about the first activity, the introductory information. Participants came from several age categories. Older adults described how rainy seasons used to be, how forests had once been extensive, and similar matters. They were very enthusiastic. Second, participants assessed facilities. Through that assessment, they learned that people's needs differ. Some needs had not previously been recognized. We invited participants to walk through the area and identify needs that might be addressed. In my observation, this activity received considerable attention from participants. If, as [FGD participant] asked, we must choose what to offer, those would be my selections.

Moderator: Do others have suggestions?

FGD Participant 10: Please, FGD Participant 01 or FGD Participant 08.

Moderator: Earlier, someone mentioned resources. What do others think about resource mapping?… the item that appeared here… Okay. If there are no comments…

FGD Participant 10: In my view, [FGD participant], the four steps are interconnected. Therefore, asking which of the four stages should receive the highest priority

FGD Participant 10: is difficult. The process begins with mapping. Climate-sensitive mapping is important because communities can identify affected areas and other impacts, including climate effects on people with disabilities and the implications for sanitation infrastructure. The third step also concerns the MKGS team context. The fourth step focuses on five types of resources, including what communities and villages possess and can mobilize to reduce the impacts. In my view, Step Four leads to

compilation of action plans from each preceding step and identifies what can be done collectively. If the steps are offered separately and a community is offered only impact mapping, participants can map the affected locations and the types of impacts. However, they would not map their available physical, economic, social, and other resources. They might then feel helpless even though they possess resources they could mobilize. Please correct me if I am mistaken, but the four stages form an integrated whole for mapping conditions related to these issues. Each step has a different emphasis, but together they allow us to plan with communities and identify their future needs.

Moderator: All right, thank you, FGD Participant 10. My next question particularly concerns MKGS. We conduct workload analysis and analyze who has decision-making authority during disasters. To what extent did that activity increase awareness or change decision-making? Did increased awareness result primarily from the earlier community based total sanitation GEDSI work rather than CCRIW? Or did CCRIW influence disaster-related decisions to modify facilities? Honestly, in our earlier mapping discussion, no one mentioned decision-making or differences in power and authority. Inclusion appeared in the discussion, but equal decision-making did not. What do colleagues think? If there is no response, perhaps this outcome is difficult to achieve through a brief activity. Does the village head remain the primary decision-maker, as usual, or has there been some change? Perhaps people now understand that vulnerable community members should be involved in decision-making. Are there examples of their greater involvement? Such an example could be selected as an evidence story. Or has the change been limited to awareness that vulnerable people should be involved, without their actual participation in decisions?

FGD Participant 10: In my view, we need to cross-check the facts. For example, did village-level decision-making change? Before Plan entered, were people with disabilities, vulnerable groups, or disaster-affected groups involved? Did that change after CCRIW? Perhaps colleagues such as FGD Participant 01 and FGD Participant 08 in [Study Area 2] have information.

In the CCRIW tools, this issue actually appears in detail in Step Three. That step examines the accessibility of affected infrastructure, who is repeatedly and most severely affected, and decision-making indicators. However, in Step Four, when action plans and resources are selected, some plans that emerged in Steps One, Two, and Three are not always revisited carefully. Each stage can generate ideas, and when action planning occurs in Step Four, many plans from each stage should be included. Personally, I recognize that CCRIW implementation quality was not complete. There was a cascading process: [program specialist] trained us, we trained subdistrict community based total sanitation teams, and those teams facilitated CCRIW at the village level. People cannot fully understand and implement a guide immediately after only one, two, or three days of training. This remains work for all of us. We need to strengthen the quality of the CCRIW process.

Moderator: Thank you. Broadly, FGD Participant 03 also explained that communities accepted the approach and that it was suitable for local implementation. As [FGD participant] noted, BPBD's own program is very similar to CCRIW. Are there suggestions about what should be changed, improved, or revised to make CCRIW more suitable, or is it already appropriate?

FGD Participant 10: In my view, [FGD participant], local-government programs such as Destana, or Disaster-Resilient Villages, could combine their tools with CCRIW because the approaches are quite similar. Disaster-Resilient Villagesfacilitation includes village-area mapping and uses several highly relevant tools. There may also be a related village program called FORKLIN [unclear]. In my opinion, the tools could be combined. Among existing government programs, CCRIW tools appear most closely related to regional disaster management agency Disaster-Resilient Villagesprogram. I have not identified equally close links with programs in other regional agencies. A regional disaster management agency emergency-management division head also said that the approach was similar to Disaster-Resilient Villagesfacilitation.

Moderator: Thank you, FGD Participant 10. I will now ask each person a broad question. First, however, I want to confirm two matters. The first concerns advocacy for action plans. Communities prepared the CCRIW action plans, and the collected format included plans for advocacy with village and district governments. Without Plan's support, did communities independently conduct any advocacy, or were the achievements largely due to Plan's advocacy?

FGD Participant 03: Hello. In response, there were two types of advocacy after follow-up plans emerged from CCRIW activities. First, Plan supported advocacy through activities intended to expand opportunities. Village-fund advocacy was conducted by Plan champions with stakeholders at the district and subdistrict levels, involving pilot villages in discussions of agreed follow-up plans. Second, some advocacy did not involve Plan. After follow-up plans were agreed during CCRIW activities, village colleagues and community leaders independently implemented the plans. Therefore, both cases existed: Plan participated in some activities, while villages independently implemented other agreed follow-up plans.

Moderator: Were village officials present at the end of CCRIW activities?

FGD Participant 03: In study location , we required or strongly encouraged village leaders, officials, or secretaries to attend because they opened and closed the activities. At least one village official was present at the opening and closing. They legitimized or signed the follow-up plans agreed collectively. We wrote the plans, posted them, and left them in the village. Therefore, village officials certainly knew what had been agreed during CCRIW.

In practice, not every village followed up independently or through advocacy. However, based on our recent findings, most villages have begun implementing the agreed follow-up plans, either through our advocacy or independently.

Moderator: Thank you, [FGD participant]. In studi location was the process similar? Were follow-up plans legitimized by village heads?

FGD Participant 01: Yes. In Study location , the final CCRIW follow-up process was similar to that in study location . A village head or village consultative board chair had to be present, acknowledge the action plan produced through CCRIW, and sign it. We asked that the action plan be posted at the village office so it would remain visible. Regarding [FGD participant]'s question about the relative influence of Plan and independent advocacy, in my personal view Plan had a very large influence on advocacy for CCRIW plans. As FGD Participant 10 noted, Plan repeatedly raised the agreed action plans through advocacy at the district level and in routine subdistrict meetings or coordination meetings. We asked village heads to accommodate them in village budget . We repeatedly reminded stakeholders at the district and subdistrict levels, including subdistrict heads and evaluation teams.

FGD Participant 01: When we returned to the villages, we reminded them again. I believe Plan made a substantial contribution to advocacy and CCRIW follow-up.

Moderator: Thank you very much.

FGD Participant 10: As discussed earlier, during the 2023 CCRIW process, we continuously coordinated with the community and village empowerment departement Office to develop the regent's regulation providing guidelines for preparation of the 2024 village budget , because it formed the basis for village heads to prepare plans and propose budgets. We asked whether a draft existed and requested and reviewed it in detail. Climate-change issues had already become a requirement from the Ministry of Villages. Once that requirement was included, we were not concerned about its adoption. The village-level requirement would exist in the regent's regulation and village budget guidance. We continued safeguarding the process before the regulation was enacted. Once enacted, during advocacy we only needed to ask the head of the community and village empowerment departement Office to explain it. If Plan spoke, village heads might ask, “Who is Plan?” However, when the community and village empowerment departementhead explained that the regulation required budgeting, the process became easier. Village heads have different characteristics. The process was not as easy as it might appear. Even if they attended meetings and understood the process, they did not necessarily include the items because many other attractive priorities competed for village budgets.

Moderator: Thank you, FGD Participant 10. This is the final question. May I ask everyone to continue until 12:45? Please order lunch immediately… [laughter]… What was that, FGD Participant 02? Each person will have only one minute so this does not take too long. What is the greatest challenge to changing community behavior and the greatest opportunity or enabling factor that could help communities change their behavior and make facilities more resilient to climate disasters? I will name one person, who will answer for one minute and then name another person to respond. I will begin with FGD Participant 04. After finishing, FGD Participant 04 should nominate the next respondent.

We have discussed government extensively, but what is the greatest challenge at the community level? What enabling factor should we consider or create? Please go ahead, FGD Participant 04.

FGD Participant 04: Thank you. Regarding challenges to changing community behavior, I am thinking particularly of study location City. Changing people's understanding and ways of thinking is very difficult, especially because people need to see concrete examples of what must be done. Several parties also need to collaborate and serve as resource persons during community socialization. That is all. Thank you.

Moderator: Whom would you like to nominate? Please focus on concrete examples and evidence. Whom would you like to nominate next?

FGD Participant 04: FGD Participant 03.

Moderator: Okay.

FGD Participant 04: Hello, hello.

FGD Participant 03: The main challenge in changing community behavior is that we may ask people to act, but many expect examples or, more bluntly, material assistance. Assistance is understood as something physical. That is perhaps the greatest challenge. One opportunity is collaboration… That is all.

Moderator: Whom would you like to nominate?

FGD Participant 03: [FGD participant].

Moderator: Please go ahead.

FGD Participant 03: Our ultimate objective is to change community behavior so people become aware and follow up on climate-related workshop activities. The challenge is whether we have sufficient authority and time to conduct continuous monitoring. Behavioral change occurs when accompaniment is sustained. With many villages and [Study Area 1]'s challenging topography, continuous support is a major and difficult challenge. One opportunity is the regulatory framework. For example, the 2025 [study district] long term development plan discusses inclusion, climate resilience, and health. If these matters are included in the RPJPD, derivative regulations will follow and strengthen and simplify advocacy. Another opportunity is Plan's strong reputation in [study district]. It is well known as a trusted nongovernmental organization, and we can help realize aspirations in [study district]. Because we are trusted, future advocacy will be easier. Thank you.

Moderator: Thank you, [FGD participant]. Whom will you nominate?

FGD Participant 03: I will nominate my most handsome colleague, [FGD participant].

Moderator: Yes, FGD Participant 03. Please focus on the community level.

FGD Participant 03: What Informants and said is similar to my view because the three of us work together. The challenge is changing behavior when we arrive with only words. Communities have developed an expectation that when outsiders arrive, they will bring physical assistance. I consistently try to convince communities that our discussion is also a form of assistance. We explain that this is collaborative work. We may do something at the village level, while colleagues at the subdistrict level undertake related activities. The key is building understanding. The opportunities have been explained by [FGD participant] and [FGD participant]. One opportunity I have used is the alignment between government and church programs. The church also recognized the importance of this issue, and the diocese designated 2024 as the Integral Ecology Year. I explain that Plan is not the only organization concerned. Government also considers the issue important, as reflected in the SDGs, and the church recognizes that climate change concerns both congregations and communities. The church supports this work through the Integral Ecology Year.

Moderator: Thank you. It is already 12:39, and I do not want to extend the session further. Does anyone want to share an example of what changed at the community level and why, in relation to CCRIW, without government assistance? Is there an example, or perhaps none? Were all household-level changes identified in FGD Participant 05's monitoring results due to government support, or were there other causes?

FGD Participant 01: They still required encouragement, [FGD participant]. It was not necessarily physical assistance. Encouragement from government was most important because communities needed to be motivated.

Moderator: What form did government encouragement take, and what results did it produce?

FGD Participant 01: It included public appeals and village-level competitions, such as cleanliness competitions during Independence Day celebrations. Physical assistance was not necessary. When such competitions occurred, activities usually proceeded on their own. That was our experience in study location .

Moderator: Did the Independence Day competition include climate-change elements?

FGD Participant 01: I did not follow the last competition closely, but it usually concerned cleanliness in villages. Waste management in an Independence Day competition can be related to climate change. Communities here have changed considerably since we began work in late 2018. Some changes resulted from government support. Many villages now allocate funds for waste management and related activities.

Moderator: All right, thank you.

FGD Participant 02: May I add something? Regarding what FGD Participant 01 said, we strongly experienced the importance of engaging key figures in communities. Those key figures then encouraged others. In study location , this relied more on gender based violen members. In study location , we worked across religious communities with Muslim leaders, sisters, and church members. They encouraged action. Colleagues from Pertamina also approached us on their own to discuss waste management because they were curious about how we managed waste successfully. We are also working on data. Although schools were not the dominant focus in [Study Area 3], we entered schools at the initiative of urban village colleagues, who asked us to visit periodically because schools are large communities in study location City. The full story is complex and there are too many examples to describe. Strong stakeholders are essential. Once key figures are engaged, activities can continue even without our presence. We often complain that we have only a small number of staff, but despite our limited numbers, we have achieved substantial results. To be honest, we also completed work beyond the formal targets. In [Study Area 3], colleagues at the water utility and PDM made major contributions to ensuring water availability. The public works office may focus on constructing household connections regardless of whether water flows, while water-utility colleagues continuously make repairs. The same applies in location study . Engaging the water-utility director, the PKK chair, and other key figures creates a major opportunity to transform situations previously considered impossible. Those are our strengths. Thank you.

Moderator: Thank you. We have one minute remaining. Does anyone want to add an example of good practice at the community level, particularly involving disability? Was there a change related to climate change, or a testimonial?

FGD Participant 02: I can add an example from location study . The PAR program in location study had ended, but in location study , [program staff member] continued accompanying participants, and they carried out many interesting activities from the subdistrict to community levels. Many people with disabilities collected very large quantities of waste. Some designed their own motorcycles for transporting waste. They also conducted replanting. Regarding disability inclusion in location study the progress is unquestionable. Among the 22 districts and cities in location study may be exceptional. Communities progressed beyond ramps and handrails and created overhead supports that people could use to move by holding or crawling along them. We feel satisfied with the work completed over six years. Although some people consider that period short, field colleagues made substantial progress. That is all. Others may add something.

Moderator: Thank you very much, everyone. It is 12:46. I will stop the recording.
