# Supplementary material for: Implementing a climate-resilient and inclusive WASH guidance in Indonesia: A mixed-methods case study
Source: PLoS One. 2026 Sep 28;21(9):e0359266. doi: 10.1371/journal.pone.0359266 (PMC13618932; doi:10.1371/journal.pone.0359266)
Supplement: Supplementary file 6 [file pone.0359266.s006.zip › S6 Appendix Transcripts/KII-01_Deidentified_English.docx]

Interviewer

Participant

Interviewer: Okay, sir, may we continue? We only want to have a discussion about the condition of inclusive and climate-resilient Community Based Total Sanitationin this [study village]. You are a village official. How long have you held that type of position?

Participant: [for many years].

Interviewer: What is your current position?

Participant: [for approximately a decade].

Interviewer: All right, sir. I will first read the protocol.

My name is [interviewer], and I am a researcher from [research institution].

This study aims to identify the factors that influence climate-change responses in implementing inclusive Water, Sanitation, and Hygiene (Community Based Total Sanitation ) programs in Indonesia. The study will improve our understanding of the enabling factors and barriers to achieving resilient and inclusive Community Based Total Sanitation services and to making decisions in village program planning.

Today, we are examining community experiences with access to inclusive and climate-resilient Water, Sanitation, and Hygiene (Community Based Total Sanitation), including solid-waste management, food hygiene, and wastewater management. Please keep these components in mind when answering the questions.

This discussion will use the terms extreme weather events and climate hazards interchangeably. When I use the term climate hazard, please remember the following examples. Resilience means being able to withstand a climate disaster, while inclusiveness means involving all parties, including marginalized groups and those who are usually rarely involved. [Continue reminding the participant about this concept.]

This discussion will take approximately one hour and will be recorded. The audio recording will be stored securely while in use, transcribed, retained for one year, and then destroyed.

Participation in this study is entirely voluntary. You may choose not to participate, skip any question, or stop the interview at any time without any consequences. Your identity will be protected because any information you provide will be kept confidential and will not be shared with anyone. Please tell us if you need any special support to participate comfortably.

Thank you for participating in this discussion. It would be very helpful if you answered honestly so that we can provide better recommendations regarding the program procedures. Every comment you provide will be kept confidential. We will not share your responses with anyone. Your answers will not affect whether the village receives a grant or another program in the future. This is the consent form that you need to read and sign.

Do you have any questions about this discussion?

I will begin the recording now.

Interviewer: Okay. This interview has three parts. First, we will ask general questions about you. Second, we will ask about your experiences and perceptions of inclusive and climate-resilientCommunity Based Total Sanitation. The final part will be the closing. To begin, could you tell us how long you have lived in this village and describe your role?

Participant: I have lived in this village for several decades and have served as a village official for more than a decade.

Interviewer: Okay.

Participant: [The participant described a long progression through village-government roles before becoming a senior village official.]

Interviewer: Thank you, sir. When did you begin participating in STBM?

Participant: If I remember correctly, I have attended three meetings.

Interviewer: In what year did you first participate?

Participant: Perhaps during an earlier program period. I have forgotten, but the most recent activity was recent.

Interviewer: The more recent activity concerned the five Community Based Total Sanitation pillars, correct?

Participant: There were two activities about the five pillars. I even served as the, what is the term?

Interviewer: Triggering facilitator?

Participant: Yes, triggering.

Interviewer: What about inclusive and climate-resilient Community Based Total Sanitation?

Participant: I participated once.

Interviewer: At that time, did you participate in a workshop, mapping, a transect walk, or a group discussion?

Participant: There was no workshop. I only participated in the group discussion, not the mapping.

Interviewer: Could you describe your role during the group discussion?

Participant: What was it called? I have forgotten. It was...

Interviewer: A facilitator?

Participant: Yes, making, what was it called?

Interviewer: Metaplan cards?

Participant: Yes, that was it, during the group discussion.

Interviewer: What was your impression after participating in the activity?

Participant: My impression was that there was cooperation. It was not only my responsibility. Everyone cooperated and shared responsibility.

Interviewer: I would like to hear about your experiences and perceptions of inclusive and climate-resilient Community Based Total Sanitation. Based on your experience, what are the main challenges in implementing the five Community Based Total Sanitation pillars?

Participant: Some local residents may have limited concern about these issues. Our community is diverse, and some people's practices differ from ours. Some bathe in the river or behind their houses. Therefore, the challenge is how to help people understand cleanliness and hygiene.

Interviewer: What are the challenges related to water access?

Participant: In terms of water access, several places have difficulty obtaining drinking water, including one hamlet area. Parts of [local settlement] and [local settlement] also experience this problem. People there live in hilly areas. They dig wells, and some wells here are as deep as 27 meters. We have made some efforts to provide clean water, and part of the plan has been realized.

Interviewer: What types of extreme weather events often occur here?

Participant: Flooding occurs particularly in [local hamlet]. Flooding has also occurred here in several past years, including recently. In [local hamlet], however, it occurs every year.

Interviewer: In [local hamlet], which experiences flooding every year, what challenges do people face in accessing clean water during floods?

Participant: Not everyone is affected. Only certain areas are affected, so people can evacuate to unaffected places.

Interviewer: Some people are affected, but there are solutions because not everyone is affected. During flooding, are facilities damaged, such as water channels or wells? Does floodwater enter the wells?

Participant: Beds and other household facilities must be cleaned again.

Interviewer: In your opinion, what types of facilities should be built, such as what type of well, to minimize flood risk?

Participant: One solution is a managed clean-water system for which users pay monthly fees. Water comes from a source here and is distributed through pipes.Community-Based Water Supply and Sanitation water can be used for purposes other than drinking.

Interviewer: How long has Community-Based Water Supply and Sanitation operated here?

Participant:Community-Based Water Supply and Sanitationhas existed since that earlier program was introduced here.

Interviewer: Is there a village organization that specifically manages water? Is there also an Community Based Total Sanitation organization? Do both organizations have a chair and secretary?

Participant: PAMSIMAS has a management group in [local hamlet], but the group in another hamlet does not function very well. A group was established here at that time because of drought conditions. There is also an STBM team.

Interviewer: To your knowledge, do the water-management team and Community Based Total Sanitationeam divide their roles?

Participant: Yes, they generally consist of community members.

Interviewer: Are women and persons with disabilities involved in water management here?

Participant: Women are involved in water management only through Community-Based Water Supply and Sanitation, I believe.

Interviewer: What roles do the women hold? To your knowledge, do they also participate in decision-making? Are women active in the Community Based Total Sanitation team?

Participant: The STBM team includes representatives of women, persons with disabilities, and young people.

Interviewer: How does the community perceive climate hazards, such as flooding and water shortages, and how does it respond?

Participant: The community responds actively because we hold a village development-planning meeting every year. People are very enthusiastic about providing input during that meeting.

Interviewer: Do people report problems involving water conditions and retaining walls? Once these problems have been reported, how quickly do the water managers or Community Based Total Sanitationteam respond?

Participant: The STBM team submits a request to the village government concerning possible solutions. This year, thank God, the village head obtained assistance from [government housing program], and we are waiting for its implementation.

Interviewer: What form will the assistance take?

Participant: A drilled well in one area, because the other area already has one water point.

Interviewer: We will now discuss governance and community involvement. In your opinion, is the community involved in selecting options and making decisions concerning Community Based Total Sanitation?

Participant: Yes.

Interviewer: Is the community also involved in decision-making concerning water management?

Participant: Yes.

Interviewer: Did community involvement begin after this program, or had village residents already been active in STBM and water issues?

Participant: They had already been involved, but the STBM team increased their understanding.

Interviewer: Who makes decisions when flooding occurs?

Participant: The village head. He immediately contacts the disaster-response team.

Interviewer: Does the village head contact the community directly, or does the information first go through the hamlet head?

Participant: It goes to the neighborhood head and then through the hamlet head.

Interviewer: So the village head makes the decision, but communication first goes through the hamlet head. Who has the greatest role in decision-making concerning Community Based Total Sanitationand water, the hamlet head or the community?

Participant: In practice, all decisions ultimately come from the village head.

Interviewer: Could you provide an example of an activity conducted jointly by the community, village government, and Community Based Total Sanitation team to address sanitation and water problems?

Participant: As I mentioned, after the Community Based Total Sanitation program was introduced, the village government allocated sanitation funding. We identified households that did not yet have sanitation facilities and provided assistance. If I remember correctly, it was a form of stimulus assistance. The important point was that sanitation facilities were actually constructed at the household level.

Interviewer: The village head made the formal decision, but were there efforts to involve the community and reflect its interests? How was it ensured that the village head's decision was based on community participation?

Participant: Community members participated by providing a great deal of input, especially concerning...

Interviewer: We will now discuss institutional relationships. You explained that sanitation funding was allocated in [local hamlet]. Did it come entirely from the village fund?

Participant: Yes, [village funding was allocated].

Interviewer: In addition to the funding, are there examples of collaboration among the STBM team, village government, and community to ensure that water remains available during disasters, such as floods or droughts?

Participant: In terms of collaboration with the community, flooding does not last very long. Usually, after one day, the facilities can be used again.

Interviewer: In your opinion, do characteristics such as being elderly or having a disability affect people's participation in inclusive and climate-resilient Community Based Total Sanitation?

Participant: Only to a limited extent. There are still a few attitudes or characteristics that are difficult to change.

Interviewer: Have they been given opportunities but chosen not to participate, or is there another reason?

Participant: Most people here are farmers, but generally the people whom we invite participate sufficiently.

Interviewer: So they have been given opportunities, although they may not always be able to contribute. In your opinion, who are the key actors with the authority or influence to make major decisions about clean-water and Community Based Total Sanitationservices in this village?

Participant: For most problems, the hamlet head is the first person involved. If the matter cannot be resolved at that level, it is referred to the village head.

Interviewer: Are there differences in power among the village head, hamlet heads, and community members that affect people's ability to obtain water or maintain better Community Based Total Sanitation conditions during floods?

Participant: No. The situation is reasonably secure.

Interviewer: Regarding vulnerability, does poverty or disability affect people's ability to obtain clean water or otherCommunity Based Total Sanitation services?

Participant: The issue exists to a limited extent and depends on people's financial capacity. It affects some people in the mountain hamlets, but not those who use Community-Based Water Supply and Sanitation

Interviewer: So the main challenge for poor people and persons with disabilities is financial capacity. Are there any other barriers?

Participant: When there are other barriers, we provide assistance and assess their sanitation needs.

Interviewer: Are there local rules concerning sanitation or women's participation, such as rules that women may not participate or requirements that women must be included?

Participant: When regulations are prepared, we establish a 30 percent participation standard.

Interviewer: In your assessment, are village residents aware that climate change is occurring?

Participant: They have a limited level of awareness.

Interviewer: Could you describe the difference between people who are aware and those who are not? How do those who are aware respond?

Participant: Those who are aware have some capacity to understand climate change, particularly people with more education. We also explain what preparations should be made during the rainy season and what should be done during the dry season.

Interviewer: But do they understand that changes in rainfall and flooding are related to climate change?

Participant: Some people can predict that certain months are part of the rainy season and that other months mark the dry season.

Interviewer: That resembles the traditional knowledge you mentioned earlier. Besides traditional knowledge about climate change, are there traditional practices for addressing flood impacts?

Participant: At a minimum, we conduct simulations, such as a tsunami simulation because some areas are close to the coast. We also discuss what should be done during the rainy season.

Interviewer: Have these practices existed for a long time, and do people continue practicing them rather than treating them only as beliefs?

Participant: A tsunami has not occurred here. During the rainy season, however, people continue to participate in activities outside their homes.

Interviewer: People understand that the weather is changing, but they may not understand climate change itself. What prevents them from understanding climate change?

Participant: [Unclear response.]

Interviewer: When flooding occurs, what attitudes or behaviors do you observe, particularly regarding clean water and Community Based Total Sanitation? During a disaster, people may be reluctant to use facilities located behind the house, for example. How do they respond?

Participant: The village provides whatever assistance it can. Mutual assistance remains strong in this community.

Interviewer: People report the problem and the information is forwarded. Because flooding occurs repeatedly, what do residents themselves do to anticipate it? They are accustomed to the flooding, so do they have their own preventive measures?

Participant: The first measure is river normalization, which we have conducted frequently. River normalization changes the flow of floodwater and increases the channel's capacity. We have included this activity in plans since the beginning of the year and are waiting for several proposed activities. The challenge is considerable, but implementation may be possible.

Interviewer: People understand the risk, but does that risk affect their decision to act? Do they take any action themselves, or do they only report the problem and wait for the government to address it?

Participant: Our first response is to do what we can, such as constructing an embankment.

Interviewer: As a village official and Community Based Total Sanitation participant, what concerns do you have that may affect the ability to address flood risk?

Participant: The first factor that affects us comes from the... sector. We are concerned about conditions when work begins. Therefore, we provided assistance, and last year we also implemented...

Interviewer: How strongly do you support the climate-resilient Community Based Total Sanitation program?

Participant: It is related to changing people's mindset...

Interviewer: During the program's implementation here, were there any elements that were inappropriate or unacceptable to the community?

Participant: Some people found it difficult to accept the guidance and change their way of life. The challenge involved changing the community's mindset and introducing a cleaner way of life.

Interviewer: So many things were considered inappropriate at the beginning?

Participant: Yes, many things were initially considered inappropriate, particularly when we first instructed people not to practice open defecation.

Interviewer: Has the village adopted the climate-resilient Community Based Total Sanitation program during the past two years? For example, has it adopted gender-equality monitoring?

Participant: Comprehensive STBM mapping has been conducted.

Interviewer: In your opinion, what actions are needed to adopt this activity effectively? When Plan first comes to the village, what should be done so that the program is accepted and adopted?

Participant: Before the program, the village had already made some Community Based Total Sanitationprogress. When commitments and other requirements were introduced, we assessed whether they had been implemented. We then inventoried the conditions of all households in the surrounding area.

Interviewer: What is the most effective strategy for encouraging the government to implement this program? Could incentives or competitions be used?

Participant: We hold an activity every year. For several years, there has been a competition among neighborhood units to stimulate community participation.

Interviewer: Who organizes the competition for each neighborhood unit? Is it organized by the village government?

Participant: We allocate funding for it every year.

Interviewer: How suitable is this program for implementation in your village so thatCommunity Based Total Sanitation can become inclusive and climate-resilient?

Participant: The earlier Community Based Total Sanitationprogram was suitable. The newer Plan program has operated for only two years.

Interviewer: Could you explain why the earlier program progressed well but the current one has not?

Participant: It concerns the community's understanding...

Interviewer: The earlier program already provided a foundation, so why has the second program not developed as well when it is linked to climate change?

Participant: Disasters are sometimes difficult to predict. Making changes requires addressing the issues I described, such as river normalization and durable protective structures. Assistance could be provided to anticipate flooding, for example by constructing gabions along riverbanks.

Interviewer: In your opinion, the community may not yet understand climate change, although people experience its impacts. Changes under the five-pillar program, such as ending open defecation or changing bathing practices, were visible, but changes related to climate resilience are less visible. It is fine; we are simply having a conversation.

Participant: Please tell me if anything I say is incorrect.

Interviewer: That is precisely the information we need, so that we understand the actual conditions and recognize that the changes may not yet be very significant. Has the village government modified the current program so that it can achieve success similar to the five-pillar program?

Participant: We can only explore possible measures. Because the changes require funding, we can currently install only limited temporary measures using the resources available.

Interviewer: I would like to ask about funding. Does adopting climate-resilientCommunity Based Total Sanitation create a financial burden for the village or require additional expenditures?

Participant: No.

Interviewer: But you mentioned that the village had allocated funding.

Participant: Yes, funding is allocated to support the activities.

Interviewer: Do you have suggestions for implementing inclusive and climate-resilient Community Based Total Sanitation at lower cost, including village-level activities?

Participant: We motivate the entire community. Residents are accustomed to contributing, and any funding we provide is only a limited stimulus to support continuity.

Interviewer: What strategies could be used to ensure that Community Based Total Sanitationactivities continue?

Participant: Activities should continue every year. There are also programs that can use village funds, as you mentioned earlier.

Interviewer: Besides financial support, what human resources or capacities are important for sustaining the program?

Participant: The first requirement is mutual cooperation and collective work.

Interviewer: We will now discuss the intervention's results. In your opinion, has the inclusive and climate-resilient Community Based Total Sanitation program affected the community's ability to obtain clean-water and Community Based Total Sanitation services, or did residents already have these services before the program?

Participant: People had already used clean water from stone-lined dug wells. Many residents also use bottled water, and Community-Based Water Supply and Sanitation is available.

Participant: The use of bottled water has increased considerably.

Interviewer: What effects has the climate-resilience component itself had on access to water and Community Based Total Sanitation?

Participant: It has had many effects. During the dry season, many existing wells dry up.

Interviewer: What effects have appeared since the program was introduced? Has the program changed how people view Community Based Total Sanitation or the negative effects of climate change?

Participant: There have clearly been effects.

Interviewer: Before the climate-related Community Based Total Sanitation program existed, were conditions different, or were they generally the same?

Participant: The effects already existed. We also have a tree-planting program. We discussed with the subdistrict government a plan to procure seedlings and plant them throughout the [study village] subdistrict area. At that time, we allocated 25,000 [units unclear] for tree planting in four villages.

Interviewer: Allocating funding for tree planting represents a change. What strong motivating factor caused the village to change so that water andCommunity Based Total Sanitation facilities could become more climate-resilient?

Participant: The plan is to add more water points, including additional dug wells and drilled wells.

Interviewer: But those measures will also be ineffective if the groundwater-recharge area is not in good condition, correct?

Participant: The current season is actually the best time to dig wells because the groundwater level is relatively high. During the dry season, it falls.

Interviewer: Turning to village programming, has inclusive and climate-resilient Community Based Total Sanitation influenced water and sanitation planning in the village?

Participant: Yes. The climate-related Community Based Total Sanitation program was introduced before we prepared the village plan. Its presence therefore led us to adjust the budget during the village deliberation and planning process.

Interviewer: Besides the newer STBM program, the five-pillar Community Based Total Sanitation program also influenced the village. Did five-pillar Community Based Total Sanitation outreach affect climate-resilience programs in the village? Compared with the current program, which Plan program had the greatest influence?

Participant: The earlier five-pillar program had the greatest influence.

Interviewer: What was the strongest factor driving improvements in Community Based Total Sanitation and water? Was it government support?

Participant: There should be collaboration between the community and the government. The government must listen to people at the community level rather than provide things that they do not need.

Interviewer: As a final question about awareness, what awareness did you gain from the inclusive and climate-resilient program, particularly regarding water and sanitation?

Participant: We hope that Community Based Total Sanitation will never end. The more Community Based Total Sanitation activities are conducted, the more the community's mindset changes. Community Based Total Sanitationhas changed many behaviors, including how people manage clean water and maintain healthy living practices. Through Community Based Total Sanitation, conditions changed for residents who previously lacked clean water, and community members also became more active in seeking access to clean water.

Interviewer: Have you personally observed a change?

Participant: I am personally somewhat satisfied because clean-water services have been realized for residents who previously lacked them.

Interviewer: After participating in the activity, did your views about decision-making and gender roles change, such as the idea that certain work is only for men or only for women?

Participant: Community Based Total Sanitationchanged our awareness. We realized that women have a very large role, including caring for children, and often carry multiple responsibilities. After recognizing this, men can also take on some of those roles.

Interviewer: What household roles have you taken on since participating inCommunity Based Total Sanitation?

Participant: I now do things that I did not usually do before, such as sweeping and cooking. When my wife is busy, I can take over those tasks, including helping with washing.

Interviewer: In the past, did your wife always have to prepare your coffee?

Participant: Yes, but now I make it myself.

Interviewer: Besides the activities during the past two years, was there anything else that encouraged you to take a more active role in the household?

Participant: We previously thought that those tasks were women's roles.Community Based Total Sanitation helped us understand that household responsibilities require cooperation.

Interviewer: Before we conclude, is there anything else that you would like to share?

Participant: Before triggering and related activities were conducted, community members were accustomed to expecting assistance. After we explained the purpose through triggering, people understood the direction of the program.
