# Supplementary material for: Implementing a climate-resilient and inclusive WASH guidance in Indonesia: A mixed-methods case study
Source: PLoS One. 2026 Sep 28;21(9):e0359266. doi: 10.1371/journal.pone.0359266 (PMC13618932; doi:10.1371/journal.pone.0359266)
Supplement: Supplementary file 6 [file pone.0359266.s006.zip › S6 Appendix Transcripts/KII-02_Deidentified_English.docx]

Interviewer

Participant

Participant: [participant]

[Involved in the earlier program for several years but no longer active when CCRIW was implemented.]Community Based Total SanitationCommunity Based Total Sanitation

Interviewer: Has it not opened yet?

Participant: No, not yet. There are education and general staff positions, but the recruitment has not opened.

Interviewer: What is your full name, ma'am?

Participant: [participant]e.

Interviewer: So you have participated inCommunity Based Total Sanitation since when?

Participant: [For several years, beginning before the COVID-19 period.]

Interviewer: I will read the consent information.

My name is [interviewer], and I am a researcher from [research institution].

This study aims to identify the factors that influence climate-change responses in implementing inclusive Water, Sanitation, and Hygiene (Community Based Total Sanitation) programs in Indonesia. It will improve our understanding of the enabling factors and barriers to achieving resilient and inclusive Community Based Total Sanitationservices and to making decisions in village program planning.

Today, we are examining community experiences with access to inclusive and climate-resilient Water, Sanitation, and Hygiene (Community Based Total Sanitation), including solid-waste management, food hygiene, and wastewater management. Please keep these components in mind when answering the questions.

This discussion will use the terms extreme weather events and climate hazards interchangeably. When I use the term climate hazard, please remember the following examples. Resilience means being able to withstand climate disasters, while inclusiveness means involving everyone, including marginalized groups and people who are usually rarely involved. [Continue reminding the participant about this concept.]

This discussion will take approximately one hour and will be recorded. The audio recording will be stored securely while in use, transcribed, retained for one year, and then destroyed.

Participation in this study is entirely voluntary. You may choose not to participate, skip any question, or stop the interview at any time without consequences. Your identity will be protected because any information you provide will be kept confidential and will not be shared with anyone. Please tell us if you need special support to participate comfortably.

Thank you for participating in this discussion. It would be very helpful if you answered honestly so that we can provide better recommendations concerning program procedures. Every comment you provide will be kept confidential. We will not share your responses with anyone. Your answers will not affect whether the village receives a grant or another program in the future. This is the consent form that you need to read and sign.

Do you have any questions about this discussion?

I will begin the recording now.

Interviewer: Okay, we will continue. This interview has three main parts: your experiences and perceptions of inclusive and climate-resilientCommunity Based Total Sanitation, followed by the closing. Could you describe how long you have lived in this village and your role here, [participant]?

Participant: [participant]y name is [participant]. I have lived here since birth and am originally from this village.

Interviewer: What role have you held in the village?

Participant: I am a member of the Family Welfare Movement, or PKK. I participated in five-pillar Community Based Total Sanitation and observed community processes to help facilitate village sanitation, such as providing garbage containers and other facilities.

Interviewer: Have you participated in inclusive and climate-resilient Community Based Total Sanitation?

Participant: I have not participated in that activity.

Interviewer: So you participated in five-pillarCommunity Based Total Sanitation. Let us review the context. These images show disasters. In [study village], droughts and floods affect resilience. Previously, Community Based Total Sanitation focused on successfully implementing the five pillars under normal conditions. During the past two years, inclusive and climate-resilient Community Based Total Sanitation has examined whether all five pillars can still be met during flooding or water shortages. The concern is whether Community Based Total Sanitation services remain available during floods and dry seasons, rather than only under normal conditions.

Interviewer: In your general assessment, [participant], what are the main challenges in implementing the five Community Based Total Sanitation pillars?

Participant: Some people in this village still dispose of garbage in inappropriate places. Some also defecate in the river or in flowing water.

Interviewer: In which hamlet does that occur?

Participant: There are several hamlets. In one settlement, people still throw garbage near the river. People who live at the river mouth also commonly defecate or bathe in the river.

Interviewer: Does that still occur now?

Participant: A few people remain stubborn. Perhaps they find it easier or more comfortable to use the river.

Interviewer: What challenges affect water access?

Participant: Thank God, there are no major water-access challenges from the upper area down to the rice fields and residential areas.

Interviewer: Do people here use Community Based Total Sanitation water or wells?

Participant: We use well water, although the wells sometimes dry up. My well often has limited water. When no water comes out, I turn off the pump and wait several minutes or an hour. When I turn it on again, water usually comes out.

Interviewer: Does that occur every year or only during certain seasons?

Participant: It occurs every month and every year, as it does now.

Interviewer: I thought it occurred only during a particular season. Where do you go when your household has no water, [participant]?

Participant: I simply wait.

Interviewer: What problems affect Community Based Total Sanitationservice here?

Participant: There is no Local Water Supply Utility service in this area. Most people use wells or drilled wells. Local Water Supply Utility serves [local hamlet], including the kiosk in front.

Interviewer: Does that service experience any problems?

Participant: I have not observed any problems because the water flow appears strong.

Interviewer: What extreme weather event occurs most often here?

Participant: Flooding occurs frequently inside the settlement, especially in [local settlement]. The water enters through the alley in front of the mosque.

Interviewer: Where does the water come from?

Participant: It comes down from the mountain and can rise to thigh level.

Interviewer: How long does it usually remain?

Participant: It can remain overnight when the rainfall is intense.

Interviewer: Is there no drainage channel?

Participant: [participant]ost places have drainage channels, but gravel carried by the water settles in them. Trees have been cut down to plant corn, which contributes to the sediment.

Interviewer: Have there been efforts to clean the channels?

Participant: Not yet. People only work together to clean up after a flood.

Interviewer: To your knowledge, [participant], when flooding causes standing water...

Interviewer: ...does it become difficult to access toilets or practice the five Community Based Total Sanitationpillars during and after a flood?

Participant: During floods, some women take the opportunity to throw household garbage into the floodwater.

Interviewer: Are there serious difficulties in practicing the five Community Based Total Sanitation pillars during flooding?

Participant: There are no severe difficulties. People sometimes follow government instructions, such as directing the flow of water toward a certain place.

Interviewer: Are some toilets level with the houses or built too low, or are most toilets elevated?

Participant: Some are elevated and some are low.

Interviewer: Which toilets are elevated?

Participant: Newly built toilets are elevated because flooding occurs frequently. Households whose bathrooms often flooded renovated them.

Interviewer: Were the renovations privately funded or supported through assistance?

Participant: They were privately funded. When assistance is available, some households receive a free toilet.

Interviewer: How do you personally maintain water availability, [participant]?

Participant: In the next few days, we may need to deepen the well by several centimeters.

Interviewer: Why does the well run out of water? Has it always happened frequently, or only in recent years?

Participant: I only recently moved into this house. I previously lived farther inside the settlement, where the well was inside the house and had a large supply of water.

Interviewer: Did that house flood?

Participant: No, because it was a raised house. Water did not remain there for long because the house was on a slope and had a drainage channel that led directly to the river.

Interviewer: Thank you. To your knowledge, is there currently an Community Based Total Sanitation team?

Participant: I do not know much about the current situation. I previously participated and knew the other members, but because people became busy with other activities, the team became less active. Perhaps only government staff are now active.

Interviewer: Is there an organization that manages water?

Participant: Do you mean businesses that manage refill drinking water? There are now many refill-water businesses. Some process well water, while others manage water from mountain springs.

Interviewer: Is mountain water distributed here? Who manages it?

Participant: I think it is privately managed.

Interviewer: Is there an organization?

Participant: I do not know of an organization. The existing services appear to be independent businesses.

Interviewer: Before I arrived, had you heard about climate change or climate hazards, [participant]?

Participant: We generally understand what climate change is, but we do not know the details or how it relates to Community Based Total Sanitation

Interviewer: How quickly do residents respond after a flood? How quickly do they clean toilets and restore clean-water services?

Participant: Some respond quickly, some respond slowly, and others are indifferent.

Interviewer: Are toilets damaged?

Participant: Toilets are not damaged; the house walls are more likely to be affected.

Interviewer: We would like to understand community involvement in Community Based Total Sanitation. In either the earlier or current Community Based Total Sanitation activities, were community members involved in decision-making?

Participant: The village government usually manages the activities and sometimes asks neighborhood and hamlet leaders for assistance. Ordinary residents do not make the decisions, but they help and participate in collective work.

Interviewer: Can residents submit proposals?

Participant: During the village development-planning meeting, they can express their opinions.

Interviewer: Are those proposals addressed?

Participant: Some are addressed if they are feasible. Other ideas are acknowledged but not adopted.

Interviewer: When a disaster such as flooding occurs, who usually makes decisions?

Participant: Government officials, including the subdistrict head and village head, can make decisions. Residents whose houses are affected also take action on their own initiative.

Interviewer: Have you observed examples of collective work to improve clean-water or sanitation access?

Participant: I have not seen it for clean water inside the settlement, but I often see collective work in the rice-field area.

Interviewer: During a disaster, is there coordination among residents to distribute clean water to an affected hamlet?

Participant: No. People generally work independently or act on their own initiative.

Interviewer: Did such initiatives arise because Community Based Total Sanitation had previously been implemented?

Participant: In the past, the government mainly distributed free soap, buckets, and handwashing containers.

Interviewer: You have not participated in inclusive and climate-resilient Community Based Total Sanitation, but you may have heard people discuss inclusive STBM. Based on your experience with the earlier Community Based Total Sanitation program, how did the village government support it?

Participant: The village government was committed and supportive.

Interviewer: Did theCommunity Based Total Sanitation team or community have difficulty communicating with the village government?

Participant: No, there were no difficulties.

Interviewer: Although you were not involved in the program during the past two years, you participated in Community Based Total Sanitation. Do gender, disability, or age affect a person's participation?

Participant: Sometimes they do.

Interviewer: How?

Participant: [A community member with a disability was described as continuing to collect drinking water from the river despite advice to adopt safer practices.]

Interviewer: So that is one barrier?

Participant: Yes. It remains difficult to advise that person. However, it is not only persons with disabilities. Some community members have attended school but are also difficult to advise.

Interviewer: If they do not practice the recommendations, they are also less involved in decision-making. What prevents them from participating in or following Community Based Total Sanitation?

Participant: They say that purchased water contains chlorine, although that is not necessarily true.

Participant: They also say that river water tastes better and is free.

Interviewer: So limited understanding prevents them from participating in decision-making?

Participant: When we advise them, they agree verbally, but they do not follow the advice.

Interviewer: Is there a key person who is highly influential, respected, or powerful in relation to STBM or clean-water services in the village?

Participant: The human-resource characteristics here are very individualistic. People tend to say, "You take care of your affairs, and I will take care of mine." Therefore, no particular person is elevated or widely respected in Community Based Total Sanitationmatters.

Interviewer: Could the village head serve as a key actor?

Participant: People listen to the village head and hamlet heads, but residents can be negligent and indifferent.

Interviewer: Regarding vulnerability, does disability affect people's ability to practice the five Community Based Total Sanitation pillars and obtain water?

Participant: The couple with disabilities is married and stays in a relative's house. They had a child who required surgery, and the child was placed with relatives because both parents have disabilities. The child is cared for by the wife's family. The couple still cooks with firewood and collects water from the river. The wife sometimes urinates in her clothes.

Interviewer: What factors influence that situation?

Participant: Their mental condition and lack of money. They depend on other people's compassion and assistance from the local government.

Interviewer: Do they receive assistance?

Participant: They receive some assistance from the local government, perhaps food staples, although I do not remember the exact type.

Interviewer: Do they live together?

Participant: The husband and wife live together. They have a place to live, but it belongs to the husband's relative. Before a family member died, they sometimes stayed in a field hut while watching cattle.

Interviewer: So persons with disabilities are vulnerable in accessing these services?

Participant: First, because they do not have money, and second, because of their mental condition.

Interviewer: What steps could help the couple practice Community Based Total Sanitation? Have any supportive efforts been made?

Participant: I do not know what the government has done because I have not observed any change in their situation from the past until now.

Interviewer: What is the husband like? Is he the person I will visit later?

Participant: He has a strong personality. Even ordinary conversation with him is difficult.

Interviewer: I would like to ask about knowledge and capacity. Did older generations have knowledge about reducing flood or drought risk?

Participant: I do not think so. Perhaps the older people now have all become modern.

Interviewer: Have you read or learned from the Community Based Total Sanitation team about ways to prevent flooding?

Participant: In my opinion, one important measure is to stop illegal tree cutting. Many migrants in my settlement grow and sell corn, and their families have now settled here. Flooding did not occur in the past before people began planting corn. After corn cultivation expanded, flooding began.

Interviewer: In your opinion, does a person's capacity strongly influence the ability to minimize flood risk?

Participant: Yes.

Interviewer: How seriously do climate disasters threaten water availability and Community Based Total Sanitation services here?

Participant: The effects are very serious. They include clean-water shortages and increased mosquito populations.

Interviewer: Have you personally experienced flooding or drought that affected your daily life?

Participant: I operate a laundry business, so water shortages delay my work. Customers often want their clothes returned quickly. Work that would normally take two days can take three or four days because of the lack of water.

Interviewer: What do you do to anticipate drought?

Participant: So far, I have not taken any action. I am waiting until I have enough money to deepen the well.

Interviewer: Do you have concerns that may influence the actions you take to address the water problem?

Participant: No.

Interviewer: So you have never participated in an inclusive and climate-resilient Community Based Total Sanitationworkshop?

Participant: No, never.

Interviewer: In your opinion, did Plan's earlier program affect water access and implementation of the five pillars?

Participant: In the past, many people practiced open defecation in the fields. Now, many have built toilets. Households without toilets received toilet assistance. Many people also changed because of their own awareness, and handwashing has improved.

Interviewer: What was the strongest factor that encouraged residents to change and practice the five Community Based Total Sanitation pillars?

Participant: We often frightened people by explaining the risks. There were many cases of diarrhea associated with not washing hands and practicing open defecation, including defecating in holes and covering the waste with soil. Mothers became afraid because some people developed severe diarrhea and vomiting. Given the local character and level of understanding, people sometimes need to hear sensitive and frightening examples before they act.

Interviewer: Did you personally change after participating in Community Based Total Sanitation?

Participant: Yes. In the past, I washed dishes using water stored in a bathing container, which I sometimes cleaned only once a week. When I looked carefully, I saw mosquito larvae because the water had been stored too long. Now, I wash dishes under running water.

Interviewer: When you participated in Community Based Total Sanitation, were women and persons with disabilities involved in decision-making?

Participant: No, they were not involved.

Interviewer: Who was involved?

Participant: Local government officials and village-office staff were involved. I became involved only when village competitions were held and I was given instructions.

Interviewer: So there was no change in decision-making?

Participant: No, there was no change in decision-making.

Interviewer: Besides Plan's five-pillar program, was there anything else that increased your awareness of water and Community Based Total Sanitation conditions?

Participant: Community Based Total Sanitation itself changed me, particularly during the COVID-19 period.

Interviewer: Is there anything you would like to say aboutCommunity Based Total Sanitation in relation to flooding or drought here?

Participant: I hope Community Based Total Sanitation will contribute more and provide occasional outreach. It has been a long time since activities were held. We are very grateful for Community Based Total Sanitation because it motivates us to live healthier lives.
