# Supplementary material for: Implementing a climate-resilient and inclusive WASH guidance in Indonesia: A mixed-methods case study
Source: PLoS One. 2026 Sep 28;21(9):e0359266. doi: 10.1371/journal.pone.0359266 (PMC13618932; doi:10.1371/journal.pone.0359266)
Supplement: Supplementary file 6 [file pone.0359266.s006.zip › S6 Appendix Transcripts/KII-03_Deidentified_English.docx]

Interviewer

Participant

Interviewer: Okay, so you were initially involved in five-pillar Community Based Total Sanitation and now in climate-resilient Community Based Total Sanitation. My name is [interviewer]. This study aims to hear about your experiences with inclusive and climate-resilient Community Based Total Sanitation and your views about what the community has experienced. What is your full name?

Participant: [participant].

Interviewer: What is your current position?

Participant: Hamlet head in [local hamlet].

Interviewer: Is the village dry?

Participant: It could be described as dry, but residents can still use well water, and crops do not experience severe drought.

Interviewer: Have you served as hamlet head for a long time?

Participant: [For more than a decade.]

Interviewer: Besides serving as hamlet head, do you farm or raise livestock?

Participant: No. I do irregular work. If someone asks me to work as a laborer, I do that, and when I have an opportunity, I go fishing at sea.

Interviewer: So you have served for more than a decade under different village heads. I will read the protocol. My name is [interviewer], and I am a researcher from [research institution].

This study aims to identify the factors that influence climate-change responses in implementing inclusive Water, Sanitation, and Hygiene (Community Based Total Sanitation) programs in Indonesia. It will improve our understanding of the enabling factors and barriers to achieving resilient and inclusive Community Based Total Sanitationservices and to making decisions in village program planning.

Today, we are examining community experiences with access to inclusive and climate-resilient Water, Sanitation, and Hygiene (Community Based Total Sanitation), including solid-waste management, food hygiene, and wastewater management. Please keep these components in mind when answering the questions.

This discussion will use the terms extreme weather events and climate hazards interchangeably. When I use the term climate hazard, please remember the following examples. Resilience means being able to withstand climate disasters, while inclusiveness means involving everyone, including marginalized groups and people who are usually rarely involved. [Continue reminding the participant about this concept.]

This discussion will take approximately one hour and will be recorded. The audio recording will be stored securely while in use, transcribed, retained for one year, and then destroyed.

Participation in this study is entirely voluntary. You may choose not to participate, skip any question, or stop the interview at any time without consequences. Your identity will be protected because any information you provide will be kept confidential and will not be shared with anyone. Please tell us if you need special support to participate comfortably.

Thank you for participating in this discussion. It would be very helpful if you answered honestly so that we can provide better recommendations concerning program procedures. Every comment you provide will be kept confidential. We will not share your responses with anyone. Your answers will not affect whether the village receives a grant or another program in the future. This is the consent form that you need to read and sign.

Do you have any questions about this discussion?

I will begin the recording now.

A question was asked about how the interview results would affect the Community Based Total Sanitation team, whether the results would be shared with the community, and whether the village might quietly receive assistance.

It was confirmed that the study was not connected to assistance, but that its findings could inform future policy recommendations.

Interviewer: Before asking more detailed questions, could you explain how long you have lived in this village and describe your role?

Participant: Thank God, my family has lived in [study village] for generations. My parents and grandparents were born here, and I was also born here. Before I was entrusted with a village-government position as hamlet head, I had been active since my youth in the youth organization and other community organizations. I also served as a "invite," a trusted person who visited homes individually to invite residents to community events. That role was connected to the local youth organization and involved delivering invitations. Thank God, I continue to carry responsibility for [study village] today.

Interviewer: When did you begin participating in STBMCommunity Based Total Sanitation, and have you attended any workshop or activity organized by Plan?

Participant: I participated once as a village team member.

Interviewer: Is there also a subdistrict team?

Participant: Yes.

Interviewer: With whom did you participate?

Participant: With [local facilitator].

Interviewer: With the village head?

Participant: Yes, once. My name remains on the team list. When the district team visits, I am assigned to assist at the village level.

Interviewer: So it was a village-level activity. Did you participate in a workshop involving mapping?

Participant: I participated when monitoring and village mapping were conducted because I am part of the village government.

Interviewer: Did you participate in the transect walk?

Participant: Yes, I accompanied colleagues who came from the district and subdistrict.

Interviewer: But you did not attend the workshop itself?

Participant: My involvement was limited to those activities.

Interviewer: Do you know anyone from this village who attended the inclusive and climate-resilient STBM workshop?

Participant: I know that [a woman from the subdistrict level] and [a village official] also participated.

Interviewer: So you did not attend the workshop. What activities did you perform under inclusive and climate-resilient Community Based Total Sanitation?

Participant: When the district team visited, I helped conduct activities with the community. For example, we invited a person who was blind, women representing the gender group, and elderly people. We helped the government contact and bring those participants to the activity.

Interviewer: So you are a member of the village Community Based Total Sanitation team. What is your position?

Participant: Yes, I am a member.

Interviewer: Are you the chair?

Participant: No.

Interviewer: Who is the chair?

Participant: Perhaps the village secretary. I am only a member.

Interviewer: So the village secretary is likely the chair. During field transects and mapping, you only accompanied the team?

Participant: Yes. Sometimes I was trusted to guide the team because they did not understand the locations well.

Interviewer: As an Community Based Total Sanitation team member, do you know whether the village head or secretary attended a district-level workshop?

Participant: I do not know about activities at that level.

Interviewer: I would like to ask about your experience as someone who has long been involved in STBMCommunity Based Total Sanitation. You do not necessarily have to have attended the workshop because you have extensive experience. In your opinion, what are the main challenges in implementing the five Community Based Total Sanitation pillars and accessing water in this village?

Participant: The five-pillar challenge relates to the characteristics of village communities, which differ from urban communities. The first pillar is handwashing. After returning from a workshop, we gathered residents and provided a water container in front of each house. Thank God, the practice continued for approximately two years. However, most residents are farmers and are busy. When they return from their gardens or rice fields, some wash their hands, but others forget. For environmental cleanliness and household wastewater, STBM has produced improvement. People previously disposed of waste carelessly, but I estimate that our Community Based Total Sanitation membership efforts achieved approximately 80 percent progress.

Interviewer: So fatigue and forgetfulness are challenges. Are there challenges in accessing water?

Participant: Do you mean water for washing or drinking?

Interviewer: Both. I mean the general challenges in obtaining water for washing and drinking.

Participant: There are some challenges. The village is not entirely located on low ground. Some residents live on high ground in [local neighborhood], in an area similar to the hills of [Study Area 2]. Their wells must be approximately 20 meters deep. Among the many households in that area, only a small minority can afford to dig a well. We have therefore repeatedly sought a drilled well for them.

Interviewer: In which hamlet is that area?

Participant: [Local neighborhood] in [local hamlet]. We hope another area will receive assistance this year.

Interviewer: What extreme weather occurs here, such as flooding, drought, or landslides?

Participant: Excuse me, I need to answer the telephone.

Interviewer: That is fine. Please go and answer it.

Interviewer: We were discussing disasters and extreme weather. Does this area experience flooding or drought?

Participant: The settlement is surrounded by hills, so heavy rain causes flooding. Water sometimes reaches knee height inside the settlement, but it does not remain. It recedes within approximately two hours after the rain stops. Drought is not severe, although some land cannot be cultivated.

Interviewer: When flooding occurs, are sanitation facilities or toilets inundated or damaged?

Participant: As I said, water passes through the area but does not remain. It is gone one or two hours after the rain ends.

Interviewer: Even if flooding is brief, does it affect drainage channels or toilets?

Participant: It has an effect because the water carries a great deal of garbage, which sometimes accumulates in the alleys.

Interviewer: Are toilets damaged?

Participant: No, they are not damaged.

Interviewer: Does floodwater enter wells?

Participant: Some wells become slightly turbid, but they can still be used.

Interviewer: What type of well and toilet should residents of [study village] have so that flooding has less effect?

Participant: Most toilets here are attached to the house.

Interviewer: Are they elevated?

Participant: That depends on the elevation of the land.

Interviewer: Are any newly constructed toilets located outside the house?

Participant: Some have been constructed using village funding.

Interviewer: What type of well should be built so that it does not dry up during the dry season or become turbid during flooding?

Participant: A drilled well is best. A dug well must be covered. If its upper wall is only a few rings above the ground, soil and surface water can enter.

Interviewer: Some wells receive floodwater during the rainy season and decline during the dry season. What do residents do to address those conditions?

Participant: During drought, they temporarily obtain water from a neighbor's well.

Interviewer: So they seek a deeper well. What do they do during floods to prevent water from entering their wells?

Participant: So far, we have not received formal complaints from residents. Floodwater spreads across the area, but people do not usually submit complaints to the village government. They mainly ask how the government will address the problem. In addition to wells, one or two houses on lower ground are inundated. The water does not come from a river; it comes down from the hills. In our local language, we call it "kokar ode or ditch ."

Interviewer: What maintenance should be undertaken so that water sources, toilets, and drainage channels remain usable during floods and droughts?

Participant: For drainage channels and toilets, the village government should first divide the flow of water. There is an old large irrigation channel that belonged to the public-works agency. We have requested permission to use it, but the agency has not yet approved our request.

Interviewer: Where is the channel?

Participant: It is inside the settlement. It was formerly an irrigation channel from a dam that supplied farmland, but the land was later purchased by a Chinese company. The channel became blocked and level with the surrounding ground. We want to excavate and reuse it to divide the water flowing down from the hills. If reopened, it could substantially reduce flooding by directing the flow. Part of it has already become a road.

Interviewer: So it became a road, contributing to flooding?

Participant: Yes. The public-works agency stopped using it a long time ago.

Interviewer: If the channel were reopened, what would happen?

Participant: The area would be safe.

Interviewer: In which hamlet is it located?

Participant: It is in [local hamlet], but [local settlement] experiences the impact.

Interviewer: Is that different from the flooding in [local hamlet]?

Participant: Yes. [Local hamlet] has a small active stream, and its flooding is affected by farming activities upstream.

Interviewer: So flooding occurs in both [local hamlet] and [local hamlet], but the causes differ. In one place it results from land clearing, while in the other it results from a blocked channel. I would like to ask about water and Community Based Total Sanitationmanagement. Is there an organizational structure that manages villageCommunity Based Total Sanitation?

Participant: Yes.

Interviewer: To your knowledge, what role does theCommunity Based Total Sanitation management group perform?

Participant: Some of the people involved are also village-government officials. Therefore, village-government programs and Community Based Total Sanitation programs operate continuously together because the same people implement both.

Interviewer: So the village government and Community Based Total Sanitation team are aligned. Are roles and responsibilities also shared with other residents?

Participant: Thank God, they are shared. The village Community Based Total Sanitationteam and village government cooperate. For example, they worked together so that the village could achieve the status related to toilets, what was the term?

Interviewer: ODF?

Participant: Thank God, the village government created a special program to provide toilets to residents so the village could achieve ODF status. That is evidence of cooperation between Community Based Total Sanitation and the village government. Garbage management has also improved. Waste is collected because the Community Based Total Sanitation team urged the village government to act. Funding was then allocated through the village budget to pay collectors each month, and a 3R waste-processing site is now available.

Interviewer: So the Community Based Total Sanitation team does not collect the garbage. Community members are paid to collect it?

Participant: Yes.

Interviewer: Are women and persons with disabilities involved in Community Based Total Sanitation?

Participant: Persons with disabilities are involved.

Interviewer: Are they involved in management?

Participant: Yes.

Interviewer: What about women? Does a woman serve as a coordinator or hold another position in Community Based Total Sanitation?

Participant: Yes. [A village official] coordinates the village STBM team.

Interviewer: In your opinion, does the community understand and respond to climate change?

Participant: Thank God, they understand it.

Interviewer: How do they respond to climate hazards such as flooding?

Participant: They report what occurs to the government and to Community Based Total Sanitationstaff whom they know. If they know that a particular person is on the Community Based Total Sanitation team, they go to that person.

Interviewer: Could you describe how the community participates in Community Based Total SanitationSTBM decision-making?

Participant: From the beginning, after we attended five-pillarCommunity Based Total Sanitation training, we invited residents to form groups at the village office. Thank God, their enthusiasm was extraordinary. They spoke honestly about their practices, such as where they used to dispose of garbage. People who previously practiced open defecation in the river now have toilets. The community accepted and supported Community Based Total Sanitation, and the village government implemented its responsibilities.

Interviewer: Can community members participate in making decisions?

Participant: Yes, they can.

Interviewer: Where can their participation be observed?

Participant: Community members who participated in Community Based Total Sanitationactivities share information with neighbors who do not yet understand. They explain what healthy living practices should look like.

Interviewer: What is an example of communication or coordination betweenCommunity Based Total Sanitation and the community?

Participant: What type of example do you mean?

Interviewer: An example of addressing Community Based Total Sanitationand clean-water problems while ensuring that women and persons with disabilities benefit and do not have difficulty accessing water or toilets.

Participant: As I said, we first invite persons with disabilities and people who are difficult to reach so that we can understand their living conditions. When they cannot attend, we visit the field. We divide the village by hamlet and neighborhood, gather residents in each location, and conduct activities there. Thank God, we have observed changes among several community groups we visited.

Interviewer: You said that the Community Based Total Sanitation team works as one with the government. Does the government provide support?

Participant: Thank God, yes.

Interviewer: What is an example of government support for climate-resilient clean water?

Participant: WhenCommunity Based Total Sanitationentered the village, we gained knowledge for the next steps. Government support came from the village and district levels. For example, when we began practicing the five pillars, the government involved the community-police officer and military village officer. It installed banners in different parts of the village and organized neighborhood cleanliness competitions with prizes funded by the village government. Residents responded enthusiastically.Community Based Total Sanitation produced major changes. Before Community Based Total SanitationSTBM, the environment was disorganized, and government efforts were sometimes incomplete. Since Community Based Total SanitationSTBM, both officials and residents have become accustomed to healthy and clean living, although village officials cannot be present everywhere at all times.

Interviewer: So obtaining government support has not been a major challenge. Do capacity, gender, disability, age, or ethnic background affect participation in Community Based Total Sanitation

Participant: So far, we have not encountered serious difficulties or major barriers.

Interviewer: Does ethnic background have any effect?

Participant: The largest groups here are [local ethnic group], [another local ethnic group], and [Study Area 2] people.

Interviewer: Do differences among those groups affect participation inCommunity Based Total Sanitation?

Participant: Do you mean whether participation is restricted?

Interviewer: No. I mean whether certain groups participate differently.

Participant: There may be minor differences, as minorities anywhere may not be exactly the same as the majority. However, I have not observed a meaningful negative effect because we treat all ethnic groups equally. The current village head is especially inclusive. When he eats or becomes tired while visiting residents of another ethnic group, he is comfortable eating or resting there.

Interviewer: So no group has a dominant or unequal role in Community Based Total Sanitation?

Participant: Their roles are equal.

Interviewer: Do women or persons with disabilities experience unequal participation or unequal access to services?

Participant: No, they are treated equally.

Interviewer: Does disability affect people's ability to obtain water and inclusive Community Based Total Sanitation services?

Participant: A person with a disability should not live alone if parents or relatives are available. In most cases, water is near the house, sometimes from a well outside and sometimes from a well inside. We mainly provide practical guidance so that the person does not experience difficulty.

Interviewer: What strategies are needed so that women and persons with disabilities in [study village] can withstand climate-change impacts such as flooding and water scarcity while continuing to obtain water, use toilets, and practice during the dry season?

Participant: The government and team must continually share the knowledge gained through, provide guidance, and conduct field checks. For example, in one settlement with many [local ethnic group] residents, we sometimes visit and cooperate with the neighborhood leaders. We inspect community conditions and report residents' complaints to the government.

Interviewer: Is there knowledge passed down from grandparents about reducing disaster risk during floods or droughts?

Participant: Do you mean traditional methods used by older people?

Interviewer: Yes, methods that are still practiced today.

Participant: We have not practiced them recently.

Interviewer: But did such methods exist?

Participant: Yes, although the present differs from the past.

Interviewer: What was done in the past?

Participant: People built a "small dam." When water flowed too strongly, they constructed a barrier to divide or redirect the flow.

Interviewer: What about now?

Participant: We now live in a modern era.

Interviewer: What do you mean? A traditional method could still be used today.

Participant: Yes, but the conditions have changed. [Study village] did not experience flooding inside the settlement as it does now. Older people built a small dam near the dam to direct water into rice fields.

Interviewer: What happens now?

Participant: [Study village] is becoming more urban. Concrete paving and walls direct water into a single channel, making the flow appear larger instead of spreading across the land.

Interviewer: So increased construction and concrete paving mean that small dam structures can no longer be used, and water is directed into one channel?

Participant: Yes, the water flows in one direction.

Interviewer: Is any traditional knowledge still useful for preventing flooding or drought today?

Participant: No, because there is no longer a suitable place to apply it.

Interviewer: Is there no remaining land?

Participant: Correct. When wells dry up now, people simply dig a new well.

Interviewer: Is water available when a new well is dug?

Participant: Yes. On low ground, ordinary wells reach water at approximately seven or eight meters. In the hilly areas, wells may need to be more than ten meters deep.

Interviewer: So ordinary wells are not very deep?

Participant: They are not very deep. Drilled wells are different and may reach 30 meters for larger water needs.

Interviewer: Does the community in [study village] have current knowledge for addressing climate-change impacts, rather than only knowledge from the past?

Participant: Regarding current knowledge, [study village] is described as dry, but irrigated rice fields can still produce three crops a year.

Interviewer: Even now?

Participant: Yes, even during the dry season. Nonproductive or rainfed land generally produces only one crop per year. If farmers want a second crop, they dig a well. Regarding flooding or heat, thank God the weather has not caused all the trees to die. It is simply hot weather.

Interviewer: In your opinion, how dangerous is climate change, including flooding?

Participant: It is not very dangerous here, except in [local hamlet], where the impact comes from the mountain and is linked to human actions. Over time, it may become dangerous. For example, the entire settlement in [local hamlet] could be swept away if the structure near the bridge fails because of water flowing down from the mountain.

Interviewer: So it seriously threatens the environment, clean water, and STBMCommunity Based Total Sanitation?

Participant: Yes, it threatens the entire environment in [local hamlet], and another [local hamlet] area would also be affected.

Interviewer: Because the community does not have sufficient knowledge about what to do, are you concerned that this knowledge gap will increase the risk from climate change?

Participant: Yes, I am concerned. When the community is threatened and our knowledge is limited, the clearest response is to cooperate with the government.

Interviewer: So you are concerned?

Participant: Yes. We may need support from the district government because the problem is large-scale. Constructing gabions, for example, requires substantial funding.

Interviewer: I would like to ask about the results of climate-resilient STBM. You participated in the earlier STBM program and in climate-resilient activities. Did those activities affect you personally?

Participant: Yes. Community Based Total Sanitation began by changing our own personal lives. Neighbors who observed us gradually began adopting healthy habits as well.

Interviewer: Has water access become easier, and do you better understand what type of toilet is needed?

Participant: Yes. In the past, the river in [study village] carried a large amount of water and did not dry up even during the dry season, so many people bathed there.

Interviewer: Until approximately what year did that continue?

Participant: Perhaps until approximately a decade ago, when the village leadership changed. Women bathed in the river, people defecated there, and they collected drinking water from a place called "ai batu." The water tasted fresh. After Community Based Total Sanitation began, we conducted field monitoring and demonstrations. Only one or two people now continue those practices because they truly cannot afford bottled water. Some people still wash clothes there.

Interviewer: Does your hamlet still have difficulty obtaining water? Have any clean-water or sanitation facilities been modified so that they remain functional during floods or droughts, such as changing well designs?

Participant: Some modifications have been made. People often first dig wells during a season like this, when the water table is high, and deepen them again during the same season.

Interviewer: Does deepening the well actually reach water, or might there be no spring below it?

Participant: Water remains available deeper underground. That is what people do.

Interviewer: Are there any other methods?

Participant: No, because the methods remain traditional.

Interviewer: You said that Plan, particularly inclusive and climate-resilient Community Based Total Sanitation increased people's knowledge. Are there other programs from other organizations that address inclusive and climate-resilientCommunity Based Total Sanitation?

Participant: Do you mean programs from other institutions?

Interviewer: Yes. Are there any?

Participant: I do not think any program resembles Plan's program. The district government may issue guidance, but it is generally limited to instructions. I have not seen another program that includes practical work and direct field visits with the community.

Interviewer: Compared with climate-resilientCommunity Based Total Sanitation which other Plan activities increased the community's ability to obtain clean water, withstand disasters, and improve sanitation?

Participant: Do you mean activities other than material assistance?

Interviewer: Any type of activity. For example, was triggering conducted previously?

Participant: During the past two years?

Interviewer: Before the past two years.

Participant: Activities from Plan?

Interviewer: Yes. Besides discussions and transect walks, what else do you remember?

Participant: Those are the only activities I remember from the climate program. I do not remember any others, although I also participated whenever other mapping was conducted.

Interviewer: I would like your views about awareness and decision-making. After training under inclusive and climate-resilient Community Based Total Sanitation who makes decisions about clean-water access when a climate disaster occurs?

Participant: Not everyone experiences the effects of flooding. When action is needed, women can also express opinions and make decisions. For example, when water enters a low area, both women and men decide to block the lower gate with sacks.

Interviewer: Women and men now make decisions together. Was decision-making different before the program?

Participant: Yes, it was different.

Interviewer: Who made decisions before?

Participant: People used to say that certain tasks were not women's work or not men's work. Now, after the program, we would feel ashamed not to help, even when a task was not traditionally considered ours.

Interviewer: In the past, people simply said that a task belonged to women, but that distinction no longer applies?

Participant: For example, men previously did not want to sweep the yard.

Interviewer: What about now?

Participant: Now it is considered everyone's responsibility.

Interviewer: Did that change occur after this program?

Participant: Yes, after the program.

Interviewer: Besides sweeping the yard, what work was previously done only by women but is now also done by men, particularly inCommunity Based Total Sanitation and water provision?

Participant: In the past, people often collected water from the river. After wells were dug at their houses, men also began collecting and managing household water. Men realized that some of their previous assumptions were wrong.

Interviewer: Why did the program cause people to recognize that those assumptions were wrong?

Participant: What is the modern term for it?

Interviewer: Gender equality?

Participant: It concerns people's knowledge and capacity. Before receiving experience, knowledge, and guidance, people may not understand what is correct.

Interviewer: So gender-responsive and inclusive Community Based Total Sanitationprovided information about gender equality?

Participant: Yes, it discussed gender equality and mutual cooperation involving women. For example, collective work at the mosque previously involved only men, but women now also participate.

Interviewer: Besides this program, has any other program increased awareness of the importance of involving women, men, and persons with disabilities in clean-water and Community Based Total Sanitation matters?

Participant: I do not know whether other participants attended different training sessions. I can only speak about STBM. I also once attended another activity at a hotel, but its concept was different. Community Based Total Sanitation has had the greatest effect on the community and daily life. It even teaches handwashing in very careful detail.

Interviewer: What was the strongest reason that this activity truly changed awareness about sharing roles, including with persons with disabilities?

Interviewer: What caused awareness to change?

Participant: First, awareness itself.

Interviewer: What changed that awareness?

Participant: A sense of embarrassment after receiving knowledge. People's egos were previously high, but now they recognize that such ego is wrong. There is a local saying about men's roles, but over time that attitude was overcome by the knowledge we received.

Interviewer: So the meetings had an effect. I thought people might simply attend and then forget everything afterward.

Participant: No.

Interviewer: Was the message immediately accepted at the beginning?

Participant: Women initially said that men would never become self-aware. Thank God, we invited the same people two or three times. After they understood, we invited different participants, and the earlier participants shared the message with the wider community.

Interviewer: Is there anything you would like to share about your experience with inclusive and climate-resilient STBM, including anything that changed you or that did not fit local conditions?

Participant: First, I thank you for coming to our village and reviewing these issues. This interview helps us remember knowledge that can be forgotten when we become busy, even after attending training. Regarding the positive aspects, STBMCommunity Based Total Sanitation has been extremely valuable from the five-pillar stage through the climate-resilience stage. Change cannot happen instantly. As you mentioned, improving sanitation during floods depends on our capacity, and the government cannot solve everything immediately. Therefore, the Community Based Total Sanitation team continually guides and informs residents. Most importantly, Community Based Total Sanitationprovides knowledge and education about healthy living in [study village]. People have different views, so perfection is impossible. In the future, I hope the system continues through cooperation between local and national governments and Plan, which we understand is connected to Australia. If cooperation benefits both countries, it should continue. Material development is important, but improving village human resources is especially important. Community Based Total Sanitationshould also strengthen community knowledge and capacity, because any program becomes easier when people have strong capacity.

Interviewer: Have you personally changed, such as recognizing that household work is not only women's responsibility?

Participant: Personally, I believeCommunity Based Total Sanitation influenced many people. In Islam, the Prophet taught men to make their wives happy. STBM gave us the courage not only to practice these values privately but also to face the community and explain them to others.

Interviewer: Because we feel that we have the knowledge and capacity. Thank you very much, sir.
