# Supplementary material for: Implementing a climate-resilient and inclusive WASH guidance in Indonesia: A mixed-methods case study
Source: PLoS One. 2026 Sep 28;21(9):e0359266. doi: 10.1371/journal.pone.0359266 (PMC13618932; doi:10.1371/journal.pone.0359266)
Supplement: Supplementary file 6 [file pone.0359266.s006.zip › S6 Appendix Transcripts/KII-04_Deidentified_English.docx]

Interviewer

Participant

Interviewer: [village official], we actually only have three sessions. First, there are general questions about your activities, your experience and perceptions concerning inclusive and climate-resilient Community Based Total Sanitation, and then a closing section. Could you tell us about your daily role?

Participant: My role in Community Based Total Sanitation, you mean?

Interviewer: Your general role in the village.

Participant: Our general role in this village is, first, to administer the government properly in accordance with the mandate of the law. Second, we make efforts to improve community welfare. In particular, Community Based Total Sanitationis truly remarkable because it focuses more on triggering changes in mindsets and behavior. It is very useful because it supports the implementation of government programs under my leadership as village head.

Interviewer: May I ask how long you have lived here?

Participant: [middle-aged, lifelong local resident and village leader]

Interviewer: So you have participated in a climate-resilient Community Based Total Sanitation workshop?

Participant: Yes, I have.

Interviewer: Is there any part of the workshop that you remember, such as what you did?

Participant: We mapped locations to identify which parts or areas of [study village] were experiencing the effects of climate change as felt by the community, so that in the future we would know how to address them.

Interviewer: What was your role during the workshop?

Participant: I served as an observer at the time because the activities were carried out by groups of older people, middle-aged people, women, and others. I received the results of what they discussed.

Interviewer: What was your response after the workshop?

Participant: After the workshop, I felt that we came to understand what the effects of climate change were like. I only learned from the workshop, when people expressed all their concerns, that springs were no longer being protected, the hills were becoming bare, and this would result in flooding in the future.

Interviewer: So you learned during the workshop that these issues were related to water sources. Does that mean there is drought here?

Participant: The drought situation varies by area. About 200 hectares in this part are dry land, while the rest is wet land.

Interviewer: I will continue to the section about your experience and perceptions concerning inclusive and climate-resilient STBMCommunity Based Total Sanitation. In your opinion, what are the main challenges to the five Community Based Total Sanitation pillars in [study village]?

Participant: Based on my several years of experience, there are many ethnic groups here. There are [local ethnic group], [another local ethnic group], [another local ethnic and religious group], and a small number of [people from another local area]. [local ethnic group] people understand cleanliness. When they build a house, they make sure it has a toilet. But some [another local ethnic group] and [local ethnic group] Hindu residents build houses without toilets, and sometimes pig and cattle pens are located around the house. We have to change that mindset.

Interviewer: What about challenges in accessing water?

Participant: People here use dug wells for clean water. Only one area, [local hamlet], has brackish water because it is beside the sea. Recently, substantial funding was obtained for a clean-water system in [local hamlet]. Construction of another clean-water system in a mountainous area is also planned. We are just waiting. Only one location remains, in a place called [local settlement].

Interviewer: Is that on the coast, sir?

Participant: It is on the coast. Clean-water services have not yet reached it, and the infrastructure is also still in poor condition, with livestock and everything else around.

Interviewer: What extreme weather event occurs most frequently here?

Participant: The extreme weather event here is flooding.

Interviewer: Where does it occur?

Participant: Flooding occurs in [local hamlet]. There are three areas, [local settlement], [local settlement], and [local hamlet]. Those are the areas that are genuinely vulnerable to the effects of climate change.

Interviewer: Are they located on the coast?

Participant: They are on the coast, but also along the main road.

Interviewer: Where does the flooding come from?

Participant: The flooding comes from the mountain, from mountain runoff, because the residents' livelihood is corn farming.

Interviewer: I thought it came from an overflowing river.

Participant: No, no.

Interviewer: In [local hamlet], are the residents [local ethnic group], Hindu, or [local ethnic group]?

Participant: There are [local ethnic group] and [local ethnic group] residents, with [local ethnic group] residents forming a large proportion of the population.

Interviewer: What challenges do people in this flood-prone hamlet face in implementing Community Based Total Sanitation during floods?

Participant: This is the major challenge. On the one hand, they have limited livelihood options. Their first sources of household income are corn and livestock. Now they have returned to corn because it is promising. We cannot simply prohibit them. The steepest slopes are probably the only areas that are still empty. The other land has already been occupied because people began controlling it before I became village head. If I intervene to address the effects of climate change, I may also be attacked. That is an extraordinary constraint.

Interviewer: During a flood, is their water access disrupted? Is that a problem?

Participant: Yes, it is disrupted. We continue to support the provision of clean water. In the village, when flooding begins, we coordinate with the Regional Disaster Management Agency, the subdistrict government, and others. When a flood occurs, we prepare to deliver clean water.

Interviewer: What about facilities such as toilets and drainage channels?

Participant: Yes, but fortunately the water does not remain pooled. It usually lasts only about one or two hours. If the water suddenly rises, it also recedes quickly, so it is not too severe.

Interviewer: Does this mean that a certain type of well must be built so that it will not be damaged during a flood?

Participant: It should ideally be a borewell because most wells there are dug wells. Floodwater can enter a dug well, especially when the well wall is not high enough. With a borewell, we can raise the pipe, so it is safer.

Interviewer: So borewells are considered safe?

Participant: Yes, borewells.

Interviewer: What type of toilet would be appropriate so that it would not be affected?

Participant: That is the issue. Recently, I think we received a substantial number of toilets through the Community Based Total Sanitation program from the District Health Office.

Interviewer: When flooding occurs, are the toilets provided by the Health Office inundated, or were they built slightly higher?

Participant: Yes, that is why the toilets we built were elevated above the estimated flood level so that they would not be affected.

Interviewer: Okay, we can take this slowly, sir. People may consider water and sanitation trivial, but they are very fundamental. In general, how do people in [study village] choose or adapt their water and sanitation conditions so that they will not be affected by flooding?

Participant: From what I see, most of [study village] is not severely affected, only [local hamlet]. Most house foundations there are rather low. Fortunately, residents already have perimeter walls. We now anticipate how water, toilets, and houses can remain usable. We advise them to raise low houses or strengthen and raise the walls. Alternatively, we can use tarpaulins and wood as temporary protective measures.

Interviewer: Is there an Community Based Total Sanitation organizational structure here?

Participant: Yes. I used to be the chair when I was on the Village Consultative Body. It has now been handed over to [named local actor].

Interviewer: So there is a village Community Based Total Sanitationteam. Is there also a water-management team?

Participant: We divide water management by hamlet. There is a PAMSIMAS system, so it has its own management team. The team in [local hamlet] also has its own managers.

Interviewer: So it is organized by hamlet? Could you explain how it works?

Participant: The clean-water management structure is currently functioning. Areas of [study village] without problems do not require our intervention, but we address problems in [local hamlet]. That area previously received a complete clean-water system with water meters. Residents pay 20,000 rupiah per month to cover electricity credit, cleaning, and maintenance costs.

Interviewer: So the payment is made to the management group?

Participant: Yes, to the managers, for situations such as when the water system stops working and so forth.

Interviewer: Do only the managers conduct maintenance, or does the community also participate?

Participant: The community also participates.

Interviewer: Could you give an example?

Participant: Sometimes the collected fees are insufficient to cover the cost of damage. As users, community members are invited to a meeting. We explain that the available funds are this amount while the damage costs that amount, and then we contribute resources and work together through mutual assistance.

Interviewer: So this is done through meetings? Do women and persons with disabilities participate in the planning and management of water in [local hamlet]?

Participant: Yes, they participate. Women use water the most, so we involve them, and they are actually the ones who provide input.

Interviewer: Are any women coordinators in the water-management group?

Participant: Yes. For example, a woman may coordinate fee collection because women better understand how to communicate with other women.

Interviewer: Because [local hamlet] experiences flooding, in your assessment, how quickly do residents respond to improve their toilets and water systems so that they will not be seriously affected when flooding occurs?

Participant: In terms of response speed, they already understand. During seasons or months of very heavy rainfall, they prepare themselves for possible flooding. We experienced flooding in recent years because a substantial section of a large embankment collapsed. The river and the settlement were at the same level. We have not been able to repair the damaged section to this day. Flooding occurred only in these last two years, not in earlier years.

Interviewer: I would like to ask about governance and community involvement. You said that the community participates in decisions about water. Is any person or group dominant in decisions about water management or Community Based Total Sanitation?

Participant: Overall, there is a formally established management committee. Even if some parts no longer function, men and women generally work together.

Interviewer: When flooding occurs, who usually decides what should be done?

Participant: In that area, it is usually the neighborhood head, the youth group, and the hamlet head. They then report the matter to us.

Interviewer: In your view, neighborhood and community-unit leaders have the strongest role. Are there any customary rules or values that determine who must make decisions, or that women must act in a certain way?

Participant: No. In general, we use deliberation and consensus. Whether a decision is proposed by a man or a woman does not matter as long as there is agreement. The village government only provides direction, mediates, and listens to their opinions and conclusions. If we in the village government insist on winning or imposing our own views, that causes conflict.

Interviewer: Could you give an example of communication or coordination used to address water and Community Based Total Sanitationproblems? How do the village government and community communicate so that everyone can be involved?

Participant: First, we always provide guidance, and sometimes we call people together. During the earlier Community Based Total Sanitation process, we also traveled around using a loudspeaker mounted on a three-wheeled vehicle. We used it when the rainy season began or when flooding was possible. We responded to the findings from Plan. Before Community Based Total Sanitation entered, [study village] was known for livestock roaming everywhere. If there was a traffic accident around here, people would say it must be in [study village]. People lived together with horses and cattle before my term. After I took office and Community Based Total Sanitation began, we issued a village regulation to control livestock. This addressed cleanliness. Praise be to God, about 90 percent of livestock are now controlled. Women can also play an active role, for example by planting during the rainy season. Another effect of Community Based Total Sanitation that I felt concerns waste. Previously, waste was scattered everywhere, at bridge ends and in the river. Because this is part of Community Based Total Sanitation I worked with the Village Consultative Body to issue a village regulation on waste control and management. We also received a 3R waste-processing facility worth approximately 650 million rupiah from the Environmental Office. That was another effect of Community Based Total Sanitation and involved the participation of the entire community. Issuing the village regulation was a long process.

Interviewer: What efforts have you made as the village government to ensure that the whole community participates in climate-resilient STBMCommunity Based Total Sanitation?

Participant: As village head, I continue to rely on neighborhood and community-unit leaders and the hamlet heads to remain active in climate-resilient Community Based Total Sanitation. Through them, we learn about Community Based Total Sanitation and climate-change problems and then design what we will do. The main point is that problems can be addressed when we sit together and participate. The village government cannot work alone. The benefits are for the residents themselves. Sometimes, when an area has a problem, we hold the meeting in that location. There is now a new clean-water problem involving a communal toilet in [local hamlet].

Interviewer: [local hamlet]?

Participant: It is at the corn market area. This is a major problem. The communal toilet was built before my term, many years ago. It now genuinely creates problems. This is also related to climate conditions because it causes air pollution or odors. It worked well in the early years. Based on the Public Works and Environmental Office design, the facilities were combined in one location, and the wastewater was discharged directly into an irrigation channel.

Interviewer: The wastewater?

Participant: Yes, the wastewater. Even if it is relatively clean, there is no problem when water flows through the channel. But when the irrigation channel is dry, the whole settlement smells. In addition, many of the three-inch pipes have deteriorated and leak. We continue to involve the neighborhood and community-unit leaders and deliberate with them.

Interviewer: Does the village support the repairs?

Participant: Not in this case. The users should be responsible so that they understand. The village could pay, but it would not be appropriate for us to keep paying when they are the ones using it.

Interviewer: During your term, what forms of support has the village provided for Community Based Total Sanitation and water conditions?

Participant: I have mentioned the forms of support. First, to make the village clean, we issued a regulation concerning livestock. To free the village from waste, we addressed waste management seriously. We allocate a monthly budget for vehicle rental and the waste collector's incentive, with collection conducted twice each week. The community also pays fees.

Interviewer: Okay. That support comes from the village government. What about the district government?

Participant: Its support includes toilets, latrines, clean-water facilities, and a three-wheeled vehicle for transporting waste.

Interviewer: That is quite a lot, sir.

Participant: Yes, it is fairly substantial.

Interviewer: I would like to turn to individual factors that influenceCommunity Based Total Sanitation. Do a person's capacity, gender, age, or disability status affect participation in Community Based Total Sanitation?

Participant: They have a strong influence. As I mentioned, there are [another local ethnic group] and [local ethnic group] residents. Many people marry young. Their awareness of how to establish and manage a household is very limited. They have difficulty thinking about their own needs, let alone other people. We therefore continue to cooperate with the Religious Affairs Office regarding early marriage.

Participant: That is what creates problems for us, because of their mindset.

Interviewer: Do young people participate inCommunity Based Total Sanitation decision-making?

Participant: We continue to involve them because there is a youth organization, young people's groups, and sports communities. We sometimes involve them in the Activity Management Team for all forms of local development. We also involve them in sanitation management teams and other activities.

Interviewer: So there are no barriers to accessing water based on whether someone is male or female. Is there a key figure here who controls access to water or Community Based Total Sanitation?

Participant: A key figure? I would probably refer to the hamlet head.

Interviewer: In this location, does a person's education or disability status affect their ability to obtain services?

Participant: Which services?

Interviewer: Water and solid-waste services. Are they the same for everyone?

Participant: Yes, they are the same.

Interviewer: In your view, do women, persons with disabilities, or older people face any barriers when trying to access waste or water services?

Participant: I do not think there are any specific barriers. Services are managed within the household. An older person may have a son- or daughter-in-law or a child, and a person with a disability may have another family member who can help. To this day, many people provide assistance.

Interviewer: So there are currently no barriers to water access. To your knowledge, are people aware of climate change?

Participant: Yes, they are aware.

Interviewer: What does that awareness look like?

Participant: They understand that human actions contribute to climate change and that the effects return to us. However, livelihood pressures remain. To balance this and address climate change, we have focused intensively on several springs. Two or three years ago, the surrounding areas were cleared right up to the springs. During the last two or three years, we have tried to protect the springs. We have protected two or three sources, and we also allocated village funds for reforestation.

Interviewer: Where did that budget come from?

Participant: From the village budget. We hope that this amount will serve as a stimulus and that the district government will provide additional support.

Interviewer: Is there traditional knowledge or a traditional practice here for protecting the area and maintaining water sources?

Participant: These practices remain more active among [local ethnic group] residents. Among [local ethnic group] people, they have begun to disappear.

Interviewer: What is an example among [local ethnic group] residents?

Participant: To protect a source, they perform rituals around the spring. But if there is no rain... and if rain comes... they conduct an offering ritual, sometimes a large activity or special ceremony. Among us, the practice may be dhikr or prayer.

Interviewer: But does that no longer take place?

Participant: It still occurs occasionally, but not often. Usually people pray for rain. Sometimes they ask for heavy rain, but that can also become dangerous or cause a disaster.

Interviewer: Okay. Are there any barriers that prevent the community from addressing climate change?

Participant: When we discuss barriers, the issues include mountain runoff that causes flooding and the areas around springs that have begun to be controlled or occupied. We are trying to respond gradually. The areas around the springs had been completely cleared. Now, land within at least 50 meters of a spring must not be disturbed.

Interviewer: Who ensures compliance with that rule?

Participant: We do, together with the farmer groups. We conduct triggering activities and provide them with information and understanding.

Interviewer: In your view, how does the community respond to climate change? What is their attitude toward it?

Participant: Their attitude is that they want to find ways to protect the area. For example, when people encroach on the forest upstream, there is [local area] and another upstream village. In response, the village government communicates with both upstream villages so that we can cooperate with the communities to protect locations that could cause serious flooding. Residents respond quickly. When they see something that may increase climate-related damage, they report it promptly. That is why the river has remained safe until today.

Interviewer: I would now like to ask about implementation results. How satisfied are you with this inclusive and climate-resilient program?

Participant: I am satisfied. The benefit I feel is the change in people's mindsets concerning the effects of climate change. That is knowledge. The village government and the community have gained that knowledge. We provide information and immediately put it into practice through mapping and other activities. People say, "Oh, so this is how it works." That is the first effect. Our next task is to keep the knowledge in their minds by supporting it through programs. In an area affected by severe flooding, for example, we consider what type of toilet should be built. We need to elevate it. We ask the community how high the water rises. They show us, and we raise the toilet slightly above that level. That is how it works.

Interviewer: Is there any part of the program that you consider unsuitable for your village, such as an intervention that did not fit local needs?

Participant: During my two or three years here, there has been none because what we requested matched our needs. However, I rejected a proposed communal toilet that was going to be placed in [local hamlet]. I did not want it. I preferred to receive nothing rather than accept it, so that we would not experience the same problem as another hamlet.

Interviewer: Which agency proposed it?

Participant: It came from the Public Works sanitation program. I rejected it because the discharge would go into what we locally call the nanga, or river. The river is where people fish for their daily food, including fish and shrimp. If the wastewater entered the river, everything would be destroyed.

Interviewer: So climate-resilient Community Based Total Sanitation in [study village] has included Community Based Total Sanitationmapping?

Participant: Yes.

Interviewer: What about anCommunity Based Total Sanitation audit and gender-equality monitoring?

Participant: Those have also been done.

Interviewer: Is there anything that you think has not yet been completed?

Participant: I think everything has been done. We only need a follow-up action plan. The damaged section we discussed has already been addressed and is now safer from flooding. A substantial retaining wall was constructed. That is in [local hamlet].

Interviewer: So [local hamlet] has insufficient clean water but also experiences flooding. Is that correct?

Participant: Yes.

Interviewer: What strategy would effectively encourage climate-resilient STBM so that the program is accepted and applied?

Participant: Reforestation along riverbanks is important. Although the process is gradual, it has long-term effects because climate change cannot be addressed instantly. We have also deepened and normalized the river. Other issues are fairly ordinary. The provincial government cleans drainage channels every year, but mainly those beside the main road.

Interviewer: Regarding the need to elevate toilets, did people only understand that after participating in this program?

Participant: They had some understanding and an idea beforehand, but the program strengthened it. Previously, it was only an imagined concept, and then they understood it more clearly.

Interviewer: When the toilets were provided, were they built that way? Were there any modifications, or did you only follow the standard design?

Participant: As I explained, the modifications or innovations also included the village regulation on waste.

Interviewer: How many village regulations were there?

Participant: Two, concerning waste and livestock.

Interviewer: Has the program had a financial effect on the village because funding had to be allocated?

Participant: Yes, it has required expenditures. The largest toilet budget was allocated several years ago. A continuing issue is newly married couples. We may reach zero households without toilets, and no one practices open defecation, but then newly married couples establish households and build houses without toilets. They appear again in the data. That is why we collect data again every year.

Interviewer: Do you have any suggestions for making the program more cost-efficient so that it does not require village funding?

Participant: This program is actually our shared responsibility. Sometimes the burden falls on the village budget. At a minimum, the village or district budget must be involved.

Interviewer: Can we expect community self-help contributions?

Participant: Yes. The important thing is to communicate appropriately. It is difficult when something is not explained well. So far, there have been no problems with community contributions.

Interviewer: Plan's Community Based Total Sanitationprogram will end. Do you have a specific way to ensure that it continues?

Participant: Even when Plan is no longer present, we will continue our efforts because the effects are ultimately experienced by the community. I see Plan as a mechanism that triggers change in the community. As far as I know, it has not constructed much physical infrastructure, apart from a toilet at the health center. There has been little direct physical support for residents; the village has funded that support.

Interviewer: What support does the village need so that these activities can continue?

Participant: We still need support for household sanitation and water, particularly to decide whether household wastewater from bathing and washing should be channeled directly to the river or managed separately. This remains a problem in [local hamlet]. We cannot address it this year, perhaps next year, because the drainage pipes are clogged. Residents do not have the technical capacity to construct their own systems, and their financial capacity is also limited. The village government must intervene according to its available resources. If our resources are insufficient, we request assistance from relevant district agencies. If Plan can continue, the situation will be more secure.

Interviewer: Apart from this program, have any other programs influenced inclusive and climate-resilient Community Based Total Sanitationduring your term?

Participant: No.

Interviewer: Although you mainly observed the activities, did Plan's presence influence Community Based Total Sanitationpractices here?

Participant: It had a strong influence, which is why we hope Plan will remain.

Interviewer: Has any other organization's program influenced climate-resilient Community Based Total Sanitation?

Participant: For now, only Plan.

Interviewer: What factors are needed to create change so that Community Based Total Sanitation and water services become climate resilient?

Participant: Awareness is the main factor. However, people must also understand that forest management and control are not under the village or district government but the provincial government. Provincial officers are limited. Some areas are outside our authority, and there is no firm action against activities that contribute to climate change. For example, forest encroachment has not been addressed seriously. Therefore, we should not blame communities for encroachment that contributes to climate change when reports to the province take a long time to process. Neighboring villages also face political influences related to [local area]. For example, local decisions may become political products. Before someone is elected to a particular office, promises may be made to the community, and those promises later influence policy because the community demands that they be fulfilled.

Interviewer: So awareness is the most important factor. What type of awareness has the climate-change Community Based Total Sanitation program created?

Participant: There are two aspects. First, awareness is promoted through community deliberations and even through sermons. Second, our current rules are still very weak. We want customary regulations, or [customary regulations], defining which areas may and may not be disturbed. Farmer groups could plant trees every year. We hesitate to report people to the police because we also feel sympathy for them.

Interviewer: Has the program increased awareness about involving older people, women, persons with disabilities, and everyone else?

Participant: We already involve everyone because we have village development-planning meetings. The pattern is similar to Community Based Total Sanitation. We hold discussions in each hamlet. We also involve persons with disabilities. Because some persons with disabilities have limitations in speaking or expressing opinions, we must actively observe and respond to their needs. We also continue to involve women actively.

Interviewer: Apart from climate-resilient STBMCommunity Based Total Sanitation, have other programs influenced the involvement of women and persons with disabilities?

Participant: That involvement has already been part of our approach from the beginning.

Interviewer: In your opinion, is this a good program?

Participant: Yes, it is very good. I have followed it from the five pillars through the climate-change component. I take it very seriously because few programs genuinely touch people's hearts. From what I have observed, the triggering approach does that. Our previous village approach focused on funding interventions through the village fund. Now there is triggering and collective deliberation. The community carries out the work, and the village supports it with funding.

Interviewer: Are you confident that the community can continue it?

Participant: Yes, because otherwise we would return to zero.

Interviewer: All right, sir. Thank you.

Traditional practices are mainly carried out by [local ethnic group] residents here. They conduct a special ritual around the spring.

Regarding barriers to addressing climate change, we have begun applying a rule that an area within 50 meters of a spring must be protected. Farmer groups are responsible for ensuring this protection, and we conduct triggering activities.
