# Supplementary material for: Implementing a climate-resilient and inclusive WASH guidance in Indonesia: A mixed-methods case study
Source: PLoS One. 2026 Sep 28;21(9):e0359266. doi: 10.1371/journal.pone.0359266 (PMC13618932; doi:10.1371/journal.pone.0359266)
Supplement: Supplementary file 6 [file pone.0359266.s006.zip › S6 Appendix Transcripts/KII-05_Deidentified_English.docx]

Interviewer

Participant

Participant: I usually go to the sea to fish.

Interviewer: How do you get there?

Participant: I walk and use a fishing net.

Interviewer: What time do you usually go?

Participant: Sometimes in the morning. I usually return at noon or in the afternoon.

Interviewer: Do you go alone?

Participant: I go with my wife. You know, I do not know how to be harsh with my wife. [Laughing]

Interviewer: Okay. Does the village head often come here, or have you ever gone to the village office?

Participant: I have gone to the village office, usually to collect rice assistance.

Interviewer: Have people from the village office ever come here?

Participant: Oh, never.

Interviewer: So you are always the one who goes there?

Participant: Yes.

Interviewer: Have you ever heard about handwashing?

Participant: Oh yes, I have. We had a meeting about toilets, the condition of the yard, where the houses and alleys were located. Those were the things they asked about.

Interviewer: When was that?

Participant: Usually it was one group.

Interviewer: When was the most recent meeting?

Participant: I do not remember.

Interviewer: Was it sometime last year, when you went to identify where the alleys and other things were located?

Participant: Yes, that is right. It was last year. They asked where we disposed of garbage, where we collected water, and how long it took to return home after collecting water. Those were the kinds of things they asked. They also asked what we used to cook rice, whether we had a toilet, whether we had a well, and where we disposed of garbage.

Interviewer: Oh yes. Where is your toilet?

Participant: Inside the house.

Interviewer: Where do you dispose of your garbage?

Participant: A motorcycle collects it.

Interviewer: Where do you collect drinking water?

Participant: From the river.

Interviewer: What is this in front of the house?

Participant: Well water. We use it to wash dishes.

Interviewer: You do not drink this well water?

Participant: No.

Interviewer: It must be tiring to collect water from the river.

Participant: No, because I have a small cart.

Interviewer: Oh, I thought you could simply draw water from this well in front of the house and drink it.

Participant: No. I do not like collecting water from the well. There is spring water by the river, and we simply filter it. It tastes good.

Interviewer: So you do not boil your drinking water?

Participant: We only filter it through white cloth.

Interviewer: So you have participated inCommunity Based Total Sanitation activities?

Participant: They were held twice every six months, every six months. But I participated three times, including once in [local hamlet].

Interviewer: What did you do at the activity? Please describe it.

Participant: It was about those same things, mainly environmental conditions. People from [local hamlet] were asked where their toilets were, where they disposed of garbage, and where they obtained drinking water. Many different questions were asked. Then we drew a rectangular outline like this and marked the location of our houses and alleys. It was complicated, but we understood it little by little.

Interviewer: What did you do during the meeting?

Participant: There were no other activities. We were only asked those questions. We received an invitation to attend the meeting.

Interviewer: What did they ask you?

Participant: They asked about my toilet, where I obtained water, and whether I helped my wife. I said that I always helped her. That was all they asked.

Interviewer: Since when have you lived in this village?

Participant: For a very long time, since I was a child.

Interviewer: So you were born here? What are your and your wife's daily activities?

Participant: We do not have much to do. We usually go to the hills to collect firewood or to the sea to catch fish. Those are our activities. We would like to farm, but we do not have rice fields.

Interviewer: What types of events usually occur here? Flooding?

Participant: It floods here, ma'am. The water enters the house.

Interviewer: Where does the floodwater come from?

Participant: It comes from rainwater. The water in front of the house cannot drain because the channel is blocked, so it flows back into the house. If we do not close the front gate properly, water enters.

Interviewer: Does it also enter this well?

Participant: It does not enter the well, but it enters the house because the well has a high foundation.

Interviewer: Has this well ever run out of water?

Participant: Never. It always has water. The drilled wells are the ones that often dry up.

Interviewer: How long does water usually remain inside the house?

Participant: There is an opening on the side, so we push the water toward the front so that it can flow out. It is truly exhausting.

Interviewer: For example, does water enter your toilet?

Participant: No, water does not enter it.

Interviewer: Why does water not enter it?

Participant: The previous owner of this house built the toilet on higher ground.

Interviewer: So your drinking water comes only from the river?

Participant: Yes, from the river.

Interviewer: To your knowledge, is there an Community Based Total Sanitation group here?

Participant: Yes. Basically, it includes everyone who was invited. Many people here participated, including people whose houses are near the field. There were quite a lot of people, but I do not remember all of them because everyone dispersed when they went home, so we did not know where they went.

Interviewer: Have you ever heard about climate change or climate hazards?

Participant: Never.

Interviewer: So you have only heard about Community Based Total Sanitation?

Participant: I have heard about it.

Interviewer: In the future, when you hear the term climate hazard or climate disaster, flooding is one example.

Participant: Oh, I see. Here, people only call it flooding. They do not use that other term.

Interviewer: Besides your house, where else does flooding often occur?

Participant: Usually near the river and sometimes inside the settlement.

Interviewer: When water enters the house, what do you do?

Participant: We clean everything up.

Interviewer: How long does the water usually remain?

Participant: It can remain overnight when the rain is intense. We then sleep outside on a "pantar" [a raised sleeping platform]. After the water recedes, we clean the tile floor and remove all the mud and garbage. It is very exhausting.

Interviewer: During the meeting, did you ever propose anything?

Participant: Never.

Interviewer: What did you do, then? Did you only sit and listen?

Participant: They only asked those questions about the environment, toilets, our work, and how much we earned when we worked as construction helpers.

Interviewer: When your house floods, does anyone else help you clean it? Has anyone from the government ever come to inspect your situation?

Participant: We clean it ourselves. We have told the village office about this condition and asked them to raise the foundation of the drainage channel slightly. People at the village office said that they would consider it, but nothing has been done up to today. Therefore, we bear the risk ourselves.

Interviewer: How many houses are affected?

Participant: Many houses are affected, particularly those with low foundations. They are affected almost every year. However, when the rain is very heavy, the water does not enter, whereas lighter rain can cause water to accumulate.

Interviewer: So you have informed the government about the condition and asked them to prevent future flooding?

Participant: That is what we conveyed. Whatever happens elsewhere on the road or at other people's houses is different, but the problem is here. If the foundation is raised, water will not enter.

Interviewer: So you do not have difficulty accessing the toilet, but you still collect drinking water from the river. To your knowledge, how many people still collect drinking water from the river?

Participant: Many people, both young and old, collect water there.

Interviewer: Do you ever buy bottled water? Why or why not?

Participant: No, because we do not have money to buy it. Therefore, we collect spring water by the river because it is free. If we had to keep buying water, it would be very difficult.

Interviewer: Can you still cook during a flood?

Participant: Yes, because the cooking area is elevated. This house is still connected to the previous owner, so the electricity service was disconnected by National Elictricity Company.

Interviewer: When you were a child, did you ever hear about ways to prevent flooding?

Participant: I do not know about that. Perhaps the older people knew.

Interviewer: Is this flooding dangerous?

Participant: It is not very dangerous here. We can remain calm. Flooding at the river is dangerous because a lot of timber is carried downstream. Here, it is only water.

Interviewer: So you can still obtain drinking water even when the settlement floods?

Participant: Yes. But if the river floods, we prepare a supply of water in advance. We no longer collect water from the river because we have already stored it in water containers and buckets, so the problem is resolved.

Interviewer: Please describe the activity held at the village office.

Participant: It involved the same things that I described earlier.

Interviewer: What about the division of work between you and your wife?

Participant: We always work together. Sometimes, when she is away, I wash the dishes and cook rice.

Interviewer: Have you always done that, or did you begin recently?

Participant: I have done it since the beginning of our marriage. When people ask why I wash dishes, I say, "That is fine. Who else will help my wife?" If other people do not want to help their wives, that is their decision.

Interviewer: Could you describe anything else that you learned during the meeting?

Participant: They said that they wanted to build toilets, but I already had a toilet.

Participant: Then the discussion shifted to other matters, such as where we collected firewood in the hills, where we obtained water, and how long it took us to walk there. That was all.

Interviewer: Do you also work as an agricultural laborer?

Participant: Yes. I sometimes help sow seeds and earn 100,000 IDR per day. I go whenever someone offers me work. If I work for two days, I earn 200,000 IDR

Interviewer: How do you obtain rice?

Participant: We receive rice from the village office, although it takes a long time to arrive.
