# Supplementary material for: Implementing a climate-resilient and inclusive WASH guidance in Indonesia: A mixed-methods case study
Source: PLoS One. 2026 Sep 28;21(9):e0359266. doi: 10.1371/journal.pone.0359266 (PMC13618932; doi:10.1371/journal.pone.0359266)
Supplement: Supplementary file 6 [file pone.0359266.s006.zip › S6 Appendix Transcripts/KII-06_Deidentified_English.docx]

Interviewer

Participant

Additional participant

Interviewer: (...) Okay, [participant], we can begin. As I explained earlier, you understand the purpose of this research, and you have signed the consent form, so I understand that you are willing to be interviewed. Let us go directly to the first question about water supply and sanitation in [study urban village ]. In your view, how does the water-supply system work, what are its challenges, and how is it related to sanitation?

Participant: Thank you. Regarding water availability in [study urban village ], I think it is now quite sufficient, much more sufficient than it used to be. In addition to water supplied by the municipal water utility, there are also several springs in [study urban village ]. In the last few years, however, frequent illegal logging has led to a rotational water-distribution system in [study urban village ]. Sometimes the water flow is good and sometimes it is not. Regarding sanitation, the community now appears to understand how to use household wastewater. Mothers, including my mother at home, sometimes pour dishwashing water onto plants behind the house. For example, my mother grows vegetables, so rather than wasting the water, she pours it on the plants. In addition, many residents raise livestock. Study location people commonly raise pigs, so food scraps can also be added to pig feed.

Interviewer: Excellent. Your response to the first question has already answered several other questions. Thank you very much. We can skip the remaining questions about water and sanitation because you have already explained them in detail. The next topic is extreme weather. What types of extreme weather have occurred frequently in [study urban village ], either recently or in the past?

Participant: Thank you. Based on my personal experience with extreme weather, many residents of [study urban village ] earn a living by collecting wood (NOISE). As a result, there is a great deal of illegal logging (laughing). This may not only involve residents of [study

urban village ], but people more generally. We can now see that weather patterns are unpredictable. The dry season used to run from approximately April to October, but now it is unpredictable. That is what climate change means, conditions no longer follow the expected pattern (NOISE).

Interviewer: Thank you very much, [participant] (laughing). So the effect occurs more often because trees are being cut down when people collect wood. In the past, people collected only the wood they genuinely needed. Now they even cut trees that should protect springs. What usually results from that tree cutting? Landslides, flooding, or water shortages?

Participant: Water shortages are more common (???). Flooding and landslides are rare.

Interviewer: You said that reduced water discharge occurs most often. Is that an effect of drought?

Participant: Yes. It is one effect of drought and illegal logging.

Interviewer: So drought reduces the water discharge, as does tree cutting. Is there a special structural design for protecting springs? For example, does a storage reservoir need a specific design? Or, to your knowledge, how should it be designed to withstand floods or landslides? What about the septic tank at your house?

Participant: (???) I do not know about the water-storage reservoir in the urban village . At my house, however, we have a septic tank. It can also withstand conditions such as the current rainy season.

Interviewer: In your neighborhood, have you ever experienced a septic tank collapsing during the rainy season?

Participant: So far, that has not occurred in our neighborhood.

Interviewer: (...) You said that drought occurs frequently. How do people in your neighborhood respond when it happens (???)?

Participant: Thank you. Some community members address it by replanting trees in their own gardens. The kelurahan also sometimes distributes tree seedlings provided by government agencies to each neighborhood for planting. [a household member held a local leadership role]

Interviewer: [Question about the household member's local leadership role omitted]

Participant: [a household member held a local leadership role]

Interviewer: Oh, we went to [local neighborhood] earlier.

Participant: Because I am often called there, I am accustomed to it (laughing) (...).

Interviewer: You are already very familiar with Community Based Total Sanitation and reforestation (???). If there is a problem such as interrupted water service, a leaking pipe, or another damaged or collapsed facility related to water or sanitation, where do you report it?

Participant: If there is damage, we usually report it directly to the water utility (???). Although [study urban village ] has several water sources, part of the community still uses water from the utility (???).

Interviewer: What are the operational maintenance practices for the water system? For example, how do people prevent pipes from being damaged, or what happens when a pipe has already been damaged without intervention from the water utility?

Participant: To be honest, I do not know. But when damage occurs, people can report it (???), and the system is usually checked. When a problem occurs, residents contact an available telephone number, and an officer can come and inspect it.

Interviewer: Is the officer from the water utility or the village?

Participant: From the water utility.

Interviewer: So one specific maintenance activity is reforestation, as you mentioned. For daily operations, if something is damaged, residents call the water utility's contact numbers, and there are designated employees. Around what year were the trees planted?

Participant: Regarding the tree planting, I do not know about the other activities because I happened to (??? laughing). It was last year.

Interviewer: So you have not yet seen or learned whether it is effective because it is still new?

Participant: Yes, it was last year, and there was another activity this year, in March.

Interviewer: In March as well? Was it also organized by the Forestry Office?

Participant: I do not know which agency provided them (???).

Interviewer: The next topic is water and sanitation management. Have you ever been taught about the fiveCommunity Based Total Sanitation pillars?

Participant: Yes, I have.

Interviewer: What was your role or responsibility at that time?

Participant: First, after attending the training, I shared what I had learned with my family and neighbors. I explained the benefits of STBM. The first point was that people should not defecate indiscriminately (???). At present, open defecation no longer occurs because almost every house has a toilet. In the past, people still used the old system (???). The second point was handwashing. Many people still do not use soap and only wash with water. Regarding waste management, as I mentioned, wastewater can be used to water plants, and leftover food can be used as pig feed (laughing), because many people happen to raise pigs (laughing).

Interviewer: Okay (...). Were you a participant at the time?

Participant: Yes, we were participants.

Interviewer: After the training, you said you shared the information with your family and neighbors. Was there another outreach activity in which you went from house to house with a team?

Participant: No, there was no such activity, except during the training.

Interviewer: So was the training held only at the urban village office, or did you also visit houses?

Participant: It was only at the urban village office.

Interviewer: So when you taught your neighbors about handwashing, that was your own initiative?

Participant: Yes (...).

Interviewer: Among those who attended that day, were most participants women, or were persons with disabilities also invited?

Participant: Many men and women were invited and attended that day. No persons with disabilities attended, but there were pregnant women.

Interviewer: Among the women and men, who spoke or made suggestions most frequently?

Participant: Women spoke more that day because the issues occur in women's daily sphere, especially matters involving wastewater. They said that when washing, the same water cannot be used for everything and that two containers or changes of water are needed (???).

Interviewer: We are now facing climate hazards. As you said, the weather is unpredictable, sometimes dry and sometimes rainy. How does the community respond to current climate hazards? You may first give your own opinion. Have you also heard neighbors discuss the weather?

Participant: In my personal opinion, these conditions are partly caused by the community's own mistakes, particularly failure to protect the environment and trees. More houses are now being built because the population is growing (???), so housing development is also increasing. There are more young people now, and the number of births is also increasing. What can we do? It has already happened (???). Most community members and neighbors complain about the changing weather. Many people are now becoming ill with influenza, coughs, and fever. There are many illnesses.

Interviewer: What about skin irritation?

Participant: Yes, skin irritation as well. (NOISE) There are current issues concerning [local area] (NOISE).

Interviewer: Okay, those are the general effects of climate change. What are the effects of climate change on water access and sanitation management?

Well, drought has already occurred, correct? Its relationship with water access and sanitation has already been explained at the beginning. You do not need to answer again.

(...) In your view, how quickly and strongly did the Community Based Total Sanitationteam respond? You were part of the Community Based Total Sanitation team, correct? How quickly did the team respond to climate change? Now that drought has occurred, what should be done? Was there any further outreach?

Participant: Thank you. I have not yet felt any follow-up from that activity. We attended the training, but afterward there was nothing else, at least not to this day.

Interviewer: There has been no follow-up. Do you know whether there was a particular reason why they conducted the training there?

Participant: I do not know because we were only participants (NOISE).

Interviewer: The next topic is community involvement in decisions about water and sanitation. Could you explain how the community participates in decisions concerning water and sanitation? When discussions about water and sanitation take place at the kelurahan office, who is invited? Based on your experience, or at least what you have heard, who is invited?

Participant: I do not know exactly who is invited to discussions about water and sanitation. Usually, however, the kelurahan government invites neighborhood heads, customary leaders, and others (???) (NOISE).

Interviewer: When they are invited and make proposals, have those proposals produced changes, as far as you know?

Participant: I do not know. The community is very large, so I am not familiar with what happened in those cases.

Interviewer: At the urban village meeting you attended, who most often made decisions?

Participant: I think everyone made decisions, both women and men. They had to decide together (???).

Interviewer: You said that members of marginalized groups were not present at the meeting you attended?

Participant: Correct.

Interviewer: What about your father's experience? Have you ever heard that persons with disabilities were also invited?

Participant: Yes, they were invited that day, but they could not attend because they had another obstacle.

Interviewer: So they were invited. In your opinion, what would be a good strategy to make the process inclusive so everyone can make decisions and proposals, including members of marginalized groups, when decisions about water and sanitation are made at the urban village office or [traditional meeting place] (???)?

Participant: Honestly, I do not know, but in my view everyone should be present, including marginalized people (NOISE), so they can be asked what their needs are. Even if they cannot speak, a family member can help communicate what they want to convey. Healthy people can speak for themselves (???), but some persons with disabilities may not be able to express their needs directly (NOISE).

Interviewer: Thank you. I am pleased to hear that (laughing). It is rare for people discussing strategy to show such sensitivity to marginalized groups.

Interviewer: The next question is whether there is government support to ensure that you can continue accessing Community Based Total Sanitation services during (???). For example, when drought occurs (NOISE).

Let us continue briefly. You may also have a drink. How are you, [interviewer/participant]? Okay, it will not take much longer. We have about half an hour remaining.

Interviewer: As I was saying, when drought occurs or water service stops, is there support from a government institution, such as tanker-water deliveries from the utility, or any other form of support?

Participant: Thank God, I think water is still sufficient in our neighborhood even though it is distributed on a rotating schedule. So far, we have not received tanker-water assistance. We also happen to have a spring (NOISE) (???).

Interviewer: Could you describe an experience of collaboration between the community and government? For example, when you complained about interrupted water service, were you able to help communicate community concerns successfully? This may be your experience or your father's, but it should relate to water and sanitation.

Participant: As I said, when there is a complaint, we contact the water utility. If the response is delayed, an employee of the utility can assist (???). Someone who works there can be asked to inspect or repair the problem.

Interviewer: And the main point is that the problem was successfully addressed and repaired. (...)

Interviewer: The next topic concerns power and capacity. What characteristics or conditions prevent community members from participating in decision-making? For example, what might keep them from expressing their views (NOISE)?

Participant: Thank you. I do not really know. Some people may be afraid to express their opinions (NOISE). In general, some people are still hesitant or afraid.

Interviewer: (NOISE) What is the nature of that fear? Do they want to express their objectives but feel unable to do so? What is it like?

Participant: It is not that they are afraid no one will listen. They are afraid or nervous about speaking (NOISE). Older people sometimes struggle to speak fluently in Indonesian.

Participant: So far, however, conditions in [study urban village ] are good. Regarding water and cleanliness, I think the situation is already quite good. Every household has a toilet, and the government sometimes provides assistance (???).

Interviewer: How are decisions usually made in your neighborhood, and who makes them (NOISE)? For example, if there is a disaster or drought, does your community have a practice of preparing certain things before the drought occurs in order to prevent problems?

Participant: I do not think there is such a practice. We do not know whether it will be dry or rainy now (laughing), so there is no specific preparation. I do not know about other people, but in my household and among my neighbors there is no special preparation for the rainy or dry season (laughing). We simply adapt to climate change (laughing). The greatest problem is for people who have gardens or crops. During the dry season, they have insufficient water for irrigation.

Interviewer: What is the main livelihood?

Participant: Farming.

Interviewer: Vegetable farming?

Participant: There are many types of farming, including vegetables (laughing). There are many crops (NOISE). Have you never been there?

Interviewer: The area is extensive (NOISE).

Participant: So you know A?

Additional participant: Yes, we were in the same cohort (NOISE).

Interviewer: I thought he was your spiritual leader, Kraeng?

Additional participant: Yes (laughing), I often go there (NOISE).

Interviewer: (NOISE) Could you explain specifically how Community Based Total Sanitation is managed in the neighborhood? To your knowledge, how do people manage it (NOISE)?

Participant: In our area, marginalized people are generally accepted well by the community. Regarding defecation and sanitation, there is one older man there who is ill (NOISE). Because he has been ill for a long time, cleanliness can be difficult, but his family continues to care for him and maintain hygiene. Another person had a stroke. During theCommunity Based Total Sanitation outreach, we learned that a handrail should be installed for entering the bathroom and reaching the toilet, so the person has something to hold (NOISE). There was an activity concerning that recently (NOISE).

Interviewer: So it has already been implemented?

Participant: They also said that lighting must be provided.

Interviewer: Has there been a change?

Participant: I do not know the current condition. When we visited previously, it was fairly good as a preventive measure.

Interviewer: Are there social norms or cultural practices that influence this? For example, is there a cultural belief that marginalized groups should not be involved? Does such a practice still exist?

Participant: I do not know much about whether marginalized people are included in decision-making. From what I have seen, they are rarely involved directly. They may be present or represented, but I have not seen much beyond that (???).

Interviewer: In your opinion, how extensive is community knowledge about climate disasters, particularly the effects of climate change onCommunity Based Total Sanitation? You have explained your own understanding. What about the community's knowledge?

Participant: Honestly, many community members do not know the term Community Based Total Sanitation, even though they certainly practice some of its principles in daily life. People generally do not know what Community Based Total Sanitation is unless they have participated in an activity. I only learned the term that day because of the activity (NOISE). Regarding understanding, community members notice changes themselves (NOISE).

Interviewer: Do people discuss how drought affects their water supply and household hygiene?

Participant: I do not know. Personally, I work and rarely meet with my family and neighbors, let alone other community members, so I do not know.

Interviewer: Is there local wisdom or a local tradition that influences adaptation to climate hazards? For example, to prevent landslides or floods, one form of local wisdom is the [local water-source protection tradition] tradition, which includes planting trees around springs to reduce disaster risk. Is there any special local tradition used by the community to anticipate climate hazards?

Participant: I do not think there is.

Interviewer: Do you know of any preventive tradition? Have you heard the term [local water-source protection tradition]?

Participant: I have heard the term [local water-source protection tradition], but I do not know what it involves (laughing).

Interviewer: Understood (NOISE). Psychologically, how does the community view climate hazards? Do people discuss drought?

Participant: They discuss and complain about drought. Sometimes they complain of illness, prolonged influenza, and coughing. One day may be dry and the next rainy. It also affects livelihoods (NOISE).

Interviewer: Based on what people say, would you rate the danger as severe, moderate, or something else?

Participant: Moderate (laughing).

Interviewer: So it is moderate. People are not extremely afraid (laughing)?

Participant: Correct. Perhaps they have already adapted to the climate (NOISE).

Interviewer: (...) So there have been no events in which heavy rain, flooding, or landslides caused septic tanks to collapse or toilets to overflow?

Participant: No, not so far. The main problem is the drainage channel. During heavy rain, the channel becomes full of waste, and the water flows onto the main road.

Interviewer: When that happens, who cleans it?

Participant: The residents themselves, particularly those whose houses are nearby (NOISE).

Interviewer: Through community self-help (laughing). Are there also communal work or neighborhood cleanup activities?

Participant: Yes. The urban village government usually announces a cleanup activity, such as a clean Saturday. If people are busy, they at least clean their own surroundings.

Interviewer: Regarding drought, how do people usually prepare? Do they keep water-storage containers or use another measure?

Participant: In our neighborhood, we do not use a special storage tank for drought. If piped water does not arrive, there is still another source (???). I do not know about residents near the stadium area of the urban village .

Interviewer: At home, how do you store water? Do you use jerry cans or a large tank?

Participant: At home, we usually use (??? NOISE).

Interviewer: There has never been a situation in which you had to go somewhere to draw water?

Participant: No, except when there is absolutely no water. Fortunately, there is [local spring] near the house.

Interviewer: Has there been a water shortage during the last year?

Participant: Yes. It occurred before water from the utility had reached the house.

Interviewer: How far is the spring?

Participant: Approximately 50 meters (NOISE). The community has used that spring for a long time.

Interviewer: Is it still used by the water utility or by nearby residents?

Participant: By nearby residents (NOISE).

Interviewer: (...) During the recent activity, were you taught about inclusive and climate-resilient Community Based Total Sanitation, or only Community Based Total Sanitation in general?

Participant: Yes, climate-resilient STBM was included, but I have forgotten much of it (laughing).

Interviewer: (???) (NOISE) Have you experienced inclusive access to Community Based Total Sanitation, meaning that facilities can be used by everyone, including persons with disabilities? For example, have you seen whether public facilities in [study urban village ], such as the kelurahan office, have inclusive toilets?

Participant: Yes, the toilet at the urban village office is adequate.

Interviewer: Does it have handrails and a sufficiently wide access route?

Participant: I only vaguely remember the new urban village office. The old office had not yet officially become the urban village office. It was simple and did not have handrails, but it was fairly adequate.

Interviewer: Could persons with disabilities access it?

Participant: Yes.

Interviewer: So you do not know the condition of the current facility?

Participant: I know the facility, but I have not examined details such as whether it has handrails (NOISE).

Interviewer: As far as I know, we visited it earlier. In my view, it is already inclusive. It has handrails, and although it has a squat toilet rather than a seated toilet, the fixtures are within reach. The remaining problem is the entrance, because there is no ramp for a wheelchair (NOISE). The toilet itself is accessible. (...) Apart from inclusive and climate-resilient Community Based Total Sanitation, has another Plan program influenced decision-making dynamics in the community?

Participant: I do not think so (NOISE). I only attended the training and was not involved in other activities.

Interviewer: Are Community Based Total Sanitation activities the only Plan programs you know?

Participant: Yes, only .

Interviewer: Did anCommunity Based Total Sanitation team ever visit your house from door to door?

Participant: No.

Interviewer: So if you had not attended the activity at the urban village office, you would not know what Community Based Total Sanitation is (laughing)?

Participant: Correct, hhhhh.

(NOISE)

Interviewer: Those are the questions we wanted to ask. One of your earlier responses already answered several questions. This interview has been useful for expanding our knowledge. Your responses are impressive for a young person who attended only one activity. You have provided several important points that we can summarize. Thank you very much for your time amid your busy schedule. All your responses will be used as material for analysis (???).
