# Supplementary material for: Implementing a climate-resilient and inclusive WASH guidance in Indonesia: A mixed-methods case study
Source: PLoS One. 2026 Sep 28;21(9):e0359266. doi: 10.1371/journal.pone.0359266 (PMC13618932; doi:10.1371/journal.pone.0359266)
Supplement: Supplementary file 6 [file pone.0359266.s006.zip › S6 Appendix Transcripts/KII-07_Deidentified_English.docx]

Interviewer

Participant

Interviewer: Okay, good morning, I, um...

Participant: Good morning.

Interviewer: As I explained earlier, you understand the research process and its purpose, so we can begin directly with the questions.

Interviewer: First, I would like to know about water availability and how it relates to cleanliness in your neighborhood. You happen to be a neighborhood head. How has the water-supply system worked, and how has it affected sanitation or environmental cleanliness in your area, sir?

Participant: First, during the dry season we prepare water drums because the water does not flow. Sometimes it flows, but not as abundantly from the spring as it did in the past.

Interviewer: How does the system work now? Let us compare conditions five years ago with conditions today.

Participant: Five years ago, the water was very, what is the word...

Interviewer: Abundant.

Participant: It was abundant, so even when we prepared drums, we did not need to fill all of them. Now there is less water, so we must always store it, and distribution is even scheduled by rotation.

Interviewer: Oh, a rotational system.

Participant: If the water arrives at night, we must wake up at night to collect it. That was last year. It is still distributed by rotation now, but at two-day intervals.

Interviewer: So it flows once every two days, from morning until night?

Participant: For example, our water did not flow today. It may flow tomorrow or the day after, or perhaps in the afternoon, because that is how the water utility schedules it.

Interviewer: You said water was abundant five years ago but is now distributed on a rotational schedule. Do you know the reason and the challenges behind this change?

Participant: In my view, the dry season has become too long, so the water discharge has decreased.

Interviewer: Did the water utility or another party provide that information?

Participant: That is my own analysis because the water becomes very abundant when it rains.

Interviewer: Mhm.

Participant: That is my conclusion. Perhaps the drought is too prolonged. Interviewer: But the water utility has never informed you that this is why it uses rotational distribution?

Participant: No, it has never explained that. When we go to pay the bill, we always complain that the water is not flowing.

Interviewer: Yet the monthly charge continues and has even increased, correct?

Participant: They always answer that the water discharge has decreased.

Interviewer: If the water is insufficient, why do they not explain the reason?

Participant: Yes, so it is only my own conclusion.

Interviewer: Okay. The next topic is extreme weather. What types of extreme weather frequently occur around here? I can show you the pictures. Do floods or landslides occur here? One moment.

Interviewer: Examples of extreme weather include landslides, sea-level rise, which does not occur here because this is a mountainous area, drought, or leng bail usang, meaning excessive rainfall that causes landslides or floods.

Participant: I will choose two answers.

Interviewer: That is fine. Which two occur most frequently here?

Participant: Drought occurs frequently. Our plants are automatically affected. For example, cassava leaves die, so we lack vegetables.

Interviewer: What form of extreme weather has occurred most often, drought, landslides, or floods?

Participant: Drought occurs frequently. Flooding does not. However, during the rainy season, especially during prolonged rain, excessive runoff becomes full and causes water to pool around this house because it is on low ground. I proposed at the kelurahan office that a surrounding drainage channel be constructed along the road from [local neighborhood] to [local neighborhood]. That would help address the runoff. Dirt and waste from [local neighborhood] accumulate here.

Interviewer: Are there more houses farther downhill?

Additional participant: Yes, including the house of [a former district official]District . Waste accumulates there.

Participant: Regarding cleanliness, the urban village does organize activities, and we at the neighborhood level handle them. Our basic church community group also conducts joint environmental cleanup activities. However, not all residents participate. Some do and some do not, and we cannot order them around like children.

Interviewer: You said that extreme weather sometimes causes what you described as a landslide. How does that affect you and the surrounding area?

Participant: We do not feel comfortable here.

Interviewer: As you said, waste from the higher area accumulates here.

Participant: It creates an emotional burden because it would not be appropriate for us to go and become angry with the neighboring neighborhood, even though its residents are not conscious about cleanliness. Rain also affects utility water. Much more water comes out, but it is turbid.

Interviewer: So the two problems here are drought and landslides or runoff?

Interviewer: For the drought and landslide conditions you described, are there special structural designs, such as a particular type of water reservoir or protection at the spring, so that the infrastructure can withstand landslides, floods, or the dry season?

Participant: During the rainy season, as far as I know, although I have never visited the spring, I assume the reservoir may not be covered because the water becomes very dirty when it rains. That is only my own conclusion.

Interviewer: You did not receive that information from anyone?

Participant: No.

Interviewer: Is there another design that would make it resistant to extreme weather?

Participant: I do not understand that. We simply face each season as it comes.

Interviewer: You said that you raised the runoff or landslide problem during the urban village development-planning meeting, but it has not been implemented. Therefore, you remain uncomfortable during the rainy season because waste from uphill accumulates here and you fear runoff or landslides because your house is on low ground.

Participant: For residents' safety, I also proposed improvements to this road at the urban village office. I requested a footpath because [long-term resident and local neighborhood leader], and no footpath program has ever been implemented specifically in our neighborhood. At this year's planning meeting, I requested a footpath for residents' safety, particularly because pregnant women must walk on a slippery path.

Interviewer: I am pleased that you speak about and care for vulnerable groups such as pregnant women. We will also become old someday. It was extremely difficult for us to walk down from above.

Additional information:

Participant: "I always care about vulnerable groups, especially pregnant women and children." Under the current program, pregnant women must be reported to the health center and must declare when they are economically disadvantaged. This ensures that all administrative arrangements are completed at the health center so they face no barriers when giving birth.

Interviewer: Do you help report pregnant residents, or do residents usually report that they are pregnant and request a low-income certificate from you?

Participant: The urban village instructed us to report them. We ask pregnant women to obtain a certificate stating that they are economically disadvantaged so that they will not have to manage additional documents when they give birth. I have done that before.

Interviewer: Understood. Thank you.

Interviewer: You have described landslides or runoff and drought here. Could you explain how people in [participant]'s neighborhood address these challenges?

Participant: As I said, we could address them together through communal work, but residents do not always think the way I do.

Interviewer: Have you conducted communal work during the last year? How often, and when was the most recent activity?

Participant: We are actually supposed to conduct communal work once a month.

Interviewer: Mhm.

Participant: This year, we have done it only two or three times. We may conduct another activity in August in preparation for Independence Day.

Interviewer: Regarding drought, you said people have many storage containers. In the past, when water was abundant, people often wasted wastewater. Have you seen people reuse washing water to water their yards or for a similar purpose?

Participant: Not yet. Every household understands that it must prepare many drums for the dry season. We previously discussed drums and water at the old urban village office, when the office was still in a rented building, together with health personnel.

Interviewer: Yes.

Participant: At the time, I complained about the rotational water schedule. They told us to prepare many drums. I said that we had already prepared them, but collecting water required cooperation among husbands, wives, and children.

Interviewer: Did they teach you that?

Participant: Yes. I said that we often cooperate to collect water, but when it arrives at midnight, we husbands become like night security guards.

Interviewer: Hahahahahaha.

Participant: I said that we could not possibly wake our wives and children.

Interviewer: What an excellent husband.

Participant: The men must do it. Excuse me, but every household should prepare many drums. We have all experienced the problem.

Interviewer: Thank you. In your neighborhood, how do you address runoff from uphill? Do you have a way to manage it, or do you simply accept it?

Participant: For now, the neighbors still understand our situation. There is no house on the adjacent lot yet, so they have not blocked the route we use to discharge rainwater from uphill. We can direct it there because they have a drainage channel.

Interviewer: So you can direct the water from your property to [named local official/resident]'s drain?

Participant: We have not received formal permission to construct a drain from our property. It is his private drain, but because of the current conditions, fortunately [named local official/resident] understands. I do not know what will happen in the future. The property on the other side belongs to an aunt, and because she has not built a house, water can also be directed there.

Participant: If they close those routes in the future, we will have no choice but to accept it because there will be nowhere else for the water to go.

Participant: For household wastewater, however, we constructed a soak pit so the water does not flow outside.

Additional participant: Rainwater cannot be contained in the soak pit, but it has never entered the house because there is a drain above it.

Interviewer: So there is a drain above the house, and runoff from uphill can be directed there. Meanwhile, you have constructed a soak pit for household wastewater so it does not flow into another person's house or garden.

Interviewer: We have been discussing extreme weather and Community Based Total Sanitation. Now...

Interviewer: What have you done to ensure that your structures are protected? Do you have a dedicated reservoir here?

Participant: No.

Interviewer: What about your toilet? Because of runoff or landslides, there may be concern that it could collapse or become damaged. What ensures that it can withstand those conditions and the dry season?

Participant: Regarding that, I am also a construction worker, so from the beginning I built the toilet in a way that would prevent damage.

Interviewer: Okay. How did you construct it?

Participant: I cast it with cement and reinforcing steel, so it will remain safe even if there is a landslide. It is the structure below [local structure] there (pointing outside). That is the floor. Hehehe.

Participant: We also did not construct a water tank because, to make cleaning easier, we simply prepare drums.

Interviewer: I do the same. Hehehehehehe.

Interviewer: If a pipe is damaged or the water stops flowing, where do you usually report it?

Participant: When a pipe on my property is damaged, I do not call the water utility. I glue and reconnect it myself. If damage occurs along the road, we may report it, but when it is a pipe we can repair, we repair it ourselves.

Interviewer: So you do not have to wait for the water utility. Have residents ever reported a problem to you and you handled it yourself, or is there a water officer?

Participant: All residents here work the way I do. Sometimes, if we meet while doing the work, we cooperate. Our only concern is that the water utility may not authorize us to reconnect the pipe.

Interviewer: But this is only for reconnecting a leaking pipe, correct?

Participant: Yes, when we have a replacement pipe. We have often observed how the utility workers connect pipes. This is only a half-inch pipe. If there is no coupling, we heat the end so it can function as a coupling. We already know how to do that. Officially, we should report it to the utility so that they can (NOISE)...

Interviewer: But the repair is effective, correct? The water eventually flows again.

Interviewer: The next topic is water and sanitation management in your kelurahan. Has anyone ever come here to teach the five Community Based Total Sanitationpillars, such as handwashing, food management, and waste disposal?

Participant: They have never come to the house.

Interviewer: Were you ever invited to an activity?

Participant: Yes, all of us were invited to the kelurahan office.

Interviewer: Who was invited?

Participant: All neighborhood heads.

Interviewer: Were there both men and women among the neighborhood heads?

Participant: There are no female neighborhood heads here. All are men. There are several [local neighborhood] community units. I have forgotten some of the material because I am already old. Regarding the method, um...

Interviewer: But you were taught something. Please explain what you still remember.

Participant: First, we were told to place a handwashing container with a tap in front of the house.

Interviewer: Did you construct one?

Participant: I never constructed one at that time, hehehehehehe, but I always wash my hands when I return from work (laughing).

Interviewer: Is that because your bathroom is located at the front of the house?

Participant: I placed the toilet at the front of the house because I always thought that when guests came, or when children or anyone else gathered at my house, everything should be available at the front. During COVID-19, the handwashing facility was also there, as long as the water was flowing.

Participant: What were the women who attended called?

Interviewer: Cadres.

Interviewer: At meetings like that, when women speak or participate in decisions, are their views heard?

Participant: Some of them certainly are.

Interviewer: We now face climate hazards. It continues to rain even though this should be the dry season. The dry season can also be prolonged, but then rain suddenly occurs. How does the community respond to these climate hazards?

Participant: I can only give my own response because I do not know what others think. When it rains like this and I have not yet gone to work, I have concerns. First, water flows down here from above. Then, if I go to work, I must always carry an umbrella or something similar. The dry season also strongly affects customary obligations and gatherings. During a season like this, we often lack income. We receive income, but expenditures are often high. That may be a personal matter.

Interviewer: That is all right. Customary gatherings also require water.

Participant: When it rains, there are both benefits and disadvantages. I do not like prolonged rain because the paths are often slippery. Fortunately, rain refreshes vegetable leaves. During the dry season, there is only dust and insufficient water.

Interviewer: Regarding water access and sanitation management, if a landslide occurs near the spring, do residents go there together, or is it solely the water utility's responsibility?

Participant: Residents do not go there (NOISE). I also cannot place all responsibility on the utility because my earlier explanation was only my own conclusion. Perhaps the reservoir there has a problem, because the water always becomes dirty when it rains. Whether it is rainy or dry, my wife and I attach a hose to the tap and tie cloth around the end to filter out dirt.

Interviewer: Has anyone here ever said that rain makes it difficult to reach a toilet because the toilet is far from the house, causing someone to fall? This might involve a pregnant woman, a person with a disability, or an older person.

Participant: No. We only attended a meeting with health personnel about constructing safe toilets for people who are ill or pregnant, including the need for handrails. That was all. There was no broader outreach. Only we neighborhood heads attended the meeting, and we were overwhelmed and did not pass all the information to residents.

Interviewer: Do you usually hold neighborhood meetings here?

Participant: ...

Interviewer: Has your neighborhood ever held a meeting?

Participant: Not yet.

Interviewer: Never?

Participant: No. For communal work, I go from house to house or ask women who have a messaging group to help.

Interviewer: Oh.

Participant: They announce that we will conduct communal work on a certain day or date.

Interviewer: Regarding drought, when water does not flow and the stored water is exhausted, have residents ever had to draw water from a spring?

Participant: Yes. About four or five years ago, we experienced a water shortage and went to draw water from a spring in [traditional community area]. We also bathed there.

Interviewer: How far is it on foot?

Participant: It is far. Walking from here toward [study city], it can take an hour to reach the spring. It does not take long by vehicle.

Interviewer: Did you usually use a vehicle?

Participant: We washed clothes there once, and some clothing, including trousers, was eventually lost.

Participant and Interviewer: (laughing)

Participant: That was an effect of the water shortage.

Participant: I do not know what the utility does during the dry season, but I assume that when its source has insufficient water, it transports tanker water from another spring to our reservoir. The water is then distributed through the taps rather than directly from the tanker.

Interviewer: In your view, how responsive is the water-management group or the urban village Community Based Total Sanitation team in addressing climate hazards? You mentioned outreach activities. Is there a special water-management team?

Participant: Not yet. There is only anCommunity Based Total Sanitation team.

Interviewer: How responsive is that team in preparing for drought and deciding what should be done?

Participant: As I said, husbands, wives, and children must cooperate, prepare drums, and guard or monitor the water.

Interviewer: The main point is that they do respond by explaining how people should adapt and prepare when water service stops or a landslide occurs. Is their response good, and do they pay attention to these issues?

Participant: The water utility has never provided information like that. As [participant] said earlier, it has provided no explanation at all.

Interviewer: The Community Based Total Sanitation team has conducted outreach, but you were overwhelmed and have not yet shared it with all your residents (laughing).

Interviewer: That is all right. We are fortunate to live in the city because I am certain health personnel do not work only through neighborhood heads. They also have integrated-health-post cadres who likely share the same information.

Participant: We often receive a great deal of information from health personnel, beginning with the first meeting, although not all of it reaches this area.

Interviewer: Okay.

Participant: For example, they explained that national health insurance has long been available. We also participate in an integrated health post for older people. All those health messages resulted from meetings at the kelurahan office.

Interviewer: You have described community involvement in decisions about water, sanitation, and hygiene. Neighborhood heads and cadres participate most often, and customary and religious leaders are also invited, correct? Who most often makes decisions after a disaster? For example, when runoff comes down from above, who decides how to prevent water from accumulating on your property?

Participant: I am usually the one who makes the proposal. I repeatedly request action at each urban village development-planning meeting, but it has not yet been implemented.

Interviewer: So you make decisions about matters such as constructing drains on both sides and propose them to the kelurahan, but they have not been implemented?

Interviewer: Is there a particular norm or customary practice under which only neighborhood heads are invited to theurban village office?

Participant: School representatives are also invited, including [local secondary school], primary schools, and early-childhood education centers.

Interviewer: What about the marginalized or vulnerable groups you mentioned? Are women and persons with disabilities invited?

Participant: Female cadres and neighborhood heads have been invited.

Interviewer: Have persons with disabilities ever been invited to the urban village office?

Participant: Not that I have seen.

Interviewer: So you have never seen that occur.

Interviewer: What strategy do you use to involve the community in Community Based Total Sanitationactivities, such as communal cleanup work?

Participant: To invite people to communal work, I first go from house to house. If I do not have time to walk around, I ask women or men who manage messaging groups to distribute the information to all residents. Because people still did not cooperate, I also began making announcements during every rotating prayer gathering, such as the Rosary-month prayer. If they have no time to join the communal activity, they must at least clean the area in front of their own house.

Interviewer: So when people cannot gather at the same time, each household cleans its own area. That is a good strategy for involving the community in the kelurahan's Community Based Total Sanitationactivities.

Interviewer: Have you received government assistance or support to ensure access to Community Based Total Sanitationservices?

Interviewer: During rain, for example, are you still able to obtain clean drinking water because of support from a government institution?

Participant: Only the support from the water utility that I mentioned earlier.

Participant: There is something we may tell you.

Interviewer: That is fine.

Participant: Residents here live in harmony, very harmoniously. We are actually enthusiastic about communal work. We once had a neighborhood-owned grass-cutting machine, but it is now damaged. Without that equipment, the activities became less active, even though we remain enthusiastic.

Interviewer: Thank you very much. I also understood that you have reported the proposal to the urban village but it has not yet been implemented.

Participant: I may have reported it a few years ago. I have served as a neighborhood representative for several years.

Interviewer: Apart from the lack of time for residents to gather for communal work to maintain sanitation in the neighborhood, what other barriers are there?

Participant: The first factor is that many residents here are salaried employees.

Interviewer: Okay.

Participant: That makes it very difficult to schedule activities. If I set communal work for Saturday, teachers cannot attend, and often office employees cannot attend either. Only a small number of us are farmers, so we cannot ensure that everyone can gather. The urban village also organizes many activities. In August, for example, we hold neighborhood-level sports activities for Independence Day. That is one occasion when people from the neighborhoods and urban village come together.

Interviewer: Du hitu po cumang cepisa, meaning people only meet during that activity.

Participant: (laughing)

Interviewer: Who most often makes decisions, and who is most influential when a disaster occurs? For example, who decides when action is needed to adapt to, reduce, or prevent a disaster?

Participant: Regarding runoff or landslide problems, affected residents often report them to me.

Interviewer: Okay.

Participant: I can only respond by asking them to conduct communal work. There has been no response from another government agency to date.

Interviewer: Could you explain the specific challenges faced by vulnerable groups, as I mentioned earlier? Why are they not involved, and what strategy might involve them in activities such as communal work?

Participant: Whether people are healthy or vulnerable, I personally always think in terms of humanity, so I do not order or pressure them.

Interviewer: Are there persons with disabilities in this area?

Additional participant: No, perhaps only in [local neighborhood].

Interviewer: Have you heard from another neighborhood head that persons with disabilities were invited to communal work or involved in a social activity?

Participant: As far as I know, that has not occurred. I once helped the neighboring neighborhood transport them when personnel from the Social Affairs Office came.

Interviewer: Oh, you helped take them there?

Interviewer: In your view, how extensive is community knowledge about the effects of climate change? What do people understand about climate change?

Participant: We happen to discuss it during rotating gatherings.

Interviewer: So people talk about it?

Participant: We sometimes discuss climate hazards, as I explained earlier. Rain has benefits and disadvantages. The benefits include vegetables becoming fresh again, while the disadvantages include landslides or runoff.

Interviewer: Who begins that discussion? Is there a presenter, or do you raise the topic yourself?

Participant: The group leader presents the material.

Interviewer: The group leader provides the material?

Participant: I have forgotten what it is called, but there is a question-and-answer session.

Interviewer: Catechesis?

Participant: Yes, catechesis.

Interviewer: That is remarkable, catechesis about climate issues.

Interviewer: Is there local wisdom or a traditional way of adapting to landslides or drought?

Participant: The main adaptation is to remain prepared and maintain cleanliness, including cleaning the drains and remaining vigilant.

Interviewer: Is there also a tradition used to prevent landslides? Forgive me, but you are district , correct?

Interviewer: Are you familiar with the term [local water-source protection tradition], a special District ritual in District that honors water?

Participant: Yes, it is practiced here.

Interviewer: That is a form of local tradition that helps protect springs. When has it been conducted here?

Participant: During Congko Lokap, a ceremony to cleanse a traditional house of impurities so that it is fit for people to occupy and a drum can be hung inside as a symbol of authority over customary lands.

Interviewer: Was that conducted recently?

Participant: It was about six years ago. [local water-source protection tradition] was certainly included because it is not performed frequently and must be connected to a particular ceremony.

Interviewer: Is it performed once a year here?

Participant: It depends.

Interviewer: How often is it usually performed here?

Participant: It may be performed during PENTI, a District thanksgiving ceremony to ancestors, supernatural beings, or the supreme being, Mori Kraeng, conducted formally and joyfully by a community. It is not frequent. Last year, I only participated when Congko Lokap was held in [traditional community area]. To help maintain the spring's stability, we have also conducted reforestation.

Interviewer: Did you participate? Was that the recent activity at the spring above, together with persons with disabilities?

Participant: I participated in an activity last year at [local spring]. The more recent activity may not have involved our group. Because [traditional community area] also has a tourism site, we have planted trees there through an activity organized by the urban district and neighborhood units.

Interviewer: [local water-source protection tradition] and tree planting are forms of mitigation intended to prevent disaster. Is there another local tradition used specifically to adapt to climate hazards?

Participant: No.

Interviewer: Okay, thank you.

Participant: A recent event was a tornado, although it did not originate in [traditional community area]. Because we are connected to [traditional community area], I heard from a neighboring resident that the tornado initially came from [study city], but people there had a customary requirement or practice for driving it away.

Interviewer: Mhm, impressive.

Participant: I no longer know the details, hehehe. They were able to address or withstand it, so its effects were eventually felt in [traditional community area].

Interviewer: Did they know a tornado was coming and therefore manage to withstand it?

Participant: When it arrived, they performed the practice.

Interviewer: Okay.

Interviewer: Because climate hazards have been discussed during catechesis, there must be some community concern. Based on what you know, see, or hear, how dangerous do people consider these hazards?

Participant: There is certainly concern. For example, rain by itself may be manageable, but concern increases when it is accompanied by wind, especially during a prolonged rainy season.

Interviewer: Mhm.

Participant: There is certainly concern because such events occur frequently here.

Participant: If it is not lightning, it is wind. Wind can cause damage, and parts of houses may be damaged, so the concern is always present.

Participant: I personally always prepare a hammer and nails.

Interviewer: For what purpose?

Participant: When strong wind occurs, we can immediately make repairs. That is a form of preparedness.

Interviewer: What experiences have you heard about from people in the neighborhood?

Participant: Hahaha. If a house or pig pen collapses, people usually only complain, "Oh, the wind is too strong," hahahaha.

Interviewer: But they do not take preventive action such as preparing nails and a hammer? Participant: The purpose is to make repairs afterward. In [traditional community area], the government may respond and provide assistance. For events like that here, however, there is no assistance, hehehe.

Interviewer: To your knowledge, is access to Community Based Total Sanitationfacilities, toilets, or water in public places already inclusive? By inclusive, I mean that persons with disabilities can use them without assistance. You mentioned learning that handrails are needed. Have you seen inclusive facilities at the kelurahan office or another public place?

Participant: We only received information during the meeting. Those of us who heard it can, fortunately, apply it in our own construction.

Interviewer: So no inclusive infrastructure has yet been modified or constructed in your urban village ?

Participant: Regarding sanitation, I proposed assistance for people without toilets, including myself, because in my opinion my toilet is not in good condition.

Interviewer: I would not say it is in poor condition. What I mean by inclusive is that it should have handrails. We will all grow old, and persons with disabilities also need support because reaching the toilet can become very difficult.

Participant: Such assistance has not yet been provided to residents.

Interviewer: So there may be no inclusive infrastructure in this area because there are no persons with disabilities nearby. Even at public facilities in the urban village , you have not seen it.

Interviewer: Have you seen such facilities elsewhere in [study city], perhaps at the subdistrict office, with wheelchair handrails and a ramp for persons with disabilities?

Participant: I have seen handrails at the urban village office, hehehehehe.

Interviewer: Oh, they are already there? Are the handrails at the office entrance or in the toilet?

Participant: In the toilet.

Interviewer: That is what I meant. So the urban village office already has them. I hope it becomes an example for the community.

Participant: The meeting was also held at the urban village office.

Interviewer: I think those are all the questions we need to ask. Thank you very much for your time. You were supposed to go to the market but still received us here.

Interviewer: Before I close, is there anything about water, sanitation, and hygiene that I have not asked, or do you have a proposal for improving STBM in your urban village ?

Participant: My main concern is water. When water is insufficient or dirty, we hope the water utility will explain the reason to customers. When people pay their bills, they should be told why the water supply is insufficient or why the water is dirty.

Interviewer: Or why the bill increases even when less water is supplied?

Interviewer: Understood.

Interviewer: Thank you very much for your time, sir. Thank you as well, ma'am. Hehehe.
