# Supplementary material for: Implementing a climate-resilient and inclusive WASH guidance in Indonesia: A mixed-methods case study
Source: PLoS One. 2026 Sep 28;21(9):e0359266. doi: 10.1371/journal.pone.0359266 (PMC13618932; doi:10.1371/journal.pone.0359266)
Supplement: Supplementary file 6 [file pone.0359266.s006.zip › S6 Appendix Transcripts/KII-08_Deidentified_English.docx]

Interviewer

Participant

Interviewer: Thank you very much for making time, [participant]. The interview has three parts: questions about you, your experience and perceptions of inclusive and climate-resilientCommunity Based Total Sanitation , and a closing section.

Interviewer: First, could you tell us about your role in the Urban Village , how long you have served as lurah, and how you have participated inCommunity Based Total Sanitation?

Participant: Thank you. [a recently appointed kelurahan official]. Since I became head of urban village , colleagues from Plan have conducted several Community Based Total Sanitation activities. We also learned aboutCommunity Based Total Sanitation from environmental-health personnel at [local health center].

Participant: As far as I know, our provision of bathing, washing, and toilet facilities has reached 100 percent, according to the previous report.

Participant: Regarding inclusive Community Based Total Sanitation, we also gained knowledge from Plan through several activities, including...

Participant: ...how to provide facilities and infrastructure for people with inclusion or accessibility needs. For example, access to bathing, washing, and toilet facilities also concerns providing access to public places.

Participant: Regarding inclusive and climate-resilientCommunity Based Total Sanitation , we have made several efforts, including initiatives from the kelurahan itself. We planted trees, if I remember correctly, [during a recent year]. We planted at a location with a spring that we were also planning to develop as a tourism site, [local water-source site], because it has a waterfall. We cooperated with [local vocational school] in that tree-planting activity.

Interviewer: Thank you. You learned about inclusive and climate-resilient Community Based Total Sanitation from both health-center environmental-health personnel and Plan. When did you most recently participate in an Community Based Total Sanitation activity or training?

Participant: If I remember correctly, it was during a previous year at a former office. It included mapping and transect activities. We involved several age categories, including younger adult men and women, older people, and women at risk, such as pregnant women. We involved all of them.

Interviewer: Were you a member of theCommunity Based Total Sanitation team at that time, or what was your role?

Participant: According to theCommunity Based Total Sanitation appointment decree, we were members of the STBM team.

Interviewer: What was your impression of the workshop?

Participant: I thought it was a good activity. We are grateful to Plan for providing so much education, especially because there is [a small group of persons with disabilities] here, in addition to high-risk groups such as pregnant women and others. It was very useful because it gave us new knowledge that these people have the same role in daily life as everyone else. The knowledge Plan provided about Community Based Total Sanitation and accessibility was extremely helpful for our community members with special needs.

Interviewer: That is very interesting. You invited people from all age groups and marginalized groups?

Participant: Yes. If I remember correctly, representatives of children with disabilities were also involved.

Interviewer: You understand the fiveCommunity Based Total Sanitation pillars. Are there challenges to implementing them in your area, perhaps related to water access or availability?

Participant: It appears that, perhaps because of climate factors, water shortages have emerged in recent years. Honestly, [study locality] should not normally have water difficulties. Yet at certain times, especially in the dry season, we receive water according to a schedule. Water is not supplied continuously every day. Some areas receive it only once or three times a week. Of course, this...

Interviewer: ...affects implementation of the five pillars?

Participant: It has a very strong effect.

Interviewer: Could you give an example of how lack of water affects the five pillars?

Participant: Handwashing with soap becomes difficult, and wastewater management and other sanitation practices are seriously disrupted.

Interviewer: Does it have a greater effect on women, who manage activities in the household?

Participant: Hahaha. Yes, certainly. It especially affects mothers who care for children and manage bathing, washing, toilet use, and other household activities.

Interviewer: We are experiencing a water crisis. It is ironic that an area once considered a "king of water" now lacks water. Have you heard of any research or people's experiences explaining this? Could the decline in discharge be related to drinking-water companies operating nearby and extracting water in the mountains, or is it entirely due to climate change and drought?

Participant: We can only speak from experience because...

Participant: ...we do not know for certain. One of the commercial water sources in [study city] is located in our area.

Interviewer: The one behind [local secondary school]?

Participant: Yes. The production warehouse of [local company] is in [nearby urban village ], but its borehole is in our area. It may have obtained a drilling permit from the regional government in the past. We do not know how deeply it drills.

Interviewer: Or how it affects us?

Participant: It must have some effect. Extracting groundwater can affect the availability of surface water. This is only an assumption, not a research finding. In recent years, we and perhaps all of [study city] have suddenly experienced shortages, while many commercial-water permits have been issued in [study city]. That may be one contributing factor, but it is only our assumption.

Interviewer: It deserves investigation. I also do not understand how an area once rich in water reached this condition alongside the growth of bottled-water companies.

Participant: I do not know whether higher levels of government previously required company owners to plant trees around springs. At a minimum, the company should have some responsibility, perhaps through corporate social responsibility funds. Mining areas often use such funds to restore forests. The community is aware that drilling may have effects, but we do not have sufficient access to information about them, hahaha.

Interviewer: Even if it is only an assumption, the drilling depth is relevant while local people rely on shallower water. Thank you. This is interesting information.

Interviewer: What types of extreme weather have occurred most frequently in the urban village recently, such as drought or landslides?

Participant: Drought and landslides. Part of our territory lies near river basins, especially agricultural areas, where landslides occur most often. We have warned settlements near river channels to remain cautious. Another relatively frequent disaster is a tornado, which has occurred several times in recent years.

Interviewer: Yes, including the most recent event.

Participant: An event occurred in 2022 in [study urban village ] and [nearby locality], directly along the tornado's path. It has occurred several times. Landslides and drought are most frequent in agricultural areas.

Interviewer: Those events also challenge implementation of the five Community Based Total Sanitationpillars. Have you experienced how landslides or tornadoes affect them?

Participant: A disaster causes losses for affected people. In one case, a house was severely damaged. The household had to evacuate and stay with neighbors. Under such conditions, the five pillars cannot function normally.

Interviewer: Do you know whether water tanks or pipes are specially designed to withstand landslides, so they do not break, or whether tanks are designed to collect more rainwater?

Interviewer: Is there any special design for water and sanitation infrastructure associated with the five Community Based Total Sanitation pillars, such as a landslide-resistant tank or pipe?

Participant: I do not have enough knowledge about that because almost everyone here uses water from the municipal utility. We do not understand the specific designs. As customers, we mainly expect water to be available continuously every day.

Interviewer: What about the design of your own septic tank? Septic tanks have sometimes collapsed during the rainy season. When you built it, did you consider how to make it resilient to disaster hazards?

Participant: Until now, we have generally built structures by following customary practice and copying what other people do. At my house, for example, the septic tank was made by digging a hole about seven meters deep, then covering it with cement in a way that seemed durable. That was all.

Interviewer: Hahaha. I was the same. Only after learning about this did I realize that disaster risks should also be considered when constructing something. Previously, we simply used the ordinary design.

Participant: We never considered disaster risk, hhhhh.

(Interlude while drinking)

Participant: This is outside the interview topic, but there is also a Healthy Schools Movement, usually called GSS. She was a member of the GSS team.

Additional participant: It is the Healthy Schools Movement. There were several considerations behind it. The School Health Unit had become little more than a name.

Interviewer: Yes, it existed mainly to complete administrative requirements.

Additional participant: That was one consideration. Research by experts may also have shown that children's health was declining. In [study district], [local primary school] was selected for the primary-school level, one school for the entire district, as a demonstration or learning site. We are still learning and have not yet become a model because of many factors. [local early-childhood center] was selected for early-childhood education, [local secondary school] for junior secondary school, and [local senior secondary school] for senior secondary school.

Interviewer: So all of the selected schools are from this [study locality] area?

Additional participant: Yes. There is one school at each level for [study district]. The Healthy Schools Movement has five areas of health: nutrition, physical health, immunization, mental health, and environmental health.

Interviewer: I am especially interested in mental health because programs rarely focus on it. Usually, they focus on physical health even though psychological well-being may be weak. Someone once told me about an activity conducted by the [local community organization] community in [local subdistrict].

Additional participant: Regarding mental health, we plan to conduct outreach about violence against children and other mental-health issues. Our greatest current challenge, miss, concerns environmental health. Children's awareness of waste is very low. Genuine internal awareness is very different from compliance imposed from outside. We discuss waste almost every morning: waste, waste, waste, and waste. Yet perhaps only one or two children listen. We collect waste together in the morning, but by the lunch break it has already appeared again. That means awareness is absent. It is extremely difficult to make children understand.

Participant: And this is still inside the school.

Interviewer: Where their behavior can still be monitored.

Additional participant: We have not even discussed behavior outside school. The problem may be lack of awareness. A healthy lifestyle should begin in the household. If there is no waste bin, at least prepare a plastic bag. Our school previously threw waste behind the building, although not into a dug pit. When GSS began, I suggested to the principal that we collect waste in sacks and contact the environmental-cleaning team to transport it. That would be better. We began cooperating in May, several months ago. We have not yet contacted the Environmental Office directly, although this issue requires cross-sectoral cooperation. The principal has not approached the office. I directly contacted the local waste operator, who happens to be from this area. We now secure the school's waste in sacks, and children are asked to bring sacks from home. The school also provides plastic bags, but children's waste can fill more than one bag in a single day. Children in grades one to three follow instructions because they are afraid of their teachers, but those in grades four to six do not. Their attitudes and awareness are very different. Older pupils are actually the ones who litter. Their classrooms are downstairs, and most waste accumulates there. The younger pupils in the upstairs classrooms always bring sacks when asked.

Interviewer: I am very pleased that you initiated a program without waiting for intervention from another party, including the school. That is remarkable.

Participant: Regarding nutrition, every Friday they...

Additional participant: For healthy nutrition, we have established a habit of drinking at least two glasses of water during school activities. Pupils must now bring drinking water from home every day and are not allowed to bring packaged mineral drinks. Parents are encouraged to prepare water in reusable bottles to reduce plastic waste.

Interviewer: Those are excellent innovations, [participant], hhhh. Usually, people wait for an outreach program before acting, but you have already implemented the idea.

Additional participant: When people ask whether there is funding, that is always the final issue. Even a small matter such as plastic bags and transporting waste to a collection site requires funds. As I said, we want good cooperation with the Environmental Office. Ideally, plastic and organic waste should be separated. In reality, our school currently focuses first on keeping the environment clean. We do not yet separate waste because we want to begin with a manageable step.

Interviewer: That small step is very good in practice. We may still be discussing theory, but the school has already begun implementing it.

Additional participant: That is the first area. Another area is handwashing and clean and healthy living. Recently, water service has been unreliable. At a school, even one day without water creates serious difficulty.

Interviewer: How have you handled that situation?

Additional participant: That is the problem.

Participant: We have no choice but to ask pupils to fetch water.

Additional participant: But that creates risk. We worry that an accident could occur during school hours after we have sent someone else's child to collect water.

Interviewer: Although this is outside the main topic, it has broadened our understanding. It will be useful input, and [colleague] may also be pleased to hear your account.

Additional participant: The problem is that the school has not prepared an anticipatory measure, such as maintaining a reserve water supply for periods when water does not flow.

Interviewer: This relates to our discussion because adaptation and disaster mitigation are part of the theory. Thinking about installing a fiber water tank is a mitigation measure. Preparing such a tank at home is also disaster mitigation.

Additional participant: We learned from experience. We had prepared only several drums, and then the water suddenly stopped flowing.

Interviewer: The drums were still insufficient?

Additional participant: Yes. Five people must all bathe before leaving the house in the morning, so the water does not last long.

Interviewer: That is your household's experience. Have you heard how residents manage when water is insufficient?

Participant: Residents make similar efforts, mainly by preparing many drums and jerry cans.

Interviewer: If those supplies are still insufficient, do they fetch water as you described earlier?

Participant: There are several springs in this area, including [local spring], which we visited yesterday. It becomes a backup source when municipal water does not flow.

Interviewer: So it is a backup source, not one used every day?

Participant: Correct. Before [local spring] was transferred to the water utility, it was a public source open to everyone. When utility water did not flow, we fetched water there. When the utility purchased the site, it kindly left one pipe specifically for nearby residents, and other people can also access it. There is another spring, [local spring], in [local settlement]. Several households in [local settlement] can obtain water there, and residents around [local neighborhood] can also access it when water is difficult to obtain.

Interviewer: It is ironic. People in [nearby town] and [nearby district] must laugh when they see us complaining about water on social media, because many people have posted complaints.

Participant: We wonder whether the problem may partly result from errors within the water utility. Perhaps the issue is not an absolute lack of water but a problem with the installation or pipeline network. At a meeting in the subdistrict office, I asked the utility director why reservoirs were not constructed at several locations, for example one storage tank in several urban village . During water shortages, water could remain available in those reservoirs. They could also supply water during fires while people waited for the fire department. We proposed that reservoirs be mapped so that each could serve several customers. Experts in pipeline systems should examine whether such a design is needed in [study city].

Interviewer: The situation does not make sense to me because water service often stops while bills increase.

Participant: Exactly. The water utility purchases springs every year. It owns many sources in [study city], including several here, such as [local spring] and [local spring]. Yet we still experience water shortages. Perhaps sources in our area are also used to meet demand in surrounding parts of [study subdistrict]. The utility may pursue connection targets or quantity rather than service quality. That is only a possibility, but it reflects the reality we observe.

Interviewer: Does the urban village have an organizational structure for an Community Based Total Sanitation or water-management team?

Participant: There is no Community Based Total Sanitation water-management organization like the community water-management bodies found in villages. Because we remain highly dependent on the municipal water utility, no local management structure has been established.

Interviewer: Given unpredictable weather and extreme events, how does the community respond? Are people worried about climate hazards? Have you heard residents complain about unpredictable weather or sudden rainfall that causes landslides?

Participant: There is certainly concern. In addition to health, food availability is a major issue. [study locality] has considerable potential for coffee cultivation.

Participant: During the last five years, coffee production has declined, and in some years there was no harvest. Because many residents depend heavily on plantations, their income falls. Food crops are also affected. Rice fields dominate food production here, and people worry because agricultural yields are declining under extreme weather. The same applies to horticultural or mixed crops cultivated by several farmers. Some residents still depend on cutting timber in the forest for sale as construction material. This remains a major task for the urban village because it disrupts habitat. Recently, people have reported monkeys entering the settlement because their habitat has been disturbed, partly because residents continue to collect timber. The monkeys damage crops and have even entered kitchens to steal rice, hhh. If this continues, the problem may become more severe. Seasonal patterns have also changed. In the past, October to March was the rainy season and April to September the dry season. That is no longer true. This August, after several cold weeks, rain suddenly began.

Interviewer: This season is already the season for celebrations.

Participant: This is usually harvest season, so people hold celebrations. But harvests are now smaller. We also have to discuss community behavior and social problems. What will become of us if gambling continues alongside these problems?

Interviewer: It is ironic that harvests fail while customary celebrations and gambling continue. When people can maintain such behavior, I wonder how they always have money.

Interviewer: At urban village meetings, is Community Based Total Sanitation discussed? How does the community participate, and who is usually invited?

Participant: We usually invite local figures, including neighborhood and community-unit leaders, members of the Family Welfare Movement team, integrated-health-post cadres, and other health cadres.

Interviewer: What about the marginalized groups you mentioned?

Participant: They are included.

Interviewer: In discussions like that, who usually makes the most proposals or speaks most often?

Participant: Because the discussion concerns daily life, women know the issues best and usually make the most proposals.

Interviewer: So women are now confident enough to speak?

Participant: Hhhh, yes.

Interviewer: I have only a few more questions. What specific characteristics influence community participation in STBM? Sometimes, when people receive education or outreach, they first ask who is delivering it. They may listen because the speaker is the head of urban village a health worker, or a teacher, but ignore someone else. In your experience, what type of person does the community trust when receiving education?

Participant: In my experience with activities at the urban village and community levels, the important issue is not who delivers the material but what information is provided. When people receive useful new knowledge, they are enthusiastic. Regardless of the presenter, they welcome information that can produce positive change. When we invite residents to the office, they are often pleased simply because they feel respected. They may not know the topic in advance, but they value being invited and participating. So far, the response has been good without regard to the presenter's identity.

Interviewer: When drought or a landslide occurs, is support provided by a government or nongovernmental institution?

Participant: In our activities, we usually coordinate with the Regional Disaster Management Agency, the Social Affairs Office, and other institutions.

Interviewer: Could you give one example of a recent case and the agencies with which you coordinated?

Participant: During the recent natural disaster, we received considerable support. The Social Affairs Office provided rice and tarpaulins as an emergency response to the tornado. The Regional Disaster Management Agency provided corrugated metal roofing. Indonesian Bank provided construction tools and household necessities such as soap, detergent, and blankets. The local diocese provided cash to reduce the household's burden.

Interviewer: How did that coordination occur? Did you visit agencies, or did they hear the news and contact you?

Participant: The media played a role. Journalists came immediately after the event. Once the story was published, many people learned about it and assistance arrived. As part of the government structure, we also sent formal letters to the relevant agencies.

Participant: The foundation learned about the event through the media and contacted us on its own initiative.

Interviewer: The next topic concerns adaptation and mitigation. We have discussed buildings and infrastructure, such as preparing a fiber water tank. Is there local wisdom or a [Study Area 1] tradition for reducing disaster risk, perhaps a proverb that discourages forest encroachment?

Participant: Honestly, because this is an urban community and [study locality]'s customary institutional structure is not strong, local wisdom has limited influence. Ideally, customary institutions and elders would remind people through sayings such as neka poka puar, meaning "do not clear the forest." People should be reminded that clearing forest increases landslide risk because no large trees remain to hold water on the mountain. We have repeatedly said that the traditional house institution still has an important role.

Interviewer: Has the urban village cooperated with the traditional house institution?

Participant: That is why I raised the issue. In reality, our customary institutions remain weak. If they were strong, people would hear the same message from the traditional house. In villages where culture remains strong, local wisdom may still be influential. Here, modern lifestyles dominate, and young people pay less attention to such matters. That will be a challenge in the future.

Interviewer: Thank you. This may be the final question. Community Based Total Sanitation emphasizes implementation. Have inclusive and climate-resilient Community Based Total Sanitation facilities been constructed in the urban village or at public facilities?

Participant: Honestly, our efforts remain temporary and have mainly involved facilities at the urban village office.

Participant: We previously advised the subdistrict head to design the office so that access to the toilet would be wider. We also initiated the installation of a simple iron handrail for persons with disabilities.

Interviewer: We saw it earlier, hhhhh. That is remarkable. It demonstrates an effect of inclusive and climate-resilient Community Based Total Sanitation. The urban village has responded well. Without learning about inclusive STBM, we might not think that way because we do not currently have disabilities. Yet when we become old or ill, we will also need a handrail.

Participant: The original plan actually included these considerations, but they may have been forgotten during construction. The route to the toilet should have used rough, nonslip tiles. The former subdistrict head regretted the omission after construction was completed. I appreciate that he was very concerned about such issues.

Interviewer: Did the inclusive additions have a financial effect on the urban village ? Perhaps the cost did not increase substantially for a handrail, or was there a separate budget? The original budget may have covered only ordinaryCommunity Based Total Sanitation.

Participant: The features were included as a package in the original construction plan, but they were forgotten during implementation. Therefore, we had to rely on community contributions. Several welding workshops operate here, so we asked them to help prepare the necessary items. There was no specific budget, only self-help support.

Interviewer: That is interesting and creative. You did not wait for outside assistance but proactively used local human resources.

Participant: We could do that because the material had already been taught to us.

Interviewer: How satisfied are you with the inclusive Community Based Total Sanitation program?

Participant: I am very satisfied because it provided a great deal of new knowledge. We once discussed improving access at the traditional house because many people use it. Accessibility does not have to be expensive. A bamboo handrail can be sufficient. We also visited a house occupied by a wheelchair user and advised the owner to improve access or install a simple, inexpensive handrail. They responded positively.

Interviewer: Has anyone already installed one?

Participant: Yes. One household has already made a simple handrail, and nearby people responded positively as well.

Interviewer: Plan's program is ending, and we do not know whether Community Based Total Sanitation activities will continue. How do you view the sustainability of inclusive STBM in your urban village ? Do you have a particular commitment?

Participant: Even if Plan's activities end, at a minimum...

Participant: ...we retain the knowledge that was provided, and we will continue the activities in our own way.

Interviewer: What elements of the five pillars do you intend to continue?

Participant: We can continue simple measures such as using local materials to improve access to bathing, washing, and toilet facilities. Regarding climate resilience, we can replicate some of Plan's activities and include them in development planning through the urban village development-planning meeting. Village and urban village planning meetings differ, but we will continue raising Community Based Total Sanitationin those forums and make efforts within the kelurahan's limited capacity.

Interviewer: Thank you very much. Although I asked only a few questions, you answered all the issues we needed to cover. We are pleased that you shared your experience and expressed a commitment to continue the program after Plan's involvement ends. I will close the interview here.
