# Supplementary material for: Implementing a climate-resilient and inclusive WASH guidance in Indonesia: A mixed-methods case study
Source: PLoS One. 2026 Sep 28;21(9):e0359266. doi: 10.1371/journal.pone.0359266 (PMC13618932; doi:10.1371/journal.pone.0359266)
Supplement: Supplementary file 6 [file pone.0359266.s006.zip › S6 Appendix Transcripts/KII-09_Deidentified_English.docx]

Interviewer

Participant

Interviewer: As I explained earlier through the informed-consent form, you have agreed to be interviewed, so we can begin with the first question. This interview has three parts: general questions about you, your experience and perceptions of inclusive and climate-resilientCommunity Based Total Sanitation , and a closing section.

Interviewer: First, could you tell us about your role in this urban village and how long you have worked here?

Participant: I serve as [a local-government section official].

Interviewer: Mhm.

Participant: I work in [a local-government section] for the community of [study locality].urban village

Interviewer: How is your role related toCommunity Based Total Sanitation

Participant: It is related to maintaining cleanliness. For example, if a latrine is unclean or unsuitable, we usually report it to an agency that may help construct an adequate toilet or latrine.

Interviewer: Thank you. Have you ever participated in activities related to the five Community Based Total Sanitationpillars?

Participant: Yes. I participated in community outreach from house to house.

Interviewer: Mhm.

Participant: It was several years ago, perhaps in 2022. We educated the community about toilet hygiene so that it would not cause diarrhea, skin irritation, or other illnesses.

Interviewer: Before conducting outreach, did you receive training? How did the process lead you to visit houses and explain the five pillars?

Participant: We worked with a team from the Health Office, [local health center], and [local health center] or [local auxiliary health post].

Interviewer: Was Plan also involved?

Participant: Yes, I think Plan was involved.

Interviewer: Where did you meet?

Participant: We met at the lurah's office, and from there we went from house to house.

Interviewer: At the [study urban village ] office?

Participant: Yes, at the [study urban village ] office.

Interviewer: So you first learned aboutCommunity Based Total Sanitation at the lurah's office?

Participant: Yes.

Interviewer: Were participants from other urban village also invited?

Participant: Others were there, but not from other urban village . They were from [local health center], the Health Office, [local health center], and Plan.

Interviewer: [Another local participant] was also there at the time.

Interviewer: They conducted outreach there. Did you visit communities on the same day?

Participant: We went the next day or two days later. During that week, after the office meeting and briefing, we visited houses.

Interviewer: What was your role in the workshop or training?

Participant: I was probably a participant representing the urban village .

Interviewer: What was your overall impression? The five pillars were something new to us.

Participant: They were new.

Interviewer: They addressed seemingly small matters that have major effects. What was your impression?

Participant: I learned that wastewater, which we usually consider useless and discharge into a drain, can be treated so that it becomes cleaner or no longer produces an odor.

Interviewer: A strong odor.

Participant: Yes, an offensive odor that disturbs other people. Dirty wastewater can be treated using gravel.

Interviewer: Through infiltration.

Participant: Yes, infiltration. That was one point I understood (NOISE).

Interviewer: That is one item you personally remember. What was your overall impression of the activity and its benefits?

Participant: My personal impression?

Interviewer: Yes, for example why you considered it beneficial.

Participant: They provided community education, including information about people with disabilities, clean toilets, clean living, washing hands with soap after using the bathroom, and proper handwashing. It was about maintaining a clean way of life.

Interviewer: Thank you. We will now move to your experience and perceptions of inclusive and climate-resilient STBM.

Interviewer: What are the main challenges to implementing the five pillars in [study urban village ]? You received training and conducted outreach, but did you encounter challenges during the household visits?

Participant: Yes. When we conducted outreach, some residents thought we had come to build a toilet or bring assistance.

Participant: They misunderstood education or health information as an assistance program. Once, we visited an older man in [local neighborhood]. He asked, "Did you come to bring assistance, ma'am?" He initially rejected us. We explained that we only wanted to ask where his toilet was. He did not have a private toilet and used his neighbor's.

Interviewer: So he expected assistance.

Participant: Yes, he expected toilet assistance. We asked where he would go if the neighbor did not open the bathroom or house at night when he urgently needed to defecate or had stomach pain. He answered, "Well, I just defecate in the garden. What else can I do?"

Interviewer and Participant: Community awareness was low.

Participant: We explained that if he defecated in the open, flies could land on the feces and then on his food, causing diarrhea or another illness. Only then did he understand. Sometimes people also dug a small hole, defecated in it, and covered it again.

Interviewer: Jesus...

Participant: But what if a dog comes and digs it up again?

Interviewer: Does that still happen?

Participant: I do not know what happened afterward.

Interviewer: After the education, understood.

Interviewer: What about other challenges? For example, to implement handwashing with soap, how adequate is water availability in [study urban village ]?

Participant: Water availability in [study urban village ] is fairly good and rarely stops, perhaps because we are close to the main source.

Interviewer: So in terms of implementing the five pillars and water availability...

Participant: Water is not a problem. The problem is community awareness and perhaps human-resource capacity, miss. Some people still do not have toilets and use their neighbors' facilities. During the visit, a woman from the Health Office asked an older man what he would do if he urgently needed to defecate at night. He answered, "Miss, I will just go in the garden. What else can I do?" That occurred in [local neighborhood].

Interviewer: Perhaps you could help monitor the current situation.

Participant: He is often ill as well.

Interviewer: Our district has declared itself open-defecation-free, yet behavior like that apparently still exists.

Participant: Perhaps the situation changed after the education. People were advised to construct a simple goose-neck latrine by digging a pit, so they would not defecate in the open.

Interviewer: They were also taught how to construct one?

Participant: Yes, they were taught.

Interviewer: Let us move to extreme weather. What types of extreme weather occur frequently in this urban village , such as floods, landslides, or drought?

Participant: Landslides are unlikely. Flooding can occur when water overflows from drainage channels. At the old office, we often saw the drain overflow.

Interviewer: Was that because something accumulated in the drain?

Participant: Waste.

Interviewer: Correct, because it is also a low-lying area.

Participant: The drain usually overflows.

Interviewer: Under those conditions, what challenges arise in implementing the five pillars during extreme weather, such as flooding?

Participant: We once proposed constructing drainage along the road, but it has not been addressed, perhaps because no construction program is available. We do conduct Clean Friday activities with the community, including cutting grass and removing waste from the drains near the old urban village office.

Interviewer: So along the road in front of the urban village office, there is no proper drainage channel, and the response remains entirely manual.

Participant: Yes.

Interviewer: During extreme events, has any water or sanitation infrastructure been damaged, apart from the absence of drainage?

Participant: Such as a leaking pipe?

Interviewer: Yes.

Participant: No.

Interviewer: Have residents reported any damage?

Participant: No.

Interviewer: What about at the spring?

Participant: No.

Interviewer: So there has been no such report (NOISE).

Interviewer: Regarding structural design, is a water-storage reservoir specially designed to withstand landslides or the dry season? Have you received any information about that?

Participant: We do not have a reservoir of our own. The main reservoir is farther below [study urban village ], in the area toward...

Additional participant: [local spring].

Participant: Yes, [local spring].

Interviewer: Have you received information that the reservoir there was specially designed to withstand landslides or drought?

Participant: I think it may have been, because it was constructed directly by the municipal water utility rather than the community.

Interviewer: The urban village was not involved?

Participant: No.

Interviewer: What form of support does the urban village provide?

Participant: Mainly cleaning. We remove wild vegetation so it does not encroach on the spring. The community and urban village officials conduct communal work together.

Interviewer: How does the community of [study urban village ] address extreme weather? Apart from communal cleaning, do you conduct other activities, such as maintenance of water facilities or reforestation?

Participant: Oh, reforestation.

Interviewer: Are there activities like that?

Participant: Tree planting has been conducted near [local reforestation site]. Some of the land is private rather than public, but trees were replanted there, including through a regional-government activity.

Interviewer: When rain makes the water turbid, what maintenance system is used to ensure residents receive clean water? Is there government or urban village support, or do households act individually?

Participant: We rarely receive reports of turbid water. No one has reported that water became turbid during the rainy season, so we do not know about it (NOISE).

Interviewer: Does it happen at your own house?

Participant: Yes, it does.

Interviewer: What do you usually do?

Participant: When the water is turbid, we store it and wait for the sediment to settle before using the clean portion, or we filter it.

Interviewer: The next topic is water and Community Based Total Sanitation management in the urban village , but we can keep the discussion relaxed.

Interviewer and Participant: Relaxed, hahahahaha.

Interviewer: Is there an organizational structure for water management and the Community Based Total Sanitationteam in the urban village ?

Participant: It was not established by the urban village . The activity involved cooperation, and we were only included as members of a structure they formed. For Community Based Total Sanitation, I was one of theurban village representatives. I think there is a formal structure, but you should ask...

Additional participant: Perhaps the health center.

Participant: You should ask the health center, auxiliary health post, or Health Office. The people involved included [named person], [named person], [named person], [named person], [named person], and [named person].

Interviewer: Once the group was formed, what roles and responsibilities did its members have toward the community?

Participant: As far as I know, Community Based Total Sanitationconcerns more than water.

Interviewer: Correct.

Participant: Our activity concerned not only clean water but also sanitation, such as clean toilets and preventing open defecation. That is what I remember from the community outreach and education.

Interviewer: So, as anCommunity Based Total Sanitation team member, your role and responsibility were to conduct community outreach?

Participant: To educate people about clean latrines and preventing open defecation.

Interviewer: Were only women involved in the outreach and education, or were men also involved?

Participant: Men were also involved.

Interviewer: Were there more women or men?

Participant: More women.

Interviewer: Were persons with disabilities also invited?

Participant: They were taught how to wash their hands at the office and what to do when going to crowded places.

Interviewer: They received that instruction?

Participant: Yes. [named person] and another woman worked with them using strings or cords.

Interviewer: Oh, mapping.

Participant: Yes, mapping, with colored paper (NOISE).

Participant: We only observed and were not deeply involved.

Interviewer: Did they later join the house-to-house outreach?

Participant: Several months later, an activity was held for persons with disabilities.

Interviewer: We now face unpredictable weather. Last year this month was dry, but now it is suddenly raining. Have residents expressed concerns or complaints to the urban village about climate hazards?

Participant: So far, no one has come to complain, so we cannot draw a conclusion.

Interviewer: What is your personal response to the current climate situation?

Participant: Personally, we must protect our health, for example...

Participant: What specific aspect did you mean?

Interviewer: Water and sanitation.

Participant: During the rainy season, water often becomes turbid because mud from the source may enter the pipes. Personally, I try to filter water at the pipe or tap using cloth, or store it until the sediment settles before using it.

Interviewer: Mhm. In your view, since there is already...

Participant: Yes.

Interviewer: How quickly did the water-management group and Community Based Total Sanitationteam members, such as [named person] and [named person], respond with outreach in view of current extreme weather? Was their response fast or slow?

Participant: During the earlier program, their response was fast. I think that was in 2022. They quickly addressed the issue and provided soap bottles so children could wash their hands after using the bathroom. But that program is no longer active.

Interviewer: The urban village does not have such a program?

Participant: No, unless residents submit a complaint. If no report is received, we do not independently visit and inspect.

Interviewer: Are community members involved in decision-making about Community Based Total Sanitation?

Participant: They do not make the final decision, but they provide proposals and suggestions. We invite all neighborhood and community-unit leaders in [study urban village ], including representatives of several [local neighborhood] units. We accept their proposals and suggestions.

Interviewer: But the urban village still makes the dominant or final decision?

Participant: Yes.

Interviewer: You mentioned neighborhood and community-unit leaders. Are women's groups also invited?

Participant: Yes, including various cadres and cadres for older people.

Interviewer: Are only neighborhood leaders and cadres invited?

Participant: Community leaders are also invited.

Interviewer: Are these groups selected through a particular urban village assessment? Why are other vulnerable groups not invited? Is there a cultural norm under which women's voices are not considered important, so they do not need to be invited?

Participant: No, that is not the reason.

Interviewer: Then how do you decide whom to invite?

Participant: At that time, the urban village office was small, so we invited only several people, such as customary elders, neighborhood and community-unit leaders, and cadres. We invited them because they could later conduct outreach or education in their own communities. As members of the team, people might listen to them.

Interviewer: Could you give an example of how residents communicate or coordinate with the head of urban village to address water-access problems?

Participant: Could you repeat that?

Interviewer: For example, when they want to complain that water service has stopped.

Participant: Or that the water is dirty?

Interviewer: How do they coordinate or communicate with the urban village ?

Participant: Residents usually report directly to the urban village . The urban village then sends a letter to the relevant institution, such as the municipal water utility. The utility comes to inspect the water problem.

Interviewer: What about sanitation, such as a latrine damaged by a landslide? Has that occurred?

Participant: Several years ago, a latrine collapsed, not because of a landslide but perhaps because of very heavy rain.

Interviewer: How was the situation handled?

Participant: We reported it directly to the Health Office, if I remember correctly, or to the health center. They came and constructed a replacement.

Interviewer: Was the construction supported by the urban village or the relevant agency?

Participant: It was funded directly by the relevant agency. We only helped coordinate and monitored the progress of construction.

Interviewer: What government support is provided to ensure that everyone has adequate access to water and sanitation?

Participant: Do you mean special support? Usually agencies inspect homes through activities conducted by Plan or Community Based Total Sanitation. If they find or receive a report that a household's toilet is unsuitable, they may construct an adequate, clean toilet. Several households have received toilet assistance.

Interviewer: Could you describe one successful example in which you communicated a community complaint about water or sanitation and the relevant agency responded?

Participant: Made a decision?

Interviewer: Yes, responded to the complaint.

Participant: In one neighborhood, I think it was [local neighborhood], a household did not have a clean latrine. The neighborhood head reported it to the head of urban village . The head of urban village sent a letter explaining that the household's toilet was unsuitable. Several months later, officials surveyed the site, confirmed that the facility was not suitable, and constructed a new one. I think several households received assistance that day.

Interviewer: Did you encounter any particular challenge obtaining support from the relevant agency after coordinating with it?

Participant: No.

Interviewer: So the support was implemented?

Participant: Yes. The agency also conducts its own survey because we cannot submit a false report. Officials confirm that a household genuinely lacks a clean latrine. The urban village report verifies the condition, the relevant agency surveys several sites, and then construction is undertaken.

Interviewer: It is excellent that many agencies now respond. In the past, we might have thought...

Participant: In the past, people only talked without taking action.

Participant: Recently, a household affected by a tornado quickly received housing assistance. [The household recently received housing assistance for materials and labor, and construction began promptly.] The response was remarkable.

Interviewer: People now know which channel to use.

Participant: Government is also more open. It no longer provides assistance only to its own people. Everyone is treated equally, and everything is transparent, so it cannot be concealed.

Interviewer: Correct. Government is now transparent. (...)

Participant: This is only a small community, but many parties assisted after the recent event. Assistance did not come only from the housing agency. Another institution brought corrugated roofing, perhaps the family-planning agency.

Participant: Yes, the National Population and Family Planning Agency also brought assistance.

Interviewer: That is cross-sectoral cooperation.

Participant: The important point is to respond quickly. As lower-level government employees, we interact directly with the community. When a report is received, we write to higher levels or the relevant institution. We do the same for land and property tax records, such as requesting a change of name when a parcel no longer belongs to the person shown in the record. We prepare a letter and take the identity card and documents directly to the revenue office so that the correct name appears the following year.

Interviewer: Let us continue.

Participant: Yes.

Interviewer: Do particular characteristics, such as being a urban village employee, being a woman whose voice must be heard, having a disability, age, or ethnicity, influence community participation iCommunity Based Total Sanitationn ? Do residents listen because of such characteristics?

Participant: People are now knowledgeable and can evaluate what they hear. At least some can see that women and men have come to provide something useful. When we teach positive practices, such as preventing open defecation and explaining its consequences, people listen. Many residents now understand changes promoted by the government. We are part of the government, but they do not listen only because of our status.

Interviewer: What about in the past?

Participant: They would say, "We are going to the garden."

Interviewer: Or, "Who are you?"

Participant: Or, "What assistance did you bring?"

Interviewer: Sometimes they refused to listen unless the speaker was a urban village or health employee.

Participant: Correct.

Interviewer: But now they will listen to anyone who brings useful information.

Participant: Yes, they will.

Interviewer: The community most often receives information from the urban village and health personnel. Do persons with disabilities also listen because they are included in activities?

Participant: Yes. They receive particular attention here. We have [a small number of persons with disabilities] registered at the local office.urban village They often receive assistance from the Social Affairs Office, Plan, the Health Office, or the auxiliary health post, so their needs are considered.

Interviewer: Is there a particular person or key community figure who controls water and STBM services?

Participant: No, mainly the kelurahan office.

Interviewer: What about a customary leader, for example when people meet at a traditional house to discuss a spring?

Participant: Yes. A spring here was purchased by the water utility, and we held a meeting at [traditional community venue]. We met there to reach an agreement among customary elders, landowners, and the urban village government.

Participant: The water utility also participated. The meeting's decision was then conveyed to the utility as the community's position.

Interviewer: In a meeting like that, is the customary leader's voice heard most strongly, while the urban village participates only as an invited party?

Participant: No. The head of urban village convened the meeting, but it was held at the traditional house because there was a minor dispute. Some people did not want to release the spring, so the parties were brought together.

Interviewer: Mhm.

Participant: Customary elders from several traditional-house communities, including [traditional community venue] and [named person]'s group, attended. They reached a shared agreement concerning what people wanted, such as whether compensation should be paid and how much.

Interviewer: (...)

Participant: Compensation was eventually paid.

Interviewer: The next topic concerns vulnerability. There are persons with disabilities, gender differences, and older people.

Participant: There are no transgender people here, hahahahaha.

Interviewer: No, I mean...

Participant: You mean gender as in women?

Interviewer: Yes. In some customary settings, women's voices are not heard. Does any factor here affect people's ability to access Community Based Total Sanitation services?

Participant: No. Everyone can access the services. No particular group is more influential in that way.

Interviewer: Do persons with disabilities face any special challenge when going to public places? The urban village office now has...

Participant: The toilet has handrails for them, and there is a seated toilet.

Interviewer: What about other public places in the urban village ?

Participant: I do not know about schools. The health center must have accessible facilities. At [local auxiliary health post], there is already an access route and handrails for persons with disabilities.

Interviewer: This next question may concern households. Is there local wisdom for reducing disaster risk?

Interviewer: District people have the term [local water-source protection tradition]. What local wisdom or tradition is used here to reduce disaster risk?

Interviewer: Oh, you do not understand the question. That is all right.

Participant: We are also migrants here.

Participant: Perhaps [named person] could answer that question.

Interviewer: I mean traditions such as [local water-source protection tradition], whichDistrict people use to reduce disaster risk. Does the community in this urban village have a similar practice?

Additional participant: (...) ???

Additional participant: Local wisdom used to be deeply embedded.

Participant: The area has become increasingly urban.

Interviewer: Hehehehehe.

Participant: In the past, when the rainy season approached, older people prepared and stored their harvest in a special place. Then, during the rainy season, they could remain at home without going out.

Interviewer: Yes, it was called a langkok, a place for storing harvests, hahaha.

Participant: Correct, [named person]?

Participant: If they knew rain would begin the following month, they collected a great deal of firewood and stored it in the leba, the space above the cooking fire.

Participant: That was how our grandparents lived.

Interviewer: Those practices no longer exist?

Participant: Hehehehehe.

Participant: Motorcycle taxis pass frequently, and traveling wood sellers also come by. Interviewer: Modern technology has made everything easier.

Participant: Yes, this is the modern era. When we were in primary school, people still stored corn and purchased salt in advance, placing it in large baskets so they did not have to leave the house during the rainy season.

Interviewer: Now technology makes things easier even during rain.

Participant: Wood and vegetable sellers continue to pass by, so people do not need to prepare as long as they have money. Older generations were not as wasteful as we are.

Interviewer: Given the climate hazards you described, what concerns does the government have, and what actions is it taking now to reduce disaster risk?

Participant: Conditions here are not extremely severe, so there is no special rainy- or dry-season action. You mean specifically in [study urban village ]?

Interviewer: Earlier you mentioned...

Participant: Clean water.

Interviewer: And cleaning drainage channels in preparation for rain.

Participant: Or gathering and removing debris after rain.

Additional participant: (...) ???

Participant: Relevant agencies may also conduct surveys.

Additional participant: (...) ???

Participant: As I said, residents report to us, and we, as the lowest level of government, convey the issue to the relevant government agency.

Interviewer: Did Plan teach or socialize the CCRIW program, or did the material cover only Community Based Total Sanitation? Was inclusive and climate-resilient STBM included?

Participant: Yes, the climate-related component was included.

Interviewer: How satisfied are you with the program's implementation in the urban village ?

Participant: As a participating member, I was satisfied because we learned ways to address all those issues.

Interviewer: Was there any CCRIW idea that you found unacceptable or unsatisfactory?

Participant: No. They taught useful matters such as sanitation and the five Community Based Total Sanitationpillars.

Interviewer: Has the urban village adopted any CCRIW activity? Could you give an example?

Participant: For example, we installed handrails to help persons with disabilities reach and use the toilet. The toilet at the back is a seated toilet.

Interviewer: So it is inclusive. Has the building also been designed to withstand landslides, especially because I consider this area vulnerable?

Participant: Yes, it is very close to us.

Interviewer: Close to the mountains?

Participant: We do not know where we would evacuate, hahahahaha. But before constructing a building, the builders and supervising consultant presumably assess whether it can withstand landslides.

Interviewer: When CCRIW measures are adopted, such as an inclusive toilet, do they have a financial effect by increasing construction costs?

Participant: No, because those features were already included in the office-construction project.

Interviewer: Do you have any suggestion for reducing costs, or is that unnecessary because the features were already budgeted?

Participant: They were already included in the budget.

(...)

Interviewer: I think those are all my questions.

Participant: Do you want to photograph the facility as evidence?

Interviewer: Yes, in a moment. There were many issues in the interview guide, but a single response often answered several questions. Thank you very much for sharing your ideas and experience with us.

Interviewer: We will summarize your responses. As I explained at the beginning, we will follow procedures to protect your confidentiality. Thank you very much.

Interviewer: I will end the interview now, hehehehe.
