# Supplementary material for: Implementing a climate-resilient and inclusive WASH guidance in Indonesia: A mixed-methods case study
Source: PLoS One. 2026 Sep 28;21(9):e0359266. doi: 10.1371/journal.pone.0359266 (PMC13618932; doi:10.1371/journal.pone.0359266)
Supplement: Supplementary file 6 [file pone.0359266.s006.zip › S6 Appendix Transcripts/KII-11_Deidentified_English.docx]

Interviewer, Participant

Interviewer: All right, sir. Thank you for your time. I explained the purpose of my visit earlier. We can begin with the first question. Could you explain your role in the village and how long you have been here in [study village]?

Participant: First, I would like to answer regarding my role, particularly with Plan.

Interviewer: Yes.

Participant: During my work with Plan, I served as a key point of contact for the community. I conveyed all guidance from Plan to the community. As the primary person responsible, I had to think about how to ensure that the community followed the guidance and rules provided by Plan. That is the first point. What was the second question?

Interviewer: How long have you been there?

Participant: [village official with several years of service]

Interviewer: Oh, as an acting official.

Participant: Yes, that is correct.

Interviewer: But you do not live in [study village]?

Participant: No. I travel from here in the morning, leave the office at two, and return here.

Interviewer: You live in [participant's home urban village ], correct?

Participant: In [a nearby locality].urban village

Interviewer: All right. Have you ever participated in an activity called inclusive and climate-resilient STBM (Community Based Total Sanitation)?

Participant: Yes. I participated, but only in a socialization activity at the village office. After it ended, we conveyed the information to the community. I did not make the rules, but I explained how they should be applied.

Interviewer: What was your impression of the socialization?

Participant: It was good, good, very good.

Interviewer: In what way was it good?

Participant: It was highly beneficial for the community. The activity was very beneficial.

Interviewer: What form did the socialization take? Was there triggering, or did they only explain the meaning of inclusive and climate-resilient STBM(Community Based Total Sanitation)?

Participant: There was an explanation, and we also went directly to nearby locations around the village office.

Interviewer: To inspect or monitor them?

Participant: Yes, first to inspect them and then to explain better ways of using them.

Interviewer: Using toilets?

Participant: Better toilets and better ways of constructing toilets.

Interviewer: At residents' homes?

Participant: Yes.

Interviewer: All right. We would now like to understand your experience and perception of inclusive and climate-resilient STBM(Community Based Total Sanitation). So far, have you modified or constructed new STBM (Community Based Total Sanitation) infrastructure to make it more climate resilient? Following the socialization, did you make modifications at the village office or residents' homes?

Participant: We visited residents' homes and provided guidance about the best approaches. That day, I and several village staff visited several houses. The community response was good. We did not force anyone. We only conveyed the information, and they responded, “Okay, thank you.”

Interviewer: What modifications were made at the village office? How were the sanitation facilities or the toilet access modified?

Participant: Thank you. The village office is a public facility. We paid full attention to the needs of people with disabilities. Following the guidance, they visited and examined the location and considered how people would move through it. We arranged everything properly.

Interviewer: Does it have a handrail?

Participant: Yes, it has a handrail. We also collaborated with others. According to Plan's assessment, [study village] ranked first for sanitation.

Interviewer: Was the handrail made manually, or was it made of metal?

Participant: Metal. It was welded and embedded.

Interviewer: What was the floor like?

Participant: Ceramic tile.

Interviewer: Ceramic tile. Was the entire route from inside the village office to the toilet tiled?

Participant: Yes, all of it was tiled.

Interviewer: The entire route?

Participant: Yes, beginning at the entrance.

Interviewer: Would that make it slippery?

Participant: No, it was not slippery.

Interviewer: So it was not the slippery type of tile?

Participant: Correct.

Interviewer: In your view, were these efforts beneficial for vulnerable groups, such as people with disabilities, pregnant women, or children using the toilet?

Participant: Yes, they were very beneficial. The village office is a public facility for everyone, including visitors and community members attending activities. We often hold meetings there. We specifically invite people with disabilities and older people. Plan also provided guidance on how to arrange the facility. As I mentioned, Plan's sanitation assessment ranked us first in [study city] Subdistrict.

Interviewer: What impressions did people with disabilities or other community members have after using the sanitation facility?

Participant: They said it was good and rated it positively. It was the first time they had used a toilet like that and the first time they had seen one.

Interviewer: After seeing the facility at the village office, did anyone adopt or imitate the same features at home?

Participant: No.

Participant: Toe mangan e, meaning there was none.

Participant: The main factor was funding.

Interviewer: A handrail does not necessarily have to be welded metal like the one at the village office. Some people use wood for residents with disabilities. Did anyone do that?

Participant: I do not know about every community member's home, but as far as I know, no one did.

Interviewer: All right, one moment.

Participant: Regarding sanitation, we followed Plan's guidance.

Interviewer: Besides what you have described, did Plan Indonesia or the government conduct any other activities concerning climate-resilient water and STBM(Community Based Total Sanitation) in [study village], apart from CCRIW?

Participant: There is another activity there that is similar, conducted by [local NGO]. We are now prioritizing protection of water sources. During the next month, [local NGO] will provide 2,000 seedlings to plant around the water sources.

Interviewer: Apart from CCRIW socialization, did Plan Indonesia conduct any other activity to make STBM(Community Based Total Sanitation) climate resilient?

Participant: No.

Interviewer: So the only activity was modification of the sanitation facility?

Participant: Yes, the sanitation facility.

Interviewer: Regarding village funds, were village funds used to rehabilitate or construct the sanitation facility?

Participant: At present, no. Honestly, no.

Interviewer: But you said the sanitation facility had been constructed. Did the funding come from Plan or from village funds?

Participant: From village funds. There was no funding from Plan Indonesia. Honestly, there was none, although I do not know about other places.

Interviewer: So Plan's role regarding the sanitation facility was not to construct the infrastructure, but to explain how it should be built?

Participant: Plan showed us the way. In previous years, Plan only provided guidance on appropriate and adequate sanitation. The funding came from our own resources.

Interviewer: So the funding still came from village funds?

Participant: Yes.

Interviewer: All right. You made the facility as a follow-up to Plan's socialization. Was the modification also made because disasters frequently occurred?

Participant: No. During my time there, no disasters occurred.

Interviewer: What climate hazards commonly occur there?

Participant: There are no hazards. During the rainy season, for example, there are no natural disasters. The only problem is insufficient water.

Interviewer: So drought also occurs frequently there?

Participant: Yes.

Interviewer: What happens to the sanitation facility or toilet when water is suddenly unavailable?

Participant: Most community members pump water from (NOISE) (10:48).

Interviewer: All right. The next question focuses on the CCRIW workshop and training. In your view, was CCRIW, which connects water and sanitation with climate change, suitable for implementation in your village?

Participant: Yes, it was suitable. Our primary constraint is water during the dry season. Nearly every household purchases a pump and hose to bring water to the house.

Interviewer: Do they now use boreholes?

Participant: No, not boreholes. They pump water from the river.

Interviewer: From the river. I see.

Participant: Yes.

Interviewer: Were the pumps financed by village funds or through collective contributions?

Participant: They were purchased privately by community members.

Interviewer: That is significant. So the facilities exist, but water becomes difficult to obtain during drought?

Participant: Yes.

Interviewer: I will ask you to rate the CCRIW activity on a scale from one to five, where one means dissatisfied and five means satisfied. What rating would you give?

Participant: I was satisfied. The community also welcomed it. The main constraint is funding. When sanitation facilities are damaged, how can we help residents? They must make their own efforts. At most, we can explain how to construct proper sanitation facilities.

Interviewer: On the scale from one to five, what number would you choose? One means dissatisfied, five means satisfied, and three is neutral.

Participant: I will choose three.

Interviewer: That is fine. Thank you. Which aspect made you less satisfied?

Participant: With Plan, or?

Interviewer: No, with the program we discussed.

Participant: Regarding dissatisfaction, the first issue is the community. Our main constraint is money. What do I mean? People's attitudes are now problematic regarding sanitation. When a sanitation facility is slightly damaged, they say, “Sir, this has happened to us. Is there funding to help repair our sanitation facility?” That is what I dislike about the community in [study village]. That is why I selected three.

Interviewer: So the issue is not only the program but also community readiness.

Participant: Yes, they are not sufficiently prepared.

Interviewer: Thank you. The next question concerns adoption. You explained that you constructed a sanitation facility at the village office. Is there anything else related to climate resilience that could be adopted from CCRIW? For example, you mentioned that [local NGO] would provide tree seedlings.

Participant: Two thousand.

Interviewer: Those seedlings are one form of disaster mitigation. Apart from that, was there anything else from Plan?

Participant: There was nothing else from Plan for my community. Honestly, there was nothing. What did Plan provide? Only information about how to do things.

Interviewer: All right. Thank you for your honesty. One successful adoption from CCRIW was the construction of an inclusive and climate-resilient sanitation facility. In your view, what is needed to ensure the program's success and follow-up? Do you need additional training or special guidance?

Participant: From Plan in the future?

Interviewer: Yes, or perhaps integration with other programs.

Participant: I will answer cautiously because we do not know how many more years we will remain there. I am concerned that if we say we need something, we may suddenly be transferred back to [study city].

Interviewer: That is understandable. Do you have any ideas about it at present?

Participant: Not at this time.

Interviewer: All right. You said the village had adopted CCRIW activities. What was the funding source? Was there a specific budget nomenclature for CCRIW in the village fund, or which budget category was used to construct the sanitation facility?

Participant: There was a specific budget.

Interviewer: A specific allocation from village funds?

Participant: Yes.

Interviewer: Did this create a financial burden for the village, such as depleting village funds?

Participant: No, because the budget line already existed.

Interviewer: Because it already had a budget line.

Participant: Yes.

Interviewer: Thank you. Do you have suggestions for making the program more cost-efficient?

Interviewer: From Plan, or?

Participant: From the village, from Plan, or based on your own ideas.

Interviewer: From my perspective as an official in [study village], it is manageable because we are prepared to finance it. When funds are available under a budget line, the activity must be implemented. Regarding Plan, I hope the relationship between Plan and [study village] is not severed. During these two years, village officials and Plan partners have become like a close family.

Interviewer: So, in your view, cost savings are not necessary because a budget line exists and sufficient funds remain.

Participant: Yes.

Interviewer: All right. Regarding program sustainability, what approach could allow CCRIW to continue in your village? Participant: I hope it continues. That depends on Plan.

Interviewer: Plan's program is approaching its end. Is there anything the village can do independently without Plan's intervention?

Participant: In the future, I hope to continue applying and sharing the knowledge I received from Plan with the community.

Interviewer: Do you have an idea such as, “Without Plan, I will implement this activity”?

Participant: Yes, I can continue.

Interviewer: What specific idea do you have? Could you give one example?

Participant: A small example is that I plan to implement something there concerning sanitation and funding.

Interviewer: Yes, all right.

Participant: That is all.

Interviewer: Apart from funding, what else have you considered?

Participant: Only that. Everything in the village involves funding. Community requests ultimately require money. If funding is unavailable, I say that we must postpone the activity.

Interviewer: All right. We will now discuss enabling and inhibiting factors for CCRIW. Does your village have an organizational structure for water management and an STBM(Community Based Total Sanitation) team? Villages usually have an STBM(Community Based Total Sanitation) team.

Participant: Yes, we have an OPAM( Community based water system).

Interviewer: An OPAM( Community based water system). Does that manage piped water, or? Participant: No, it manages village water. This water is specifically for residents of [study village]. We call the group responsible for maintaining it OPAM( Community based water system). That is their work. However, during dry seasons, the flow remains but water comes from [neighboring study village]. As the pipe crosses residents' gardens, if someone is growing vegetables or tomatoes, the hose is sometimes cut.

Interviewer: They take water from it, causing difficulty downstream. Participant: Yes. People downstream around [local hamlet] experience severe water shortages, particularly for drinking water. They now have no choice but to buy pumps to draw water from the river.

Interviewer: Can that meet residents' needs?

Participant: Yes.

Interviewer: They use their own funds. Fortunately, the river still contains water.

Participant: Yes.

Interviewer: Fortunately, although we do not know the water quality. Participant: We also use river water at the office. Interviewer: So you also use a pump?

Participant: Yes. The hose is large, and the electrical cable is approximately 300 meters long. Interviewer: So OPAM ( Community based water system) is temporarily not functioning? Participant: Yes, temporarily. Interviewer: During which months is water normally abundant? Participant: Approximately December through July. Water flows abundantly then.

Interviewer: What about the STBM(Community Based Total Sanitation) team? Is there a village STBM(Community Based Total Sanitation) team?

Participant: At present, there is none.

Interviewer: There is no team that conducts monitoring visits? Which STBM(Community Based Total Sanitation) team conducted the household monitoring?

Participant: We only use OPAM( Community based water system) because its work concerns water inspections. Interviewer: So there is no village STBM team that includes health personnel and conducts monitoring and evaluation by visiting households and inspecting sanitation facilities?

Participant: There is also such a group. However, the main obstacle during this season is water scarcity. People can be very demanding. When we visit to inspect facilities, they say, “You come every day or every week to inspect us. We are struggling with water and thought you were coming to bring water.”

Interviewer: But is there a formal STBM(Community Based Total Sanitation) management group or team in the village? In other villages, there is usually a village STBM team, and the village provides transportation funding for field visits.

Participant: Yes, we have one. The team works with OPAM( Community based water system).

Interviewer: So there is both a village team and OPAM( Community based water system)?

Participant: Yes.

Interviewer: Thank you. Does the STBM(Community Based Total Sanitation) team include more women or more men?

Participant: It is balanced, including health personnel.

Interviewer: What are the roles and responsibilities of OPAM( Community based water system) and the STBM(Community Based Total Sanitation) team in village water management, as you understand them?

Participant: Based on my observations, they cooperate well and always coordinate with me. If they do not contact me, I call them and say, “Let us visit each household to ask about or discuss the current water situation with the community.”

Interviewer: Apart from gender balance, are people with disabilities included in OPAM( Community based water system) or the STBM(Community Based Total Sanitation) team?

Participant: Yes.

Interviewer: How many people with disabilities are involved?

Participant: We have two in [local hamlet].

Interviewer: Are they members of the STBM(Community Based Total Sanitation)team?

Participant: Yes.

Interviewer: Are any members of OPAM( Community based water system)? Participant: No.

Interviewer: So they are only on the STBM(Community Based Total Sanitation) team?

Participant: Yes.

Interviewer: Thank you. How has the community responded to current climate hazards? You mentioned drought and severe difficulty obtaining water, which affects sanitation access. Have residents complained to you about drought or other climate hazards?

Participant: During my time there, not particularly. They understand the situation. Even if they complain to me that the spring or its flow has decreased, they ultimately find a solution by pumping water from the river.

Interviewer: Have women complained that they previously did not have to collect water but now must travel farther to obtain it?

Participant: No. They do not carry water, and the source is not far.

Interviewer: So they rely entirely on pumps?

Participant: Yes.

Interviewer: Apart from Plan, does the government provide support to ensure access to water and STBM(Community Based Total Sanitation) services during climate disasters?

Participant: The government only provides recommendations, particularly from the district government to village governments. First, it urges communities to plant as many trees as possible, especially around water sources. Every year during this season, water scarcity becomes severe. Springs exist, but their flow is extremely low. The main problem is the absence of trees.

Interviewer: Has deforestation occurred there?

Participant: Yes. There is essentially no forest left.

Interviewer: So government support consists only of recommendations?

Participant: Yes.

Interviewer: Who provided the tree seedlings you mentioned? Was it KPH(Forest Management Unit)?

Participant: It was a collaboration between the local government and .

Interviewer: Does Plan also have a seedling-planting activity?

Participant: Hopefully it does.

Interviewer: So you have not yet received information about it?

Participant: Correct.

Interviewer: You only know about the activity from [local organization]?

Participant: Yes. We also discussed it at Bapemda (Village and Regency Governments) , on the 30th, if I remember correctly.

Interviewer: During the last two years you have been there, have climate hazards other than drought occurred, such as flooding or landslides?

Participant: No.

Interviewer: So only drought?

Participant: Yes, only drought.

Interviewer: Have you complained to the government about it? How do you coordinate with government regarding drought?

Participant: I complained to colleagues at the district level. The proposed solution was that when the rainy season approaches in December, communities should plant as many tree seedlings as possible. They recommended certain species, including fig trees, if I remember correctly. [local organization] also informed me that 2,000 seedlings would be prepared in the second week of November.

Interviewer: Who are the key individuals or community leaders with authority concerning water and STBM(Community Based Total Sanitation) services? Are there customary leaders?

Participant: No.

Interviewer: So responsibility rests entirely with the village government. At the community level, such as in the traditional house, have people discussed water and sanitation? Participant: Customary elders have discussed water. I shared my thoughts with them. I said, “During this season, we also struggle with water in [study village]. However, those of us in the upper area find ways to obtain clean water by digging wells.” People there do not use piped water. In my own experience, piped water becomes dirty during the rainy season. It is better to dig a well and obtain clean water. I advised community members that wells were appropriate there because well water is clean and clear. They did not want to do it because of the cost. I explained that a well cost only three to four million rupiah and could provide water for a lifetime, rather than collecting unsuitable water from a drainage channel where people bathe upstream.

Interviewer: So the village government remains the primary actor discussing water and sanitation access?

Participant: Yes.

Interviewer: During drought, how do vulnerable groups, particularly women and people with disabilities, obtain drinking water?

Participant: This affects everyone, including women and people with disabilities. Water is not actually very difficult to access, but people prefer not to make the effort, so they use pumps.

Interviewer: So the pumps make access easier because water flows directly into their homes?

Participant: Yes.

Interviewer: Do the pumps ever stop working?

Participant: No.

Interviewer: The key is having electricity credit?

Participant: Yes, that is the key. At the office, we purchased a large fiber tank, a hose, and almost 300 meters of electrical cable. The large black hose extends a long distance.

Interviewer: Do the hoses enter from behind the houses?

Participant: Yes.

Interviewer: Regarding climate disasters, how aware is the community of climate-change impacts?

Participant: The impacts are frightening. Their rice fields are now in very poor condition, and drinking water is also severely affected. Interviewer: I mean whether people feel fear, anxiety, or concern about these sudden climate changes, or whether they consider them normal.

Participant: What can they say? They have accepted the reality. They understand that they are not the only people affected.

Interviewer: Are there no preventive efforts for the future, such as planting trees? We must consider life beyond the present.

Participant: Nothing. They have not thought that far ahead.

Interviewer: So there are no traditional practices such as [local water-source protection tradition] to protect water sources?

Participant: No.

Interviewer: What concerns do community members, officials, or leaders have that might motivate them to reduce climate-disaster risks?

Participant: Based on my observations, the village also had a land-certification program. Many years ago, I traveled from garden to garden. The hills above are now completely bare. I thought, “In several years, what will happen in [study village]?” Heavy rainfall could cause landslides because the upper area is completely denuded.

Interviewer: Does the community have similar concerns?

Participant: I do not know. Personally, I believe something will certainly happen during the heavy rains in January or February.

Interviewer: I have asked several questions. Finally, is there anything else you would like to share, such as other achievements during your work with Plan, dissatisfaction with the program, or commitments and follow-up actions as Plan's program approaches its end?

Participant: I would like to thank Plan. Our full year of cooperation was good. Personally, the sanitation facility at the village office resulted from collaboration with Plan. Although I contributed ideas, I also sought Plan's experience and then implemented the improvements. From the bottom of my heart, I regret that our relationship with Plan must end. I would prefer to continue working together every year, but the decision belongs to Plan. That is my impression. I express my sincere thanks to Plan colleagues.

Interviewer: Several Plan programs have been implemented in [study village]. Is there one that you are committed to continuing without Plan's intervention?

Participant: Yes. One thing I cannot forget from Plan concerns community sanitation. I continue thinking about how to improve it. Although we are all human beings, regardless of being [Study Area 1] people, even if we eat a great deal, we still need a place for waste disposal.

Interviewer: So that is the program you are committed to sustaining?

Participant: Yes.

Interviewer: Thank you for your answers and ideas and for your time. Good afternoon.
