# Supplementary material for: Implementing a climate-resilient and inclusive WASH guidance in Indonesia: A mixed-methods case study
Source: PLoS One. 2026 Sep 28;21(9):e0359266. doi: 10.1371/journal.pone.0359266 (PMC13618932; doi:10.1371/journal.pone.0359266)
Supplement: Supplementary file 6 [file pone.0359266.s006.zip › S6 Appendix Transcripts/KII-12_Deidentified_English.docx]

Interviewer, Participant

Interviewer: Good morning. I apologize for disturbing you on a holiday. I previously explained the consent procedure and the purpose of the research. We can begin with the first question. This discussion has three parts: general questions about you, your experience and perceptions of inclusive and climate-resilient STBM(Community Based Total Sanitation), and a closing section. It will take approximately one hour. First, could you describe your role in the urban village and how long you have lived here?

Participant: Good morning, and thank you for the opportunity. I will first explain my role in the urban village. [a recently appointed urban village official]. I perform the duties assigned to me. Regarding STBM (Community Based Total Sanitation), I participated only once in my new urban village, and afterward there were no further STBM(Community Based Total Sanitation) activities. In my previous experience as a staff member at [former workplace], I frequently participated in STBM(Community Based Total Sanitation) activities. We visited communities directly to inspect handwashing facilities. Even when residents did not place them in front of their homes, we saw that they had bathrooms and at least some means of washing their hands. I also participated in an activity at [local secondary school] concerning water and sanitation and how students should maintain cleanliness.

Interviewer: So, in your new urban village, you have only participated in the climate-resilience activity, while for STBM(Community Based Total Sanitation) GEDSI(Gender Equality and Disability Social Inclusion) you had prior local-government and school experience. What impression did you have of STBM(Community Based Total Sanitation)?

Participant: I am very grateful for STBM(Community Based Total Sanitation). Government has many responsibilities and cannot examine every household in detail. I am grateful that another party cares about the community. These programs remind us about healthy lifestyles, beginning with handwashing and eliminating open defecation, followed by waste, water, food, and solid-waste management. We prepare proper waste bins so people do not contract diseases. I appreciate such activities. I hope support will continue even though Plan's STBM(Community Based Total Sanitation) program may have ended. I hope someone will continue supporting activities like these.

Interviewer: Thank you. You have considerable experience with inclusive STBM(Community Based Total Sanitation), even though you have been in the new urban village for only seven months.

Let us continue with your experience and perceptions of inclusive and climate-resilient STBM(Community Based Total Sanitation). Following Plan's intervention, has the urban village office modified or constructed any new climate-resilient STBM (Community Based Total Sanitation)infrastructure?

Participant: Several years ago, a damaged toilet at the urban village office was repaired. There are now two toilets. One is inside and is usually used by me or by visitors. Because I use it, it is often clean. The toilet at the back is used by the public and is also cleaned regularly. Regarding climate resilience, these toilets should be accessible to everyone, including women, children, and people with disabilities. When they come to the office for administrative matters, they should be able to use the facilities. Plan provided a support device,

Interviewer: Oh, a handrail.

Participant: Yes, a toilet handrail to facilitate access, particularly for people with disabilities.

Interviewer: Has it been installed?

Participant: Yes. In the future, people from both inside and outside the office can use it. That is one modification made at the urban village office. Regarding wastewater and water, facilities already existed from earlier periods. We still use plastic waste bins.

Interviewer: The handrail concerns inclusive STBM(Community Based Total Sanitation) because it makes access possible for everyone. Regarding climate resilience, are there measures related to water or sanitation facilities?

Participant: Regarding water facilities, communities currently rely mainly on the water utility and wells.

Interviewer: Is there climate mitigation, such as preparing storage tanks at the urban village office or for residents?

Participant: That is exactly what the community hopes for. They sometimes tell me, “Madam, even if water becomes available, there is no place to store it because we do not have money.”

Interviewer: A storage container?

Participant: Yes. I hope the new urban village may receive future assistance that directly reaches the community. In addition to occasional refresher sessions and facilities, we want STBM(Community Based Total Sanitation) to take direct action in the community.

Interviewer: Storage tanks are a mitigation measure. How do communities currently adapt to climate change, given the water shortages you mentioned?

Participant: We all experience the same problem. Water-utility service is sometimes reliable and sometimes unavailable. Some residents take the initiative to use wells. However, even when a well exists, it is less effective if there is no storage container.

Interviewer: When one neighbor has a well and another lacks water, can the second household obtain water from the neighbor?

Participant: Yes, that is common.

Interviewer: Thank you. I will refer to inclusive and climate-resilient STBM(Community Based Total Sanitation) as CCRIW. In your view, how can CCRIW influence the planning and implementation of water and STBM(Community Based Total Sanitation) programs in the urban village?

Participant: In my view, CCRIW has a positive influence because it supports mitigation. Although direct community implementation may not yet have occurred, plans and program designs are beginning to emerge. I hope it will have a strongly positive influence.

Interviewer: So, after the intervention, the head of urban village and urban village began considering plans and programs.

Participant: Yes.

Interviewer: Thank you. Compared with CCRIW, which Plan or other organizational activities have most influenced water and sanitation programs in the urban village?

Participant: I am still new here, so I do not fully understand other organizations' work on sanitation and water. However, Plan and STBM(Community Based Total Sanitation) have had a major influence. Communities are beginning to recognize that water scarcity is becoming difficult. I continually recommend reforestation and planting so that water can infiltrate the soil. At present, this is only advice because such activities require funding. Even organizing people for reforestation requires resources, especially when transportation is needed.

Interviewer: With whom does the urban village collaborate on reforestation?

Participant: Usually with community leaders and nearby residents. Recently, for example, PMI (Indonesian Red Cross Society) and MCC began planting in the lower area, although that is a different topic.

Interviewer: Has Plan collaborated with the urban village on reforestation?

Participant: As far as I know, Plan participated in planting at [former workplace]. I am new in this urban village and have not participated in such an activity here, but Plan joined tree planting at [former workplace].

Interviewer: Thank you. Does the urban village have funding to rehabilitate or construct climate-resilient water and sanitation facilities?

Participant: This is the difference between a urban village and a village. Villages have larger budgets and manage them independently. A kelurahan's budget is regulated through the subdistrict DPA (Budget Execution Document ). We must wait for the subdistrict to disburse funds, and as far as I know, no budget exists for

Interviewer: sanitation rehabilitation?

Participant: Correct, none.

Interviewer: Has there been none during your term, or also before it?

Participant: There has been none during my term.

Interviewer: When was the toilet last constructed?

Participant: It already existed before I arrived. Colleagues told me it had only recently been constructed when I began working here.

Interviewer: Was it funded through the subdistrict DPA (Budget Execution Document )?

Participant: I do not know. The previous official, who has since died, once told me he planned to construct a toilet. I asked where the funding would come from, and he said he would try to find it. By the time I arrived, it had been completed.

Interviewer: So Plan did not intervene to construct inclusive and climate-resilient sanitation infrastructure, perhaps because of the budget issue you described. In villages, Plan interventions encouraged construction of sanitation facilities, and then…

Participant: Villages could construct them themselves.

Interviewer: Yes. That may be the difference. Nevertheless, your toilet has a handrail and has been modified. Thank you. In your view, is CCRIW suitable for implementation at the urban village level?

Participant: I think it is very suitable.

Interviewer: On a scale from one to five, where one means dissatisfied and five means satisfied, how would you rate CCRIW?

Participant: I would select five.

Interviewer: Very satisfied.

Participant: Yes, because I hope it continues. Implementation is still gradual rather than comprehensive. I hope it will continue so that, in the future, assistance with water and sanitation will not merely provide facilities but will also help us think about how to improve community health.

Interviewer: Could you describe your household monitoring after Plan's intervention concerning the five STBM(Community Based Total Sanitation) pillars?

Participant: In the new urban village, I joined a Plan activity in which we collected data. We asked whether each household had water, identified its water source, completed the required fields, and asked whether it had a toilet. I am grateful that most households in the new urban village have water-utility connections, although perhaps a few lack meters. The constraint is that water sometimes flows and sometimes does not.

Interviewer: Water availability?

Participant: Yes, water availability.

Interviewer: But the facilities exist?

Participant: Some households also use wells. The continuing problem is that they lack storage containers. During the rainy season this is less serious because water-utility service is more reliable, but the dry season is difficult.

Interviewer: Villages can adopt CCRIW by constructing inclusive and climate-resilient sanitation facilities, but urban village face the budget constraints you described. Is there another strategy that could achieve inclusive and climate-resilient STBM(Community Based Total Sanitation) in the new urban village?

Participant: When residents complain, I respond, but I do not answer immediately. I coordinate with higher levels and with community leaders or other residents who may help. We have considered forming groups to construct storage tanks. I suggested collaborating with government and submitting a proposal to the public works office so that water tanks might be constructed. The same applies to waste bins. It would be useful to construct a permanent waste container in front of the office. In large cities such as Surabaya, many houses have permanent waste containers in front. Residents place their waste there, and a vehicle collects it, preventing scattered waste and disease. I have also considered water issues. I work directly with communities and always try possible solutions before admitting that something cannot be done.

Interviewer: That is excellent. If the urban village adopts CCRIW, what steps would you take to ensure success? Three pillars remain incomplete.

Participant: The first step is…

Interviewer: Is additional training or guidance needed, or should the program be integrated with other programs?

Participant: Training is needed first. It provides knowledge, guidance, and education about how to use, maintain, and protect facilities. Providing assistance is pointless if the facilities are not maintained and quickly become damaged. I would be pleased to participate in training first. After guidance, implementation can begin. We should not take action without first having sufficient knowledge.

Interviewer: Was the modified handrail installed only at the urban village office, or did nearby residents also modify facilities following Plan's intervention?

Participant: When the disability-access handrail was installed, community leaders were invited. It was not limited to urban village staff. Information was provided through a form of socialization. Several houses already have similar features, but not all do, because many facilities remain temporary. [laughs]

Interviewer: Correct. So the urban village has no funding to construct sanitation facilities such as toilets?

Participant: There is no funding.

Interviewer: I see.

Participant: The budget process works like this. Sometimes proposals have already been prepared [NOISE] (23:00). I am new in this position, while proposals were submitted last year, so I must follow the existing plan.

Interviewer: So a new proposal could be made for next year?

Participant: We can try to submit a proposal for next year. Hopefully it will be approved. We will seek cross-sector coordination with relevant agencies. The disaster-management agency may be able to assist in some form, while the public works office might provide cement or other materials. We will try to coordinate.

Interviewer: Plan's program is approaching its end, so sustainability is expected. Without Plan, will the urban village government intervene to ensure program continuity? What does the urban village plan to do?

Participant: The urban village often intervenes directly with communities, but at present this is limited to cleanliness messages and moral support. We have not yet provided funding. In the future, I will continue considering what infrastructure must be developed.

Interviewer: Is this expected to become part of your 2025 planning?

Participant: Yes.

Interviewer: Thank you.

Participant: We would submit the proposal in 2025, so implementation might occur in 2026. The 2024 proposals already concern 2025. Perhaps we can implement one item each year, gradually. At least we should begin rather than do nothing.

Interviewer: We will now discuss enabling and inhibiting factors affecting CCRIW results. Does the urban village have an organizational structure for water and sanitation management?

Participant: Health personnel usually handle it here.

Interviewer: Does that include the STBM(Community Based Total Sanitation) team?

Participant: Yes, it comes from the health sector.

Interviewer: What about a urban village STBM(Community Based Total Sanitation) team?

Participant: Within the urban village, we mainly use community groups.

Interviewer: What are their roles and responsibilities?

Participant: At present, they mainly convey complaints, for example, “Madam, the storage tank is dry, and water is difficult to obtain.” Their work is not yet fully organized because it requires support from many parties. We cannot work alone. For now, we proceed with what is available.

Interviewer: Do the water-management group and STBM(Community Based Total Sanitation) team include marginalized groups?

Participant: Yes. As far as I know, a meeting at the subdistrict once encouraged attention to sanitation and these groups. In the new urban village, the groups may need to be strengthened. We face many constraints. Even when we ask people for help, they may have more urgent personal responsibilities.

Interviewer: It is complicated.

Participant: Yes. Most work here depends on whether people can afford to participate.

Interviewer: It also depends on human resources.

Participant: Yes, and many members work as teachers. They leave in the morning, return in the afternoon, and are tired. They may be available only on holidays.

Interviewer: We are facing climate change. How does the community respond to severe drought?

Participant: There are many responses. People say, “Madam, why is it so dry and hot? Perhaps the water is disappearing.” I advise them to plant more vegetation because plants help retain water and support groundwater. Reforestation does not require traveling elsewhere. It can occur around people's homes. Plants produce clean air and oxygen, reduce dust in hot areas, and help water infiltrate the soil.

Interviewer: You said water availability was sufficient. Have people ever defecated at the coast to conserve water because no water was available, or is water for sanitation still adequate?

Participant: I have visited several places, although I prefer not to name them. Some areas are informal and vulnerable settlements. It appears that residents may be reluctant to construct toilets.

Interviewer: Are they in a steep area?

Participant: Yes, uphill. The area smells when we go there. The situation is difficult. Similar conditions also exist beside the river, perhaps because of water constraints. Residents often respond by asking whether we will provide assistance.

Interviewer: They have become accustomed to requesting assistance?

Participant: I tell them that this concerns their own health. Others complain about private companies and say that if a company had previously been established there, their livelihoods would be secure.

Interviewer: They blame others.

Participant: Yes. I tell them the past cannot be changed, so we must find other solutions. I advise them to construct small infiltration channels around their houses so that their wells do not dry out.

Interviewer: Has extremely limited water availability caused illness?

Participant: Not broadly, as far as I know. It depends on whether individuals maintain their health. Problems may occur in a few locations rather than throughout the urban village. We cannot directly intervene in every household. If someone has stomach pain, for example, they are taken to the health center.

Interviewer: In a urban village, the health center usually participates in the urban villageSTBM(Community Based Total Sanitation) team, and there may also be a water-management group. Is there an OPAM( Community based water system) here, or is it called something else?

Participant: There is no OPAM( Community based water system) here.

Interviewer: So the water utility manages the water system?

Participant: The water utility manages its network, but other water sources are currently managed privately by households.

Interviewer: So there is no organization.

Participant: There is no specialized organization or designated person paid to manage water.

Interviewer: None?

Participant: None.

Interviewer: So there is only a urban village STBM(Community Based Total Sanitation) team.

Participant: Yes, but its members work voluntarily. Regarding water, we simply wait for the seasons. During the dry season, water becomes scarce, and when rain returns the problem decreases.

Interviewer: During drought, have sanitation facilities or pipes been damaged? Where do residents report damaged facilities?

Participant: I encourage residents not to bring every problem directly to the urban village. They should first address it at the RT level. If the RT cannot resolve it, they can bring it here. If we also cannot resolve it, we identify the next responsible institution. We first try to address matters within the community.

Interviewer: How is the STBM(Community Based Total Sanitation) team involved in water and sanitation?

Participant: In the new urban village, there have not been many complaints. Occasionally people say that water has dried up, but everyone experiences the same shortage. Their primary request is for storage tanks. I ask households how long their well water lasts. They say that even if a well dries up, a storage tank would retain water for later use. I tell them that is correct.

Interviewer: When you conducted monitoring visits, did you go with the STBM(Community Based Total Sanitation) team?

Participant: Yes.

Interviewer: With colleagues?

Participant: With colleagues from the health center.

Interviewer: Is the STBM(Community Based Total Sanitation) team based in the urban village or the health center?

Participant: The health center. Its staff are the most active.

Interviewer: So the people working as the urban village STBM(Community Based Total Sanitation) team are actually from the health center?

Participant: Yes.

Interviewer: Are there no members from the urban village?

Participant: Kelurahan members participate voluntarily. We have youth organizations, community self-help organizations, and disaster-preparedness groups that can be involved. These organizations were formed some time ago.

Interviewer: Is the disaster-preparedness group affiliated with the Red Cross?

Participant: Yes. I usually ask those groups to assist with water-related matters because we do not yet have a formally structured and funded organization. My concern is that…

Interviewer: We use the available human resources.

Participant: If we formally establish such a team through a decree, funding must be available. That is the problem.

Interviewer: So the urban village STBM(Community Based Total Sanitation) team…

Participant: is drawn from…

Interviewer: It does not yet have a formal decree and consists primarily of health-center staff.

Participant: Yes, from the health center.

Interviewer: I understand. Let us continue to institutional support. The urban village government cannot work alone and requires support from government and private institutions. How have these relationships helped ensure good water and sanitation access, particularly for marginalized groups? What support have you received?

Participant: From the community?

Interviewer: No, from government agencies, regional organizations, Plan, or other non governmental organizations.

Participant: Government support has focused on infrastructure. Water-storage tanks and waste facilities have not yet been provided, but many drainage channels have been constructed to manage floodwater.

Interviewer: Yes.

Participant: These were developed through collaboration with government, including the public works office and other agencies responsible for infrastructure.

Interviewer: Yes.

Participant: Such support exists, although it has not reached every area. Implementation must be gradual.

Interviewer: So institutional collaboration has produced support and results. During your last seven months in office, have you received support from the public works office, or did it occur under previous programs?

Participant: Not during the last seven months. However, earlier support is evident in the drainage channel in front of the office.

Interviewer: Have you coordinated with government officials regarding water and STBM(Community Based Total Sanitation) issues in the urban village?

Participant: We usually discuss water and drought with health-center staff. They then communicate the issues to higher levels, including the Health Office, through cross-sector coordination. We can also take the initiative. However, annual development plans have already been determined. Perhaps in 2025, if there is a revised budget, we can coordinate again.

Interviewer: In [study city], there are traditional community houses called gendang, correct?

Participant: Yes.

Interviewer: Does the new urban village have an individual or community leader who plays a key role in water matters? For example, a gendang has a leader who discusses water. What is the situation here?

Participant: There is no gendang here because most residents are migrants. Some are [study city] people, but the new urban village has no gendang.

Interviewer: Apart from the urban village government, who plays a key role in water issues?

Participant: When the urban village government faces a problem, we deliberate and invite community leaders.

Interviewer: Community leaders.

Participant: Yes, including religious leaders. We invite them to discuss the issue.

Interviewer: Have you discussed water issues with religious and community leaders?

Participant: The topic was mentioned recently, but only briefly and not as seriously as in this discussion. The effects of drought are only now becoming strongly apparent. People are still considering the issue individually. I hope future activities can continue because water sources should not be taken lightly. We cannot live without them.

Interviewer: Even in [study city]. Could you explain specific challenges faced by vulnerable groups, such as people with disabilities, women, and older adults, when climate change reduces water availability and creates difficulties accessing water and sanitation?

Participant: The greatest responsibility still rests with their families. We can only observe their living conditions.

Interviewer: Have women had to carry water containers while also carrying children?

Participant: Not at present. This is an urban area. Such situations may be common in villages, but the city still has water-utility service. Although it is sometimes interrupted, some water remains available. Children and people with disabilities are vulnerable depending on availability. I hope we will prioritize them by providing facilities.

Interviewer: Do cultural practices or norms affect these challenges? For example, do people regard people with disabilities as deserving sympathy and assistance? How does the community view vulnerable groups?

Participant: Community members generally feel sympathy. Some people with disabilities accept assistance, while others do not because they feel capable. The impulse to help exists. People say that it is not merely pity but an expression of faith and compassion.

Interviewer: Regarding knowledge and capacity, how aware is the community of climate-change impacts?

Participant: At present, they continue using the available facilities.

Interviewer: So they can adapt?

Participant: Yes, they can adapt, although they still complain and ask for more reliable water availability and facilities.

Interviewer: Are there traditional practices used to prevent or address water scarcity? In our area, for example, [local water-source protection tradition] is conducted annually to help maintain spring flow.

Participant: In customary terms…

Interviewer: What traditional practices exist here?

Participant: As I said, there is no gendang. Therefore, such practices do not currently exist here.

Interviewer: People only adapt?

Participant: Yes, they only adapt. It would be different if a gendang existed. I have seen people conduct torok rituals at water sources.

Interviewer: Yes.

Participant: Although I am not originally from [study city] and come from [another island/region], I have participated in such customary rituals. People ask their ancestors and the land to provide water.

Interviewer: But that does not occur here?

Participant: No, because there is no gendang and many residents are migrants rather than [study city] people.

Interviewer: Psychologically, how concerned is the community about climate change?

Participant: Many people are concerned and ask what will happen if the situation continues. I tell them that infrastructure is one matter, but the water source itself is the critical issue. We need both effort and prayer. Sometimes God provides unexpected opportunities, but we should still act.

Interviewer: Those were my questions. Is there anything else you would like to share, perhaps from your experience in the previous urban village concerning planning, implementation, and monitoring of inclusive and climate-resilient STBM(Community Based Total Sanitation)?

Participant: Planning usually involved the urban village and all community groups. Community leaders, people with disabilities, older adults, and women were invited to discuss proposed activities. They were also involved during implementation, even if their capacities differed. In the new urban village, I only encountered the final stage of the process.

Interviewer: During your time here, have you conducted household monitoring concerning STBM(Community Based Total Sanitation)?

Participant: Yes.

Interviewer: What were the results?

Participant: The results were reasonably positive. Communities are becoming aware. They wash their hands before eating and after handling waste. I do not always ask directly. During a household visit, I may say, “You have mangoes here. Let us eat one.” The resident then says, “Yes, madam, but let us wash our hands first.”

Interviewer: So the intervention produced results.

Participant: Yes. I do not usually speak directly.

Interviewer: You use diplomatic language.

Participant: I analyze people's character and use my own approach rather than directly reprimanding them. I encourage them to understand and follow the desired behavior while I observe whether they actually practice it.

Interviewer: I think that is sufficient. Do you have another story?

Participant: Regarding STBM(Community Based Total Sanitation), health, and handwashing, my continuing hope concerns infrastructure. That is the key. Even when a well exists, one source may serve several families. A storage tank would allow them to store abundant water for use when the well or utility supply becomes dry or stops temporarily.

Interviewer: Thank you for sharing your experience today and for giving your time on a holiday. I will end the interview.
