# Supplementary material for: Implementing a climate-resilient and inclusive WASH guidance in Indonesia: A mixed-methods case study
Source: PLoS One. 2026 Sep 28;21(9):e0359266. doi: 10.1371/journal.pone.0359266 (PMC13618932; doi:10.1371/journal.pone.0359266)
Supplement: Supplementary file 6 [file pone.0359266.s006.zip › S6 Appendix Transcripts/KII-13_Deidentified_English.docx]

Interviewer, Participant

INTERVIEW NOTES

Interviewer: All right, [village official]. Thank you for your time. This interview has three parts: general questions about you, your experience and perceptions of inclusive and climate-resilient STBM(Community Based Total Sanitation), and a closing section. First, could you describe your role in the village and how long you have lived here?

Participant: In the village, I develop policies intended to help communities, particularly with water scarcity. Water is my priority and was one of my campaign promises. Every year, the largest allocation is for water, including efforts to protect water sources.

Interviewer: How long have you lived here?

Participant: For approximately fifty years, since birth.

Interviewer: Have you heard of and participated in inclusive and climate-resilient STBM(Community Based Total Sanitation)?

Participant: Yes. I participated in my capacity as village head and invited residents to hear an explanation of STBM(Community Based Total Sanitation) and climate change. It was [program staff member]'s last activity in the village.

Interviewer: What was your impression of the workshop?

Participant: It was a campaign to address global warming through small actions, particularly water-source conservation. Communities were made aware that water is essential. People often consume water continuously without considering how to preserve it for their children and grandchildren. The workshop promoted actions such as planting trees at water sources and using water appropriately. The response was positive because water was already my policy priority as a leader. This concerns not only delivering water to the community but also ensuring that water remains available by protecting its source.

Interviewer: Excellent. It was aligned with your vision and mission.

Participant: Yes, the program was aligned with my priorities.

Interviewer: Following the workshop, did you modify or construct any household water and sanitation facilities to make them more climate resilient?

Participant: Yes, but only small measures. We purchased water-storage containers because the water system here uses pumps.

Interviewer: From boreholes?

Participant: No. Water comes from a spring and is pumped to the settlement using machinery. This requires funding, and water does not flow continuously. It is pumped at scheduled times, so we prepare containers to store it for use. We have permanent indoor and outdoor bathing, washing, and toilet facilities, as well as a storage tank for handwashing.

Interviewer: Does each household pump water, or is it a communal system?

Participant: There are still two large communal points. Village funds have not yet financed household connections. My long-term goal and political promise is to complete household connections and install meters. Once all household connections are completed, we will improve drinking-water services until each household has a tap.

Interviewer: [laughs] How have you modified sanitation facilities such as toilets to make them resilient to disasters?

Participant: The household facilities are all permanent. They do not treat wastewater for reuse. They use a single septic-tank system constructed from masonry. Waste is collected there without further treatment, but the facilities are complete.

Interviewer: Have the septic tanks ever overflowed?

Participant: No, never.

Interviewer: Have floods or landslides occurred here?

Participant: No.

Interviewer: So the facilities are resilient to local disasters, and mitigation focuses only on drought.

Participant: Yes, drought. It occurs periodically, and the dry season is necessarily dry.

Interviewer: But you have already implemented mitigation?

Participant: Yes, through the measures I described.

Interviewer: Through storage containers.

Participant: Yes, by providing storage.

Interviewer: Thank you. So drought is the only common extreme-weather event. During drought, how do the water and sanitation facilities perform? Do they remain safe, or are any damaged?

Participant: The water flow decreases. The facilities are generally not damaged. Minor damage sometimes occurs, but workers responsible for the system can usually repair it.

Interviewer: Are there specific design features that help maintain water services and the five STBM(Community Based Total Sanitation) pillars in this dry area? For example, flood-prone areas might elevate drainage channels or construct embankments. What is needed for drought?

Participant: We still use water-sealed toilets. They require considerable water, but the issue is how we manage water so that enough remains for those needs.

Interviewer: Thank you. That concerns your household. How do nearby community members address the challenge? Do they use the same measures?

Participant: Not everyone has a large fiber tank. Most use water drums or large jerry cans stored in their homes. The important point is that they have some form of storage.

Interviewer: Is water currently sufficient, or has the flow declined while still meeting needs?

Participant: It still meets needs.

Interviewer: Do residents complain that water and sanitation are difficult to access?

Participant: Communities naturally want better services. We must construct the system gradually according to village finances. We would prefer to complete everything at once, but village finances do not allow that. Nevertheless, water remains our focus.

Interviewer: Does the village have a water-management organization and an STBM(Community Based Total Sanitation) team?

Participant: Yes.

Interviewer: What are their roles and responsibilities?

Participant: The water team works every day because the system is pump-based. They ensure that water is available in the storage basin at the spring. They ensure that the pump and pipes are not damaged so water can be supplied. They also ensure that water flows from the communal points. This is OPAM's(Community Based Water System) work. Regarding salaries or incentives, their work is primarily voluntary participation.

Interviewer: If a pipe or another water or sanitation facility is damaged, who is responsible?

Participant: The village government is ultimately responsible, but OPAM( Community based water system) performs the work. OPAM( Community based water system) ensures that pipes remain functional and carry water to the storage basin. If members can repair damage themselves, they do so. If the repair exceeds their capacity, they report it to the village.

Interviewer: Are marginalized groups involved in OPAM( Community based water system) or the village STBM(Community Based Total Sanitation) team?

Participant: Not at present.

Interviewer: Are they invited to meetings or decisions?

Participant: Yes, they are invited.

Interviewer: They are invited. Is there representation from any particular group?

Participant: There is a representative of people with disabilities.

Interviewer: Are you involved in the water-management group and STBM(Community Based Total Sanitation) team?

Participant: As village head, I am always involved. In any hamlet, when the issue concerns water, I am present. Whether assistance is planned or urgent, I am involved. If a pump has even minor damage and funding is not yet available, I find my own way to help when they face difficulty.

Interviewer: The village now faces drought as a climate hazard. How does the community respond, particularly regarding access to water and sanitation?

Participant: Every year, communities automatically adjust according to the length of the dry season and the timing of rain. However, impacts remain. Spring flow decreases significantly during the dry season. Shortages can also occur when the storage basin at the spring leaks and water cannot be retained, which affects overall availability.

Interviewer: Under those conditions, can the community only adapt?

Participant: The community is proactive, particularly in paying water fees. The fee is divided among several purposes. Because electricity is not yet available here, other villages use prepaid electricity credit. The fee also covers management incentives and depreciation or replacement of equipment, so funds are available if a pump breaks or a pipe is severed. All operating costs are added together and divided among beneficiary households in the settlement. The community determines the fee through deliberation. Members are highly active in those discussions.

Interviewer: The involvement you described concerns technical matters such as fees. How does the community communicate and propose solutions to water and sanitation problems?

Participant: To protect springs from climate hazards, this year we planted trees in collaboration with the diocese. We planted at the spring and regulated access to the spring area. The area is legally protected because a systematic land-registration activity was conducted here. People cannot enter or use the area arbitrarily because the boundary pillars have been installed and monitored. Moving a marker can lead to legal consequences. Land outside the marked boundary is no longer privately controlled. We will also construct a small fence around the spring.

Interviewer: Has another government agency or private party supported this work?

Participant: Yes, the diocese supported it. The pumping system was also financed through an earlier Special Allocation Fund grant that we managed properly. The original budget included several large fiber tanks. We considered that tanks would deteriorate when left empty, so we constructed ten permanent basins with equivalent storage capacity instead.

Interviewer: […] Would you like to rest?

Participant: Let us continue.

Interviewer: The next topic is the division of labor. How does climate change affect older men compared with older women?

Participant: Climate change clearly reduces water availability and flow. Women bear more of the workload, approximately 65 percent compared with 35 percent for men.

Interviewer: Does that also apply specifically to older adults?

Participant: Older adults. People around our age, below 50… Are older adults defined as 45 and above?

Interviewer: Usually 50 and above.

Participant: Yes, above 50. The situation is the same for men and women.

Interviewer: Is the distribution still approximately 65:35?

Participant: Yes.

Interviewer: Who decides whether to modify water and sanitation facilities, the husband, the wife, or both?

Participant: Decision-making is approximately 40 percent women and 60 percent men.

Interviewer: Why is that?

Participant: I am not certain. Male dominance remains relatively strong.

Interviewer: Have you participated in gender equality and inclusion activities, and what did you learn?

Participant: The training made us aware that no work must be done only by women, except biological functions such as breastfeeding and giving birth. Other work can be performed jointly. That was my understanding.

Interviewer: What good gender-equality practices now occur in your household? Do you help wash dishes, for example?

Participant: I even cook. If my wife is unwell or very busy, I perform any work I can do.

Interviewer: Did this result from Plan's intervention, or did it already occur?

Participant: It occurred before, but not as much. After receiving Plan's training, my willingness increased.

Interviewer: Are you embarrassed by people who comment that the village head is washing dishes?

Participant: People do make such comments. I respond that if we can do the work, there is no reason not to do it.

Interviewer: Thank you. How are vulnerable groups involved in accessing water and sanitation in [study village]?

Participant: Everyone receives the same treatment, including people with disabilities and other vulnerable groups. They do not receive special treatment, but we are gradually bringing services closer to them.

Interviewer: During drought, have people with disabilities needed to use toilets when insufficient water was available? Has anyone complained to you?

Participant: They have not complained. People with disabilities usually do not live alone and have family members who assist them. Families are accustomed to providing that support.

Interviewer: Do neighbors support one another during drought or disasters? Elsewhere, households without boreholes may obtain water from neighbors who have them. What happens here?

Participant: If a household lacks stored water, it can borrow water. Before the pumping system existed, a household without water often borrowed several jerry cans from a neighbor and returned the same amount later. Since the pumping system began operating without major breakdowns, that practice has gradually disappeared because every household now has sufficient water.

Interviewer: Conditions have improved substantially. Do you know of local wisdom that helps reduce climate-hazard risks?

Participant: Every year, we hold a [local customary ritual] celebration. It expresses gratitude to our ancestors and God for what we have received and asks for blessings for the new year and planting season. It includes the [local customary ritual] rite and is conducted every year without exception.

Interviewer: What does the [local customary ritual] involve, and how is it related to reducing climate-hazard risk?

Participant: We ask our ancestors to ensure that water continues flowing and does not disappear, so it remains sufficient for residents' needs. That is the ritual's intention.

Interviewer: Are there other traditional practices that help address climate-disaster impacts, apart from the local wisdom you described?

Participant: No.

Interviewer: On a scale from one to five, where one means no effect and five means a very severe effect, how strongly do climate disasters currently threaten your access to water and STBM(Community Based Total Sanitation) services?

Participant: You mean the level of risk?

Interviewer: Yes. One means no effect, two means some effect, three is neutral, four means an effect, and five means a severe effect.

Participant: I would select two, meaning there is some effect.

Interviewer: Could you describe a personal experience in which a climate disaster affected your access to water and STBM(Community Based Total Sanitation) services?

Participant: Our water system has a storage basin. When water is abundant and leakage is small, water still accumulates. When the flow declines and a leak is not repaired, water is continually lost until none remains in storage. This creates a short-term water shortage until repairs are completed.

Interviewer: Was there a specific challenge in that experience?

Participant: Do you mean the community's role or my own?

Interviewer: At the household level.

Participant: At the household level, the challenge was not severe.

Interviewer: How do past and current conditions compare?

Participant: Water access is now better and reasonably good.

Interviewer: How long has it been better?

Participant: For approximately 12 years.

Interviewer: Finally, what concerns might motivate you to take action to reduce climate risks? We have discussed climate hazards. What worries you about what might happen, and what actions or plans do you envision?

Participant: My concern is that one day water may stop emerging from the spring. If the spring stops flowing, where will we obtain water? We have acted by regulating land ownership around the spring. This protection is permanent. Boundary markers have been installed, so no one can disturb the area. If anyone moves a marker, we can involve law-enforcement authorities. Community members respect the area. Since the land was surveyed and marked, they have not interfered with it.

Interviewer: Was Plan involved in climate-resilience activities only through the workshop, or were there other activities such as tree planting?

Participant: We discussed tree planting, but no action has occurred. At the village office, Plan donated one toilet bowl. The office toilet was being relocated, and Plan noted that it could not function without a toilet bowl. Plan provided one temporarily, and we installed the water-sealed toilet and an access handrail.

Interviewer: Is the facility inclusive?

Participant: No, it is a standard facility.

Interviewer: Does your household toilet have modifications such as handrails?

Participant: No, everything is standard.

Interviewer: So not yet.

Participant: Because they are not yet needed.

Interviewer: But you understand the concept and have knowledge about it?

Participant: Yes.

Interviewer: Those are my questions. Is there anything else you would like to share regarding Plan, its programs, or the community's response?

Participant: I found one important issue concerning spring conservation. I understand that, under state regulations, land within 200 meters of a spring cannot be privately owned. As a leader, I may have appeared to threaten people, but it was for the common good. I said, “If you do not allow me to survey the land we need, I will apply the full 200-meter rule, and all of this land will become state property.” That was the approach I used. The community complied partly from awareness and partly because state law remains binding once written and applied.

Interviewer: I forgot to ask about handwashing practices at your home.

Participant: We installed a tap near the septic-tank area. It does not currently have water, but another facility below it always has water.

Interviewer: What about the wastewater channel? Is there a dedicated drainage channel, or does the water flow into an open area?

Interviewer: There is an outlet from the washing area to the outside of the house. The water infiltrates there because this is not a waterlogged area. Pumpkins planted there grow well.

Interviewer: How are food and drinking water managed? Does water need to be boiled?

Participant: Water must be boiled.

Interviewer: It must be boiled?

Participant: Yes, because the lime content is very high.

Interviewer: Is food usually covered?

Participant: Yes. Water drums and tanks are also covered. Nothing is left open.

Interviewer: How is solid waste managed? Is it burned?

Participant: We place it in used drums and burn it when the drums are full. There is one in front and one behind the house. Kitchen waste goes into the rear drum, while waste from the front goes into the front drum.

Interviewer: So the waste is separated?

Participant: No. It is disposed of together.

Interviewer: Kitchen waste goes behind the house, and everything is burned?

Participant: Yes. It is burned when dry.

Interviewer: Thank you very much for your time. I apologize for disturbing you on a holiday.

Participant: You are welcome.
