# Supplementary material for: Implementing a climate-resilient and inclusive WASH guidance in Indonesia: A mixed-methods case study
Source: PLoS One. 2026 Sep 28;21(9):e0359266. doi: 10.1371/journal.pone.0359266 (PMC13618932; doi:10.1371/journal.pone.0359266)
Supplement: Supplementary file 6 [file pone.0359266.s006.zip › S6 Appendix Transcripts/KII-14_Deidentified_English.docx]

Interviewer and participant labels standardized

INTERVIEW NOTES

Interviewer: Hello, good evening.

Participant: Good evening, madam.

Interviewer: Hello, [participant]. Good evening.

Participant: Good evening.

Interviewer: I apologize for disturbing you.

Participant: Yes.

Interviewer: Can you hear me clearly?

Participant: Yes, madam.

Interviewer: As I explained earlier, I will send the interview consent form afterward. The important point is that I have already asked for your permission.

Participant: Yes, madam.

Interviewer: Can we begin with the questions?

Participant: Yes, I am ready.

Interviewer: What is your current daily occupation?

Participant: I am a homemaker.

Interviewer: Excellent.

Interviewer: What is your role with Plan and in the village?

Participant: I am an STBM(Community Based Total Sanitation) cadre.

Interviewer: How long have you been a village STBM(Community Based Total Sanitation) cadre?

Participant: I have been an STBM cadre for several years.(Community Based Total Sanitation)

Interviewer: You are very experienced with the five STBM(Community Based Total Sanitation) pillars.

Participant: Yes. [laughs]

Interviewer: Could you describe your village role and your experience participating in workshops or socialization about the five STBM(Community Based Total Sanitation) GEDSI(Gender Equality and Disability Social Inclusion) pillars and climate resilience?

Participant: I have participated in STBM(Community Based Total Sanitation) GEDSI(Gender Equality and Disability Social Inclusion) activities for a long time, but I have rarely joined inclusive STBM activities.

Interviewer: Previously, you participated mainly in STBM GEDSI(Gender Equality and Disability Social Inclusion). What about climate resilience?

Participant: I participated once.

Interviewer: Could you explain your roles in STBM GEDSI(Gender Equality and Disability Social Inclusion) and climate-resilient STBM activities and what you learned?

Participant: First, I thank Plan for trusting me as an STBM cadre. I gained considerable knowledge from STBM activities. We first mapped the community in relation to the five STBM GEDSI(Gender Equality and Disability Social Inclusion) pillars. We marked hamlet boundaries and identified the locations of bathing, washing, and toilet facilities and waste-disposal sites in my village.

Interviewer: Excellent. You still remember it. [laughs]

Participant: I remember a little.

Interviewer: For how many years were you highly active?

Participant: I participated in STBM GEDSI for several years.(Gender Equality and Disability Social Inclusion)

Interviewer: Was socialization conducted every year or at another interval?

Participant: We participated in GEDSI(Gender Equality and Disability Social Inclusion) activities every year.

Interviewer: Did you conduct socialization only in the village, or were you invited to other villages?

Participant: Plan invited me to activities in [study subdistrict/city]. We conducted triggering training in [study subdistrict/city] and [study subdistrict] during earlier program years.

Interviewer: Afterward, you practiced the approach in [study village].

Participant: Yes, in [study village], my own village.

Interviewer: After working in the village, were you invited to socialization activities elsewhere?

Participant: I did not join socialization elsewhere, but village and subdistrict colleagues frequently asked us to conduct monitoring and evaluation in other villages.

Interviewer: In which village?

Participant: Most recently, I worked in [neighboring village], which is next to my village.

Interviewer: What did you do during monitoring and evaluation?

Participant: We inspected toilets, bathing and washing facilities, and clean drinking-water facilities in residents' homes.

Interviewer: What did you need to verify regarding toilets and water?

Participant: We verified that toilets were in good condition and usable by everyone, including older adults and households with members with disabilities. We also examined handwashing and clean drinking-water facilities.

Interviewer: Excellent. So all five pillars were evaluated.

Participant: Yes, we conducted the evaluation.

Interviewer: During your monitoring, did you encounter facilities that did not meet the five-pillar standards?

Participant: At first, many did not meet the standards. Thank God, conditions improved from year to year because village funds are now focused on community infrastructure, including clean drinking water and bathing, washing, and toilet facilities.

Interviewer: Thank you for sharing your experience. You have described STBM GEDSI(Gender Equality and Disability Social Inclusion). What about climate resilience?

Participant: Regarding climate resilience, we conducted…

Interviewer: Have you participated?

Participant: Yes, but only once. We conducted socialization with the community about adapting to climate change.

Interviewer: Did you participate only once because Plan conducted only one socialization activity, or because you were given only one opportunity?

Participant: I heard that Plan conducted inclusive STBM(Community Based Total Sanitation) activities approximately every three months, but I did not have opportunities to participate.

Interviewer: During the one socialization activity you attended, what useful information did you receive?

Participant: We learned about adapting to climate change. During dry periods, we must accept current climate conditions and prepare through mitigation. For example, we should prepare larger water-storage containers so more water is available during the dry season. Thank God, however, [study village] has not experienced drought during the last five years since Plan arrived. I am very grateful to Plan because the community no longer experiences drought.

Interviewer: That is interesting. What intervention did Plan implement so that [study village] no longer has difficulty obtaining water during drought?

Participant: Plan provided many facilities in [study village]. First, it constructed bathing, washing, and toilet facilities at the school and one at the auxiliary health post. It also provided large water-storage facilities. For inclusive STBM, Plan constructed a bathing, washing, and toilet facility specifically for a household with a member with a disability.

Interviewer: Thank you. You have participated in STBM GEDSI(Gender Equality and Disability Social Inclusion), inclusive STBM(Community Based Total Sanitation), and climate-resilient STBM(Community Based Total Sanitation) and have described good practices. Following the socialization, did you apply similar practices at home? What did you modify regarding water and sanitation?

Participant: Yes, certainly. Before conveying information to the community, I must first practice it at home. Otherwise, community members may refuse to act because I do not follow my own advice.

Interviewer: That is the mark of a true cadre. What did you do at home?

Participant: The path to my toilet used to be slippery. I spread sand on it so family members would not slip while going to the toilet during the rainy season.

Interviewer: Excellent. Regarding climate change, what extreme weather commonly occurs in your area?

Participant: Recently, landslides during the rainy season and strong winds have been the main extreme events.

Interviewer: When did the most recent landslide occur?

Participant: Last February.

Interviewer: In February during the rainy season.

Participant: Yes.

Interviewer: What effect did the landslide have on access to water and sanitation?

Participant: The impacts are clear. Water becomes turbid and sometimes cannot flow because dirt enters the storage basin and blocks it.

Interviewer: Are any pipes damaged?

Participant: Yes, some are automatically damaged.

Interviewer: When damage occurs, to whom is it reported, the STBM(Community Based Total Sanitation) cadre or another party?

Participant: Thank God, the village now has an OPAM(Community Based Water System) with designated staff who inspect damaged pipes and water-storage facilities.

Interviewer: What did STBM(Community Based Total Sanitation) cadres do during a climate disaster, such as the recent landslide?

Participant: STBM(Community Based Total Sanitation) cadres monitored conditions and informed the community about preparing for possible landslides. Regarding handwashing, when tap water was unavailable, we used water stored in jerry cans. Two years ago, the village government distributed taps to every household for handwashing facilities so that they could use flowing water.

Interviewer: But they did not have to depend on flowing water.

Participant: Correct. They did not have to depend on tap water.

Interviewer: All right.

Participant: Particularly during landslides.

Interviewer: Did these good practices occur only after socialization, or do they continue?

Participant: They continue today. Plan activities may occur only according to a schedule, but the village PKK movement now mobilizes all members to protect community health. Thank God, residents of [study village] now practice clean and healthy living behavior.

Interviewer: Excellent. The cadre understands the issue very well. [laughs]

Interviewer: Let us continue. You explained that landslides have occurred. Are there specific designs that help maintain water services and the five STBM(Community Based Total Sanitation) pillars in the village?

Participant: What do you mean by design?

Interviewer: Specific facility designs.

Participant: During landslide seasons, we try to locate toilets on higher ground and provide larger water-storage facilities.

Interviewer: What is the connection between larger water storage and landslides?

Participant: It is a preparedness measure. During landslides, water becomes discolored and turbid and cannot be used. We therefore prepare larger storage containers. Before a landslide occurs, OPAM(Community Based Water System) staff also inspect the pipes.

Interviewer: Thank you very much.

Interviewer: Could you explain how people access handwashing facilities during climate disasters?

Participant: As I mentioned, two years ago the village government distributed taps to every household for

handwashing facilities. We use jerry cans specifically for handwashing water and place basins beneath them. We do not waste the used water. We use it to water plants around our houses.

Interviewer: That is an excellent idea. Even during a climate disaster, handwashing facilities remain accessible. How do other residents of [study village] respond to situations similar to those your household experiences?

Participant: The community's response is now good. I observe that they practice what we have done and communicated.

Interviewer: During climate disasters such as landslides, do community members use good practices to maintain access to drinking water and toilets?

Participant: Yes. They apply the practices used in my household, such as spreading sand on paths to toilets. They also install supporting handrails near toilets.

Interviewer: What about people with disabilities?

Participant: People with disabilities have responded positively and use these practices. We no longer focus only on households that currently include a person with a disability. We do not know what may happen to us in the future, so we prepare accessible paths to toilets and handwashing facilities now.

Interviewer: Correct. So the facilities can be accessed by everyone, including people with disabilities.

Participant: Yes, yes.

Interviewer: Does the village have a water-management organization and an STBM(Community Based Total Sanitation) team?

Participant: Yes. There is an OPAM(Community Based Water System) and an STBM(Community Based Total Sanitation) team that collaborates with the health center and sanitarian. Youth, women, and people with disabilities also participate.

Interviewer: So all groups are involved.

Participant: Yes.

Interviewer: How many female cadres are there?

Participant: There are [a small number of female STBM cadres] in [study village].(Community Based Total Sanitation)

Interviewer: How many people with disabilities?

Participant: [A small number].

Interviewer: Are all of them involved?

Participant: Yes, all are involved.

Interviewer: What about young people?

Participant: All young people in the village participate.

Interviewer: How many are formally on the STBM(Community Based Total Sanitation) team?

Participant: [Several young people] are members.

Interviewer: They serve as representatives.

Participant: Yes, from each hamlet.

Interviewer: What roles and responsibilities do OPAM(Community Based Water System) and the STBM(Community Based Total Sanitation) team have toward community members?

Participant: STBM(Community Based Total Sanitation) and OPAM(Community Based Water System) members actively monitor sanitation and clean drinking-water services in the village.

Interviewer: Do they participate only in Plan activities, or also in other water and sanitation activities?

Participant: The groups I described do not focus only on Plan activities. The village has many other activities, and they participate in all of them.

Interviewer: In many places, people with disabilities have rarely been involved until recently. Did the participation of women and people with disabilities result from Plan's intervention, or did it already exist?

Participant: I have repeatedly said that, as an STBM(Community Based Total Sanitation) cadre, I am very grateful to Plan. Since Plan entered the village, gender equality has been practiced. There is no distinction between women and men or among age groups, including older adults. Most importantly, children and adults with disabilities are included. I am grateful that Plan's presence eliminated distinctions and enabled everyone to participate.

Interviewer: Thank you. The next question concerns

how the community responds to climate hazards in managing access to water and STBM(Community Based Total Sanitation).

Participant: Their response?

Interviewer: The community's response.

Participant: Regarding climate change, they appear to accept the current reality.

Interviewer: And adapt?

Participant: Yes, they adapt to the environment, but they also prepare for climate change.

Interviewer: Is that partly due to socialization by STBM(Community Based Total Sanitation) cadres?

Participant: Yes.

Interviewer: Thank you. How is the community involved in STBM(Community Based Total Sanitation)-related decision-making? Are residents invited to meetings or socialization about the five STBM(Community Based Total Sanitation) pillars?

Participant: We involve the entire community in decision-making. STBM(Community Based Total Sanitation) begins with triggering in each hamlet. We invite every resident without exception, and everyone is involved in decisions.

Interviewer: Are all age groups represented?

Participant: Yes.

Interviewer: Do you remember which age groups participated in triggering?

Participant: Children, adults, older people, people with disabilities, and women participated.

Interviewer: Thank you. You remember it well. [laughs] When was the most recent triggering?

Participant: In 2019.

Interviewer: So that was the most recent.

Participant: Yes, it was the most recent.

Interviewer: When was your most recent household monitoring visit?

Participant: I conducted household monitoring in 2021.

Interviewer: During the COVID period?

Participant: Yes.

Interviewer: We have discussed community involvement and disability. I apologize if we are moving too quickly. Please take a breath if needed.

Participant: It is fine.

Interviewer: How do you communicate and coordinate with people with disabilities so they can access water and sanitation during climate change?

Participant: We now have little difficulty explaining climate adaptation to people with disabilities, particularly regarding drought. Thank God, the village no longer experiences drought because mitigation was prepared from the beginning. Bathing, washing, and toilet facilities have been prepared so every family member can access them.

Interviewer: In our area, drought currently creates water shortages. You said that, because of Plan's intervention, water in [study village] is no longer severely limited. Can people with disabilities still access water and sanitation facilities during drought?

Participant: Yes.

Interviewer: Do they need assistance from others?

Participant: No. Thank God, the village has intervened. We now have OPAM(Community Based Water System) and meters at every household, so we no longer lack water during the dry season. People with disabilities also often prefer not to receive help. They can now go to the toilet and collect water independently because dedicated accessible facilities exist. They know the route to the toilet. That is why I am very grateful that Plan came to [study village].

Interviewer: Did Plan teach the approach, provide facilities for people with disabilities, or both?

Participant: Plan provided some facilities for people with disabilities, and the village government also intervened.

Interviewer: Did the village provide funding?

Participant: Yes. Village funds were used to provide facilities for households with members with disabilities.

Interviewer: What is an example?

Participant: A toilet specifically designed for people with disabilities.

Interviewer: What features does it have?

Participant: Standard toilets may be sufficient for people without disabilities, but households with members with disabilities need supports or handrails near the toilet so users do not fall and can sit comfortably.

Interviewer: So there is a handrail. Thank you. The progress in the village is remarkable.

Participant: Yes, it is all thanks to Plan.

Interviewer: Regarding institutional support, what assistance have government or other institutions provided to ensure continued access to water and STBM(Community Based Total Sanitation) during climate disasters? Was there support from institutions other than Plan?

Participant: Yes, from the village and other nongovernmental organizations. The village collaborates with [local organization], which conducted toilet-construction activities several years ago.

Interviewer: So support continues through connected collaborations.

Participant: Yes, one follows another, so support does not stop.

Interviewer: In that case, perhaps Plan may leave.

Participant: No, we still support Plan and want it to remain.

Interviewer: Let us continue. We discussed GEDSI(Gender Equality and Disability Social Inclusion), women and men, and equality. During climate change, how do older women and older men respond, and how is household work divided?

Participant: In the village, older people, whether women or men, are not assigned work. They mainly rest.

Interviewer: Is that true for nearly everyone? During a climate event such as a landslide that interrupts water, have older people ever had to cope without help or live alone?

Participant: No. Their children and children-in-law assist them.

Interviewer: Who has a larger role in deciding to modify household water and sanitation facilities, the husband or the wife?

Participant: Everyone is involved in decisions concerning sanitation and the community environment. It is not limited to men and women. Even children participate.

Interviewer: Excellent. The same is true in your household. Your husband does not make all water and sanitation decisions. You and the children also influence them.

Participant: Yes. Children and older family members are involved.

Interviewer: You have participated in gender-equality and inclusion activities. During a climate disaster, whose water- and sanitation-related workload increases most, men's or women's?

Participant: Who is most affected in what way?

Interviewer: Whose workload becomes highest?

Participant: No one is burdened alone. Everyone participates.

Interviewer: Thank you. Are there specific challenges that people with disabilities or other vulnerable groups face in accessing water and STBM(Community Based Total Sanitation) during climate disasters?

Participant: At present, I do not think they face major challenges because accessible facilities have been prepared.

Interviewer: As a cadre, you have the capacity to conduct socialization and ensure access to water and toilets. Does [study village] have local wisdom related to climate-risk reduction?

Participant: The village practices [local water-source protection tradition]. We remember the elders' saying, “neka poka puar, neka tapa satar,” so water remains abundant and spring flow does not decline during the dry season. It means that forests should not be cut or burned.

Interviewer: I understand. Thank you for recalling the [study city]saying. Are there traditional practices in the village that help address climate disasters? I once heard that a settlement performed a ritual so strong winds would not strike its area but would move elsewhere. Does anything similar occur?

Participant: Yes.

Interviewer: What is an example?

Participant: We conduct Penti in the village. At the new year, we perform teing hang in the traditional house.

Interviewer: What request is made? Is it a request that disasters not occur?

Participant: We ask that disasters not occur and express gratitude to our ancestors for what we received during the past year. We prepare to welcome new blessings in the coming year. The ritual is conducted every New Year's Eve.

Interviewer: One prayer is that disasters will not occur.

Participant: Yes, that no disaster will strike the settlement.

Interviewer: On a scale from one to five, where one means no effect and five means a very severe effect, how strongly do climate disasters threaten access to water and STBM(Community Based Total Sanitation) facilities in your area? You mentioned landslides. Which number would you select?

Participant: I would select three.

Interviewer: Why?

Participant: We no longer face water shortages. The main hazards we must anticipate are landslides and strong winds.

Interviewer: Before the final question, what concerns do you have that motivate action to reduce damage caused by climate disasters?

Participant: I am concerned about landslides because they make water turbid. I am also concerned about strong winds because many residents plant large trees near their houses. During strong winds, a tree could fall onto a neighbor's house or our own.

Interviewer: A fallen tree could also affect water facilities.

Participant: Yes.

Interviewer: Thank you. The final question is about Plan's program approaching its end.

Participant: Yes.

Interviewer: What are your impressions and messages? Which Plan programs could you continue as an STBM(Community Based Total Sanitation) cadre without Plan's intervention?

Participant: I am very grateful to Plan. Without Plan, I could not have done these things. Through Plan, I learned a great deal and gained knowledge. My message is that, if possible, Plan should not end its work because the community still needs it. Honestly, community behavior is difficult to change. With Plan's presence, changing behavior became easier. We still need Plan.

Interviewer: Which program could you continue as an STBM(Community Based Total Sanitation) cadre without Plan's intervention?

Participant: The five-pillar program.

Interviewer: Which pillar, or all of them?

Participant: We will continue focusing on handwashing and clean drinking water. In fact, we will continue all of them.

Interviewer: Those are all my questions. Is there anything else you would like to share?

Participant: No, the questions were complete.

Interviewer: Thank you for your time this evening and for sharing with us. We will convey the information to Plan colleagues. I hope we meet again.

Interviewer: Goodbye.

Participant: Thank you. Goodbye.
