# Supplementary material for: Implementing a climate-resilient and inclusive WASH guidance in Indonesia: A mixed-methods case study
Source: PLoS One. 2026 Sep 28;21(9):e0359266. doi: 10.1371/journal.pone.0359266 (PMC13618932; doi:10.1371/journal.pone.0359266)
Supplement: Supplementary file 6 [file pone.0359266.s006.zip › S6 Appendix Transcripts/KII-15_Deidentified_English.docx]

Interviewer, Participant

Interviewer: Good afternoon, [participant]. Thank you for making time for this interview. May we begin?

Interviewer: Could you introduce yourself, explain your role in the village, and tell us how long you have lived there?

Participant: My full name?

Interviewer: Yes.

Participant: My full name is [participant].

Interviewer: What is your role in the village?

Participant: I am an ordinary community member and have no village position.

Interviewer: What is your role in relation to Plan?

Participant: Um, the same as… who was it?…

Interviewer: [local facilitator]?

Participant: Yes.

Interviewer: Oh, you are a member of the village STBM(Community Based Total Sanitation) team?

Interviewer: How long have you held that role?

Participant: Since Plan first entered [study village/local beach].

Interviewer: So you have participated since the program began, as long as [local facilitator].

Interviewer: You have participated since the beginning. Could you explain climate-resilient STBM(Community Based Total Sanitation) GEDSI(Gender Equality and Disability Social Inclusion)? Have you attended socialization?

Participant: Climate resilience?

Interviewer: Or STBM(Community Based Total Sanitation) GEDSI(Gender Equality and Disability Social Inclusion)first.

Participant: I know the five pillars, such as providing handwashing water and separating rubber waste from paper waste.

Interviewer: Do you remember why it is called GEDSI(Gender Equality and Disability Social Inclusion) and how it relates to you?

Participant: Yes, because I can also use the facilities.

Interviewer: Everyone can access them, including [participant]. Have you participated in climate-resilience activities?

Participant: Yes, although I only partly remember. They discussed floods, droughts, and sea-level rise.

Interviewer: Was that explained during village socialization?

Participant: Yes.

Interviewer: What was your role?

Participant: What was my role at that time… um…

Interviewer: Were you a participant or facilitator?

Participant: I was a facilitator with [local facilitator].

Interviewer: All right, a facilitator.

Participant: [local facilitator] had the main role, and I accompanied [local facilitator].

Interviewer: Thank you.

Interviewer: What was your impression of Plan's socialization?

Participant: It helped us understand the importance of cleanliness, the risks of dirty conditions, waste, and disease.

Interviewer: Thank you. After participating in climate-resilient STBM(Community Based Total Sanitation) GEDSI(Gender Equality and Disability Social Inclusion) socialization, did you modify anything at home, particularly water or sanitation facilities?

Participant: I provided a handwashing bucket in front of the house, but it broke, and I have not yet purchased another.

Interviewer: What else?

Participant: We use a large earthen water-storage container.

Interviewer: What about access to the toilet?

Participant: We constructed a wastewater drainage channel.

Interviewer: You constructed a wastewater drain?

Interviewer: Did you install a handrail between the house and toilet?

Participant: No, because I can move relatively easily.

Interviewer: So you did not need a special modification and can reach the toilet easily?

Interviewer: Are there other difficulties accessing water or sanitation?

Participant: No. If piped water does not flow, a neighbor has a well where we can collect water.

Interviewer: Is it difficult for you to collect water yourself? You only need to go to a neighbor?

Participant: Yes, several nearby neighbors have wells, so it is not far.

Interviewer: You constructed wastewater drainage as a preventive measure so that…

Participant: Water does not stagnate, particularly wastewater from washing dishes or clothes.

Interviewer: Does flooding occur during heavy rain?

Participant: Water infiltrates directly because there are no large drainage channels.

Interviewer: Your house is near the market and coast, so does the water flow directly toward the sea?

Participant: Yes.

Interviewer: The area appears low-lying. Does water enter the house?

Participant: No.

Interviewer: Is there a drain along the front of the house?

Participant: The front of the house is higher, so water does not enter.

Interviewer: How did your experience change after climate-resilient STBM(Community Based Total Sanitation) GEDSI(Gender Equality and Disability Social Inclusion) socialization?

Participant: I met people from different subdistricts.

Interviewer: You expanded your network.

Participant: And, what is it called…

Interviewer: What about employment?

Participant: I am grateful that I was given work and am now involved.

Interviewer: Was that due to Plan's intervention?

Participant: Yes, through cooperation with Plan. [program staff member] once explained it.

Interviewer: All right.

Participant: I became a representative from [study subdistrict].

Interviewer: Is flooding the most common extreme weather here?

Participant: No, only drought.

Interviewer: During drought, are water and sanitation facilities damaged, or do they remain safe?

Participant: Water may stop for one or two days, but then it flows again. The reason is usually a fallen tree or a broken main pipe.

Interviewer: Does the water utility repair it?

Participant: Yes, the water utility.

Interviewer: Is there a village OPAM(Community Based Water System) , or is the service managed by government?

Participant: There is a subdistrict water utility.

Interviewer: How do people maintain water and sanitation services through specific designs? For example, what prevents floodwater from entering the house?

Participant: A drainage channel.

Interviewer: The drainage channel prevents heavy rain from entering the house.

Interviewer: Did you construct the roadside drain, or did government build it?

Participant: The roadside drain belongs to the government.

Interviewer: What about drainage inside the property?

Participant: We constructed it permanently.

Interviewer: You mean your wastewater drain. Did you construct it yourself after participating in Plan's activity?

Participant: Yes. After participating, I understood the purpose of drainage, including preventing mosquitoes.

Interviewer: During climate events, particularly drought, can you still use handwashing facilities, or do you conserve water because flow is low?

Participant: We use them as usual. If piped water does not flow, we collect well water.

Interviewer: Thank you. That was your personal response. Based on your observations, how does the community cope with drought?

Participant: It depends on each person. Residents in one RT were once advised to use a designated waste-disposal site, but over time people stopped caring and managed waste individually. Sometimes the group leader organizes communal work. Last week, for example, we cleaned the beach at [study village/local beach].

Interviewer: Did you collect waste there?

Participant: We cleaned the area and burned waste so that people unloading fish at [study village/local beach] and all residents of the market RT could use it.

Interviewer: Was everyone involved together with the government?

Participant: Yes, with the village government.

Interviewer: Does the village have a water-management organization such as OPAM(Community Based Water System) ?

Participant: I do not think there is an OPAM(Community Based Water System) .

Interviewer: But there is an STBM team that includes you and [local facilitator].

Are young people represented on the team?

Participant: Young people?

Interviewer: Yes, on the STBM(Community Based Total Sanitation) team.

Participant: I think there are some.

Interviewer: How have you been involved in the STBM(Community Based Total Sanitation) team at village or district level?

Participant: I participated at the district level with [local facilitator].

Interviewer: What did you do at the district level?

Participant: I participated in an activity at the [local hotel].

Interviewer: Did it concern STBM(Community Based Total Sanitation)?

Participant: It covered several topics.

Interviewer: Did it include climate-resilient STBM(Community Based Total Sanitation) GEDSI(Gender Equality and Disability Social Inclusion)?

Participant: Yes.

Interviewer: Were you a participant?

Participant: Yes, and also with a facilitator.

Interviewer: All right.

Participant: Actually, [local facilitator] was the facilitator.

Interviewer: Oh, you were a co-facilitator?

Interviewer: After the socialization, did you conduct practical activities in villages?

Participant: Not yet.

Interviewer: Never?

Participant: Oh, you mean visits to villages? Yes.

Interviewer: What did you practice?

Participant: During the village visit…

Interviewer: I recall that you once visited a kelurahan in [study city]. Do you remember?

Participant: Oh, a kelurahan in [study city].

Interviewer: What activities did you conduct there?

Participant: We participated in a kelurahan activity with children with disabilities.

Interviewer: Do you remember what you explained? Did you teach people with disabilities about toilet access?

Participant: It depended on each person's disability. Some were deaf, for example.

Interviewer: For people with physical disabilities, such as you, what did you explain about toilet access?

Participant: I use the toilet normally, although a seated toilet is better.

Interviewer: Did you recommend installing handrails?

Participant: [NOISE]

Interviewer: How do communities respond to current climate hazards, such as sudden shifts between rain and heat?

[…]

Interviewer: Can they adapt as you do?

Participant: Yes, they can adapt.

Interviewer: They simply accept it?

Participant: Yes. [laughs]

Interviewer: Could you describe community involvement in decision-making? For example, during household monitoring, did you successfully persuade a household to stop open defecation or change another practice?

Participant: Yes. At the market, we taught people not to defecate or dispose of waste indiscriminately and to separate wet and dry waste.

Interviewer: Did they listen because you were an STBM(Community Based Total Sanitation) team member?

Participant: They also wanted to make those changes themselves.

Interviewer: As an STBM(Community Based Total Sanitation) team member, how do you communicate with communities about water-access problems during drought?

Participant: Yes…

Interviewer: What do you tell them?

Participant: Wells need to be drained and cleaned to remove mosquito larvae. Water-storage containers should also be washed once a week.

Interviewer: Thank you.

Participant: Drinking water should be boiled.

Interviewer: During climate hazards such as drought, how do workloads differ between older men and older women?

Participant: Older women appear to do more.

Interviewer: Why?

Participant: Because women can do many different tasks.

Interviewer: Is that based on your observations or only your personal experience?

Participant: It is what I have observed.

Interviewer: When deciding to modify household water and sanitation facilities, such as constructing a toilet or installing a water meter, who decides, the husband or the wife?

Participant: Usually the matter is discussed with the wife first and then the husband.

Interviewer: Is it a joint decision, or does the wife have a larger role?

Participant: The wife has a larger role and manages more of it.

Interviewer: Are you personally involved in household water and sanitation decisions?

Interviewer: Because you live with your children, do you manage everything?

Interviewer: What are the experiences of other people with disabilities in [study village/local beach]?

Participant: Their experiences are normal.

Interviewer: Is there anyone with a severe disability who uses a wheelchair or has difficulty reaching the toilet?

Participant: There is a boy in a house below here.

Interviewer: How does he access the toilet? Have you visited his home? Participant: They constructed a handrail and a squatting support.

Interviewer: Did you teach them to do that?

Participant: I think [local facilitator] handled that area. We were assigned to the market area.

Interviewer: Did you inspect his toilet?

Participant: Not yet.

Interviewer: Who told you that a handrail had been constructed?

Participant: [local facilitator].

Interviewer: Between men and women, whose water, sanitation, and STBM(Community Based Total Sanitation) workload is higher?

Participant: I think women's.

Interviewer: Women have the more dominant role?

Participant: Most residents in our market area are women, and women are more active.

Interviewer: Do vulnerable groups, including you, face specific barriers to obtaining drinking water and sanitation?

Participant: Not particularly. When water stops, bottled water is sold. We may boil it again, although some bottled water has already been treated. [NOISE]

Interviewer: So the main obstacle is the need to spend money on water. Do you have difficulty reaching the toilet or collecting water?

Participant: No.

Interviewer: During drought, neighbors provide access to wells, as you described.

Participant: Yes.

Interviewer: Is there local wisdom that reduces disaster risks, such as preventing landslides or prolonged rainfall?

Participant: Cutting trees is prohibited here, particularly in [local protected forest], the protected forest near the market. People may collect dry wood but may not cut trees. This is one way to prevent flooding.

Interviewer: On a scale from one to five, where one means no effect and five means a very severe effect, how strongly do climate disasters threaten your access to water and sanitation?

Interviewer: Which number would you select?

Participant: Neutral, three.

Interviewer: Why?

Participant: It corresponds to the situation.

Interviewer: What is the current climate like?

Participant: Dry and hot.

Interviewer: Can you still adapt to the current weather?

Participant: Yes.

Interviewer: Could you describe a personal experience in which a climate disaster affected your access to water or STBM?

Interviewer: For example, during drought, was well water ever unavailable?

Participant: Yes, I have gone to the river.

Interviewer: You went to the river when well water was unavailable?

Participant: Yes. During drought, the water level in the well declines and little remains, so we go to the river.

Interviewer: How do you reach the river?

Participant: I walk.

Interviewer: Is that difficult?

Participant: No.

Interviewer: What about other people with disabilities? Do they receive assistance?

Participant: They may receive help, but I have not seen another person with a disability go to the river.

Interviewer: Apart from you, have you seen another person with a disability go there?

Participant: No, only me.

Interviewer: What concerns do you have about your ability to act during a future climate disaster?

Participant: I am mainly concerned about strong winds and heavy rain that could cause sea levels to rise.

Interviewer: Has seawater ever risen?

Participant: No. I am only concerned that it might.

Interviewer: That is your concern.

Participant: Yes, especially because we live near the coast.

Interviewer: Thank you. Is there anything else you would like to share about your experience and involvement with Plan?

Participant: Nothing else. I only hope we can repeat these activities and gather together again, unlike before.

Interviewer: Plan's program is approaching its end. What message or impression would you like to share, and which program would you continue if Plan were no longer present?

Participant: If Plan is no longer here, I hope it can return.

Interviewer: So you still need Plan?

Participant: Yes.

Interviewer: What is your message and impression of Plan?

Participant: Through Plan, we became acquainted, learned to respect one another, and developed relationships.

Interviewer: All right.

Participant: That is all.

Interviewer: Thank you. Plan involved you not only in STBM(Community Based Total Sanitation) but also in other social activities. Were you involved in social activities before Plan, or only after you became connected with Plan?

Participant: I became involved after meeting Plan. Even my current work resulted from Plan.

Interviewer: Thank you. Is there anything else you would like to share?

Participant: No.

Interviewer: Thank you for your time, and I apologize for interrupting your work.

Participant: It is fine.

Interviewer: Thank you. Good afternoon.
