# Supplementary material for: Implementing a climate-resilient and inclusive WASH guidance in Indonesia: A mixed-methods case study
Source: PLoS One. 2026 Sep 28;21(9):e0359266. doi: 10.1371/journal.pone.0359266 (PMC13618932; doi:10.1371/journal.pone.0359266)
Supplement: Supplementary file 6 [file pone.0359266.s006.zip › S6 Appendix Transcripts/KII-16_Deidentified_English.docx]

Interviewer, Participant

INTERVIEW NOTES

Interviewer: Thank you for your time. Let us begin with the first question. How long have you worked here?

Participant: [long-serving village employee and program team chair]

Interviewer: What is your village role related to Plan?

Participant: [long-serving village employee and program team chair]

Interviewer: Chair?

Participant: Chair of the team in [study village].

Interviewer: As chair, have you participated in inclusive STBM(Community Based Total Sanitation)activities?

Participant: Yes, in many activities.

Interviewer: Could you describe the activities Plan conducted?

Participant: At first, Plan introduced the five pillars. After the five pillars, the program moved to GC. The most recent topic, if I remember correctly, was climate.

Interviewer: Climate?

Participant: Yes, in relation to the five pillars.

Interviewer: What was your role in the workshop?

Participant: I was usually a participant, while on several occasions I served as a

Interviewer: protocol officer?

Participant: Facilitator.

Interviewer: Do you remember activities from STBM(Community Based Total Sanitation) GC and climate-resilience sessions, such as mapping?

Participant: We conducted mapping in several areas of [study village]. The first was [local neighborhood] near the coast, and the second was [local neighborhood] in the rice-field area.

Interviewer: Apart from mapping, did you conduct household monitoring?

Participant: Yes, we visited households. Several households had begun making changes in drinking-water storage, bathroom access, and solid-waste management.

Interviewer: How did they manage wastewater?

Participant: Among households I visited in [study village], including my own, most reuse household wastewater to water plants or dampen the ground because the climate is hot. Watering the ground reduces dust.

Interviewer: So water does not stagnate?

Participant: Correct, because stagnant water could affect breathing.

Interviewer: There is no standing water?

Participant: Correct.

Interviewer: Thank you. You have described your village role and participation in inclusive and climate-resilient STBM(Community Based Total Sanitation) as an STBM(Community Based Total Sanitation) team member. These questions now focus on your household. As team chair, what modifications did you make at home after participating in inclusive and climate-resilient STBM(Community Based Total Sanitation) activities?

Participant: We currently rent our house, and the bathroom is inside, so access is not particularly

Interviewer: problematic.

Participant: My husband and I are still young, so we do not currently need inclusive access features. When we own a house, we may construct them in preparation for old age. Regarding household drinking water, access in [study village] is difficult. During heavy rain, water pipes often break and supply may stop completely. Therefore, our main modification is increased storage. We purchase more water drums.

Interviewer: Because only you and your husband live there, bathroom access is not difficult. What about visitors or relatives? Do they face access difficulties?

Participant: Based on my experience, no.

Interviewer: If you build your own house, would you consider modifications?

Participant: Yes, they are very necessary. In old age, our backs may be weak, so we will need handrails and appropriate access to the bathroom.

Interviewer: To the toilet.

Participant: Yes.

Interviewer: Why should disaster-resilient facilities be inclusive and accessible to everyone?

Participant: Inclusivity is important because we do not live alone. Other people, including people with disabilities, also need access. We are not the only people living on Earth, and others may need these facilities more than we do.

Interviewer: As you said, we may also develop joint problems in old age.

Participant: Yes. We may not need the features now, but everyone will grow old.

Interviewer: What extreme weather has recently occurred in [study village]?

Participant: During the last several months, the most extreme condition has been intense heat, which may have reduced water availability and flow.

Interviewer: Is the current heat similar to or different from the last five years?

Participant: It is hotter now than in previous years. One or two years ago, [study village] was also very hot and experienced an entire year without rain. Early in 2024 it rained, but afterward

Interviewer: the heat became extreme.

Participant: Yes, extremely hot.

Interviewer: All right.

Participant: It remains hot here even when rain has begun in [nearby city].

Interviewer: It is still hot?

Participant: Yes.

Interviewer: During drought or other climate events such as flooding and heavy rain, are water and sanitation facilities damaged?

Participant: During hot weather, water flow declines. When utility water flows, we must fill many storage drums so we do not run out. During heavy rain, pipes often leak or break. The village or water utility uses plastic pipes. I do not know whether they are of the best quality, but heavy rain frequently causes leakage and breakage. It may be better to replace them with higher-quality pipes.

Interviewer: How old are the pipes?

Participant: I am not sure.

Interviewer: The pipes?

Participant: Water entered [study village] when we were in the first or second year of junior high school. The system has used plastic pipes from the beginning, although they are frequently replaced.

Interviewer: Does the village allocate funding to replace pipes?

Participant: No. The regional government manages pipe replacement.

Interviewer: Through the regional water utility?

Participant: Yes.

Interviewer: You have described damage during drought and rain. What specific measures could maintain water and STBM(Community Based Total Sanitation) services during disasters?

Participant: During rain, water flow is abundant, but pipes break quickly, perhaps because they cannot accommodate the high volume. During extreme heat, flow declines. Perhaps use should be reduced because almost all of [study subdistrict], not only our village, relies on the same water source.

Interviewer: I mean specific infrastructure designs. Flood-prone areas may elevate wells. Given the drought and excessive rain you described, what design would you use?

Participant: At my mother's house, floodwater from rice fields entered the kitchen because the drainage channel was too low. In the future, we may raise the drainage channel so water cannot enter the house.

Interviewer: The house again.

Participant: Yes, that is one measure.

Interviewer: Even if you have not yet constructed it, you have a plan.

Participant: Yes.

Interviewer: Does your current house flood?

Participant: The current house has a high foundation, so it does not flood. If I build my own house, I will construct drainage, especially in a low-lying area. Water flows from the rice fields.

Interviewer: Is that what causes flooding?

Participant: Yes. Rice fields cannot hold all the water. Because this housing area is low-lying, rainwater can enter

Interviewer: the houses?

Participant: Yes. Water enters several neighboring houses because the road is higher than the houses.

Interviewer: All right.

Participant: This extends beyond my personal household because I also work in the village. The infrastructure we have constructed includes drainage channels intended to prevent flooding from reaching neighboring homes. RT(Neighborhood) leaders and residents proposed drainage channels so water from the rice fields would flow into the channels rather than directly into houses.

Interviewer: So the area experiences both drought and flooding?

Participant: Yes. Rain is infrequent, but when it comes, it can be very heavy and

Interviewer: cause flooding.

Participant: Yes.

Interviewer: During climate disasters, do people have difficulty using handwashing facilities?

Participant: As I said, we use a storage system. During drought, we must always provide stored water for handwashing. We do not depend directly on taps.

Interviewer: Do you use a dipper or jerry can for handwashing?

Participant: During COVID, the village provided handwashing facilities.

Interviewer: Are they still used? That is a good practice.

Participant: Yes. In my household, I always wash my hands before cooking.

Interviewer: Thank you. Based on your observations, how do nearby communities experience and address climate-change challenges?

Participant: In [study village], when flooding sends water onto roads, residents work together to create small channels that direct it into larger drains.

Interviewer: Through community self-help?

Participant: Yes. We cannot wait indefinitely for government assistance while water damages our homes. We construct small channels ourselves. In addition, as I mentioned, almost every household in [study village] has many water-storage containers.

Interviewer: Many containers?

Participant: Yes. That is one way we

Interviewer: mitigate the impact.

Participant: Yes. During drought, water flow declines, so we store as much as possible.

Interviewer: Did Plan introduce this mitigation practice, or did residents already use extensive storage?

Participant: I was born and raised in [study village], so I know its history. When we were children, there was no piped water. Our sources were wells, and we carried water home in jerry cans and stored it. Households have always had many storage containers. Utility water entered the village when I was in the second year of junior high school, if I remember correctly.

Interviewer: Piped utility water?

Participant: Yes, water from a distant spring. Before that, we were accustomed to these conditions. Life is easier now because piped water exists, but water storage has always been common.

Interviewer: What difference did Plan's intervention make for water access if storage already existed?

Participant: Plan's contribution focused more on management and construction methods. I participated in training on constructing infiltration wells.

Interviewer: I see.

Participant: Infiltration wells prevent standing water. That was an innovation from Plan that I had not previously seen in [study village]. Plan also introduced different types of bathroom and toilet handrails.

Interviewer: Does the village have a water-management organization? You already said there is an STBM(Community Based Total Sanitation) team. Is there an organization for the piped-water system?

Participant: No. The regional government manages the water utility and recruits all employees, so I do not think a village organization exists.

Interviewer: There is none from the village. Is there an STBM(Community Based Total Sanitation) team?

Participant: Not for water.

Interviewer: But the village does have an STBM(Community Based Total Sanitation) team?

Participant: Yes, but I do not think it has a role specifically related to water.

Interviewer: It handles water management insofar as it relates to the five STBM(Community Based Total Sanitation) pillars?

Participant: Yes.

Interviewer: Does the STBM(Community Based Total Sanitation) team include representatives of youth, women, and people with disabilities?

Participant: Yes. Several colleagues have disabilities, and we always invite them to activities related to Plan.

Interviewer: How many people with disabilities are members of the team?

Participant: [A small number of people are formally on the team, and they have often participated in activities together.]

Interviewer: Thank you. How is the community involved in STBM(Community Based Total Sanitation) decision-making?

Participant: Community members here understand many issues. I will share one experience. During a survey in [local neighborhood], I visited households and learned that a child frequently defecated in the open. I repeatedly visited the family. When I saw feces beside the house, I approached the mother and said, “Please teach your son not to defecate outside. There is already a bathroom behind the house. Please help him become accustomed to using it.” Each time I returned, I provided [a small encouragement]. The child also rarely wore clothes. After several visits, he pulled me aside and said, “Look, I am wearing clothes now.” He was [a young child at the time and is now older]. He is very polite and always greets me. I asked whether he still defecated outside. In [Study City] language, he replied that he no longer did and always used the bathroom and toilet. Approaches like this can motivate even a young child. The key is repeated personal engagement.

Interviewer: Effective communication.

Participant: Yes. Simply talking repeatedly without engagement may not work.

Interviewer: Regarding community involvement and communication about water access during disasters, what approach is most effective?

Participant: These environmental conditions are familiar, so communities have become accustomed to them and act on their own.

Interviewer: Because the same events occur repeatedly?

Participant: Yes. Even without instructions, almost every household we visit provides the same response.

Interviewer: As an STBM(Community Based Total Sanitation) team member, how do you communicate so that everyone can continue accessing water and STBM(Community Based Total Sanitation) services during disasters?

Participant: We use a family-oriented approach. The most effective method is household-to-household conversation. We should not hurt people's feelings or use harsh language because of the condition of their homes. We speak gently and enter the home respectfully. That is my approach.

Interviewer: A familial approach.

Participant: Yes.

Interviewer: The STBM(Community Based Total Sanitation) team cannot work alone. Do government or other institutions provide support to ensure household access to drinking water and STBM(Community Based Total Sanitation)?

Participant: As I mentioned, the village has provided assistance several times. Households without clean-water access received support to obtain water meters. Several years ago, the Public Housing Office provided toilets to households that lacked them. During COVID, the village also provided handwashing containers because water is difficult to obtain and many houses lack taps. Village and regional governments have provided considerable sanitation support.

Interviewer: Did the disaster-management agency intervene when disasters occurred?

Participant: Once, during flooding, although the target was rice fields rather than households because the fields were damaged.

Interviewer: Through the disaster-management agency?

Participant: Yes, in collaboration with the Agriculture Office.

Interviewer: What did they construct, a drainage channel or embankment?

Participant: They repaired drainage channels and a dam damaged by flooding so rice fields would not be destroyed.

Interviewer: And this also prevented flooding from reaching houses.

Participant: Yes, that was the most important result.

Interviewer: Thank you. Based on your experience in [study village], how does climate change affect the workloads of older men and older women during disasters?

Participant: My parents live beside us. When a disaster such as

Interviewer: flooding occurs,

Participant: everyone in the family participates. We clean the drains together to prevent blockages. However, some households include an older man who has had a stroke. Reaching facilities is extremely difficult for him because surfaces may be slippery. That situation requires special attention.

Interviewer: That differs from other households.

Participant: Yes. In several households, the bathroom or toilet is outside and relatively far from the house.

Interviewer: In your household, who usually decides to modify water and sanitation facilities, the husband or the wife?

Participant: My husband and I always communicate and discuss the best decision.

Interviewer: The husband is not dominant?

Participant: Correct. Every household issue must be discussed.

Interviewer: As an STBM(Community Based Total Sanitation) team member, you are already involved in water and sanitation decisions. More generally, during climate change, whose workload is greatest, women's or men's?

Participant: In the village generally, based on what I have observed during extreme flooding, the people who take the greatest role are…

Interviewer: Those who collect water?

Participant: Not water collection. During floods, men usually take the larger role.

Interviewer: More often men?

Participant: Yes, in the wider community. But if…

Interviewer: Drought.

Participant: During drought, most of the people who draw and carry water are women.

Interviewer: Okay. So men perform more of the physical work when disasters occur, especially floods, whereas women more often go to collect water.

Participant: Yes.

Interviewer: Thank you very much. Regarding vulnerability, can you explain the specific challenges faced by vulnerable groups in accessing water and sanitation in your community? [NOISE] People with disabilities… [unclear].

Participant: [The participant described a family example in which older relatives received assistance with water and household tasks.]

Interviewer: So there was cooperation and someone helped her. Okay, we will leave the discussion of vulnerable groups. Next, regarding local wisdom, is there any local knowledge related to reducing the risk of climate hazards?

Participant: Such as?

Interviewer: For example, you may have heard about a case in [nearby city] involving strong winds. I believe the strong winds were originally in [traditional community area], but through their customs or local knowledge, they were able to redirect the windstorm toward the neighboring area.

Participant: Here, perhaps it relates to the rainy season. In [Study City], people call it *[local weather-warning expression]*. For example, during the rainy season, if there is a celebration or event, I do not really understand how it works, but the sky may already be dark and rain seems imminent, and then it suddenly becomes clear. I do not fully understand it, but it has happened.

Interviewer: And it happens often.

Participant: It often happens like that.

Interviewer: But when it is truly the rainy season, when the rainy season is already established?

Participant: No, no.

Interviewer: They no longer use *[local weather-warning expression]*?

Participant: No. At that point, it is already God's power.

Interviewer: Okay. Next, does your village have any traditional knowledge or practices for addressing the impacts of these climate disasters? What you described earlier was local wisdom, whereas this question concerns practical actions.

Participant: There are none.

Interviewer: Only the local practice you mentioned earlier?

Participant: Yes.

Interviewer: Okay. I will give you a scale from one to five, where one means no effect and five means a very severe effect. What number would you choose to indicate how much climate disasters have threatened water and sanitation access?

Participant: Three.

Interviewer: Why?

Participant: In our area, when it rains continuously for several days or throughout the day, the water supply automatically stops completely. There is also a benefit because when it rains, we can use rainwater, but it is not effective.

Interviewer: Because of the water quality?

Participant: Yes, the water quality. That is the issue. When the water flow decreases, we have no choice but to collect water from wells. As you know, well water here contains a lot of limestone, so it is not very effective to use.

Interviewer: Okay. We have discussed climate change. What concerns influence your actions to reduce disaster risk?

Participant: My concern is that if the flow from the spring continues to decrease, eventually we will no longer be able to access water. That is probably my concern. Reforestation may be necessary. I think that is what is needed.

Interviewer: Thank you very much.

Participant: Most importantly, we need to protect the spring. That is my concern.

Interviewer: Thank you very much, [participant]. Those were the main questions, and you answered them well. For the final question, I need one statement from you about PLAN's program. Please share your impressions and a message, as well as what you are committed to continuing after PLAN's intervention ends. Which activity would you continue?

Participant: Perhaps for our community, and please do not be upset, I think drinking-water management should be the main priority. It would be better if we in [study village] had our own spring rather than sharing one with another community. That is what I would prioritize without further intervention from PLAN.

Interviewer: Okay.

Participant: That is the third pillar.

Interviewer: Yes, drinking-water management.

Participant: Yes. I think that is the most important issue.

Interviewer: Thank you, [participant].

Participant: Water has many benefits. We live in a hot area and need a great deal of water. Even now, in one day we drink more than eight glasses because we are constantly dehydrated. When we are active, we keep sweating, so we need water.

Interviewer: You need more water.

Participant: Yes, we need more water. That is the issue.

Interviewer: You need more water, but the water flow is already decreasing.

Participant: Perhaps because consumption is too high.

Interviewer: Okay, [participant]. One more matter remains. What message or impression would you like to share with PLAN staff?

Participant: I thank the PLAN staff. I began without knowing anything, but I have learned a great deal, and…

Interviewer: You can also share information.

Participant: Yes, I can share information. The program has personally taught me a great deal about healthy living practices. That is its effect on me personally. For the village and the surrounding community, PLAN has provided a great deal of assistance.

Interviewer: Okay. I want to confirm several points. Open defecation no longer occurs because toilets are inside the houses. For handwashing, you do not necessarily use running water, but…

Participant: We have water-storage containers.

Interviewer: You have storage containers and facilities provided by the village, and you use them. Regarding the third pillar, how do you manage drinking water at home?

Participant: Here, almost everyone, not only my household, boils drinking water. We first store the water because bacteria, vegetation, or other material may enter it, and we do not know what is happening in the mountains. We allow the water to settle.

Interviewer: You allow it to settle?

Participant: Yes, we let it settle before boiling it. We are required to boil drinking water. However, some people may be too lazy to boil water and drink it directly.

Interviewer: Does that still happen?

Participant: Yes. But thank God they have not become ill. Perhaps they are already resistant. Still, almost everyone lets the water settle and then boils it.

Interviewer: So it is boiled. Okay. How do you manage wastewater?

Participant: As I mentioned earlier, wastewater from washing vegetables is usually used for feeding animals, such as pigs, or given to cattle to drink. Dishwashing water is poured onto the ground to reduce dust.

Interviewer: Okay.

Participant: It is poured onto the yard around the house.

Interviewer: Okay. What about solid-waste management?

Participant: We usually collect the waste first and then burn it.

Interviewer: Do you separate it? What do you do with plastic or household waste?

Participant: Food scraps are given to livestock. Plastic waste is burned. We dig a hole and burn it there.

Interviewer: Okay, thank you very much. I only wanted to confirm those points.

Participant: That is our usual practice here.

Interviewer: Is there anything else you would like to share that you have not discussed or that I have not asked?

Participant: No, I think that is all. I mainly want to thank PLAN, and I hope the program can be expanded in [study village].

Participant: That is all.

Interviewer: Okay.

Participant: Programs like this greatly help people like us who may have limited knowledge, because we gain a great deal of new knowledge.

Interviewer: What are your hopes at the household level?

Participant: At the household level, it is more about…

Interviewer: Perhaps program sustainability?

Participant: Yes. There should be more household visits because without continued monitoring, we may follow the recommendations only during the first week. After the staff leave, we may return to our old habits. It may be better to continue household-level engagement.

Interviewer: Thank you very much, [participant], for sharing so much.

Participant: You are welcome.

Interviewer: You remember the concepts very well. I have noted your ideas about program sustainability. I hope they are truly implemented. Thank you very much for your time. I will end the interview now.
