# Supplementary material for: Implementing a climate-resilient and inclusive WASH guidance in Indonesia: A mixed-methods case study
Source: PLoS One. 2026 Sep 28;21(9):e0359266. doi: 10.1371/journal.pone.0359266 (PMC13618932; doi:10.1371/journal.pone.0359266)
Supplement: Supplementary file 6 [file pone.0359266.s006.zip › S6 Appendix Transcripts/KII-17_Deidentified_English.docx]

Interviewer and participant labels standardized

INTERVIEW NOTES

Interviewer: Hello, good evening, [participant].

Participant: Good evening.

Interviewer: Hello, hello. [Laughing]

Participant: Good evening.

Interviewer: Good evening, [participant]. I apologize for disturbing you for a moment.

Participant: Yes, okay. Thank you.

Interviewer: Can you hear my voice clearly?

Participant: Yes.

Interviewer: Okay. You may also turn off your camera. It seems our connection is not very good.

Participant: Okay. Yes, I have been struggling to connect since earlier.

Interviewer: Yes, let us turn it off. Thank you, [participant]. Thank you for completing the consent form earlier.

Participant: Okay.

Interviewer: Can we begin now?

Participant: Yes, please continue.

Interviewer: Thank you for your time, [participant], and I apologize for disturbing you this evening. You may already have heard information about this interview from [local contact]. In essence, we want to study community experiences with inclusive and climate-resilient access to water, sanitation, and hygiene, including STBM(Community Based Total Sanitation) and its other pillars. During this interview, please keep the components of the five pillars in mind. We will also use terms such as extreme weather and climate hazards. When I use those terms, please think of examples such as floods, landslides, drought, or sea-level rise, although that does not occur in [study locality] because we are far from the coast. I am asking for approximately one hour of your time, although that may be too long. We will try to finish within half an hour so you can attend evening prayers.

Participant: We already started earlier.

Interviewer: Oh, I apologize, [participant].

Participant: It is okay.

Interviewer: Okay, [participant]. I will now formally begin recording the interview because you completed the consent form and agreed to be recorded.

Participant: Yes.

Interviewer: This interview has three parts: general questions about you, your experiences and perceptions of inclusive and climate-resilient STBM(Community Based Total Sanitation) , and then the closing. What is your position at the health center?

Participant: [local environmental-health worker with several years of service]

Interviewer: Okay, you are a Sanitarian. How long have you worked as a Sanitarian at the health center?

Participant: [tenure generalized]

Interviewer: Okay, two years. Before that, how long had you worked as a Sanitarian?

Participant: What do you mean?

Interviewer: As a Sanitarian at the health center. Has it been more than two years?

Participant: It has been [several years]. This is an early-career role.

Interviewer: Oh, so after you qualified, you began working as a Sanitarian at [local health center]. Thank you. Let us continue with the first question. Have you heard of or participated in activities related to inclusive and climate-resilient STBM(Community Based Total Sanitation) ?

Participant: I only recently participated in inclusive and climate-resilient STBM(Community Based Total Sanitation) through PLAN. However, for regular STBM(Community Based Total Sanitation) , our health center has conducted triggering activities several times in villages. The PLAN workshop was my first experience with inclusive and climate-resilient STBM(Community Based Total Sanitation) , followed immediately by field action that day, last year if I remember correctly.

Interviewer: Could you explain in detail what you did during the triggering activity and during the climate-resilience activity?

Participant: During the recent activity, we served as facilitators. Our task was to guide and conduct triggering with community members. First, we reminded the community what STBM(Community Based Total Sanitation) is, what inclusive STBM(Community Based Total Sanitation) is, and what inclusive and climate-resilient STBM(Community Based Total Sanitation) means.

Interviewer: Was there also mapping?

Participant: Yes. The activity sequence included mapping. After mapping, we conducted transect visits to households and then held another group discussion with the community.

Interviewer: Thank you. For climate resilience, did you conduct the same activities, including mapping, or was it only socialization?

Participant: During the transect visits, we also addressed climate resilience. In several activities I attended, we happened to visit the [study village] and [study villages] . The weather was extremely hot. I will describe the sanitation access.

Interviewer: Yes.

Participant: Some toilets were very far from the house. Inside the house there were vulnerable people, such as pregnant women and older adults.

Participant: They had to travel from the house to a toilet one or two meters outside. When it rained, the area was slippery and muddy, and at night there was no lighting. We offered suggestions on how to prevent, or rather address, those problems and what they should do.

Interviewer: Thank you very much. You attended the workshop and then helped implement inclusive and climate-resilient STBM(Community Based Total Sanitation) . What was your impression of the activity?

Participant: The activity was quite good, very good. During my work at the health center, I had only known discussions of STBM(Community Based Total Sanitation) , perhaps inclusive STBM(Community Based Total Sanitation) . This activity added the climate-resilience component to inclusive STBM(Community Based Total Sanitation) .

Interviewer: Yes, correct.

Participant: It was very good. As a young person, it broadened my perspective about what I should consider when building a house in the future. For example, if I build a toilet outside, what difficulties will arise later? If I place the water source far from the house, who will have difficulty? I will. I think PLAN's effort to encourage this forward-looking thinking is very good, and the activity was very useful. I have one note about the training. We received only one day of training, including reviewing the manual, to prepare for a year of practice. In my opinion, one day for both training and reviewing the manual was far too short because this was new to us and we had not been exposed to all the terminology. We may have known some terms but not others. The number of training days should be increased in the future.

Interviewer: Thank you for that forward-looking perspective from a young person. Regarding the health center's STBM(Community Based Total Sanitation) team, is it also the village STBM(Community Based Total Sanitation) team, or how are the two teams organized?

Participant: We have… Let me explain. I have another colleague named [Informan Collage] who may understand our health center STBM(Community Based Total Sanitation) team better because I joined later and do not fully understand it. The villages we have visited also have STBM(Community Based Total Sanitation) teams, but in practice they may participate only when there is an activity. They are active, but perhaps not very active.

Interviewer: Okay, but during implementation…

Participant: Oh, I am sorry. We also have an STBM(Community Based Total Sanitation) team at the health center.

Interviewer: Yes. I mean, during monitoring, evaluation, or triggering in the villages, does the village STBM(Community Based Total Sanitation) team collaborate with the health center?

Participant: Yes. When we conduct triggering, they help gather the target participants and organize the transect process.

Interviewer: Thank you. In your opinion, is the CCRIW program suitable for implementation in villages? Why or why not?

Participant: Do you mean in my home village?

Interviewer: Yes.

Participant: I am from [study village], [study subdistrict], [Study City] . If there is another training or activity in the future, it could be conducted in my village. I think it would be very suitable because, through my work at the health center, I often visit households. I still find many homes where sanitation facilities are far from the house. The house may be uphill while the toilet is downhill, and the water source may also be far away. An activity like this could expose residents to relevant information and help them consider how to bring sanitation access closer to the house. In [study village], many sanitation facilities remain far outside the house and are also inadequate.

Interviewer: Thank you for the additional information about [study village]. In the villages where you have conducted triggering, do you think inclusive and climate-resilient STBM(Community Based Total Sanitation) would be suitable?

Participant: Yes, because it returns to the issue of access. After participating in CCRIW, residents may receive the information we provide and implement it in their daily lives. It is suitable because the activity is very beneficial. However, some people remain supportive or opposed, or they may understand the information but face constraints. We cannot deny that when we conduct activities, many residents ask whether there will be this assistance or that assistance. People often expect material support.

Interviewer: That is true and cannot be denied. Regarding satisfaction with implementation, how satisfied are you with the CCRIW process at the health center or in the villages you visited? Were there any aspects you found unacceptable or unsatisfactory?

Participant: At the health center, I am quite satisfied because, to speak honestly…

Interviewer: Yes.

Participant: Before the CCRIW activity, our bathroom did not even have a handrail. The general outpatient room and clinical laboratory were also very far apart. When it rained, patients became wet while moving between them. Through this activity, the health center and PLAN began collaborating. They considered how to improve the bathroom, including adding a handrail, and they installed paving on the path toward the laboratory so it would not become muddy. I am very satisfied with the activity.

Interviewer: Was there anything unacceptable or unsatisfactory?

Participant: So far, I am satisfied. Everything was acceptable.

Interviewer: Thank you. Let us continue. In the villages you visited for triggering, have they adopted the program?

Interviewer: Or at least implemented what was introduced?

Participant: I do not know yet because our activity was in [study villages].

Interviewer: Oh, okay.

Participant: There are also villages in our service area, including [study locality] and [local water-system/community area]. However, I do not know exactly how far they have progressed in adopting the program because I have not continued following them.

Interviewer: Has there been no monitoring and evaluation?

Participant: Not yet.

Interviewer: If a village has not adopted or implemented the program, what strategy do you think would effectively encourage government support? What kind of government support is needed?

Participant: Support may be needed, but if we discuss funding and continue giving assistance to communities, people may become increasingly dependent.

Interviewer: Would they need training or specific guidance to make the program successful?

Participant: Villages may need additional training. If we continue providing assistance, when will residents develop their own initiative? We should conduct triggering again.

Participant: Triggering may help residents understand what they should do without continually waiting for assistance.

Interviewer: Thank you. You already provided a general explanation of CCRIW, so I do not need to ask again. You also said that the training should last longer than one day. Regarding sustainability, what approach could ensure that the activity continues in your village? What could the village itself do without the STBM(Community Based Total Sanitation) team or PLAN?

Participant: Perhaps…

Interviewer: What kind of communication or coordination would be needed?

Participant: Because of the community's working mentality, there may need to be pressure from above. The regional government could encourage the villages by asking how far they have progressed with the activities we initiated. The regional government could monitor the village level and revive people's memory of the previous activities and agreements. During the activities, we made agreements in each village. The facilitator team did not take those agreements with us; we left them at the village office. Government monitors could review those agreements and determine which commitments have been implemented at the village level. That may be an effective approach.

Interviewer: Thank you for the information. You said that triggering was conducted only in [study villages] and West [study villages].

Participant: [study villages], [study villages], and [study locality].

Interviewer: So there was also an activity in the service area of [local health center]?

Participant: Yes.

Interviewer: Do you know whether those villages have an organizational structure, such as a village water-management group, and what their STBM(Community Based Total Sanitation) teams are like?

Participant: In [study locality], which is in my service area, they use the public water utility.

Interviewer: Okay.

Participant: For [that study location], we have several assisted villages in our…

Interviewer: Okay.

Participant: PLAN has several pilot villages across [coded study localities]. [Study locality] uses the public water utility, while [Study village location] and [local water-system/community area] use OPAM community water-management groups.

Interviewer: Is there collaboration?

Participant: Yes. We asked about the OPAM(Community Based Water System) structures in [Study village location] and [local water-system/community area], and they have formal organizational structures. Their village STBM teams also have structures. I do not remember whether it was [Study village location] or [local water-system/community area], probably [Study village location], where the fees were not being paid regularly. As a result, the manager worked only occasionally and faced difficulties. However, the organizational structure exists in writing.

Interviewer: If there is an OPAM(Community Based Water System) and a village STBM(Community Based Total Sanitation) team, do they collaborate with the health center's STBM(Community Based Total Sanitation) team regarding water and sanitation access?

Participant: What do you mean? If they have an activity…

Interviewer: Based on your experience, how are water and sanitation access managed? Do the water group and village STBM(Community Based Total Sanitation) team collaborate with the health center STBM(Community Based Total Sanitation) team, and what are the respective roles and responsibilities of the three organizations?

Participant: Since I began working, do you mean whether a village team has collaborated with the health center by inviting us?

Interviewer: Yes, including inviting you to meetings.

Participant: Very little, or not yet. We have never received an invitation when they conduct their own village STBM(Community Based Total Sanitation) or water-access activities. That has not entered their area of work.

Interviewer: So you met the village STBM(Community Based Total Sanitation) team only during the triggering activity.

Participant: Yes, during the recent CCRIW triggering activity.

Interviewer: Within the village or health center STBM(Community Based Total Sanitation) teams, do marginalized groups, including women, young people, and people with disabilities, participate in management and leadership roles?

Participant: I do not know about village meetings generally. However, I recently heard from an acquaintance with a visual disability that people with disabilities were involved in village meetings.

Interviewer: When you conducted triggering in villages, were people with disabilities also involved?

Participant: Yes. Village activities involved young men and women, older adults, pregnant women, and people with disabilities.

Interviewer: Thank you. We have been discussing climate hazards. Climate change is now unpredictable, as you know and experience. Based on what you know, how do communities respond, especially in managing water and sanitation access during disasters such as drought?

Participant: As you explained, perhaps five years ago rain occurred during particular months, but now the seasons have shifted. It may rain during months that should be dry. In [study village], we also experience severe water shortages. Sanitation activities, including using the bathroom, require a great deal of water, so shortages can hinder sanitation. People may still urinate outside, although not necessarily defecate openly, because there is insufficient water for the bathroom and it may seem better to go directly outside.

Interviewer: Yes, they conserve water. When drought occurs, or when pipes break during landslides or floods, how does the STBM(Community Based Total Sanitation) team respond?

Participant: I think the STBM(Community Based Total Sanitation) team's response is limited. The public water utility responds.

Interviewer: So infrastructure is mainly the responsibility of OPAM(Community Based Water System) and the public water utility, while STBM(Community Based Total Sanitation) focuses on behavior.

Participant: The STBM(Community Based Total Sanitation) team may participate during triggering and transect activities, but damage such as…

Participant: leaking or broken pipes is probably the responsibility of the water utility.

Interviewer: The STBM(Community Based Total Sanitation) team and health center cannot work alone and need support from other institutions. Has the government or another institution supported the STBM(Community Based Total Sanitation) team, health center, PLAN pilot villages, or nonpilot villages in maintaining access to water and STBM(Community Based Total Sanitation) services, particularly during climate disasters?

Participant: I have not followed that issue, so I do not know.

Interviewer: Based on your understanding, has information about water access and STBM(Community Based Total Sanitation) so far come only from PLAN?

Participant: Yes, in my opinion.

Interviewer: Have you communicated or coordinated with the government regarding inclusive and climate-resilient water and STBM(Community Based Total Sanitation) ?

Participant: So far, I have not communicated with the government about that.

Interviewer: If you were asked to speak with relevant officials about water, what would you say?

Participant: Regarding water and…

Interviewer: STBM(Community Based Total Sanitation) .

Participant: Water access should be improved for communities, especially for low-income households. Access could be brought closer to them. For STBM(Community Based Total Sanitation) , we also need to assess communities carefully. Some households genuinely need and should receive assistance, while others do not need it but continually ask for support. The government should determine which households truly require assistance.

Interviewer: Thank you. In your village, do you know or communicate with key figures who hold authority and can provide information about water and STBM(Community Based Total Sanitation) services accessible to everyone? In [Study City] communities, for example, there is a *[customary community leader]*. Who are the key figures in your village?

Participant: I am sorry, could you repeat the question?

Interviewer: Villages have key figures with whom we can discuss water and STBM(Community Based Total Sanitation) . In [Study City] communities, the *[customary community leader]* is a key customary figure. Based on your knowledge, who would be the key person from whom we could obtain information about water and sanitation?

Participant: I would probably go directly to the village head because that person should understand the village best.

Interviewer: Is there an imbalance in authority that affects the effectiveness of inclusive water and STBM(Community Based Total Sanitation) services, or are roles balanced despite the village head's power?

Participant: I do not know about that.

Interviewer: That is okay. We will leave that issue.

Participant: I am afraid of giving the wrong answer. [Laughing]

Interviewer: That is fine. In your experience, do you ask only the village head about water services?

Participant: Yes. We can go directly to the village office and ask the village head.

Interviewer: Is there no specific individual who could serve as an informant?

Participant: For questions about water, we might approach OPAM(Community Based Water System). However, they mainly know where the water is distributed and the schedule for opening and closing the valves. OPAM(Community Based Water System) staff are also rarely available and difficult to meet.

Interviewer: Thank you. Would you like to take a breath and drink some water? [Laughing]

Interviewer: Okay, we seem to be rushing now.

Participant: It is fine. Please continue.

Interviewer: All right.

Participant: My voice may sound congested because I have a cold. [Laughing]

Interviewer: Yes, I can hear you. Inclusive STBM(Community Based Total Sanitation) means that everyone can access it, including people with disabilities and other vulnerable groups. In your opinion, do these vulnerable groups face specific challenges in accessing water and STBM(Community Based Total Sanitation) , particularly during climate disasters? What challenges have you observed or heard about while conducting triggering in villages?

Participant: For vulnerable groups, if access is far away, it becomes difficult to reach.

Interviewer: And if the access…

Participant: The path, the path.

Interviewer: Okay, if it is far away, or, I am sorry…

Interviewer: Please continue.

Participant: What did you ask again?

Interviewer: If the facility is far away and the path is slippery, as you mentioned, what steps have you and your colleagues taken to help vulnerable groups reach toilets, especially during climate disasters?

Participant: We can use simple materials available in the environment. We can make handrails from rope or bamboo along the path. To reduce slipperiness, we can place small stones or gravel on the path.

Interviewer: Based on your village experience, have those practices been successfully implemented at any homes of people with disabilities? Have you received reports?

Participant: Yes. In several, or rather almost all, of the homes we visited in the villages assisted by [local health center], modifications have been made.

Interviewer: They were modified?

Participant: Yes. Handrails were installed, and gravel or small stones were placed on the paths.

Interviewer: To document that you and your colleagues have done this, could you later send one or two photographs as evidence?

Participant: Okay. At the homes of people with disabilities?

Interviewer: Yes, at their homes.

Participant: Okay.

Interviewer: One or two photographs.

Participant: Okay. Please remind me tomorrow.

Interviewer: Certainly.

Participant: Yes, because I may forget.

Interviewer: Thank you very much.

Participant: I will take the photographs tomorrow.

Interviewer: Thank you. We are nearing the end. Regarding knowledge and capacity, could you describe the community's general awareness of climate-change impacts in the villages, the health center's area, or your current service area?

Participant: You mean their awareness?

Interviewer: Yes, their awareness of current climate-change impacts.

Participant: They are aware of climate change. However, returning to what we discussed earlier, their toilets may remain outside the house because of constraints known only to them. They may be unable to make changes. They recognize that the climate and seasons are shifting. They are aware of it.

Interviewer: The problem is implementation. [Laughing] It seems people simply accept the situation.

Participant: They may not know what else to do.

Interviewer: [Laughing] Okay. What concerns do community members, officials, or community leaders have that may affect their ability to take action to reduce damage caused by climate disasters?

Participant: Could you explain that again?

Interviewer: I am sorry. Do community members, village officials, or village leaders have concerns that influence how they think about potential damage caused by climate disasters? For example, when drought reduces water access, what do they think about the declining water supply?

Participant: Perhaps they have become aware of the problem. Because water is scarce, several community groups have begun planting trees near water sources and carrying out environmental activities around springs. The dry season is now very severe.

Interviewer: Yes. Which villages are currently experiencing drought?

Participant: Villages in our service area are affected. We recently visited [Study village location], which was also dry. Here, [study locality] is also dry. Many residents complain that water does not flow, or flows only in very small amounts.

Interviewer: Do people now have to travel far to collect water, or are there wells as a mitigation measure?

Participant: Some places may have wells, but many people must travel a long distance to collect water.

Interviewer: Does this increase women's or men's workloads? Is it mainly women's work?

Participant: Yes, mainly women.

Interviewer: So most of that responsibility falls on women.

Participant: In my view, yes. However, men sometimes drive them and also help transport the water.

Interviewer: So there is cooperation.

Interviewer: That means the GEDSI(Gender Equality and Disability Social Inclusion) component is functioning.

Participant: Yes. There is awareness that this is also my responsibility.

Interviewer: Perhaps there is something I forgot to ask. No, I think that is all. Is there anything else you would like to add about your triggering experience or anything related to the CCRIW program?

Participant: I think I have already shared most of it.

Interviewer: Thank you. For the final question, please make a statement about your message or impression of PLAN's CCRIW program and its influence on the pilot villages you helped accompany. Of PLAN's various activities… Please answer the first part first. I am sorry.

Participant: The connection is breaking up.

Interviewer: Yes. PLAN's program is nearing its end. Please share a message or impression from participating in CCRIW with PLAN staff and working with the community.

Participant: My impression is that the activity was extremely good and very helpful. As a young person, I received the information and also served as a facilitator. Many young people participated. Because we are all young, I think we had similar impressions. They said that CCRIW and inclusive, climate-resilient STBM(Community Based Total Sanitation) exposed them to new ideas. They now understand how they should build a house and construct a toilet in the future.

Interviewer: It should be resilient to the climate.

Participant: The activity encouraged us as young people to think far ahead. We now consider what should be done so that conditions will not make life too difficult for us in the future.

Interviewer: Yes, correct. That is a positive value. You plan to modify your future house.

Participant: We will also grow old, and it is possible that we may develop disabilities later.

Interviewer: Correct.

Participant: We need to think about and prepare for that future.

Interviewer: Finally, among the five STBM(Community Based Total Sanitation) pillars and the GEDSI- and climate-resilience-based components, which are you committed to continuing without PLAN's intervention?

Participant: I think we should implement all the pillars because they are all related to sanitation.

Interviewer: Okay.

Participant: They also relate to water. I think we must remain committed to all five pillars.

Interviewer: Thank you very much for that commitment, [participant]. Those were all the questions. I apologize for taking nearly an hour of your time, and I appreciate it. We will end the interview now.

Participant: Yes.

Interviewer: Thank you, [participant]. Good evening.

Participant: Good evening. Thank you.
