# Supplementary material for: Implementing a climate-resilient and inclusive WASH guidance in Indonesia: A mixed-methods case study
Source: PLoS One. 2026 Sep 28;21(9):e0359266. doi: 10.1371/journal.pone.0359266 (PMC13618932; doi:10.1371/journal.pone.0359266)
Supplement: Supplementary file 6 [file pone.0359266.s006.zip › S6 Appendix Transcripts/KII-18_Deidentified_English.docx]

Interviewer and participant labels standardized

INTERVIEW NOTES

Interviewer: Good evening, [participant].

Participant: Good evening, [interviewer].

Interviewer: I apologize for disturbing you this evening.

Participant: It is okay.

Interviewer: Can you hear my voice clearly?

Participant: Clearly. Very clearly.

Interviewer: Okay. Have you completed the consent form?

Interviewer: The Google Form.

Participant: Oh, the Google Form…

Interviewer: Yes. It is also fine to complete it after the interview, as long as you have agreed to be interviewed.

Participant: Yes, certainly.

Interviewer: [Participant], our connection seems weak. Perhaps you should turn off your camera. Can you still hear me clearly?

Participant: Yes, that is fine.

Interviewer: Okay, thank you. Shall we begin with the questions, or is there anything you would like to ask?

Participant: We can go directly to the questions, [interviewer], so that…

Interviewer: Thank you very much. What do you currently do at the health center?

Participant: I currently manage several programs, including environmental-health programs.

Interviewer: For the village, are you a member of the CCRIW team?

Participant: Yes. I am part of the CCRIW team.

Interviewer: How long have you been on the team? For simplicity, I will call you a facilitator, although I mean the village CCRIW team because the formal term is somewhat cumbersome. When I say facilitator, I mean your role on the village CCRIW team.

Participant: Yes.

Interviewer: Since when have you served as a village facilitator?

Participant: I have managed this program and served as a facilitator for a long time, [interviewer].

Interviewer: Approximately how many years? Since PLAN arrived?

Participant: It has been… Even before PLAN, we were already conducting those activities through the environmental-health unit in villages.

Interviewer: For PLAN specifically, it began around 2018, correct?

Participant: Yes. Since PLAN began operating in [Study City], I have served as a facilitator.

Interviewer: When was your most recent village activity?

Participant: It was in early 2024, around March or April.

Interviewer: Because you have served as a village facilitator, you have participated in inclusive and climate-resilient STBM(Community Based Total Sanitation) activities. Could you describe the activities PLAN conducted, including whether there was mapping, transect walking, or group discussion?

Participant: Regarding PLAN's intervention and our role as facilitators, PLAN first trained us. After the training, we implemented activities in several villages. The activities proceeded well, and we performed our facilitator roles well because PLAN had provided initial training that we then implemented with communities. That is the process, [interviewer].

Interviewer: Thank you. Did you participate in last year's training on inclusive, climate-resilient STBM(Community Based Total Sanitation)?

Participant: Yes, I participated.

Interviewer: Do you remember what activities were conducted? Was it only discussion, or were there other activities?

Participant: We conducted triggering in villages, especially about mitigating climate changes and involving inclusive groups in responding when climate change occurs. Ultimately, we identified solutions together with the community.

Interviewer: Was there also mapping?

Participant: Yes, there was mapping because…

Interviewer: So the activities were almost the same as the earlier GEDSI(Gender Equality and Disability Social Inclusion) STBM(Community Based Total Sanitation)

activities?

Participant: Yes. GEDSI(Gender Equality and Disability Social Inclusion) STBM(Community Based Total Sanitation) activities were quite similar. GEDSI(Gender Equality and Disability Social Inclusion) STBM(Community Based Total Sanitation) focused more specifically on gender roles and how men and women act within the STBM(Community Based Total Sanitation) program.

Interviewer: What distinguishes GEDSI(Gender Equality and Disability Social Inclusion) STBM(Community Based Total Sanitation) from climate-resilient STBM(Community Based Total Sanitation)?

Participant: The distinction is that the first program we implemented was GEDSI(Gender Equality and Disability Social Inclusion) STBM(Community Based Total Sanitation). It did not yet address how climate change affects the STBM(Community Based Total Sanitation) program. The main distinction is…

Interviewer: The community response?

Participant: Yes, [interviewer]. In the first program, GEDSI(Gender Equality and Disability Social Inclusion) STBM(Community Based Total Sanitation) did not yet include a climate-change component.

Interviewer: Yes.

Participant: The second program was continuous with the first. Climate-resilient STBM(Community Based Total Sanitation) also includes gender roles, so the GEDSI(Gender Equality and Disability Social Inclusion) STBM(Community Based Total Sanitation) program and climate-resilient STBM(Community Based Total Sanitation) form one integrated program.

Interviewer: Thank you for the information. After participating in the activity or socialization on inclusive and climate-resilient STBM(Community Based Total Sanitation), what was your impression?

Participant: In my opinion, these programs are very good for communities. Communities responded positively and were enthusiastic about implementing the activities. Several villages have now undertaken different interventions, including planting tree seedlings and planning facilities for people with disabilities. These include environmental or sanitation activities, such as providing toilets. Some villages have constructed public bathing, washing, and toilet facilities that consider access for people with disabilities visiting the village office. There are also several other examples.

Interviewer: So your conclusion is that this activity is suitable for implementation in villages?

Participant: It is very suitable, [interviewer], very suitable for villages.

Interviewer: If it is suitable, how satisfied are you with its implementation in villages?

Participant: I am very satisfied, [interviewer]. I frequently visit villages as a facilitator, and this program is highly suitable for communities.

Interviewer: Have many communities adopted this approach? How has it been received? Has anyone rejected it?

Participant: No one has rejected it. When the program is implemented in villages, it creates awareness and willingness among residents to change their behavior and ways of thinking. For example, when water flow decreased recently, they understood that it resulted from frequent tree cutting in forests or around springs, and perhaps also from waste. They were very enthusiastic about the activities. My impression is very positive.

Interviewer: Thank you. How many villages have you accompanied?

Participant: PLAN intervened in four villages.

Interviewer: Have you implemented the program only in those pilot villages, or has it spread across the [local health center] service area?

Participant: It has spread. Although PLAN intervened, I see this as a Ministry of Health program rather than something intended merely to complete PLAN's work. We use the four villages as examples for implementation in other villages that did not receive PLAN intervention, for both programs we discussed.

Interviewer: So nonpilot villages have adopted the same program?

Participant: Yes. Cross-sectoral collaboration in northern [study subdistrict] is very strong. For example, the subdistrict head instructed all villages, not only PLAN's pilot villages, to implement the same program because he considers it highly beneficial.

Interviewer: Because several villages have adopted it and the subdistrict government supports it, is additional training or special guidance still needed to ensure success and sustainability or integrate it with existing Ministry of Health programs?

Participant: Continued accompaniment is certainly needed. When we conduct activities, we must coordinate with village governments. We always inform the health center when a village implements an environmental or STBM(Community Based Total Sanitation) program, such as constructing public bathing, washing, and toilet facilities, installing water infrastructure or pipelines, or planting tree seedlings around springs.

Interviewer: You said several villages have adopted CCRIW. As far as you know, how are implementation costs funded?

Participant: An evaluation was conducted several months ago. The villages funded the activities from their own budgets and planned the programs themselves.

Interviewer: Does the village budget contain a specific allocation or budget nomenclature for CCRIW?

Participant: Yes. Villages have specific allocations for programs related to CCRIW.

Participant: Several villages have designated funding, and the activities must be implemented each year.

Interviewer: Has this created a financial impact on village budgets? For example, have people complained that funds intended for other activities must now be divided with CCRIW?

Participant: No one has complained so far.

Interviewer: As a team member or facilitator, do you advise villages on lower-cost implementation, such as using a bench or another local material as a handhold for people with disabilities?

Participant: There are different funding specifications for different activities. Larger activities include constructing water facilities, planting seedlings, maintaining pipelines, and procuring waste containers. For smaller needs, I recently attended a population-related meeting with village governments across northern [study subdistrict] at the subdistrict office, where the funding was separated. Items such as handrails for people with disabilities or other needs that do not require much funding are provided through community self-help. Direct interventions at the homes of people with disabilities may require different funding because there are many people with different types of disabilities in several villages. For public facilities, communities generally provide the modifications themselves.

Interviewer: That is where facilitators help change community behavior. Returning to the CCRIW training, what did you learn, and was the training easy to understand?

Participant: It was very easy to understand because everything was explained in detail. We implemented it well in the field, and community responses showed that the training had been effective.

Interviewer: Is there anything in the CCRIW training or implementation that should be revised or improved?

Participant: I think the training was already very good.

Interviewer: Some people said that one day was insufficient because the manual contained a great deal of material. What was your experience?

Participant: That may be true. Because of my professional background, the material connected with my existing knowledge. It may also depend on the facilitator. I do not mean to boast, but it aligned with our background.

Participant: Therefore, we could follow it naturally. I do not know about the other participants.

Interviewer: That makes sense. The other informant I interviewed may have encountered the material for the first time. Were there any challenges in the workshop facilitated by PLAN?

Participant: During all my work with PLAN, as a partner or colleague since PLAN first arrived here, I have remained enthusiastic. I have not encountered significant problems because the activities are very good and include innovations that motivate us. The main challenge is traveling from village to village.

Interviewer: What about you personally?

Participant: Everything was good.

Interviewer: Regarding sustainability, is there a particular approach that could ensure these activities continue in villages without PLAN's intervention or without facilitator assistance?

Participant: Several villages have implemented the activities, but they still need assistance or accompaniment from facilitators, including village actors and perhaps the health center.

Interviewer: What support is needed for program sustainability?

Participant: Mainly accompaniment. If something is incorrect or deviates from the intended approach, it may be because they are confused, and we can guide them.

Interviewer: Turning to technical matters, do villages have organizational structures for water-management groups?

Participant: Yes.

Interviewer: Does each village also have an STBM(Community Based Total Sanitation) team?

Participant: Yes.

Interviewer: What are their roles and responsibilities? Do the water-management groups collaborate with STBM(Community Based Total Sanitation) teams?

Participant: Yes. Members of water groups, usually OPAM(Community Based Water System) managers, are also included in STBM(Community Based Total Sanitation) teams. Water-related matters can therefore be discussed within the STBM(Community Based Total Sanitation) group and addressed appropriately. In several villages, the management structure includes a chair and members and involves all relevant community figures, including the water-management group.

Interviewer: Are vulnerable groups represented in leadership and management, including women, young people, and people with disabilities?

Participant: Yes. People with disabilities are also represented.

Interviewer: Are they invited to meetings, or are they members of the STBM(Community Based Total Sanitation) or OPAM(Community Based Water System) teams?

Participant: The STBM(Community Based Total Sanitation) team.

Interviewer: Thank you.

Participant: Several villages also involve women.

Interviewer: We now face climate hazards, and climate change has become unpredictable. How do communities respond, particularly in managing drinking-water and STBM(Community Based Total Sanitation) access?

Participant: During our activities with [named person], communities expressed considerable concern. Villages face different problems depending on their topography and other conditions. They are concerned about the changes and events occurring. Several villages specifically complain about water shortages.

Interviewer: Do they have to collect water again?

Participant: Yes. Some complain because rain has not fallen, which affects their crops and other aspects of their lives.

Interviewer: Whose workload becomes heavier, women’s or men’s?

Participant: It varies. There are particular tasks in which men take a greater role, such as collecting water. When the water source is far away, men play an important role in fetching it. From what I have seen and asked, women usually remain at home.

Interviewer: Thank you. That indicates GEDSI(Gender Equality and Disability Social Inclusion) implementation during disasters, with cooperation between women and men. Regarding institutional support, do you receive government support to ensure access to water and STBM(Community Based Total Sanitation) services during climate disasters, including for people with disabilities?

Participant: Yes, the government provides support, [named person]. In villages we visited through PLAN’s intervention, the subdistrict head issued specific instructions to educate communities and pay attention to people with disabilities, including their water access.

Interviewer: Have you communicated or coordinated with government officials about inclusive and climate-resilient water and STBM(Community Based Total Sanitation) issues?

Participant: Yes, for activities that had been jointly planned. We have implemented those activities. We have not coordinated on isolated or unplanned activities.

Interviewer: Which government officials did you coordinate with, the Health Office, the subdistrict, or another institution?

Participant: The village government.

Interviewer: When you visit villages, who are the key individuals or authority figures from whom you can obtain information about water and STBM(Community Based Total Sanitation) services accessible to everyone?

Participant: We first meet customary leaders, such as the *Tu’a Golo* or other traditional authorities. We ask them questions and seek permission before intervening, because access to certain places requires their consent. We may also approach the *Tua Gendang*.

Interviewer: What specific challenges do vulnerable groups face during climate disasters when accessing water and STBM(Community Based Total Sanitation)?

Participant: Before and during our activities, we found that when water is scarce or when vulnerable people need to use sanitation facilities such as toilets, they usually ask other family members for assistance to meet their needs.

Interviewer: Have you heard of a person with a disability living alone or in a one-person household?

Participant: No. I have not heard of or seen that. They are not abandoned.

Interviewer: Are there social norms or cultural practices that affect their vulnerability, either helping or making conditions more difficult? For example, do people ever think that a person with a disability does not need assistance?

Participant: No. They treat them like any other person.

Interviewer: Thank you. How aware are communities in the villages you support of current climate-change impacts? Have they adapted to unpredictable rain and prolonged heat?

Participant: They are now able to adapt while also trying to understand why the changes occur. They frequently discuss and ask about those issues.

Interviewer: What forms of adaptation and mitigation occur in the communities you support?

Participant: During village activities, we discuss with residents what problems are occurring and why. After identifying solutions, they implement the results of the activity. One example is planting tree seedlings. They recognized that tree planting is a solution because the most visible impact is declining water flow.

Interviewer: I understand that they have adapted and use tree planting as a mitigation measure.

Participant: They plant trees and establish rules, such as prohibiting indiscriminate waste disposal, including practices that protect soil conditions and the environment.

Interviewer: Have facilitators actively socialized those measures?

Participant: Yes.

Interviewer: Finally, what concerns do community members, officials, or village leaders have about damage that climate disasters may cause?

Participant: Based on my observations, they have several concerns. One is declining water flow. Some villages still face very difficult access because water flow is insufficient. Farmers are also concerned about unpredictable rainfall. Crops such as corn should be planted at particular times, but when rain does not fall consistently, yields are affected. The same applies to other crops.

Interviewer: So climate change affects not only STBM(Community Based Total Sanitation) but also people’s lives and livelihoods?

Participant: Yes. Some people grow vegetables for sale. Several years ago, perhaps two years ago, they used water from public pipelines to irrigate vegetables. Because water is now scarce, it can no longer be used for irrigation and is reserved for drinking and household cooking.

Interviewer: Overall, is the water supply sufficient for drinking and sanitation?

Participant: From what I observed and monitored, it was reasonably sufficient for community needs, although each village faces different problems.

Interviewer: Does insufficient water cause people to return to open defecation?

Participant: Some may do so, but the program has increased their understanding and influenced behavior related to open defecation. Men and women now try, according to their respective roles, to ensure that enough water is available at home. Because of the program’s influence, almost all houses have toilets, except perhaps newly built houses where a toilet has not yet been constructed.

Interviewer: So we can confirm that the area is genuinely open-defecation-free. Could you later send photographs from village visits, especially modifications at homes of people with disabilities, such as handrails or adapted toilet seats?

Participant: Yes, I will send photographs.

Interviewer: PLAN’s program is nearing its end. As a partner, you likely have a commitment to sustaining it. Which PLAN activity are you most confident you can continue without PLAN’s intervention?

Participant: I am confident because this work is part of my responsibilities. I am very satisfied with PLAN’s programs and must adopt them. STBM(Community Based Total Sanitation) addresses community needs, and I am committed to implementing all of these programs in villages.

Interviewer: Thank you.

Participant: Community enthusiasm is also visible. My colleagues, the health-center head, the subdistrict head, village governments, and several community leaders have met at the subdistrict office to discuss sustainability. I am proud that the program continues, as shown by villages allocating funds for the activities.

Interviewer: I agree. Support from the subdistrict government is especially encouraging.

Participant: Yes.

Interviewer: Do you have a final message or impression for PLAN staff?

Participant: I am very grateful that I came to know PLAN and was invited to work with the organization. During my work with PLAN, I encountered new ideas that I had not previously found elsewhere, through both the staff and the programs. I am thankful, and my impression is very positive, both regarding program implementation and the relationships that made the activities possible. My message is that we still need PLAN in [Study City] and hope to continue collaborating. Wherever PLAN works, I hope its work continues successfully. That is all, [interviewer].

Interviewer: Thank you very much for sharing this evening and for your message to PLAN staff. Your answers provided the information needed for this study. I apologize for taking so much of your time and appreciate the opportunity to interview you.

Participant: One final point, [interviewer]. I currently have a cold and am ill.

Interviewer: Yes, I could hear that. I apologize again. Thank you very much for your time, and I hope you recover quickly. Good evening.

Participant: Yes.
