# Supplementary material for: Implementing a climate-resilient and inclusive WASH guidance in Indonesia: A mixed-methods case study
Source: PLoS One. 2026 Sep 28;21(9):e0359266. doi: 10.1371/journal.pone.0359266 (PMC13618932; doi:10.1371/journal.pone.0359266)
Supplement: Supplementary file 6 [file pone.0359266.s006.zip › S6 Appendix Transcripts/KII-19_Deidentified_English.docx]

Interviewer and participant labels standardized

INTERVIEW NOTES

Interviewer: Good afternoon, [participant].

Participant: Good afternoon.

Interviewer: Thank you very much for your time. As I explained earlier, this is the purpose of our discussion this afternoon.

Participant: Yes.

Interviewer: You have also signed the consent form for the interview.

Participant: Yes.

Interviewer: May we begin with the first question?

Participant: Yes.

Interviewer: Please introduce yourself, describe your role in the village, and explain how long you have held it.

Participant: Good afternoon. [long-term resident and neighborhood leader].

Interviewer: Thank you very much. You have served as a neighborhood representative for several years, correct?

Participant: Yes.

Interviewer: Did you participate in PLAN's inclusive and climate-resilient STBM(Community Based Total Sanitation) activity approximately two years ago?

Participant: Yes.

Interviewer: What was discussed during the socialization? Were there activities such as triggering or mapping? What do you remember?

Participant: They explained sustainable STBM(Community Based Total Sanitation) , and we visited community members to ensure the STBM(Community Based Total Sanitation) activity reached the appropriate households.

Interviewer: During that activity, did you also visit residents' homes?

Participant: Yes.

Interviewer: What did you observe or evaluate?

Participant: We observed how residents disposed of household wastewater and whether they had adequate bathing, washing, and toilet facilities.

Interviewer: Do you remember any discussion related to climate resilience?

Participant: Yes.

Interviewer: Could you describe it?

Participant: They explained climate change and its effects on communities.

Interviewer: Did they provide an example of climate resilience related to STBM(Community Based Total Sanitation) ?

Participant: The use of clean drinking water.

Interviewer: What about constructing sanitation facilities?

Participant: Yes, that was also explained.

Interviewer: Thank you.

Interviewer: After participating in the socialization and visiting households, what was your impression? Was it positive, or did you think the activity was unsuitable?

Participant: My impression was very positive. After we visited households, we identified [a household without an adequate toilet that was using an unsafe sanitation practice]. After we met with the homeowner, the household began constructing a basic bathing, washing, and toilet facility.

Interviewer: That is an excellent immediate result of the triggering activity. During the socialization, you discussed climate resilience and climate-resilient sanitation facilities, such as how septic tanks should be constructed so they do not collapse during disasters. Did you make any modifications at your own home based on the information?

Participant: Yes. The activity had many positive effects. Regarding the septic tank, we considered the proper flow and constructed it permanently with concrete so it would remain strong despite weather changes.

Interviewer: Has a septic tank in your area ever collapsed, flooded, or cracked?

Participant: Yes, ma'am.

Interviewer: Have residents reported experiences like that?

Participant: Not at present.

Interviewer: There have been no reports?

Participant: Everything we observed was permanent and constructed with concrete.

Interviewer: Besides permanent septic tanks, have other sanitation facilities, such as wastewater disposal, handwashing stations, and waste containers, also been designed with climate resilience in mind?

Participant: Yes, so far they have.

Interviewer: What kind of handwashing facility do you have at home?

Participant: We use a sink.

Interviewer: Is the sink inside the house?

Participant: Yes, and the wastewater is discharged outside.

Interviewer: Into a wastewater drain?

Participant: Yes.

Interviewer: What about other residents? They may not all have sinks.

Participant: Their facilities are at least reasonably adequate. We cannot require every household to have the same facilities because it depends on their finances.

Interviewer: But do they have running water?

Participant: Yes.

Interviewer: Can they still wash their hands during disasters such as droughts, landslides, or floods?

Participant: So far, yes.

Interviewer: There is no water-supply problem?

Participant: Not at present. We still have abundant water.

Interviewer: Thank you very much.

Interviewer: What extreme weather commonly occurs in [study Urban village], such as floods, landslides, or drought?

Participant: From what I have observed as a neighborhood head, when rainfall is high, waste carried into our area overflows from the drains and causes flooding. However, regarding a particularly dangerous climate event…

Interviewer: Drought?

Participant: [Nods]

Interviewer: Has recent drought affected the community?

Participant: Yes. Many residents here are farmers. When water is unavailable for irrigation, they clearly experience impacts.

Interviewer: But drought has not affected the five STBM(Community Based Total Sanitation) pillars?

Participant: No.

Interviewer: You said drains are often affected during high rainfall. How are damaged drains addressed? Are they reported to the Urban village, or do residents cooperate in community cleanups?

Participant: Recently, residents were affected by flooding and accumulated waste. The Urban village addressed the problem by repairing several meters of drainage leading to the houses.

Interviewer: How many damaged drainage locations were there in [local neighborhood]?

Participant: Only one nearby.

Interviewer: Have any water pipes been damaged or broken during heavy rainfall?

Participant: No, not so far.

Interviewer: So only the drainage was affected?

Participant: Yes, the drainage and overflowing waste carried from elsewhere.

Interviewer: Before the drain was repaired, what happened to the waste? I believe your house is also located…

Participant: Yes, it sometimes overflowed. If we did not respond during heavy rainfall, the waste accumulating in the main drainage channel could create serious consequences for us.

Interviewer: Especially because your house is in a low-lying area.

Participant: Yes.

Interviewer: Thank you. Do you have any special designs to maintain water services during disasters? Besides the septic tank you described, are there other facilities designed for climate resilience in your home or residents' homes, such as wastewater drains?

Participant: There is no special design, but it at least meets the standards.

Interviewer: How do you prevent floodwater overflowing from the drain from entering your house? Is there a raised channel?

Participant: Yes, there is a channel in front of the house.

Interviewer: Is the channel high enough to prevent water from entering your house?

Participant: Yes.

Interviewer: Thank you. That concerns modifications at your own home. How do other residents address overflowing drains?

Participant: It does not affect everyone, only a few households. The response depends on the community because the waste problem, especially waste carried from elsewhere, is complicated.

Interviewer: Waste coming from the urban village uphill?

Participant: Waste from other places, and we receive the consequences.

Interviewer: I understand.

Participant: It depends on the community.

Interviewer: When the waste accumulates, where is it disposed of?

Participant: We collect it. Following the recent STBM(Community Based Total Sanitation) activity, we provide simple waste containers made from sacks rather than special bins. Residents store waste in the sacks, and a vehicle from the environmental agency collects it.

Interviewer: So there is a waste-disposal system.

Interviewer: May I continue?

Participant: Yes.

Interviewer: Does the urban village have an organizational structure for a water-management group and an STBM(Community Based Total Sanitation) team?

Participant: There is no water-management group.

Interviewer: So there is no OPAM, and water is managed directly by…

Participant: The public water utility.

Interviewer: Is there a urban village STBM(Community Based Total Sanitation) team?

Participant: Yes.

Interviewer: Are you a member of the STBM(Community Based Total Sanitation) team?

Participant: No.

Interviewer: As far as you know, does the STBM(Community Based Total Sanitation) team include women, men, or representatives of people with disabilities?

Participant: Yes.

Interviewer: Are people with disabilities members?

Participant: In [study urban village], people with disabilities are not directly included, but some members represent residents with disabilities.

Interviewer: Thank you. We now face climate hazards. In [study city], this month should normally be rainy, but rainfall is now unpredictable. How do residents respond?

Participant: Residents can only accept the situation. There are many impacts, especially for farmers, who do not receive enough water for their rice fields.

Interviewer: Regarding climate hazards, especially water access and STBM(Community Based Total Sanitation), how do residents meet sanitation needs during drought, including toilets and bathing, washing, and toilet facilities?

Participant: Their needs are met. At present, we have no water-access problems. Despite changes in weather or climate, we continue receiving water from the public utility.

Interviewer: There are no problems like those reported in [study village] and [study village]?

Participant: Not among residents in my neighborhood. They continue receiving water.

Interviewer: So women do not need to collect water from springs?

Participant: No.

Interviewer: Thank God.

Interviewer: Could you describe community involvement in STBM(Community Based Total Sanitation) decision-making? I was interested in your earlier account of triggering that motivated a resident to change behavior and construct a toilet.

Participant: Communities generally accept beneficial programs. During PLAN's recent STBM(Community Based Total Sanitation) activity, we assessed local conditions and found one household staying in a house without bathing, washing, and toilet facilities. The person either dug a hole or defecated into a water channel. After we explained the problem and encouraged the household to build even a basic facility, they were able to construct one.

Interviewer: Does the household now have its own facility?

Participant: Yes, a simple one.

Interviewer: So the household no longer practices open defecation?

Participant: Correct.

Interviewer: Thank you. That is an example of effective communication with communities about water and STBM(Community Based Total Sanitation) access.

Participant: Yes.

Interviewer: Because there is no water-access problem, women, people with disabilities, and other vulnerable groups also do not face water-access problems, correct?

Participant: Correct. Access is safe.

Interviewer: During heavy rain, do any residents have toilets outside the house that are difficult to reach because of slippery paths? Have pregnant women or older adults reported difficulty reaching toilets during heavy rain or extreme heat?

Participant: Residents have not reported such problems. During our field visits, we saw some toilets outside houses, but they were not very far away and remained easy to access.

Interviewer: Is there government or institutional support to maintain access to water and the five STBM(Community Based Total Sanitation) pillars, such as the drainage support you mentioned?

Participant: Yes.

Interviewer: What type of support did the government provide?

Participant: The government supplied construction materials.

Interviewer: To construct a new facility?

Participant: They improved sections that had only earthen drainage by constructing proper channels to address flooding and waste.

Interviewer: So sections of drainage were damaged during heavy rain?

Participant: They were not damaged. The channels existed but had never been lined with concrete.

Interviewer: So the government constructed new drainage?

Participant: Yes.

Interviewer: New drainage channels?

Participant: Yes.

Interviewer: To direct water flow?

Participant: Yes, so it flows smoothly.

Interviewer: Thank you. Have you heard the term gender, meaning equality between women and men? In your opinion, how does climate change affect older men compared with older women? During disasters, whose household workload is heavier, an older man's or an older woman's?

Participant: The older man's.

Interviewer: Why?

Participant: Because his responsibilities involve more work.

Interviewer: More physical work?

Participant: Yes, physical work.

Interviewer: There is no need to collect water here, correct?

Participant: Correct.

Interviewer: So you believe the older man's workload is heavier?

Participant: Yes, because he addresses the problems we discussed.

Interviewer: Yes.

Participant: However, viewed differently, women may perform more activities overall.

Interviewer: Older women perform more work?

Participant: Older women cook, manage water, and do everything else.

Interviewer: So older women perform more household work, while older men do more physical work?

Participant: Yes.

Interviewer: Regarding decisions between husbands and wives about modifying water and sanitation facilities, when you planned to build a toilet, did you and your wife agree that it should be strong and concreted so it could withstand climate impacts? Who was more dominant in the decision, the husband or the wife, or were decisions balanced?

Participant: In my opinion, to achieve the best long-term result for the family, both should make the decision together.

Interviewer: Is that only your opinion, or is that how decisions are actually made?

Participant: That is my view based on what I have observed.

Interviewer: What happens in your own household?

Participant: If we want to do something, we discuss it together first.

Interviewer: Thank you. What about households that include people with disabilities? Before constructing sanitation facilities, do they consider climate resilience and accessibility?

Participant: So far, I have not heard complaints about people with disabilities having accidents while going to the toilet. I conclude that their families carefully consider the needs of children or other family members with disabilities.

Interviewer: Have you visited a household that includes a person with a disability?

Participant: Not in my own neighborhood, but several years ago we visited [named resident] in [local neighborhood], where an older person used a wheelchair.

Interviewer: What were the conditions there?

Participant: The person's daily activities were normal.

Interviewer: What about toilet access? Was there a special design?

Participant: There was no special design because the person's sibling and mother helped them reach the toilet.

Interviewer: They helped the person reach the toilet?

Participant: Yes.

Interviewer: But they could not carry the person throughout toilet use. Was there a special seat or handrail?

Participant: We did not inspect the interior design. We only observed the house. However, neither the family nor the person with a disability reported problems.

Interviewer: Besides that person, have you visited other households with people with disabilities?

Participant: There are not many people with disabilities here. For older people, toilet access is generally adequate. Families sometimes construct something like a railing that an older person can hold while walking to the bathroom.

Interviewer: Do you mean they have already constructed handrails?

Participant: Yes, something that serves as a handhold.

Interviewer: So handrails have been installed for older adults?

Participant: Yes. That was the family's own initiative.

Interviewer: Was it based on the triggering activity?

Participant: Yes, after the activities.

Interviewer: Do you know of any local wisdom related to reducing disaster risk?

Participant: Mainly community cooperation through collective work. We have not experienced a major disaster here. Our collective work mainly addresses waste carried into the area.

Interviewer: Have you applied knowledge from the climate-resilience socialization to improve water and STBM(Community Based Total Sanitation) services so they are disaster-resistant and accessible? We have discussed other people, but how does your household ensure that all family members can access facilities related to the five pillars?

Participant: We simply use and benefit from the water facilities. Everyone in the family can access them.

Interviewer: What about disaster-resistant construction?

Participant: We do not have a water-storage structure. We connect directly to the public water utility's installation.

Interviewer: Is that because water flows continuously? In other places, households prepare storage tanks.

Participant: Yes, the septic tank…

Interviewer: I mean a water-storage tank.

Participant: Oh, yes, a water tank.

Interviewer: Do you have one here?

Participant: No. So far, we continue without one. [Laughing]

Interviewer: I have not found many problems here except drainage.

Participant: Yes, drainage because waste carried from elsewhere overflows.

Interviewer: On a scale from one to five, where one means no effect and five means a very severe effect, what number shows how much disasters threaten your access to water and the five STBM(Community Based Total Sanitation) pillars?

Participant: Four.

Interviewer: Why do they have an effect?

Participant: If drought continues and water flow declines, the impact on agriculture will be severe.

Interviewer: That affects agriculture, but what about water for…

Participant: Community use and sanitation activities as well.

Interviewer: Thank you very much.

Participant: Yes.

Interviewer: Have you personally experienced a climate disaster that affected your access, such as having to travel far to collect water or being unable to use the toilet because there was no water?

Participant: So far, no. We have not encountered water-access problems since living here. The main impact occurs when the weather shifts from dry conditions to heavy rain and brings waste into our area. We must work hard to collect waste that enters the yard or piles up along the road because communities have not been attentive in cleaning drainage channels.

Interviewer: What concerns do you, the community, or the urban village have regarding climate change?

Participant: As I said earlier, prolonged dry seasons and reduced rainfall severely affect agriculture. Farmers who should be harvesting now do not obtain their usual yields because of climate change.

Interviewer: Are there concerns about access to the STBM(Community Based Total Sanitation) pillars?

Participant: Certainly. If climate change continues, water flow will decline. Communities will no longer receive the usual reliable supply, and STBM(Community Based Total Sanitation) activities will be seriously affected.

Interviewer: Thank you. Those were the main questions. Is there anything else you would like to share about climate-resilient water and STBM(Community Based Total Sanitation) that I did not ask?

Participant: It returns to community behavior. When people cut forests, the main impact is declining water flow. [Study City] is also more prone to landslides than some other areas. If forests are managed properly, water flow can continue meeting community needs despite climate changes.

Interviewer: Besides reducing tree cutting, do you have ideas for mitigation, such as planting trees to prevent drought?

Participant: The community cannot address it alone. The only way is to cooperate with local government to plant as many trees as possible around the main springs that supply [Study City] communities.

Interviewer: Have you participated in tree planting?

Participant: Not so far.

Interviewer: What about other mitigation measures, such as water storage? Have you considered how to ensure household facilities related to the five STBM(Community Based Total Sanitation) pillars withstand floods, landslides, or other climate impacts?

Participant: Which aspect do you mean, bathing, washing, and toilet facilities?

Interviewer: Infrastructure construction.

Participant: We have considered the best design for the septic tank. We would also prioritize maintaining clean-water access. To address future shortages, we may construct a water tank or another storage facility.

Interviewer: PLAN's program is ending. Do you have a message or impression about its programs, and is there anything you will continue without PLAN's intervention?

Participant: Beneficial programs are well received by communities. Because we work among residents every day, the response depends on how people perceive the activities provided. Personally, I applaud PLAN's work because it has had a major positive impact on us.

Interviewer: Thank you. Is there a program you will continue?

Participant: We will continue activities based on the five pillars.

Interviewer: Could you give one prominent example, perhaps related to waste?

Participant: Our waste activity is already operating.

Interviewer: What will you do?

Participant: I regularly direct residents to store their waste neatly in sacks before environmental-agency staff collect it.

Interviewer: Do you separate waste?

Participant: Yes, into wet and dry waste. At our house, organic material is used as fertilizer. We also burn some waste, although that method may be wrong. Without burning, waste accumulates. To reduce smoke exposure for residents, we burn it at night when people are no longer active.

Interviewer: Thank you very much. Is there anything else you would like to share?

Participant: Nothing else at present, but we are very grateful for PLAN's activities.

Interviewer: Thank you very much for your time. We apologize for disturbing you this afternoon. I will end the interview now.

Participant: Good afternoon.
