# Supplementary material for: Implementing a climate-resilient and inclusive WASH guidance in Indonesia: A mixed-methods case study
Source: PLoS One. 2026 Sep 28;21(9):e0359266. doi: 10.1371/journal.pone.0359266 (PMC13618932; doi:10.1371/journal.pone.0359266)
Supplement: Supplementary file 6 [file pone.0359266.s006.zip › S6 Appendix Transcripts/KII-20_Deidentified_English.docx]

Interviewer, Participant

INTERVIEW NOTES:

Interviewer: Okay, good morning, Mr.[Participant] . Thank you very much for making time for us.

Participant: Yes, good morning, Ma’am.

Interviewer: You have signed the consent form for this interview, so may we begin with the questions?

Participant: Yes, Ma’am. Ready, ready.

Interviewer: All right. This interview has three parts. First, I will ask general questions about you. Then, I will ask you to explain your experiences and perceptions of inclusive and climate-resilient STBM(Community Based Total Sanitation) . After that, we will close the interview.

Interviewer: First, [Participant] , please describe your role in this village and how long you have served here. Also, have you participated in Plan activities related to inclusive STBM(Community Based Total Sanitation) , particularly the climate-resilience activity conducted about two years ago?

Participant: Thank you, Ma’am. I am a [long-serving village official]. Regarding STBM(Community Based Total Sanitation) activities, we conduct them every year. The most recent activity concerned climate-resilient STBM(Community Based Total Sanitation) . The local community and the village government strongly supported it. We prioritized several water-source locations across [local hamlet areas]. The sustainable climate-resilience activities included constructing a spring-capture structure at [local spring], which is a spring in [local hamlet], and improving access by constructing a footpath using [substantial local funding]. Plan and the village jointly initiated and implemented those activities. Another Plan activity approved by the village was the renovation of the village office so that it would accommodate persons with disabilities, pregnant women, and other vulnerable groups.

Interviewer: Thank you very much, Mr.[Participant] . You have explained the climate-resilience activities, the infrastructure modifications and construction under STBM(Community Based Total Sanitation) , and the collaboration between Plan and the village. Those examples concern public facilities, such as springs and the village office. What about modifications at the household level? Has climate resilience also been considered in household facilities?

Participant: The first modification-related activity involved PKK(Family Welfare Empowerment) women and posyandu (Integrated Health Center) cadres. We encouraged them to promote family medicinal gardens( TOGA). However, I do not know whether the activity was actually implemented or whether it remained only information. At the household level, the five STBM(Community Based Total Sanitation) pillars have also been implemented in [study village].

Interviewer: Thank you. You have explained both public facilities and household-level activities. Regarding the CCRIW program, were there any other activities by Plan Indonesia, another organization, or the government with similar objectives that influenced water and sanitation programs?

Participant: Outside Plan’s CCRIW activities, the [Study City] district government also provided tree seedlings. We planted them at several spring locations, especially in [local hamlet], and we have maintained them.

Interviewer: Oh, so in [local hamlet], in addition to the footpath, tree seedlings were also planted?

Participant: Not there, Ma’am. In...

Interviewer: Oh yes, in the other [local hamlet] you mentioned.

Participant: Yes, in [local hamlet], Ma’am. We protected the spring located in [local hamlet].

Interviewer: Have the tree seedlings begun to grow?

Participant: Yes, they have, Ma’am.

Interviewer: Thank you very much. Regarding this collaboration with the government, was there also...

Interviewer: ...advocacy from Plan, or was it purely cooperation between the village government and the district government?

Participant: Plan initiated the activity first. The district government then saw that the spring site had been improved and could be used when the regular water service was interrupted. The district government therefore became involved and offered support so that the water flow would remain stable and the source would remain protected.

Interviewer: Thank you very much, Mr.[Participant] . That information is very useful. Next, we would like to know about village funds for water and climate-resilient STBM(Community Based Total Sanitation) . Have village funds been used to construct new sanitation facilities or rehabilitate existing ones, and why? Was this due to Plan’s advocacy, or did it arise from the village government’s own awareness?

Participant: As I explained earlier, the activities resulted from Plan’s advocacy, and we have demonstrated that through implementation. The budget was approximately 140 million rupiah for two main locations. It covered the construction of a footpath to the spring in [local hamlet], the construction of the spring-capture structure, and renovation of the village office. Plan activities emphasized the need to consider persons with disabilities and pregnant women.

Interviewer: Okay.

Participant: Therefore, the village made efforts to ensure that persons with disabilities who attend activities at the village office, as well as pregnant women, would be safe and could access facilities, particularly the bathroom.

Interviewer: That is remarkable, Mr.[Participant] . Plan appears to have had a substantial influence in [study village], not only at the conceptual level, but also through actual implementation.

Interviewer: We may later ask for photographs as evidence, Mr.[Participant] .

Participant: Yes, certainly, Ma’am.

Interviewer: The village has used funds to rehabilitate facilities and construct new sanitation infrastructure. Does the village also prepare an annual plan specifically for inclusive and climate-resilient STBM(Community Based Total Sanitation) ? Is there a dedicated budget for it?

Participant: Regarding dedicated funding, after Plan’s activities, we also allocated funds for related work. We collaborated with Plan, including [named program/local staff member], and with the health center to reduce stunting. We have made those efforts, and this year the budget was 10 million rupiah for rewards for healthy babies. The aim was to motivate mothers to care for their babies from birth onward. In the future, we will continue to allocate approximately five to seven million rupiah, depending on the activities we manage.

Interviewer: That is excellent. Thank you very much,Mr. [Participant] . This is the first time I have heard about rewards for healthy babies in connection with stunting. Could you explain the relationship between water and sanitation and reducing stunting?

Participant: The relationship is very strong, Ma’am. Water facilities are closely connected to the issue. When water is unavailable, it can contribute to stunting. When water flows reliably, it is possible to reduce the stunting rate.

Interviewer: How can poor sanitation affect stunting, Mr.[Participant] ?

Participant: The effect is very significant, Ma’am. Previously, [the locality recorded a small number of stunting cases]. After Plan and the health center collaborated with existing stakeholders, particularly concerning the effect of water on stunting, the number declined substantially.

Interviewer: Thank you very much, Mr.[Participant] . This is valuable information about the effects of Plan’s advocacy and intervention in [study village], together with stakeholder collaboration. It did not come from Plan alone, because several parties worked together. Here we can see examples of CCRIW implementation, including a raised toilet seat for people with accessibility needs and a guiding route. A handrail or guide to the toilet does not always have to be made of metal; bamboo can also be used. I believe some households with persons with disabilities or older people may have made similar modifications. I will ask about this shortly.

Participant: In your opinion, is CCRIW suitable for villages, especially [study village], or is it not appropriate?

Participant: In my opinion, Ma’am, it is suitable. Our village has [a small number of persons with disabilities]. For people with mobility impairments, particularly those affecting the legs, the village has facilitated the provision of walking sticks. For people who are blind, we have provided...

Interviewer: Is there a guiding path like this one? [Referring to an illustration.]

Participant: There is no guiding path, Ma’am, but they use walking sticks.

Interviewer: Have any toilets been modified like this, Mr.[Participant] ? Is there a raised seat on the toilet?

Participant: There is no raised seat, Ma’am.

Interviewer: What about using a rope as a guide to the toilet?

Participant: There is none in the households of persons with disabilities.

Interviewer: What about at the village office?

Participant: We have modified the village office. Previously, there was no supporting wall. We therefore constructed one so that persons with disabilities could use it for support.

Interviewer: Thank you very much. Everything you have described so far has been positive. On a scale from one to five, where one means not satisfied and five means very satisfied, what score would you give the CCRIW program?

Interviewer: How satisfied are you with its implementation?

Participant: Plan’s activities have greatly broadened our thinking, particularly within the village. We implemented the activities, and they have proven useful and effective. Outside parties appreciate them, and the community is proud, especially those of us in the village. I would say that I am very satisfied.

Interviewer: So you give it a score of five?

Participant: Yes, a score of five.

Interviewer: [Laughing.] Thank you very much.

Interviewer: The village and its residents have adopted the CCRIW program. What are the next steps for ensuring its continued success? Achieving something may be relatively easy, but maintaining it is difficult. What will residents or the village government do to sustain the program’s success?

Participant: The village will continue to promote CCRIW, particularly clean environmental sanitation, comfortable surroundings, and an environment that is welcoming to everyone. We will cooperate with the health sector, which has consistently supported the village.

Interviewer: Do you still need assistance, specific guidance, or integration with other government programs to sustain the results?

Participant: As the village government, we never reject positive activities. We are very grateful and welcome any beneficial activity that enters the village.

Interviewer: Let us return to financing,Mr. [Participant] . The village has adopted CCRIW. Where does the funding come from? I am curious. [Laughing.] Has there been a financial impact on [study village], such as funds being diverted from another purpose or depleted because of Plan’s intervention?

Participant: The village received a performance reward of more than 100 million rupiah from the ministry, and we used that funding.

Interviewer: So the focus was sanitation?

Participant: Yes, during the last two years.

Interviewer: Sanitation that also had to be climate-resilient, correct? You have demonstrated this through tree planting, construction of the path, and modifications to the village office.

Interviewer: Mr.[Participant] , the source of funding was not fully clear. Where did it come from?

Interviewer: So the funding came from a government performance reward?

Participant: It was called a performance allowance or reward. Of 125 villages, 27 received it.

Interviewer: Does that mean the reward was given because the village performed well?

Participant: I am not entirely sure what criteria were used, Ma’am. Perhaps it was because we were diligent in providing data and responded quickly with information. However, I do not know which aspect of performance the Ministry of Home Affairs assessed.

Interviewer: All right, let us continue,Mr. [Participant] . Plan’s involvement is nearing its end. What approach will you use to ensure that CCRIW activities continue without Plan’s intervention?

Participant: We have a mandatory clean-Friday activity every Friday. In addition to the sanitation activities, we visit each spring...

Participant: ...to maintain it, and we monitor the tree seedlings that were planted so that the springs remain protected.

Interviewer: That is excellent. There are three springs in [study village]?

Participant: Yes, three springs that are still active.

Interviewer: Can [study village] also help residents of other villages through those springs?

Participant: Two of the springs remain active, but their water is primarily used for rice-field irrigation, particularly in [local area].

Interviewer: If the water is used for rice fields, does that mean the discharge is large?

Participant: Not necessarily, Ma’am. The area in [local area] uses a spring in [local hamlet]. During the dry season, it is particularly important for horticultural crops.

Interviewer: So it serves as an alternative water source.

Participant: [Study village] itself receives water from PAM(regional water utility).

Interviewer: Is it managed by PAM(regional water utility), but does the source come from this village?

Participant: No, the source is outside the village, Ma’am, because...

Participant: ...the spring is at the lowest point, while the settlement is on higher ground.

Interviewer: All right. Regarding the environmental and technical factors, does the village have a water-management structure such as OPAM(Community Based Water System), or an STBM(Community Based Total Sanitation) team? Within either organization, are vulnerable groups included as staff members or invited participants?

Participant: There is no standing organization, Ma’am. Organization occurs during customary or cultural ceremonies, when customary elders take the leading roles. There is no organization that operates daily or weekly.

Interviewer: Since water is managed by PAM(regional water utility), is there an STBM(Community Based Total Sanitation) team in the village?

Participant: No, there is not.

Interviewer: Does support therefore come from the health center?

Participant: Yes, from the health center.

Interviewer: Does the village provide funding to facilitate the team when its members conduct field visits?

Participant: As far as I know, the village has not provided such funding, Ma’am.

Interviewer: Thank you very much, sir.

Interviewer: How are vulnerable groups, such as persons with disabilities, involved in decisions about water and sanitation? Are they invited to meetings, and what form does their involvement take?

Participant: They are involved very frequently, Ma’am. We involve them in all activities, not only Plan activities. Pregnant women, persons with disabilities, posyandu (Integrated Health Center) cadres, and PKK(Family Welfare Empowerment) representatives are all invited.

Interviewer: Did this begin after Plan entered, or had it already occurred before Plan? To be honest, where I come from, persons with disabilities were rarely involved...

Interviewer: ...until disability issues were raised and people became more aware of the need to include them. What happened here?

Participant: We had already involved them before Plan’s activities, Ma’am. Plan’s work strengthened the practice, and we have continued it until now.

Interviewer: When they are involved, how do they feel? From what you have observed, do they become more confident in expressing their feelings and opinions because they feel that they are receiving attention?

Participant: In every activity, Ma’am, we require persons with disabilities or...

Participant: ...pregnant women to present the proposals and needs they have. We prioritize what they propose.

Interviewer: Thank you very much. I do not doubt that, because you have demonstrated it by modifying sanitation facilities, particularly at the village office.

Interviewer: Next, Mr. [Participant] , has there been support from other government institutions or organizations for water and sanitation, particularly support based on climate resilience?

Participant: The government support is what I mentioned earlier. After Plan’s STBM(Community Based Total Sanitation) activity proposed construction of a footpath in [local hamlet], the local government saw that it was beneficial and then provided tree seedlings.

Interviewer: All right, thank you.

Interviewer: Have you coordinated or communicated with other government officials, such as subdistrict or district officials, about water and sanitation issues, particularly during climate-related disasters?

Participant: We have made those efforts, Ma’am. Recently, the village head...

Participant: ...the subdistrict head, and [several customary elders] from [local area] consulted the regent about increasing the water supply in [study village] by constructing a small reservoir.

Participant: What...

Interviewer: Oh, was there a plan to construct a small reservoir?

Participant: It was implemented in 2024, and for the time being...

Interviewer: Has the reservoir been used?

Participant: Yes, it has. Actually, it was not a reservoir, Ma’am. I apologize. It was the repair of a dam.

Interviewer: Okay.

Participant: The irrigation system has been repaired. That was the water-related work.

Interviewer: So the coordination and communication with the local government were successful.

Interviewer: You mentioned the tu’a golo, or customary elders. I would like to ask about individuals or key figures who hold authority in the community and have the capacity to influence water and STBM (Community Based Total Sanitation) services that are accessible to everyone. How are these key figures involved?

Participant: Regarding key community figures, particularly the [customary community leader], I will be honest, Ma’am. They do not manage daily activities or regulate everything on a day-to-day basis. Their role becomes useful during customary ceremonies or [local customary practice/place] and other cultural events, when...

Participant: ...they are called upon for specific purposes.

Interviewer: Let us continue to the question of vulnerability. We have discussed vulnerable groups. When a disaster occurs in [study village], such as drought, a landslide, or flooding, how do vulnerable groups obtain water and access sanitation?

Participant: So far, the water supply in [study village] has never stopped. The water sources are very close to each area, and the access roads are good, so I do not believe vulnerable groups face a problem.

Interviewer: So there have been no reports of persons with disabilities having difficulty accessing clean water. What happens when it rains and, for example, a person’s toilet is far from the house? Have you encountered such an experience?

Participant: During Plan’s activities, persons with disabilities acknowledged that when the weather is unfavorable, some need assistance to reach the bathroom. To be honest, in [study village], there is a person with a disability who cannot stand. That person has motorcycle-repair skills but needs assistance because they cannot stand.

Interviewer: So someone already assists the person. Thank you very much, Mr.[Participant] . Could you explain the community’s awareness of climate change? In [Study City], it may rain and then quickly become hot. How does the community view these changing conditions?

Participant: In [study village], we have implemented STBM(Community Based Total Sanitation) activities. Most recently, we constructed the footpath and wastewater drainage channels from the settlement. We have also gradually made efforts to reduce dengue fever, as recommended by the health center.

Interviewer: That reflects strong collaboration among the village government, the subdistrict, the health center, and the community, Mr.[Participant] .

Interviewer: Are there traditional practices in the village that help address climate-related hazards?

Participant: Yes. Each year, every gendang community holds penti, a customary thanksgiving festival. The ceremony proceeds from the gendang to the water source. It preserves ancestral traditions and [Study City] culture.

Interviewer: Is that practice specifically related to water?

Participant: Yes, it is intended to ensure that water remains available and that the flow remains strong.

Interviewer: Thank you very much. I was going to ask whether individuals or groups face barriers based on their abilities, but that question has already been answered.

Interviewer: Finally, Mr.[Participant] , how would you describe the community’s general attitude toward climate change now?

Participant: Regarding climate change, the people of [study village]...

Interviewer: Have they been able to adapt?

Participant: Yes, they have adapted, and they have made their best efforts in doing so.

Interviewer: Have they participated in tree planting, Mr.[Participant] ?

Participant: Yes. In addition, people no longer burn land carelessly.

Interviewer: This is my final question, Mr.[Participant] . What concerns do community members or village and subdistrict officials have about facing climate hazards or climate change?

Participant: We have responded to those concerns by protecting the springs. If the PAM (regional water utility) water service used every day is interrupted, residents can return to those spring locations. We prepared the three springs well in advance so that they remain active and continue to flow.

Interviewer: Thank God. The concept, the stories, and the positive experiences you have described are excellent. Were there any barriers or negative experiences that were unacceptable?

Participant: I do not think there were any, Ma’am.

Interviewer: Thank God. Those are all my questions,Mr. [Participant] . Thank you very much. Is there anything else you would like to share that I did not ask about?

Participant: I think that is enough, Ma’am. Perhaps I can share my impression.

Interviewer: Yes, please. Thank you very much, Mr.[Participant] .

Participant: Plan’s activities have been very helpful. They have changed our mindset, particularly within the village. Plan’s presence has encouraged continued change. Third, I hope Plan’s future activities will continue so that matters the village has not yet resolved can receive further support and Plan can continue to open our hearts and minds.

Interviewer: Thank you very much, Mr.[Participant] . Is that all?

Participant: Yes.

Interviewer: Thank you very much for your time and ideas. We will summarize them and submit the results to Plan.

Interviewer: Thank you, Mr.[Participant] . May I end the interview?

Participant: Yes.
