# Supplementary material for: Implementing a climate-resilient and inclusive WASH guidance in Indonesia: A mixed-methods case study
Source: PLoS One. 2026 Sep 28;21(9):e0359266. doi: 10.1371/journal.pone.0359266 (PMC13618932; doi:10.1371/journal.pone.0359266)
Supplement: Supplementary file 6 [file pone.0359266.s006.zip › S6 Appendix Transcripts/KII-21_Deidentified_English.docx]

Interviewer, Participant

Interviewer: For this interview, [participant] is speaking not as a member of the health-center team, but as an STBM(Community Based Total Sanitation) facilitator.

Participant: Yes, yes.

Interviewer: As a facilitator. When answering, please speak in your role as a facilitator rather than as health-center staff. May we begin? First, I would like to confirm your full name. Is it simply [participant], or is there another name?

Participant: Just [participant].

Interviewer: All right. What is your current position at the health center?

Participant: Sanitarian.

Interviewer: At [local health center]?

Participant: Yes, that is correct.

Interviewer: How long have you worked as a sanitarian?

Participant: I am [a long-serving environmental-health worker at a local health center].

Interviewer: Thank you. Although you have already completed the consent form, I need to read the procedure again because the interview is being recorded. My name is [interviewer], and I am a researcher from [research institution]. This study aims to identify the factors that influence climate-change responses in the implementation of inclusive water, sanitation, and hygiene, or STBM(Community Based Total Sanitation), programs in Indonesia. The study will improve our understanding of the factors that enable or hinder climate-resilient and inclusive STBM (Community Based Total Sanitation)services and decision-making in village program planning. Today, we will discuss community experiences with inclusive and climate-resilient water, sanitation, and hygiene, including waste management, food hygiene, and wastewater management. Please keep these STBM(Community Based Total Sanitation) components in mind when answering. During the discussion, we will use the terms extreme weather events and climate hazards interchangeably. When I refer to climate hazards, please think of floods, landslides, drought, and sea-level rise. Inclusive water and STBM(Community Based Total Sanitation) facilities are facilities that everyone can access. I will write this down as a reminder during our discussion. Please continue to keep this concept in mind. We are interested in your experiences and perceptions. The interview will last approximately one hour and will be recorded. The audio recording will be stored securely while in use, transcribed, retained for one year, and then destroyed.

Participation in this study is entirely voluntary. You may decline to participate, skip any question, or stop the interview at any time without consequence. Your identity will be protected, and any information you provide will remain confidential and will not be shared with anyone. Please tell us if you need any accommodation to participate comfortably. Thank you for participating. Honest answers will help us provide better recommendations regarding the program procedures. All of your input will remain confidential. We will not share your responses with anyone, and your answers will not affect whether the village receives a grant or another program in the future.

This is the consent form that you need to read and sign. I previously sent it to you online. I have started recording the interview. For the first section, I would like to ask whether you live here, how long you have lived here, and what your role is in this village.

Participant: What do you mean?

Interviewer: You serve as a facilitator in [study village], correct?

Participant: Correct. I am a facilitator, but I do not live there.

Interviewer: All right. What is your role in [study village]?

Participant: I serve as a facilitator.

Interviewer: As a facilitator, have you participated in inclusive and climate-resilient STBM(Community Based Total Sanitation) activities?

Participant: Yes.

Interviewer: Could you provide examples of the activities you attended and explain your role in them?

Participant: The activity was climate-resilient STBM(Community Based Total Sanitation) triggering, which included a climate-resilience risk assessment. As a facilitator, I facilitated residents of [study village]. We conducted four activities. First, we carried out climate-sensitive community mapping. We asked residents to map climate-sensitive and hazard-prone places, identify affected locations, examine how climate hazards affected water, sanitation, and the five STBM(Community Based Total Sanitation) pillars, and consider the effects on vulnerable groups, including children, pregnant women, older people, women, and persons with disabilities. Second, we assessed how climate change affected WASH accessibility, including routes to sanitation facilities and water bodies during climate events, the comfort and safety of the facilities, whether residents felt safe using water and sanitation facilities, and whether backup facilities were available during disasters. We considered how climate change affected use of and access to water and sanitation and whether supporting infrastructure was available. Based on the problems identified, we discussed actions that households, communities, and local government could take. We also identified the effects of climate change on gender equality by examining workloads and changes in decision-making before and after climate events. Fourth, we mapped resources and developed a future vision. We documented five types of resources: human, social, physical, financial, and natural resources. We then discussed how the community could use those resources and invited residents to envision conditions twenty years in the future, such as being free from tidal flooding or drought. They identified what could be done during the first five years and through the twentieth year to reach that vision. Finally, we prepared action plans at the household, community, and local-government levels. Those are the activities I conducted as a facilitator.

Interviewer: Thank you. That was very comprehensive. What was your impression after participating in the activity?

Participant: It was extremely useful. Community members had never considered how climate change occurred or how it might affect their access to water and sanitation. When we invited them to prepare action plans, they identified many priorities. Thankfully, some of the plans were funded through this year's village budget. In [study village], these included establishing a final waste-disposal site, constructing drainage, building safe toilets, and planting mangroves with support from both higher levels of government and the village government.

Interviewer: In your view, are water and sanitation activities related to climate change suitable for implementation at the village level?

Participant: They are very suitable because action should begin at the village level. Disasters are partly caused by human behavior, such as cutting down trees, disposing of waste carelessly, burning forests, and encroaching on forest land. These behaviors contribute to climate change, so climate-change activities are highly appropriate at the village level.

Interviewer: On a scale from one to five, how satisfied are you with this program?

Participant: Five.

Interviewer: Why did you give it a five? Was there any part of the program that you could not accept?

Participant: We identified the locations where climate change occurred or caused impacts, visited the field to observe them directly, and prepared action plans. Another important factor was financial support. Without that support, we could not have carried out these activities on our own.

Interviewer: So, climate-resilient water and sanitation have been adopted in [study village]. Have they been implemented, for example through modifications to toilets or water facilities?

Participant: For water services, the clean-water distribution network has been expanded. For example, [local hamlet] does not have a spring, so water is supplied from another hamlet and the network has been extended. A team also visited [local hamlet], and the village budget allocated funding for clean-water facilities. A final waste-disposal site has been included in the village plan with support from the public works agency. The public works agency also provided fifty safe toilets, and drainage construction has been implemented through village funds.

Interviewer: So those measures are climate resilient?

Participant: Yes, they are climate resilient.

Interviewer: In your opinion, what is the most effective strategy for encouraging government implementation of this program?

Participant: The strategy is to approach the village government, religious leaders, community leaders, and youth leaders in the village.

Interviewer: The budget has already been incorporated through village funds and other support. Do you have suggestions for implementing climate-resilient water and sanitation more cost-effectively?

Participant: To reduce costs, we can approach community, religious, and youth leaders so that everyone works together to address climate impacts. For example, there is extensive forest encroachment, so trees need to be replanted. We have not yet done that. Mutual cooperation does not require much money. Tree planting may require cooperation with the agriculture office to obtain seedlings. Mangroves can be planted to address tidal flooding. A final waste-disposal site is also being planned. If everyone works together, the required budget can be minimized.

Interviewer: During the training with Plan, what did you learn? Please explain generally.

Participant: We were trained to become facilitators. We learned how to identify village conditions and the disasters occurring in a village. During the training, we went directly into the field to identify hazards, determine their effects on water and sanitation, and understand community characteristics. I understood the training provided by Plan. I think the activities we conducted were adequate, although there were many activities and they required considerable time. The triggering was originally planned for two days, but we engaged the community until late in the day so that it could be completed in one day.

Interviewer: What challenges did you encounter when conducting the workshop as trained by Plan, and what improvements would you suggest?

Participant: I am not sure what the challenges were.

Interviewer: Were there any challenges in repeating the training with the community? Do you have suggestions for improving the training previously delivered by Plan?

Participant: Because this was new to me, the main challenge was that I did not initially understand how to conduct it. We had to study first and prepare summaries of what we would do. One useful addition might be videos. So far, we have only been provided with modules to study.

Interviewer: Is there an approach that could help water and climate-resilient STBM(Community Based Total Sanitation) activities continue in the village?

Participant: Water and sanitation involve behavior change, so we should conduct routine monitoring, perhaps once a month. When communities are visited frequently, they feel noticed and become more willing to change. During recent monitoring, we found households that had not adopted appropriate water and sanitation practices, but conditions changed after we visited. The problem is that there has been no operational support. Field visits require water and fuel. Financial support is needed, and monitoring should involve all relevant parties rather than only the health-center team. If everyone consistently discusses water, sanitation, and climate change, the community will be more likely to change.

Interviewer: Could village funds be allocated for monitoring? Perhaps the challenge is that we have not approached the village government sufficiently. The village may not respond strongly if we communicate alone, but coordination from a higher level might be more effective.

Interviewer: Besides funding, is any other support needed?

Participant: Cooperation is difficult unless the relevant parties have made a shared commitment. Once it becomes a joint commitment, monitoring will be easier to conduct.

Interviewer: We will now discuss enabling and inhibiting factors. To your knowledge, are there a water-management group and an STBM(Community Based Total Sanitation) team in [study village]?

Participant: There are water-management groups, called KPSPAM(Community Based Water System), or drinking-water facility management groups. For example, [local hamlet] has a KPSPAM(Community Based Water System).

Participant: It manages a well whose water is distributed across [local hamlets]. There are several community-based water-system groups, each with its own management team.

Interviewer: Is there also an STBM(Community Based Total Sanitation) group?

Participant: Yes, there is a village STBM(Community Based Total Sanitation) team.

Interviewer: Can residents who are not members of the water-management group participate in water management?

Participant: Participate in what sense?

Interviewer: In village water management.

Participant: I think they do participate. For example, when a pipe leaks, residents promptly report it to the management group and work together to repair it. Women also help because they often have the greatest need for water. When water is scarce, women are usually the first to report the problem to the water managers or neighborhood leaders. Children and older people may not participate directly, and participation by persons with disabilities is also limited.

Interviewer: Regarding the STBM(Community Based Total Sanitation) team, can other community members participate in STBM(Community Based Total Sanitation) activities?

Participant: Yes, they certainly can. STBM(Community Based Total Sanitation) activities cannot operate without community participation.

Interviewer: Are women, persons with disabilities, children, and older people also involved?

Participant: Yes. In addition to community-level triggering, we conducted triggering and monitoring specifically with persons with disabilities.

Interviewer: To your knowledge, how do residents of [study village] respond to climate hazards affecting clean water and sanitation?

Participant: Regarding clean water, residents of [study village] still face serious shortages. They are already experiencing drought and have received emergency clean-water assistance from the disaster-management agency. They strongly hope that additional water sources will be developed. In one [local hamlet], piped water is currently supplied only once a month, so households must store enough water for an entire month in large tanks. They want the distribution schedule to be at least once a week so that they have adequate water.

Interviewer: During water shortages, how do the water-management group and STBM (Community Based Total Sanitation)team in [study village] respond?

Participant: They are actually very responsive, but they lack funding. They want to deepen the well to increase the flow, repair or add pumping units, and extend the pipeline network.

Interviewer: You have explained support from the village government. Has the district government provided any additional support to ensure that clean water and sanitation remain accessible during climate hazards?

Participant: The district disaster-management agency has provided support. The KPSPAM(Community Based Water System) systems in several [local hamlet] areas also received government support through cooperation with the electricity provider and public works agency. KPSPAM(Community Based Water System) systems require substantial funding.

Interviewer: When a climate disaster occurs, access to water therefore requires collaboration between the community and government. What does that collaboration look like?

Participant: The government provides funding for clean-water facilities, while the community contributes labor through mutual cooperation, such as digging wells and pipeline trenches. Construction funding comes from the government, and the labor is a community contribution.

Interviewer: How do you coordinate with the government? Are there obstacles when coordinating about clean water and sanitation during climate hazards?

Participant: I coordinate with the village and subdistrict levels. Because of my position, I cannot coordinate directly with higher or central levels of government.

Interviewer: I have a few final questions. In [study village], are there influential people who exercise control over water management or STBM(Community Based Total Sanitation)?

Participant: Yes, there are influential people.

Interviewer: Who are they?

Participant: They include the village head, the STBM(Community Based Total Sanitation) chair, [named local actor], the chair of the village consultative body, and other village-government officials. They understand STBM(Community Based Total Sanitation) because they are consistently involved in STBM(Community Based Total Sanitation) activities.

Interviewer: So the village government holds authority over STBM(Community Based Total Sanitation) and water?

Participant: The village government does.

Interviewer: In your view, is there any imbalance in these roles? Does the village government's role affect how water is distributed among residents?

Participant: No. They manage distribution according to each area's schedule.

Interviewer: How are women, children, and persons with disabilities assured access to water?

Participant: Water is distributed according to a schedule, and each household stores water based on its needs. Households include women, children, older people, and other members who are not considered vulnerable. The household members who are able to do so actively collect and store water so that everyone in the household can access clean water.

Interviewer: What challenges do vulnerable groups, such as women, persons with disabilities, and low-income residents, face in accessing clean water and STBM(Community Based Total Sanitation) services during climate disasters?

Participant: When water is scarce and they do not receive an allocation, they must purchase water. That is a major challenge. Currently, the disaster-management agency is providing assistance, but when assistance is unavailable, they have to buy water.

Interviewer: What can be done to remove these barriers for vulnerable groups? Have any efforts already been made?

Participant: Not yet. To my knowledge, there are no specific measures at the village level.

Interviewer: Are there social norms or community values that help ensure that vulnerable groups do not become even more vulnerable?

Participant: No.

Interviewer: How would you describe community awareness of climate change in [study village]? Are residents aware that the climate is changing?

Participant: They are now aware of changes such as prolonged heat and delayed rainfall. Rain that normally begins in December did not begin until January this year. They recognize the change, but previously they did not understand that human behavior contributes to it. They became aware after we conducted the activity.

Interviewer: Are there local practices or forms of knowledge that help communities address climate disasters and water or STBM(Community Based Total Sanitation) crises?

Participant: In several [local hamlet] areas, some households collect rainwater, although not all households do so.

Interviewer: How would you describe the community's attitude toward climate-change risks in [study village]?

Participant: What do you mean?

Interviewer: How do residents respond to the clean-water crisis and its effects on their ability to access STBM(Community Based Total Sanitation) services? Do they treat drought risks as ordinary because they are accustomed to them?

Participant: They tend to treat the situation as normal because they have experienced it for a long time. The coastal area has always lacked fresh water because the groundwater is brackish. Residents are accustomed to buying water for household needs. After they received assistance from another water program, they no longer had to purchase as much water.

Interviewer: Even though they no longer buy as much water, the distribution interval is still very long.

Participant: Yes. The interval needs to be shortened. It cannot be once a month. At minimum, water should be supplied once a week so residents have enough. Storing water for a long time also creates a risk of mosquito larvae if containers are not tightly covered.

Interviewer: As a facilitator, do you have any concerns when decisions are made to reduce climate-change risks?

Participant: I am concerned that tree planting is no longer occurring in several hamlets because people are expanding corn fields. If the corn-growing area continues to expand and tree cover declines, the springs may produce less water. This could contribute to longer dry seasons. There are many related concerns.

Interviewer: Is there anything else you would like to share about your experience with the climate-resilient water and STBM(Community Based Total Sanitation) program?

Participant: My experience?

Interviewer: Is there anything from your experience that you would like to share with us?

Participant: The program brought a major personal change for me. Before becoming involved, I never spoke in public forums. If I had a question, I asked a friend to raise it because I was too embarrassed to speak. After participating in Plan's training and workshops, I became confident speaking in public. I became willing to serve as a facilitator and was invited to facilitate activities in other subdistricts. Plan also trusted me to support STBM(Community Based Total Sanitation) cadres at the district level. That recognition was very meaningful to me.

Interviewer: Thank you. Is there anything else you would like to say about the CCRIW program?

Participant: I think that is enough.

Interviewer: Thank you. I will report the phone-credit reimbursement to Plan, and it will be transferred later. Thank you very much, [participant]. I wish you good health. I will end the interview now.

Participant: Thank you very much.

Interviewer: Goodbye, Ma'am.
