# Supplementary material for: Implementing a climate-resilient and inclusive WASH guidance in Indonesia: A mixed-methods case study
Source: PLoS One. 2026 Sep 28;21(9):e0359266. doi: 10.1371/journal.pone.0359266 (PMC13618932; doi:10.1371/journal.pone.0359266)
Supplement: Supplementary file 6 [file pone.0359266.s006.zip › S6 Appendix Transcripts/KII-22_Deidentified_English.docx]

Interviewer, Participant

Interviewer: Can you hear me clearly, sir?

Participant: The sound here is a little difficult because there is also a meeting about rice distribution.

Interviewer: That is all right. Can you hear my voice clearly?

Participant: Yes, yes.

Interviewer: Thank you for completing the consent form earlier. To save time, I will first read the consent statement. Please listen, and you may ask questions before the interview begins. My name is [interviewer], and I am a researcher from [research institution]. This study aims to identify factors that influence climate-change responses and their implementation in inclusive water, sanitation, and hygiene, or STBM(Community Based Total Sanitation) , programs in Indonesia. The study will improve our understanding of factors that enable or hinder climate-resilient and inclusive STBM(Community Based Total Sanitation) services and decision-making in village or urban village program planning. Today, we will discuss community experiences with inclusive and climate-resilient access to water, sanitation, and hygiene, including waste management, food hygiene, and wastewater management. Please keep these STBM(Community Based Total Sanitation) components in mind when answering. We will use the terms extreme weather events and climate hazards interchangeably. When I refer to climate hazards, please think of floods, landslides, drought, and sea-level rise. Inclusive water and STBM(Community Based Total Sanitation) facilities are facilities accessible to everyone. I will write this down as a reminder during our discussion. We are interested in your experiences and perceptions. The interview will last approximately one hour and will be recorded. The audio recording will be stored securely while in use, transcribed, retained for one year, and then destroyed. Participation is entirely voluntary. You may decline to participate, skip any question, or stop the interview at any time without consequence. Your identity will be protected, and the information you provide will remain confidential and will not be shared with anyone. Please tell us if you need any support to participate comfortably. Honest answers will help us provide better recommendations concerning program procedures. Your input will remain confidential, and your answers will not affect whether the urban village receives a grant or another program in the future. You have already signed the consent form. Because this interview is online, may we proceed? Your name is [participant]. How long have you worked as a local-government official?urban village

Participant: I have worked in this role for several years.

Interviewer: Several years in total. How long have you served in your current role in [study locality]?urban village

Participant: [Several years].

Interviewer: All right. How long have you lived in this village or urban village , and what is your role in the urban village ?

Participant: Please repeat the question.

Interviewer: Do you live in this urban village ? If so, since when, and what is your role here?

Participant: I happen to work in [study urban village ].

Interviewer: Your voice is breaking up. Is the connection poor there?

Participant: Perhaps it is the connection.

Interviewer: Could you move to a place with a better connection? Otherwise, it may affect the recording.

Participant: I will move to another room.

Interviewer: All right.

Interviewer: Hello. Can you hear my explanation now?

Participant: Yes, it is clear now.

Interviewer: I will repeat the question. How long have you lived in this urban village , and what is your role here?

Participant: I live in [home village] and serve as [a urban village official with several years of service] in [study urban village ]. In that role, I conduct outreach and coordinate with neighborhood leaders as partners of the urban village government.

Interviewer: Have you participated in Plan's inclusive and climate-resilient STBM(Community Based Total Sanitation) activities during the past two years?

Participant: We have participated in Plan activities, mainly through outreach meetings and similar events.

Interviewer: I mean activities specifically concerning inclusive and climate-resilient STBM(Community Based Total Sanitation) .

Participant: If I remember correctly, one of my staff attended a climate-resilient and inclusive activity at [local hotel].

Interviewer: What activities have you personally attended with Plan during the past two years, and what was your role?

Participant:urban village As far as I remember, we mainly attended Plan or STBM(Community Based Total Sanitation) meetings.

Participant: I do not remember all the details, but we continued implementing the STBM(Community Based Total Sanitation) movement. The most recent activity I attended was a GEDSI or gender-equality awareness session.

Interviewer: What was your impression after participating in the activity?

Participant:urban village Generally, I would explain Plan International's activity as follows.

Interviewer: Hello, sir. Can you hear me clearly?

Participant: Yes. I apologize.

Interviewer: Thank you. Could you describe your impression after attending the activity?

Participant: We benefited from knowledge about healthy living practices, both as the government of [study urban village ] and as community members. The activity was very useful because it increased our understanding of clean and healthy behavior.

Interviewer: What climate disasters occur in [study urban village ]? Are they droughts, floods, or something else?

Participant: The most common climate-related disaster is seasonal flooding. Heavy rainfall in upstream areas sends water downstream and affects [study urban village ], which lies along the river flow.

Interviewer: Does drought also occur there?

Participant: Drought affects almost all areas in [study district], including during the current dry period.

Interviewer: Have you modified STBM(Community Based Total Sanitation) facilities, such as toilets, to deal with flooding?

Participant: This remains unfinished work for us because many residents live near the river. We have plans, but we face access and implementation constraints. We would also welcome assistance or guidance from Plan regarding the type of modification required.

Interviewer: So, during the past two years, no toilets or other STBM(Community Based Total Sanitation) facilities have been modified for communities frequently affected by flooding?

Participant: No modifications have been made yet.

Interviewer: Has the CCRIW program, or Plan's program during the past two years, influenced STBM(Community Based Total Sanitation) planning or implementation in your urban village ?

Participant:urban village Yes, it has influenced planning and budgeting.

Participant: After STBM(Community Based Total Sanitation) and Plan International became involved, we initiated a mutual-cooperation movement and allocated a small budget through local units for waste patrols. We asked neighborhood leaders to encourage residents to maintain clean and healthy behavior and keep their surroundings clean.

Interviewer: In what year did the waste program begin?

Participant: We developed the waste initiative at the end of 2022 and began implementation in 2023. It continues today.

Interviewer: Besides Plan's CCRIW program, are there other programs that have influenced climate-resilient STBM(Community Based Total Sanitation) activities in your urban village ?

Participant: Our other activities focus on community empowerment. We work with organizations and institutions in the urban village so that Plan's messages can reach residents through education and advocacy. For example, together with the PKK (Family Welfare Empowerment) mobilization team, we provide information on clean and healthy living through posyandu(Integrated Health Post) activities and direct meetings with residents. That is how we follow up on Plan's activities.

Interviewer: Let me confirm again. During the past two years, no climate-resilient toilets, meaning toilets designed to withstand floods, have been constructed. The urban village 's budget has only supported waste patrols.

Participant:urban village Let me explain.

Participant: We would like to understand what an appropriate modification would look like.

Interviewer: In a flood-prone area, for example, a toilet could be elevated. If floodwater previously entered the toilet, it could be raised after the Plan activity so that flooding no longer reaches it. Has anything like that been done?

Participant:urban village Okay.

Participant: That is difficult because floodwater can reach approximately half the height of a house, or about two and a half meters.

Interviewer: I see.

Participant: It may be more realistic to focus on post-flood recovery. If we discuss elevating a bathroom so that water does not enter it, the actual flood level can be as high as two and a half meters and can submerge houses along the riverbank.

Interviewer: So simply elevating the toilet would not be feasible.

Participant: It would be highly impractical.

Interviewer: Relocation might therefore be required. Thank you. I will move to the results and implementation section.

Interviewer: In your view, are CCRIW and Plan's activities during the past two years suitable for implementation at the urban village level?

Participant: I think Plan's program is appropriate everywhere, whether in a urban village or a village. As I understand it, Plan educates residents about managing their surroundings, maintaining cleanliness, and adopting healthy living practices for their families. If a budget is required, the government must allocate it after understanding the benefits of the program. Plan's educational activities are useful in any location.

Interviewer: On a scale from one to five, where one means highly unsuitable and very dissatisfied and five means highly suitable and very satisfied, how would you rate the program?

Participant: If there were a score higher than five, I would give it that score.

Interviewer: Why are you so satisfied with the program?

Participant: Since the STBM(Community Based Total Sanitation) program began, messages about handwashing and defecating in proper places rather than in rivers have reached even remote villages.

Participant: Handwashing became widely promoted, and I believe Plan helped initiate that movement.

Participant: All of these messages are positive and remind people of the importance of healthy living. Plan assists the government in the field. Even when no physical infrastructure is visible, it builds community character and awareness, which are more important because people then remind one another.

Interviewer: Let me confirm that the program from the past two years has not yet been adopted through modifications or construction of toilets or other climate-resilient sanitation facilities.

Participant: Not yet. Plan may provide some budget support, but it has mainly been for outreach activities. We do not know whether Plan has a budget for physical facilities such as toilets, so we do not understand that aspect.

Interviewer: Suppose the program were adopted. What would the urban village need to ensure successful implementation of climate-resilient STBM(Community Based Total Sanitation) ?

Participant: It would require community awareness, cooperation, and government commitment. At present, the government mainly provides education and outreach. Adoption would require a budget. We would need to survey residents' toilets and identify those that are unsuitable. Flood-resistant modifications would still be difficult because so many houses would require work. A more feasible measure may be constructing a protective embankment, as has been done in another area, to prevent river overflow during the rainy season.

Interviewer: So, in your view, rather than modifying sanitation facilities, a protective embankment is needed.

Participant: Exactly.

Interviewer: If the program continues, what could be done so that STBM(Community Based Total Sanitation) becomes more climate resilient and inclusive?

Participant: We should focus particularly on riverbank areas, although Plan works throughout [study urban village ]. Because the topic is climate change, the riverbank requires special attention. This is not a minor issue. We need protective embankments to prevent river overflow during the rainy season, as well as river maintenance. We hope agencies such as the river-basin authority or public works office will act so that annual flooding does not enter residential areas.

Interviewer: Regarding enabling and inhibiting factors, is there a water-management group or STBM(Community Based Total Sanitation) group in your urban village ?

Participant: We established an STBM(Community Based Total Sanitation) group.

Interviewer: Is there an official decree, and can residents who are not named in the decree participate in the group?

Participant: The STBM(Community Based Total Sanitation) group includes several designated people, such as neighborhood leaders and youth-organization representatives. They serve as cadres and implementers who trigger community action and communicate STBM(Community Based Total Sanitation) messages. They coordinate with neighborhood leaders to conduct activities, including mutual-cooperation activities to maintain cleanliness.

Interviewer: Do children, women, young people, persons with disabilities, and older people also participate in the STBM(Community Based Total Sanitation) group?

Participant: Ideally, they should, and that is what we expect in the field. We assess participation through our programs. We assign neighborhood leaders responsibility for maintaining an inclusive, clean environment, not only yards and drains but sanitation more broadly. We also provide education about accessible toilets and other facilities needed by persons with disabilities.

Interviewer: How do residents respond to floods when those floods affect water and STBM(Community Based Total Sanitation) services?

Participant: Responses vary, but people generally blame conditions upstream. Residents here are mostly receiving the consequences of deforestation and land clearing upstream. Corn cultivation has expanded widely in [Study Area 2], and we experience the resulting floods. Flooding does not occur continuously, but when it recurs, there is clearly an underlying cause.

Interviewer: When flooding occurs, how quickly does the STBM(Community Based Total Sanitation) group respond? Do members mobilize?

Participant: Although there is one formal STBM(Community Based Total Sanitation) group, all residents are STBM(Community Based Total Sanitation) actors because outreach is conducted continuously, including every month. When floods occur, residents use their own initiative and the knowledge they have received about healthy living to evacuate and then clean their homes after the flood.

Interviewer: During the past two years, what support has the district government provided to ensure access to drinking water and sanitation during floods? Has flooding occurred while you have served in the urban village ?

Participant: A major flood occurred recently and inundated many houses along the riverbank. The government has not allocated funds for flood-prevention infrastructure. We have only small routine budgets, such as for access roads. STBM(Community Based Total Sanitation) itself has not yet received a dedicated budget beyond waste patrols and monitoring.

Interviewer: During the flood, what did the district government provide to ensure clean water and sanitation, such as toilets?

Participant: That is a very good question and something we overlooked. During the flood, we did not think about providing public toilets, including after residents were relocated. We also did not consider sanitation sufficiently. The assistance provided was mainly basic food supplies, which are common disaster assistance but are not part of STBM(Community Based Total Sanitation) .

Interviewer: Did you coordinate with government officials to address water or sanitation during the flood?

Participant: We received bottled drinking water from government agencies and other community members. However, this discussion has made me realize that future proposals should also request water for bathing and washing, which was not provided, as well as water and facilities for urination and defecation. This is an important issue, and we will record it.

Interviewer: Are there challenges in obtaining government support for flooding in your urban village ?

Participant: The main challenge is government budgeting. Our requests cannot always be implemented immediately. Before the next flood, we will try to coordinate so that the government and disaster-management agency prepare public toilets and water-storage tanks as part of flood-response assistance. I will ask whether they have such facilities.

Interviewer: I have several final questions about individual-level factors. Are there key individuals in the community who hold authority over STBM(Community Based Total Sanitation) implementation in the urban village ?

Participant: As I mentioned, we formally appointed a neighborhood leader who also served as the head of the STBM(Community Based Total Sanitation) group.

Participant:urban village However, we do not want to focus only on the formal appointment. All residents are responsible for STBM(Community Based Total Sanitation) . We assign greater responsibility to neighborhood leaders, who are expected to lead STBM(Community Based Total Sanitation) implementation in their respective areas. STBM(Community Based Total Sanitation) cannot depend on only one or two people, so those groups and leaders must serve as driving forces.

Participant: They become the driving force.

Interviewer: During the flood, were efforts made to ensure access to water for persons with disabilities, children, women, and older people?

Participant: We did not think specifically about that. We only considered the general population under normal conditions.

Interviewer: What challenges did persons with disabilities, older people, women, and children face during flooding?

Participant: Their challenges were generally similar because flooding affects everyone and is difficult to predict. People must save themselves, protect household facilities and possessions, and then address post-flood problems. Recovery after the flood, including psychological recovery and replacement of damaged belongings, should become a government priority.

Interviewer: So, no special measures have yet been developed for persons with disabilities, children, or women during floods.

Participant: We have only provided general education about evacuation steps before flooding.

Interviewer: To your knowledge, are there customary norms or values in [study urban village ] that encourage people to assist vulnerable groups such as children, older people, and women?

Participant: The main norm is mutual cooperation. People are expected to help one another. There is no written rule, but the practice arises naturally from local customs.

Interviewer: Do residents understand that climate change has impacts?

Participant: They are very aware of climate change. However, because we are downstream, there is little we can do to avoid the effects. For example, we cannot directly prevent floods. Much depends on government policies concerning forest management and tree cutting upstream. We mainly experience the consequences.

Interviewer: How would you describe community attitudes toward climate-disaster risks?

Participant: People consider disasters part of normal life, although they are very disturbing. They feel they cannot do much and tend simply to accept what happens.

Interviewer: Are there concerns among the government or community that influence their actions when flooding occurs?

Participant: The government's role?

Interviewer: I mean the concerns of government or community members during a disaster.

Participant: The government is certainly very concerned. There is also a dilemma. Regulations restrict settlement close to river boundaries, but many residents already live there. Government officials change over time, and we cannot simply relocate or evict established residents. We therefore warn residents to remain vigilant during the rainy season and to evacuate quickly if flooding occurs at night. Our concern is mainly expressed through warnings, even though we know that some settlements do not comply with spatial-planning rules.

Interviewer: As a final question, is there another experience related to climate-resilient STBM(Community Based Total Sanitation) and disasters in your area that you would like to share?

Participant: In [study urban village ], especially along the riverbank, floods occur frequently. Household sanitation discharges easily into the river, but the problem is that many houses do not have absorption pits or septic tanks for domestic wastewater.

Participant: Much household wastewater is discharged directly into the river. From the government's perspective, we hope Plan's programs can eventually support physical measures that protect riverbank residents. May I ask whether Plan has physical infrastructure programs, in addition to activities that build community awareness?

Interviewer: That question is outside my authority to answer. From what we have observed, Plan primarily focuses on empowerment and awareness-building through triggering activities.

Interviewer: Is there anything you would like to add about drought in the urban village ?

Participant: Drought does not have a major direct effect on our area because it is not primarily agricultural or plantation land. The weather feels hotter, but there is little economic impact.

Interviewer: Thank you very much for your time. Plan will provide phone-credit reimbursement of one hundred thousand rupiah because the interview used Zoom. I apologize for any shortcomings.

Interviewer: We can end the interview now. Do you have any other questions?

Participant: I would like to ask whether Plan will continue cooperating with us in future years.

Interviewer: We do not yet know whether Plan will remain in [study district]. The current activity is an evaluation of Plan's work, particularly the climate-resilience program implemented during the past two years. Thank you for your time. I need to interview the next area. Assalamualaikum warahmatullahi wabarakatuh.
