# Supplementary material for: Implementing a climate-resilient and inclusive WASH guidance in Indonesia: A mixed-methods case study
Source: PLoS One. 2026 Sep 28;21(9):e0359266. doi: 10.1371/journal.pone.0359266 (PMC13618932; doi:10.1371/journal.pone.0359266)
Supplement: Supplementary file 6 [file pone.0359266.s006.zip › S6 Appendix Transcripts/KII-23_Deidentified_English.docx]

Interviewer, Participant

Interviewer: Hello. Assalamualaikum, [participant].

Participant: Waalaikumsalam.

Interviewer: I apologize for interrupting you during the day.

Participant: That is all right.

Interviewer: Could you confirm your full name?

Participant: [participant].

Interviewer: Where do you currently work?

Participant: At [local health center].

Interviewer: How long have you worked there?

Participant: I am [a relatively new environmental-health worker].

Interviewer: Have you previously held a similar position elsewhere?

Participant: No, I have not.

Interviewer: So, this is your first position in [study village]. With your permission, I will read the consent information.

Participant: Yes, please.

Interviewer: My name is [interviewer], and I am a researcher from [research institution]. This study aims to identify factors that influence climate-change responses in the implementation of inclusive water, sanitation, and hygiene, or STBM(Community Based Total Sanitation), programs in Indonesia. It will improve our understanding of factors that enable or hinder climate-resilient and inclusive STBM (Community Based Total Sanitation)services and decision-making in village program planning. Today, we will discuss community experiences with inclusive and climate-resilient sanitation and hygiene, including waste management, food hygiene, and wastewater management. Please keep these STBM(Community Based Total Sanitation) components in mind when answering.

Interviewer: During this discussion, we will use the terms extreme weather events and climate hazards interchangeably. When I refer to climate hazards, please think of floods, landslides, drought, and sea-level rise. Inclusive water and STBM(Community Based Total Sanitation) facilities are facilities that everyone can access. I will write this down as a reminder during our discussion. We are interested in your experiences and perceptions. The interview will last approximately one hour and will be recorded. The recording will be stored securely while in use, transcribed, retained for one year, and then destroyed. Participation is entirely voluntary. You may decline to participate, skip any question, or stop the interview at any time without consequence. Your identity will be protected, and the information you provide will remain confidential and will not be shared with anyone. Please tell us if you need any accommodation to participate comfortably. Honest answers will help us provide better recommendations concerning program procedures. Your input will remain confidential, and your answers will not affect whether the village receives a grant or another program in the future. This is the consent form. Do you have any questions before we begin?

Participant: No.

Interviewer: For this interview, please answer in your role as an STBM(Community Based Total Sanitation) facilitator.

Interviewer: As an STBM(Community Based Total Sanitation) facilitator, sir.

Interviewer: Specifically for this past year's program. This interview concerns your role as a climate-resilient STBM(Community Based Total Sanitation) facilitator, rather than your role as a sanitarian. Do you live in [study village]? If so, how long have you lived there, and what is your role in the village?

Participant: I live in [staff housing at the local health center], which is located in [study village]. I work at the health center as a sanitarian, and in the community I participate as an ordinary community member.

Interviewer: Have you participated in climate-resilient water and STBM(Community Based Total Sanitation) activities?

Participant: Yes, I have.

Interviewer: Please provide examples of the activities you attended, your role, and what you learned from the program.

Participant: I most frequently participated in triggering activities, including triggering for the five STBM(Community Based Total Sanitation) pillars and for climate-resilient STBM(Community Based Total Sanitation). I also participated in monitoring STBM(Community Based Total Sanitation) activities in the community and clean-water conditions. Because I had no previous professional experience and began working immediately after graduating, these activities were extremely useful for my work as a sanitarian. I could directly observe the different conditions and responses among households. Some accepted the activities, while others expected assistance from our presence.

Interviewer: In your opinion, are climate-resilient water and STBM(Community Based Total Sanitation) activities suitable for implementation at the village level?

Participant: They are very suitable, especially at the household level. They need to be implemented.

Interviewer: On a scale from one to five, how satisfied are you with this climate-resilient water and sanitation program?

Participant: I would give it a five. It has been very helpful, particularly because some households in [study village] have difficulty accessing water during the dry season and face other environmental constraints. The STBM(Community Based Total Sanitation) activity helped residents express their concerns and aspirations.

Interviewer: Was there any part of the climate-resilient water and STBM(Community Based Total Sanitation) program that the community could not accept?

Participant: On average, everything was accepted.

Interviewer: To your knowledge, has [study village] adopted the climate-resilient water and STBM(Community Based Total Sanitation) program?

Participant: Yes, it has.

Interviewer: Okay.

Participant: We conducted climate-resilient STBM(Community Based Total Sanitation) activities and directly surveyed locations considered vulnerable, including places affected by drought and previous flooding. The community has now been exposed to the concept and understands climate-resilient STBM(Community Based Total Sanitation) better.

Interviewer: What strategy is most effective for encouraging implementation at the village level?

Participant: Direct field engagement is more effective. It is better to visit the community, observe conditions, and speak directly with facility owners than to gather everyone in one place only for discussion.

Interviewer: Since the program has been adopted, what steps are needed to ensure its success?

Participant: The subdistrict government needs to be involved. If only health-center staff or familiar local people visit, the activity is less effective. When government officials participate directly, as during recent STBM(Community Based Total Sanitation) monitoring with [subdistrict official], residents respond very positively. They are pleased when their leaders visit and observe conditions firsthand.

Interviewer: Besides active participation by the subdistrict team, are training or special guidelines needed?

Participant: Yes. Updated guidance and new knowledge would be very useful.

Interviewer: Since the program has been adopted, what is the funding source?

Participant: From what I understand, after we met with the village head and [subdistrict official], the village began allocating funds, although implementation was not complete. More recently, the village began reconsidering or budgeting flood-prevention measures for affected areas.

Interviewer: Do you have suggestions for making the program more cost-effective?

Participant: The community should participate through mutual cooperation so that the government does not bear the entire burden. For example, when a drain is constructed, residents can contribute labor by digging or moving stones.

Interviewer: In general, what have you learned from the climate-resilient water and STBM(Community Based Total Sanitation) program?

Participant: One important experience concerned the first STBM(Community Based Total Sanitation) pillar, ending open defecation. When I first arrived, this area had already been declared open-defecation-free. However, I once observed open defecation in [nearby village], which was not one of Plan's pilot locations.

Participant: In that non-pilot village, open defecation still occurred. We went there with [subdistrict official] and the subdistrict team because the village was being verified for the first pillar. We visited households, especially those along the river, and explained that people should no longer defecate openly and described the consequences of continuing the practice.

Interviewer: What did you learn specifically during training for climate-resilient water and STBM(Community Based Total Sanitation)? Did you attend training?

Participant: Yes. I attended training in [Study City] . It gave me experience and new knowledge beyond conventional STBM(Community Based Total Sanitation), including climate issues and disability inclusion.

Interviewer: Were you able to understand Plan's training well?

Participant: Yes.

Interviewer: Is there any aspect of the training that Plan should improve?

Participant: In my opinion, it was adequate.

Interviewer: Were there challenges in participating in the training activities?

Participant: The main challenge was scheduling. Sometimes the health center and Plan held activities at the same time, so I arrived late or had difficulty attending both.

Interviewer: What strategy could ensure that climate-resilient water and STBM(Community Based Total Sanitation) continue in the village, whether or not Plan remains involved?

Participant: Monitoring must continue, at least twice a year. Even without Plan, there should be follow-up monitoring.

Interviewer: What can you personally contribute to these activities?

Participant: As a sanitarian, I can provide education whenever I meet residents. If I see someone littering or engaging in another unhealthy practice, I can advise them not to dispose of waste carelessly, not to defecate openly, and to protect water sources.

Interviewer: Is any support needed to improve sustainability?

Participant: Yes.

Interviewer: What type of support, besides data, is needed?

Participant: Government participation is needed, not only financial support. Officials should join monitoring visits and observe field conditions directly. Communities are very pleased when government representatives visit them.

Interviewer: We will now discuss enabling and inhibiting factors. Is there an organizational structure or group that manages water and STBM(Community Based Total Sanitation) in [study village]?

Participant: An organizational structure?

Interviewer: Are there water-management groups in [study village]?

Participant: Yes. There is a PAMSIMAS(Community-Based Water Supply and Sanitation Program) group that manages water. For STBM(Community Based Total Sanitation)

, we work with posyandu(Integrated Health Post) cadres and involve them in each monitoring activity, so STBM(Community Based Total Sanitation) activities are integrated with the cadres' work.

Interviewer: Does the STBM(Community Based Total Sanitation) group divide roles and responsibilities among community members?

Participant: At present, cadre participation is still limited. Not all cadres can attend activities, and some have scheduling conflicts with other responsibilities.

Interviewer: In the PAMSIMAS(Community-Based Water Supply and Sanitation Program) group, do residents outside the group also share responsibilities?

Participant: As far as I know, only group members perform the roles, such as fee collection and maintenance. Other residents do not participate directly in management.

Interviewer: Are children, persons with disabilities, women, or young people involved in the STBM(Community Based Total Sanitation) or PAMSIMAS(Community-Based Water Supply and Sanitation Program) groups?

Participant: STBM(Community Based Total Sanitation) activities now include persons with disabilities. Even if they are not included in the formal organizational structure, we invite them to activities. PAMSIMAS(Community-Based Water Supply and Sanitation Program) has not involved them directly.

Interviewer: How does the community respond to climate hazards, particularly when they affect water and STBM(Community Based Total Sanitation) access?

Participant: Could you repeat the question?

Interviewer: How does the community respond to climate change, especially drought and its effects on water and STBM(Community Based Total Sanitation) in [study village]?

Participant: The most noticeable climate impacts are drought and flooding. The activities helped residents anticipate these events. Houses affected by short-duration runoff from the mountains have begun constructing temporary barriers. Some households have raised bathroom floors so water does not enter. We also advised residents to deepen dry wells or establish rainwater-storage systems.

Interviewer: When floods or droughts occur, how quickly do the water-management and STBM(Community Based Total Sanitation) groups respond?

Participant: They respond fairly quickly. If heavy rain occurs in the afternoon, they may visit affected households that same evening, assess damage and immediate needs, and help remove water that has entered homes.

Interviewer: What support has been provided by village, subdistrict, or district government to ensure access to water and sanitation during floods or droughts?

Participant: As far as I know, there has not yet been specific support.

Interviewer: So, when flooding or drought affects clean water, no specific village-government assistance has been provided?

Participant: Drought has not yet caused severe water impacts in this area. If one household's well dries, neighbors can still help. The government has not yet provided pumps or other dedicated equipment. During floods, assistance mainly takes the form of community mutual cooperation. Plans for climate preparedness have been discussed. During the climate-resilient STBM(Community Based Total Sanitation) meeting, the village considered budgeting for a water pump to use during flooding and including it in the action plan, but it has not yet been implemented.

Interviewer: It has not been implemented?

Participant: No, not yet.

Interviewer: As a facilitator, have you communicated with village, subdistrict, or district government about climate-resilient water and STBM(Community Based Total Sanitation) issues?

Participant: I have discussed them with officials up to the subdistrict level.

Interviewer: What challenges arise when coordinating or communicating with village or subdistrict government?

Participant: The first issue raised is usually funding. Officials ask whether a budget is available, and the discussion often stops there.

Interviewer: We will now discuss individual factors. Are there key people who hold authority over water services or STBM(Community Based Total Sanitation)?

Interviewer: Is there someone who effectively controls water or STBM(Community Based Total Sanitation) services?

Participant: Here in [study village]?

Interviewer: Yes, in [study village].

Participant: The key person?

Interviewer: One person.

Participant: Authority is strongest at the subdistrict level. When [subdistrict official] gives direction, the community usually follows. At the village level, authority rests with the village government.

Interviewer: Is there an imbalance of power affecting access to water or STBM(Community Based Total Sanitation) during climate disasters for persons with disabilities, children, older people, or young people?

Participant: Do you mean barriers?

Interviewer: For example, does a person with a disability have difficulty obtaining water, or are women not prioritized?

Participant: Everyone generally has access. During the STBM(Community Based Total Sanitation) assessment with persons with disabilities, they said they did not experience difficulties accessing sanitation facilities, particularly at home.

Interviewer: In your view, what challenges do vulnerable groups, including women, persons with disabilities, and low-income residents, face in accessing water or STBM(Community Based Total Sanitation) during climate disasters?

Participant: During the time I have been here, conditions have generally been safe, and I have not observed such barriers.

Interviewer: Are special steps taken to ensure that persons with disabilities and women can continue accessing water during floods or droughts?

Participant: What type of steps do you mean?

Interviewer: Special measures that vulnerable groups use to access water or toilets while a disaster is occurring.

Participant: If a facility owner has a disability and the toilet is outside, we might recommend adding a roof or temporary shelter during the rainy season so the facility remains usable. So far, access has generally been safe. For persons with disabilities, we recommend simple handrails, which can be made from bamboo, and replacing or covering slippery floors so they are safer.

Interviewer: You have lived there for only about three years. Have you heard of social norms or values that increase the marginalization of vulnerable groups?

Participant: No, I have not.

Interviewer: None?

Participant: The main issue is that persons with disabilities were not previously invited to outreach activities. I have not observed bullying or similar treatment. When they are invited, they are willing to participate.

Interviewer: How would you describe community awareness of climate-change impacts?

Participant: The main local concerns are floods and drought. During the dry season, people prepare by deepening wells. Before the rainy season, they clean and improve drains and raise protective walls so water does not enter their yards.

Interviewer: Are there traditional practices or older forms of knowledge for preventing or addressing climate disasters, particularly those affecting water or STBM(Community Based Total Sanitation)?

Participant: I am not aware of any traditional practice.

Interviewer: How would you describe community attitudes toward climate risks affecting water and STBM(Community Based Total Sanitation)?

Participant: The community's attitude?

Interviewer: Yes, their attitude.

Participant: Regarding water, part of [study village] lies near the coast and has difficulty obtaining water. Some locations are also affected by runoff from the mountains, which causes standing water.

Participant: Areas near the health center are generally safe and experience fewer climate impacts. The more affected area is [local hamlet], where residents feel that the government is paying attention to their environmental facilities. Even without financial assistance, they appreciate officials visiting the location. During those visits, we provide education about water, STBM(Community Based Total Sanitation), flood response, and mitigation measures, such as improving drainage routes so water does not stagnate.

Interviewer: Do concerns among community members or government officials influence their actions to prevent climate-related damage?

Interviewer: Do those concerns affect what they do?

Participant: Could you give an example?

Interviewer: For example, are people concerned that something may happen, and does that concern affect their ability to address climate impacts?

Participant: Government concerns?

Interviewer: Or community concerns.

Participant: Community members have many concerns. They worry about damage to their houses and may not know what to do. I am less familiar with the government's concerns.

Interviewer: Before we close, is there anything else you would like to share about your experience in the climate-resilient water and STBM(Community Based Total Sanitation) program?

Participant: That is approximately all. I gained a great deal of experience from the activities. Water and sanitation work helps the community and also expands my own understanding of community conditions.

Interviewer: Thank you for participating. Plan will provide one hundred thousand rupiah in phone-credit reimbursement. I will first confirm that the interview has been completed, and the reimbursement will be transferred afterward. There is also another study evaluating Plan's overall program. With your permission, one of our colleagues will meet you tomorrow for another interview.

Participant: Tomorrow?

Interviewer: Yes. Are you available?

Participant: I will be working at a primary school until approximately eleven o'clock.

Interviewer: That is fine. The village head will be interviewed first.

Participant: All right.

Interviewer: After the village-head interview, I will ask my colleague to contact you.

Participant: That is fine. Please contact me because I may not be at the health center while I am visiting the school.

Interviewer: Thank you very much, [participant].

Participant: You are welcome.

Interviewer: Assalamualaikum.

Participant: Waalaikumsalam.
