# Supplementary material for: Implementing a climate-resilient and inclusive WASH guidance in Indonesia: A mixed-methods case study
Source: PLoS One. 2026 Sep 28;21(9):e0359266. doi: 10.1371/journal.pone.0359266 (PMC13618932; doi:10.1371/journal.pone.0359266)
Supplement: Supplementary file 6 [file pone.0359266.s006.zip › S6 Appendix Transcripts/KII-24_Deidentified_English.docx]

Interviewer, Participant

Participant: Assalamualaikum warahmatullahi wabarakatuh.

Interviewer: Waalaikumsalam warahmatullahi wabarakatuh. Can you hear me clearly?

Participant: Yes, I can.

Interviewer: Before we begin, could you confirm your full name?

Participant: [participant].

Interviewer: You currently live in [study village]. In which area?

Participant: In [local hamlet].

Interviewer: All right, in [local hamlet], [study village]. What is your current occupation?

Participant: I am a community member.

Interviewer: So, you are a homemaker. Before we begin, have you completed the form?

Participant: Yes.

Interviewer: Before we begin, I will read the consent information. My name is [interviewer], and I am a researcher for Plan's evaluation study. This study aims to identify factors that influence climate-change responses in the implementation of inclusive water, sanitation, and hygiene, or STBM(Community Based Total Sanitation), programs in Indonesia. It will improve our understanding of factors that enable or hinder climate-resilient and inclusive STBM(Community Based Total Sanitation) services and decision-making in village program planning.

Interviewer: Today, we want to learn about community experiences with inclusive and climate-resilient water, sanitation, and hygiene, including waste management, food hygiene, and wastewater management. Please keep these STBM(Community Based Total Sanitation) components in mind when answering. We will use the terms extreme weather events and climate hazards interchangeably. When I refer to climate hazards, please think of floods, landslides, drought, and sea-level rise. Inclusive water and STBM(Community Based Total Sanitation) facilities are facilities that everyone can access. I will write this down as a reminder during our discussion. We are interested in your experiences and perceptions. The interview will last approximately one hour and will be recorded. The recording will be stored securely while in use, transcribed, retained for one year, and then destroyed. Participation is entirely voluntary. You may decline to participate, skip any question, or stop the interview at any time without consequence. Your identity will be protected, and the information you provide will remain confidential and will not be shared with anyone. Please tell us if you need any accommodation to participate comfortably. Honest answers will help us provide better recommendations concerning program procedures. Your input will remain confidential, and your answers will not affect whether the village receives a grant or another program in the future. This is the consent form, which you have already completed. May we begin?

Participant: Yes.

Interviewer: How long have you lived in this village, and what is your role in the village?

Participant: I am in my forties.

Interviewer: So, you were born in this village. What has your role been in [study village]?

Participant: I help many people and also assist with the STBM(Community Based Total Sanitation) program.

Interviewer: What is your role in STBM(Community Based Total Sanitation)?

Participant: I am the chair.

Interviewer: The chair of the village STBM(Community Based Total Sanitation) team?

Participant: Yes.

Interviewer: So, you participated in inclusive and climate-resilient STBM(Community Based Total Sanitation) activities.

Participant: Yes, I did.

Interviewer: Please provide examples of activities you attended and explain your role in them.

Participant: Our first STBM(Community Based Total Sanitation) activity concerned the pillars at the household level.

Interviewer: Okay.

Participant: We held an activity at [local hotel], then traveled to [comparison village] for monitoring. We also monitored each household in our own village, [study village], across its hamlets.

Interviewer: What did you monitor?

Participant: The five STBM(Community Based Total Sanitation) pillars.

Interviewer: What activities did you attend specifically under the more recent climate-resilience program?

Participant: For climate resilience, we visited [local hamlet], which is directly adjacent to the sea, and observed tidal flooding. Tidal flooding occurs in [local hamlet] and also in [local hamlet], so we requested a retaining embankment.

Interviewer: What was your role in that activity?

Participant: I participated as a community member.

Interviewer: You said that you are the chair of the STBM(Community Based Total Sanitation) team.

Participant: In that activity, however, I participated as a community member.

Interviewer: So, the STBM(Community Based Total Sanitation) team was not involved?

Participant: The STBM(Community Based Total Sanitation) team was involved. The subdistrict STBM(Community Based Total Sanitation) team also attended.

Interviewer: Please describe your impression after participating in the activity.

Participant: Alhamdulillah, it was positive. Afterward, the community became more aware of climate change, waste, and open defecation. Much of the waste here ends up in the sea, so residents became aware of its effects. They now frequently conduct mutual-cooperation activities to clean waste from the shoreline. During tidal flooding, that waste enters the settlement.

Interviewer: I will now ask about the results of the intervention. Have you modified or constructed water and STBM(Community Based Total Sanitation) facilities at your home to make them more resilient to climate change?

Interviewer: To be honest, ma'am.

Participant: Our main problem is a lack of water.

Interviewer: What modifications or other measures have been undertaken to anticipate water shortages, particularly at your home or in the surrounding area?

Participant: Here in [study village], it is not merely a shortage. There is truly no water.

Interviewer: Okay.

Participant: People here buy a tanker of water for 250,000 rupiah.

Interviewer: Is one 250,000-rupiah tanker used by one household or shared among several households?

Participant: One household.

Interviewer: How long does one tanker last for a household?

Participant: It depends on the number of household members.

Interviewer: How long does one tanker usually last in your home?

Participant: Including laundry and bathing, it can last one week.

Interviewer: So, approximately 250,000 rupiah per tanker each week. What was the price per tanker again?

Participant: Two hundred and fifty thousand rupiah.

Interviewer: That would be approximately one million rupiah per month.

Participant: More than one million per month. It depends on the family size. Even if there are only four household members, the amount changes when guests visit.

Interviewer: Yes.

Participant: In one month, we are not even certain that we will receive water.

Interviewer: Where does the water come from?

Participant: From [nearby hamlet].

Interviewer: How is it conveyed from [nearby hamlet]?

Participant: Through pipes, using a pump.

Interviewer: Okay.

Participant: We receive water by rotation. Sometimes we receive no water for an entire month because there are many neighborhood units and branches managed by the operators. One line may wait two weeks, so in a given month our turn may not come.

Interviewer: Since the climate-resilient water and STBM(Community Based Total Sanitation) activities began, have any water facilities been modified to increase the amount of water entering the village, such as through a drilled well?

Participant: Not yet, because we have not found a source here. A group from Thailand previously visited to examine a water source and determine how pipes could be connected, but their work concerned [local hamlet]. We have not yet found a source for [study village]. We recently heard that a source may exist south of the village and could be tested, but we have not obtained funding. Perhaps STBM(Community Based Total Sanitation) or Plan could help us identify funding for drilling.

Interviewer: The village experiences tidal flooding as well as water scarcity. When tidal flooding occurs, does it damage sanitation facilities?

Participant: When tidal flooding occurs, sanitation facilities and water-storage areas at houses near the river are affected.

Interviewer: Does water enter only the areas near the river?

Participant: In [local hamlet], the entire settlement is affected because it is very close to the sea. In [study village], sanitation facilities near the river are inundated.

Interviewer: Have toilets been modified so that tidal flooding does not enter them, for example by raising them?

Participant: Some have been raised. Many residents cannot afford to raise them, so we requested a retaining embankment, although it has not yet been provided. We submitted the request first. We also received district special-allocation funding for 80 toilets across [study village]. The recently constructed toilets are elevated and are unlikely to be inundated by tidal flooding.

Interviewer: So, improving the embankment is one modification intended to prevent or address tidal flooding.

Participant: But for...

Interviewer: No measure has yet addressed drought?

Participant: Efforts have been made and continue through the village government and community, but the main constraints are the limited water source and insufficient funding. We held a meeting last night because a possible water source had been found. Each household was asked to contribute 100,000 rupiah toward initial drilling. The managers may allow residents to contribute whatever they can, as long as we can begin drilling. The community strongly hopes that Plan can help identify funding for clean water in [study village]. That is what residents are requesting.

Interviewer: Thank you.

Participant: Clean water is our highest priority.

Interviewer: Is there an organization or group that manages water?

Participant: Yes. We formed an organization with a chair, secretary, and water operator who regulates the distribution schedule for each line. We pay 20,000 rupiah per month.

Interviewer: Are the group's responsibilities shared with the broader community, or does the group alone manage the water?

Participant: The managers are community members.

Interviewer: I mean, does the wider community participate, or does only the group manage distribution and fee collection?

Participant: The management group handles those matters. However, when there is a problem, such as the present lack of water because the source has dried up, the managers hold a meeting with the community. They seek solutions, such as finding another spring. Recently, they searched for another source and planned drilling, but funding remains the constraint.

Interviewer: Do women, persons with disabilities, older people, and young people participate in decision-making within the water-management group?

Participant: Yes, they do.

Interviewer: They participate as well?

Participant: All community members are invited.

Interviewer: Since you chair the STBM(Community Based Total Sanitation) team, are women, children, and persons with disabilities included in STBM activities?

Participant: Yes, they are included.

Interviewer: When facilities are damaged, water becomes scarce, or tidal flooding occurs, how quickly do the STBM(Community Based Total Sanitation) and water-management teams respond?

Participant: As quickly as possible. Once a report is received, they promptly hold a meeting to determine how to respond. Mangroves have already been planted, and another mangrove-planting activity is planned soon.

Interviewer: Where will the mangroves be planted, and who is organizing the activity?

Participant: In [local hamlet] and [local hamlet].

Interviewer: Who organized it?

Participant: The previous activity involved the STBM(Community Based Total Sanitation) team, and [named local actor] also attended. I do not yet know who is organizing the upcoming activity because I could not attend the meeting due to another commitment. I have not yet met the village head to ask. I only know that the activity is planned soon and that a senior local official is reportedly expected to attend.

Interviewer: What government support has been received to ensure clean-water availability and safe sanitation during tidal flooding?

Participant: From the village government?

Interviewer: Support from the district government may also be included.

Participant: For sanitation, the district government provided assistance for 80 toilets, as I mentioned. For clean water, the village government has searched for possible water sources. A source was recently found south of the settlement, and the village contacted the water managers to hold a community meeting, particularly with residents of [local hamlet]. This area does not merely have a shortage...

Interviewer: It has no water at all?

Participant: Yes.

Interviewer: In your view, does climate change place a greater workload on men or women within the household?

Participant: Women bear the greatest burden. We travel far to a well to wash and bathe. If we need water, we leave after the dawn prayer. If we leave late, we will not obtain any because the well dries up. People are already there at four in the morning. If we arrive at six, there is no reason to expect water to remain.

Interviewer: Who usually makes household decisions concerning facility modifications, women or men?

Participant: Since the STBM(Community Based Total Sanitation) activities, we have learned about the roles of women and men in the household. The father is not the only person who has a role. We now make decisions jointly. It is not only the father's or only the mother's decision that must be followed. Everyone has a role.

Interviewer: Did you learn that through Plan's training?

Participant: Yes, through Plan's principles.

Interviewer: Among older people, do older men or older women face a greater workload during drought or tidal flooding?

Participant: During tidal flooding, men and women both have roles. Men work outside the house, for example by blocking an entrance with soil or another material, while women clean the waste and other debris left by the flood. Tidal flooding clearly brings waste into the settlement.

Interviewer: What specific difficulties do women, children, persons with disabilities, and older people face during drought or tidal flooding?

Participant: During drought, everyone struggles to obtain water. Even children help collect water from the well and go there to wash.

Participant: Older women also go to the well.

Interviewer: Okay.

Participant: They must walk several hundred meters because the well is far outside the settlement.

Interviewer: So, older people, children, and women must all leave the village to collect water?

Participant: Yes.

Interviewer: What capacity or support exists to address these conditions, either from the community or from the village or district government?

Participant: What do you mean by capacity?

Interviewer: Support, for example. Is any support available?

Participant: Regarding water shortages, for several weeks we received a free water tanker approximately once a week, from the Red Cross or the government. Each household received only one 100-liter drum.

Interviewer: Is there traditional knowledge about addressing water scarcity or tidal flooding?

Participant: What do you mean?

Interviewer: Did earlier generations or older people teach ways to address tidal flooding or a water crisis?

Participant: In the past, we had piped water from [nearby locality].

Interviewer: Okay.

Participant: Older people often say, “If only we still had the piped water that came from [nearby locality].” It no longer exists.

Interviewer: Why is it no longer available?

Participant: We do not know why the PDAM supply from [nearby locality] disappeared.

Interviewer: So, PDAM water was previously available. Did you personally use that piped water?

Participant: Yes.

Interviewer: Until what age?

Participant: Until I was in senior high school.

Interviewer: Apart from that, did older people teach you how to protect rivers or water sources?

Interviewer: Did you receive any such knowledge from them?

Participant: No. There was no such knowledge from the older generation.

Interviewer: It has disappeared.

Interviewer: On a scale from one to five, where one means no effect and five means a very serious effect, how greatly does climate change threaten water availability?

Interviewer: Five, ma'am.

Interviewer: Why?

Interviewer: The impact is very severe because there is no water.

Interviewer: What about STBM(Community Based Total Sanitation) conditions?

Interviewer: How are STBM(Community Based Total Sanitation) conditions affected?

Interviewer: Does climate change have an impact?

Interviewer: Does climate change affect STBM(Community Based Total Sanitation) conditions?

Interviewer: It affects sanitation.

Interviewer: There is no water.

Interviewer: The sanitation impact also affects STBM(Community Based Total Sanitation).

Interviewer: Have any mitigation or preparedness measures been taken before these events occur?

Interviewer: Are there efforts to prepare before tidal flooding or drought occurs?

Participant: For tidal flooding, as I said, we requested a retaining embankment. For clean-water shortages, if you visit [study village], you will see a large storage tank in front of nearly every house. Some tanks are almost as large as the houses.

Interviewer: Okay.

Participant: We use them to store water whenever our distribution turn comes.

Interviewer: All right.

Participant: We store enough because the next supply may not arrive for a month.

Interviewer: Do you have concerns that influence what you do to address the water crisis?

Participant: Concerns?

Interviewer: For example, are you worried about how you will obtain water during the crisis?

Participant: People here become worried when the calendar approaches September or October.

Interviewer: Even in August, people already worry about how they will obtain clean water.

Interviewer: Okay?

Interviewer: This is my final question. Is there anything else you would like to share about your experience with the water crisis and tidal flooding in this village?

Interviewer: This is not merely my personal experience.

Interviewer: Okay.

Interviewer: It is not merely an experience because it...

Interviewer: truly happens.

Interviewer: Since...

Interviewer: since we were children in [study village]...

Interviewer: until now, when we have children and some people already have grandchildren, we still lack water.

Interviewer: Perhaps this is because [local hamlet] is naturally dry.

Interviewer: Okay.

Interviewer: We do not understand whether it is because we are near the sea or for another reason. In our view, all residents of [local hamlet]...

Interviewer: are severely affected by the clean-water problem.

Interviewer: It greatly burdens the homemakers here...

Interviewer: because we cannot do anything without water.

Interviewer: Even to wash dishes, we use a basin this small.

Interviewer: We have to.

Interviewer: We cannot use a large basin to wash dishes.

Interviewer: Because we must...

Interviewer: conserve water so carefully.

Interviewer: Therefore, my hope, which is also the hope of everyone in [local hamlet]...

Interviewer: is that [interviewer] will continue...

Interviewer: communicating this request to Plan...

Interviewer: so that they understand how [local hamlet]...

Interviewer: truly lacks water.

Interviewer: We recently heard that a spring may have been found, so I strongly hope that...

Interviewer: within the coming weeks...

Interviewer: another party can visit...

Interviewer: our village...

Interviewer: to...

Interviewer: determine whether it is truly a good water source...

Interviewer: and help us identify where funding for its development could come from.

Interviewer: At present, we strongly hope for assistance from Plan.

Interviewer: Yes, ma'am.

Interviewer: All right. I will record everything you have said.

Interviewer: We can submit it, although the decision will be beyond my responsibility. I can only help document your request.

Interviewer: Thank you for your time. That concludes my questions for today. Plan will provide 100,000 rupiah in phone-credit reimbursement.

Interviewer: I will coordinate with Plan regarding the reimbursement.

Interviewer: Thank you very much for your time. Is there anything else you would like to ask or add?

Interviewer: That is enough.

Interviewer: That is all.

Interviewer: We hope that all residents here can receive clean-water assistance.

Interviewer: I heard that drilling may begin tomorrow.

Interviewer: The initial drilling will use funds contributed by the community.

Interviewer: Therefore, I hope Plan can visit our village to examine the spring...

Interviewer: and see how severely people here lack water.

Interviewer: All right. Thank you very much, ma'am.

Interviewer: Stay healthy. Thank you. Assalamualaikum warahmatullahi wabarakatuh.
