# Supplementary material for: Implementing a climate-resilient and inclusive WASH guidance in Indonesia: A mixed-methods case study
Source: PLoS One. 2026 Sep 28;21(9):e0359266. doi: 10.1371/journal.pone.0359266 (PMC13618932; doi:10.1371/journal.pone.0359266)
Supplement: Supplementary file 6 [file pone.0359266.s006.zip › S6 Appendix Transcripts/KII-25_Deidentified_English.docx]

Interviewer, Participant

Participant: Assalamualaikum.

Interviewer: Waalaikumsalam warahmatullahi wabarakatuh. I apologize for interrupting you this afternoon.

Participant: Good afternoon, [interviewer].

Interviewer: Could you confirm your full name?

Participant: [participant].

Interviewer: Where do you currently work?

Participant: I do not have formal employment. I am active in the (organizations) of [study urban village] and serve on the urban village STBM(Community Based Total Sanitation) team.

Interviewer: How long have you been involved in the (organizations)?

Participant: Approximately six years.

Interviewer: Where do you live?

Participant: I live in [study urban village], specifically in [local neighborhood].

Interviewer: Thank you. Before we begin, I must read the consent information as part of the required procedure. My name is [interviewer], and I am a researcher for Plan's evaluation study. This study aims to identify factors that influence climate-change responses in the implementation of inclusive water, sanitation, and hygiene programs in Indonesia. It will improve our understanding of factors that enable or hinder climate-resilient and inclusive STBM(Community Based Total Sanitation) services and decision-making in village program planning. Today, we will discuss community experiences with inclusive and climate-resilient water, sanitation, and hygiene, including waste management, food hygiene, and wastewater management. Please keep these STBM(Community Based Total Sanitation) components in mind when answering. We will use the terms extreme weather events and climate hazards interchangeably. When I refer to climate hazards, please think of floods, landslides, drought, and sea-level rise. Inclusive water and STBM(Community Based Total Sanitation) facilities are facilities that everyone can access. I will write this down as a reminder during our discussion. We are interested in your experiences and perceptions. The interview will last approximately one hour and will be recorded. The recording will be stored securely while in use, transcribed, retained for one year, and then destroyed. Participation is entirely voluntary. You may decline to participate, skip any question, or stop the interview at any time without consequence. Your identity will be protected, and the information you provide will remain confidential and will not be shared with anyone. Please tell us if you need any accommodation to participate comfortably. Honest answers will help us provide better recommendations concerning program procedures. Your input will remain confidential, and your answers will not affect whether the village receives a grant or another program in the future. This is the consent form. To begin, please describe how long you have lived in [study urban village] and your role there.

Participant: I am [a middle-aged, lifelong resident and long-serving community volunteer] in [study urban village].

Interviewer: How long have you been involved in STBM(Community Based Total Sanitation) ?

Participant: I have been involved in STBM for several years.(Community Based Total Sanitation)

Interviewer: So, you participated in inclusive and climate-resilient STBM activities during the last two years?

Participant: Yes, I did.

Interviewer: Please provide examples of activities you attended during the last two years and explain your role.

Participant: During the last two years, we participated in STBM(Community Based Total Sanitation) activities concerning climate-change impacts such as floods, drought, and landslides. Much of [study urban village] lies along a riverbank. Many of its community units are located along the river and are therefore associated with flood risk. The floods are usually not large, although there was a major one within the last five years. As an STBM(Community Based Total Sanitation) member, my role is to advise residents through (organizations) activities, posyandu(Integrated Health Post) activities in particular, religious gatherings, and other community forums.

Interviewer: At your own home, have you modified water or STBM(Community Based Total Sanitation) facilities to make them more climate-resilient and less vulnerable during floods?

Participant: Alhamdulillah, my house is elevated. Although it is along the riverbank, it stands on higher ground than many nearby houses. Under our program, residents in flood-prone areas generally seal drilled wells with concrete so that floodwater does not contaminate drinking and clean water.

Interviewer: What about toilets?

Participant: For toilets, residents sometimes cover the toilet bowl with cloth when flooding occurs. That is one measure people use.

Interviewer: Have riverbank households raised their toilets or made other modifications?

Participant: Some riverbank households have raised their toilets. Others have not because they cannot yet afford it. We continue educating residents about protecting defecation and clean-water facilities.

Interviewer: Have those modifications proven beneficial during floods, or does water still enter the facilities?

Participant: It depends on the weather. In recent years, floodwater usually did not reach residential areas, so the facilities remained safe. When water rises beyond its usual level and enters the neighborhood, some toilets are flooded while elevated ones remain unaffected.

Interviewer: We will now discuss factors that enable or hinder climate-resilient STBM(Community Based Total Sanitation) . Is flooding the only climate hazard in [study urban village]?

Participant: Yes, flooding is the main hazard in [study urban village].

Interviewer: What type of toilet or water infrastructure can remain functional during a flood?

Participant: In recent years, floods have affected clean water. However, flooding in [study urban village] does not last long and the water recedes quickly. We distribute clean water and food when needed. Floods here generally last only two or three hours.

Interviewer: During a flood, are people unable to wash their hands?

Interviewer: What happens after the flood?

Participant: Conditions become disorderly during a flood. After a recent flood, the STBM(Community Based Total Sanitation) team visited to assess conditions. The urban village has completed the five pillars, so we again educated residents about the difficulties they faced after the climate event, such as affected kitchens. The urban village and subdistrict teams also came to assist.

Interviewer: Based on your experience, is there a way for water or toilet facilities to remain usable during flooding?

Participant: There are some measures. Our STBM(Community Based Total Sanitation) team has hoped that appropriate toilets could be provided...

Participant: particularly for riverbank areas.

Interviewer: Okay.

Participant: We also consider how to provide clean water and assist residents so that we can respond when climate-related events occur.

Interviewer: Have any measures been undertaken so that water and toilet facilities remain usable during floods?

Interviewer: In other words, can they still be used while flooding is occurring?

Participant: At present, the main option is to evacuate to neighbors who live on higher ground.

Interviewer: Is there a water-management group in [study urban village]?

Participant: No.

Interviewer: Is there an STBM(Community Based Total Sanitation) group?

Participant: There is an STBM(Community Based Total Sanitation) group and a waste-patrol group.

Interviewer: Please describe the roles of the STBM(Community Based Total Sanitation) team and waste patrol.

Participant: As I said earlier, the STBM(Community Based Total Sanitation) team provides information and education about healthy living. We educate children, pregnant women, and older people, usually through posyandu(Integrated Health Post) because it is a place where community members gather.

Interviewer: How does the community respond to climate change or climate hazards?

Participant: When a climate event occurs in [study urban village], residents ask us for assistance. The STBM(Community Based Total Sanitation) team and urban village government then help residents who are affected.

Interviewer: More specifically, how do residents respond to impacts on water and sanitation?

Participant: We advise residents according to local conditions. We encourage those in vulnerable areas to move temporarily to higher ground, although they later return to the riverbank. We also remind them to maintain cleanliness and not litter, even when no flood is occurring.

Interviewer: Does the community participate in STBM(Community Based Total Sanitation) decision-making, or does the STBM(Community Based Total Sanitation) team decide everything itself?

Participant: We involve residents. We have also included a resident with a disability from [local neighborhood]. Young women, middle-aged women, older women, young men, and other groups participate in decision-making. Before the urban village STBM(Community Based Total Sanitation) team undertakes an activity in an area, we invite residents to meet, usually at the home of an RT(Neighborhood) or RW(Neighborhood) leader. We explain our plans and deliberate together about the neighborhood's priorities.

Interviewer: What support has been received from the urban village or district government to maintain access to water and sanitation during floods?

Participant: The support has mainly consisted of emergency assistance and environmental-health support.

Interviewer: What are examples of that assistance?

Participant: Clean water, food, medicines, blankets, and clothing have been provided. The urban village and subdistrict governments also respond quickly.

Interviewer: Are public toilets available during a disaster?

Participant: There are currently no public toilets. If evacuation is necessary, residents are moved to the urban village office or another location with toilets and related facilities. As I mentioned, floods generally last only two or three hours, and people usually move to higher ground. We do not have public toilets, but we establish a community-funded public kitchen.

Interviewer: Are persons with disabilities and older people ensured access to clean water and other assistance?

Participant: Yes. Assisting persons with disabilities and older people is one of our responsibilities as the STBM(Community Based Total Sanitation) team. We facilitate their access and often work with the youth organization, which is active in [study urban village].

Interviewer: During floods, do older women or older men bear a greater workload?

Participant: Their workloads are probably similar. Older women clean the house with help from older men. We consider people over 60 to be older adults. Middle-aged adults and children also help. Mutual-cooperation work is usually undertaken mainly by middle-aged men.

Interviewer: In your field experience, are decisions about modifying toilets or water facilities generally made by women or men?

Participant: Men.

Interviewer: Men?

Participant: Men generally make those decisions because they earn the household income, but they seek women's opinions. Women also influence household consumption decisions. Physical mutual-cooperation work around the house is generally performed by men.

Interviewer: During Plan activities, did you receive information about gender equality?

Participant: Our most recent activity in [study urban village] addressed gender equality.

Interviewer: Please briefly describe what you learned about gender equality and inclusion from Plan.

Participant: In the most recent gender-equality activity, we invited six groups: young women, older women, middle-aged women, young men, middle-aged men, and older men. From the activity, we concluded that...

Participant: decision-making responsibilities are often concentrated among middle-aged men because they are household heads. However, household heads cannot act without women's involvement. Women and children are also asked for their views, although the final household decision often remains with the male household head.

Interviewer: Between women and men, who bears the greater household workload concerning water and sanitation, especially during floods?

Participant: In my view, women may bear the greater workload because we must clean the house and think about preparing food for the family, although men perform the heavier physical work.

Interviewer: During floods, what specific challenges are faced by women, persons with disabilities, and poor residents?

Participant: Women and poor residents may worry about how to respond because their resources are limited, and they may need to request assistance from the government.

Interviewer: Besides government support, is assistance available from neighbors?

Participant: Yes. Neighbors on higher ground, including people like me, help quickly. As an RT leader, I invite affected residents into my home. Even when I cannot provide material assistance, I can offer them a safe place.

Interviewer: Since childhood, have you heard of traditional practices or knowledge from earlier generations for addressing floods or water shortages?

Participant: From what I understand, flooding occurs partly because accumulated waste obstructs water flow. During the development-planning meeting, residents proposed deepening the river so that rising water would not enter residential areas.

Interviewer: Is there inherited knowledge telling residents what they should do to prevent floods?

Participant: The main lesson is to maintain cleanliness and not throw waste into the river. People also say that trees should not be cut carelessly. Along the riverbank, trees help stabilize the soil and reduce erosion.

Interviewer: On a scale from one to five, where one means no impact and five means a very severe impact, how seriously does flooding threaten water availability and sanitation services in [study urban village]?

Participant: The situation remains relatively safe. Floods have not been severe enough to create a major water shortage.

Interviewer: What number would you assign from one to five?

Participant: What does one mean?

Interviewer: One means that there is no impact.

Participant: I would give it a two because there is some impact, but it is not severe.

Interviewer: Are you certain it is a two?

Participant: I might say two or three, but the impact is not very large.

Interviewer: What about toilets?

Participant: There is a small impact. As RT and RW leaders, when water begins to rise, we warn residents to be careful.

Interviewer: Would the rating be two or three?

Participant: Three.

Interviewer: Okay.

Participant: The impact is moderate, not severe.

Interviewer: Do you have a personal experience in which flooding made it difficult to access clean water or sanitation?

Participant: I have not personally experienced a serious problem. We focus on older residents and assist them first when a flood occurs.

Interviewer: Do concerns or worries affect your ability to act when improving water or sanitation conditions?

Participant: There are always concerns, but they are not overwhelming because residents' hygiene behavior has changed substantially for several years. I am grateful that STBM(Community Based Total Sanitation) exists.

Interviewer: This is the final question. Is there anything else you would like to share about climate-resilient STBM(Community Based Total Sanitation) ?

Participant: As a resident, RT leader, and STBM(Community Based Total Sanitation) team member, I encourage everyone to live healthily, maintain environmental cleanliness, avoid open defecation, continue practicing the five pillars, and handle food hygienically for our collective well-being.

Interviewer: Thank you. Is there anything else you would like to add?

Participant: Assalamualaikum warahmatullahi wabarakatuh.

Interviewer: Plan will provide 100,000 rupiah in phone-credit reimbursement to this number.

Participant: Thank you very much.

Interviewer: Thank you. I will take my leave. Thank you for your time and information. Assalamualaikum warahmatullahi wabarakatuh, [participant].

Participant: Waalaikumsalam warahmatullahi wabarakatuh. You are welcome. Thank you, [interviewer].
