# Supplementary material for: Implementing a climate-resilient and inclusive WASH guidance in Indonesia: A mixed-methods case study
Source: PLoS One. 2026 Sep 28;21(9):e0359266. doi: 10.1371/journal.pone.0359266 (PMC13618932; doi:10.1371/journal.pone.0359266)
Supplement: Supplementary file 6 [file pone.0359266.s006.zip › S6 Appendix Transcripts/KII-26_Deidentified_English.docx]

Interviewer, Participant

Interviewer: Thank you for your time. Could you confirm your full name?

Participant: My name is [participant].

Interviewer: How long have you served as village head?

Participant: I am [a village head with several years of service].

Interviewer: Okay.

Interviewer: How long have you worked in village government? Did you begin only when you became village head, or did you hold an earlier position?

Participant: I began after becoming village head.

Interviewer: So, you had not previously served as a hamlet head, BPD member, or in another village position?

Participant: No.

Interviewer: Have you completed the Google form I sent?

Participant: Yes.

Interviewer: Thank you. I will read the consent information. My name is [interviewer], and I am a researcher for Plan's evaluation study. This study aims to identify factors that influence climate-change responses in the implementation of inclusive water, sanitation, and hygiene, or STBM(Community Based Total Sanitation) , programs in Indonesia. It will improve our understanding of factors that enable or hinder climate-resilient and inclusive STBM(Community Based Total Sanitation) services and decision-making in village program planning. Today, we will discuss community experiences with inclusive and climate-resilient water, sanitation, and hygiene, including waste management, food hygiene, and wastewater management. Please keep these STBM(Community Based Total Sanitation) components in mind when answering. We will use the terms extreme weather events and climate hazards interchangeably. When I refer to climate hazards, please think of floods, landslides, drought, and sea-level rise. Inclusive water and STBM(Community Based Total Sanitation) facilities are facilities that everyone can access. I will write this down as a reminder during our discussion. The interview will last approximately one hour and will be recorded. The recording will be stored securely while in use, transcribed, retained for one year, and then destroyed. Participation is entirely voluntary. You may decline to participate, skip any question, or stop the interview at any time without consequence. Your identity and information will remain confidential. Please tell us if you need any accommodation to participate comfortably. Honest answers will help us provide better recommendations. Your answers will not affect whether the village receives a grant or another program in the future. This is the consent form. Do you have any questions before we begin?

Participant: Alhamdulillah, it is clear enough.

Interviewer: Please describe how long you have lived in [study village] and your role there.

Participant: I am [a lifelong resident and experienced village leader] in [study village]. Before becoming village head, I was involved in managing a village-development program. After that program ended, I participated in village planning and was later elected village head. Since then, our village has received Plan International Indonesia's STBM(Community Based Total Sanitation) program. Our principal problem is waste management. Efforts are often constrained by limited funding, especially when village funds had to be redirected to direct cash assistance during the pandemic. Other STBM(Community Based Total Sanitation) activities have progressed well. Sanitation coverage in the village center has improved, while outlying areas still require alternative approaches for household wastewater, toilets, and other sanitation services. A clean-water drilling and pipeline program is also being implemented.

Participant: I no longer remember the program's name. [The participant described long-term involvement in local programs, continued implementation after external support ended, and later election to a village leadership role.]

Participant: In 2019, we received an invitation concerning an STBM(Community Based Total Sanitation) program...

Participant: I do not remember its exact name, but it concerned water use. It may have been called [earlier water program]. Through that process, we received an STBM(Community Based Total Sanitation) program from Plan International Indonesia in 2019, which continues today. Our most serious problem, as in many other villages, is waste. Efforts to address waste are constrained by insufficient funding. During COVID-19, much of the village budget had to be directed to cash assistance for residents.

Participant: As a result, our waste-management program has remained underfunded. Other programs have progressed without major obstacles. Sanitation in the central area of [study village] is approximately 70 percent complete. For [outlying settlement], we will seek other approaches for household wastewater, toilets, and related sanitation so that coverage outside the center can eventually reach 100 percent. A clean-water drilling project is now approximately 60 to 70 percent complete. The pipeline network is finished, and household meter connections are being installed. STBM(Community Based Total Sanitation) activities in [study village] have generally met expectations. Each year, STBM(Community Based Total Sanitation) activities help update village data and produce valid information. We have tried to implement Plan International Indonesia's programs as effectively as possible. We hope the program continues because it is highly beneficial and needed by residents, particularly because it has helped change household and social behavior.

Participant: Since the STBM(Community Based Total Sanitation) program began, community lifestyles have changed considerably. We hope all parts of the program can continue. Waste remains our greatest concern, and we hope Plan can support [study district] through guidance and technical assistance for waste governance. Such support could provide practical direction for resolving the waste problem. If waste is managed, communities in [study village] and across [study district] will be healthier and better understand the importance of cleanliness.

Interviewer: Thank you for the information. Plan's work had two stages: the five STBM(Community Based Total Sanitation) pillars, followed by inclusive and climate-resilient STBM(Community Based Total Sanitation) . What climate-resilience activities do you remember attending during the last two years?

Participant: Regarding climate resilience, we received information and guidance and connected it to food security. We consider this important because prolonged drought can reduce water availability and agricultural productivity. Most residents are farmers, so we have a responsibility to anticipate climate impacts and support food-security measures.

Interviewer: Does [study village] experience floods, drought, or several types of disaster?

Participant: It has experienced both floods and drought.

Interviewer: Which occurs most often?

Participant: Flooding occurs most often, almost every year.

Interviewer: Okay.

Participant: The current drought is the longest we have experienced. Previous droughts ended earlier, with rain often arriving by late September. This year, rain has still not arrived, and that creates concern about climate resilience.

Interviewer: In frequently flooded locations, are toilets usually damaged?

Interviewer: Has toilet damage occurred?

Participant: Alhamdulillah, recent floods have not affected residents because river flow remains unobstructed and water has not entered settlements. The discharge has not been high. A flood may have entered the settlement around 2018.

Interviewer: So, settlements are generally not affected?

Participant: A flash flood entered a settlement around 2012 or 2018, but it caused no damage.

Interviewer: Your audio is breaking up slightly. Has the community experienced a shortage of clean water for daily use during drought?

Participant: During clean-water shortages, not every household well dries up. In some neighborhood units, privately drilled wells still have water and owners share it with neighbors. One area regularly receives water assistance...

Participant: because an outlying neighborhood has no water source. Residents have now undertaken community-funded drilling, and we plan to cooperate on installing the pipeline.

Interviewer: Okay.

Interviewer: Will the pipeline be funded from the village budget?

Participant: InshaAllah, we will allocate village funds for the pipeline next year.

Interviewer: Did the recent Plan program initiate these improvements, or had the village already undertaken them earlier?

Participant: Even before Plan arrived, we anticipated drought by requesting clean-water deliveries from the disaster-management agency...

Participant: to supply residents.

Interviewer: Has Plan's climate-resilient water and sanitation program strengthened planning to improve water infrastructure?

Participant: Yes. That is one benefit we have received from Plan. During meetings, the facilitator provided guidance on responding to climate resilience and prolonged drought. By following that guidance, drought has not become an unmanageable crisis because we can take several measures to meet residents' clean-water needs.

Interviewer: In your opinion, is a climate-resilient water and sanitation program suitable for implementation at the village level?

Participant: It is highly relevant. [Study district] includes coastal areas, highlands, and villages like ours in intermediate areas. Villages should be given broad opportunities to participate in clean-water and climate-resilience programs.

Interviewer: On a scale from one to five, how satisfied are you with Plan's climate-resilient water and STBM(Community Based Total Sanitation) program?

Interviewer: What score would you give, where five means very satisfied?

Participant: I am very satisfied.

Interviewer: So, five. Why are you very satisfied?

Participant: Previously, we could only respond informally to community concerns about drought and food security and could not fully reach community expectations. This program gave us a clearer framework and enabled us to take concrete action for residents.

Interviewer: What strategy is most effective for ensuring that the program functions in the community?

Participant: In the long term, we need to protect water sources through reforestation and by restoring barren or deforested areas. In the short term, we need concrete measures such as drilled wells. The drilling underway in our village should help address water shortages during the dry season.

Interviewer: Whether or not Plan remains involved, what will be needed for the village to adopt and successfully continue climate-resilient water and STBM(Community Based Total Sanitation) ?

Participant: We hope the state will remain present and support communities in facing climate-related situations...

Participant: across all sectors, and that government at every level will support programs that reach the regions.

Interviewer: Are special guidelines or training needed to ensure the program continues without Plan?

Participant: Yes. Without continued training and specific guidance, the program may gradually fade from community attention. If people regard it as a completed project, implementation may stop. It would be better for the program and support to continue so that communities can sustain what has been learned.

Interviewer: So, training or guidelines are needed. Does your village have a water-management group?

Participant: The clean-water system is not yet complete and measurements are still underway. Once it is operational, we will establish a water-management group because proper governance is essential for a shared water system.

Interviewer: Does the village have an STBM(Community Based Total Sanitation) team?

Participant: Yes, it does.

Interviewer: Does the community participate in the village STBM(Community Based Total Sanitation) team?

Participant: Yes. Our village was among Plan's pilot locations in [study district]. Residents receive information about STBM(Community Based Total Sanitation) data, meetings, and related activities, and that information is then shared more broadly so the program can be implemented according to agreed procedures.

Interviewer: How quickly does the STBM(Community Based Total Sanitation) team respond when the village experiences water shortages?

Participant: The STBM(Community Based Total Sanitation) team responds to community problems, including water shortages, through coordination with village leadership. We provide instructions and information, formulate possible steps, and work with all relevant groups to address the problem.

Interviewer: What support have you received from the district government to make water and sanitation more climate-resilient?

Participant: The regional disaster-management agency supports us with clean-water deliveries every year. This year, the local government has again provided free clean water.

Interviewer: Have you faced challenges obtaining government support or communicating with the district government about water conditions?

Participant: Alhamdulillah, there have been no serious obstacles. When we provide information or submit a letter, the government generally responds well and implements assistance promptly.

Interviewer: Is there a key person who controls STBM(Community Based Total Sanitation) or water management?

Participant: Do you mean in [study village]?

Interviewer: Yes, specifically in [study village].

Participant: There is no single controlling figure. We work collectively. As village leader, I coordinate efforts to address climate resilience and other community problems. When a hamlet lacks water, the village government supplies water from another hamlet or another village for as long as necessary. This service is intended to meet everyone's needs, including persons with disabilities, older people, pregnant or postpartum women, and children.

Interviewer: Has the village established a specific rule to make it easier for women, persons with disabilities, children, or older people to obtain water?

Participant: We do not have a specific written rule, but we respond quickly when persons with disabilities, children, pregnant women, sick residents, or other vulnerable people need assistance. The aim is to prevent the water problem from creating additional social difficulties.

Interviewer: How aware are residents of climate-change impacts? Do they recognize that water shortages and floods may indicate climate change?

Participant: Residents recognize that current conditions differ from the past. We advise them to respond carefully, including when clearing yards or agricultural land, so that fires do not occur. Communities have also responded independently to prolonged drought and its effects on water availability and the economy. So far, the village has managed these conditions without a severe crisis.

Interviewer: Are there traditional practices or knowledge from earlier generations for addressing climate impacts?

Participant: We have not observed harmful or exploitative traditional practices. Conditions have remained orderly.

Interviewer: Do residents face any obstacles when acting to address climate change?

Participant: Alhamdulillah, we have not faced a fundamental obstacle concerning community needs or social and economic problems. Conditions have remained safe and peaceful, and climate change has not created an overwhelming concern.

Interviewer: How would you describe community attitudes toward climate change, particularly water shortages and floods?

Participant: When flooding occurs, residents work through mutual cooperation, help affected households, and provide comfort and assistance. During the current prolonged dry season, there has been no serious conflict or negative response. Community attitudes remain calm and conditions are stable.

Interviewer: As village head, do you have concerns that could affect your ability to address water shortages or flood damage?

Participant: We become concerned when a problem has not yet been resolved, especially when river damage could direct floodwater toward settlements located near the river. In that situation, we make every effort to find resources and address the problem quickly. We have already taken such steps, and problems have generally been resolved promptly so residents feel safe and protected.

Interviewer: Thank you. That was the final question. Is there anything else you would like to add?

Participant: We hope the program continues because [study village] and [study district] still need similar support. Plan cannot solve every problem, but it can make a valuable contribution through assistance and guidance. The program could be expanded as a shared effort to address water shortages, food security, economic concerns, and related problems.

Interviewer: Thank you for your time. Plan will provide 100,000 rupiah in phone-credit reimbursement.

Participant: Alhamdulillah. Thank you.

Interviewer: Thank you. I will end the interview. Assalamualaikum warahmatullahi wabarakatuh. Stay healthy.

Participant: Waalaikumsalam warahmatullahi wabarakatuh. Amen.
