# Supplementary material for: Implementing a climate-resilient and inclusive WASH guidance in Indonesia: A mixed-methods case study
Source: PLoS One. 2026 Sep 28;21(9):e0359266. doi: 10.1371/journal.pone.0359266 (PMC13618932; doi:10.1371/journal.pone.0359266)
Supplement: Supplementary file 6 [file pone.0359266.s006.zip › S6 Appendix Transcripts/KII-27_Deidentified_English.docx]

Interviewer, Participant

Interviewer: Hello. Assalamualaikum, village head.

Participant: Waalaikumsalam.

Participant: I am [a village head with substantial prior village-government experience].

Participant: The sound is clear.

Interviewer: Before we begin, I would like to confirm some information. Could you provide your full name?

Interviewer: Could you help with that?

Participant: [participant].

Interviewer: How long have you served as village head in [study village]?

Participant: [Several years].

Interviewer: How long have you worked in village government overall?

Participant: I began by leading a hamlet, then became a village official, and later became village head.

Interviewer: So, you have substantial village-government experience. How long did you serve in your earlier local role?

Participant: [The participant described several successive local-government roles over more than a decade.]

Interviewer: So, approximately twelve years in government. Before we begin, have you completed the link I sent?

Participant: Yes, I completed it.

Interviewer: Thank you. I will read the consent information again. My name is [interviewer], and I am a researcher for Plan's evaluation study. This study aims to identify factors that influence climate-change responses in the implementation of inclusive water, sanitation, and hygiene, or STBM(Community Based Total Sanitation), programs in Indonesia. It will improve our understanding of factors that enable or hinder climate-resilient and inclusive STBM(Community Based Total Sanitation) services and decision-making in village program planning. Today, we will discuss community experiences with inclusive and climate-resilient water, sanitation, and hygiene, including waste management, food hygiene, and wastewater management. Please keep these components in mind. We will use the terms extreme weather events and climate hazards interchangeably. Climate hazards include floods, landslides, drought, and sea-level rise. Inclusive facilities are accessible to everyone. The interview will last approximately one hour and will be recorded. The recording will be stored securely, transcribed, retained for one year, and then destroyed. Participation is voluntary. You may decline, skip questions, or stop at any time without consequence. Your information will remain confidential. Please tell us if you need any accommodation. Honest answers will help us improve program recommendations. Your answers will not affect whether the village receives a grant or another program. The interview has three parts: general questions, your experience and perceptions of inclusive and climate-resilient STBM(Community Based Total Sanitation) , and closing questions. How long have you lived in this village, and what has your role been?

Participant: After graduating from technical high school, [I served in several successive village-government roles and later became a senior village official].(Village Representative Council)

Interviewer: So, you have participated in inclusive and climate-resilient STBM(Community Based Total Sanitation) activities?

Participant: Yes.

Participant: I am [a long-serving village-government official who later became village head].

Participant: In the first activity, as village leader, I encouraged all hamlet, RT(Neighborhood), and RW(Neighborhood) leaders to participate. We worked with a district team...

Participant: to plant mangroves along the coast in [study village]. The entire coast has not yet been covered because the work is being completed in stages. My hope as village head is that mangroves will eventually be planted along the entire coastline.

Interviewer: What was your impression of the program?

Participant: I hope it continues, especially the mangrove planting.

Interviewer: Besides mangrove planting, have there been monitoring or mapping activities concerning inclusive and climate-resilient STBM(Community Based Total Sanitation) , water, or sanitation?

Participant: Yes. One requirement for achieving STBM(Community Based Total Sanitation) was for the village to become free from open defecation. The village government first assisted many households that did not have toilets. The district later provided substantial additional sanitation support, so only a small number of households in [study village] still lack toilets.

Interviewer: What climate hazards occur in your village, such as flooding or drought?

Participant: Our principal concern in [study village] is when high tide coincides with runoff from the mountains. Our rivers are shallow, so deepening and maintaining them is an important responsibility. Alhamdulillah, the village government has undertaken that work.

Interviewer: When high tide causes water to enter settlements, are sanitation facilities such as toilets inundated or damaged?

Participant: Ordinary high tide is common. The concern is when high tide coincides with water descending from the mountains and causes a much higher overflow.

Interviewer: Has that occurred before?

Participant: Yes, it occurs from time to time.

Interviewer: Have residents modified toilets, such as raising those near rivers or the sea so they are not affected when water rises?

Participant: Residents have done so because they understand the usual height of the tide and prepare accordingly.

Interviewer: Okay.

Participant: Only a small number remain vulnerable.

Interviewer: What is the condition of clean water and daily water supplies in [study village]?

Participant: The village extends over a long area with several hamlets. One area experiences water shortages during a season like this because its source dries up. Residents must wait longer for distribution. The village government has budgeted for replacement pumps when equipment breaks, and we are currently searching for a better water-source location. The problem is mainly the limited source.

Interviewer: So, the pipelines and other infrastructure are not the main problem?

Participant: I do not think there is an infrastructure problem.

Interviewer: During the last two years, Plan has focused not only on the five STBM(Community Based Total Sanitation) pillars but also on water and sanitation that can withstand tidal flooding and drought. Has this climate-resilience focus influenced village planning?

Participant: It has had an influence, and we support all of the activities.

Interviewer: Has the village adopted the program by including it in planning and budgeting?

Participant: Some elements have been included.

Interviewer: What is an example?

Participant: For example, Plan assessed water conditions and related issues.

Interviewer: Then the village budgeted for the pump?

Participant: Yes.

Interviewer: What has Plan itself provided?

Participant: Plan recently began planning and field assessment for a water pipeline in [local hamlet]. The second contribution was mangrove planting. The government will continue maintaining what Plan's team planted.

Interviewer: Okay.

Participant: Along the coast of our village, [study village].

Interviewer: On a scale from one to five, how suitable is Plan's program for [study village], and how satisfied are you, where one means dissatisfied and five means satisfied?

Participant: As village leader, I consider the program very important. Both the village government and community need all of its components.

Interviewer: So, is it suitable?

Participant: It is very suitable.

Interviewer: What satisfaction score would you give, from three to five?

Participant: Three.

Interviewer: Thank you. The village has adapted some Plan activities, such as purchasing a water pump and incorporating planning measures.

Participant: The remaining STBM(Community Based Total Sanitation) issue in [study village] is waste. In 2024, we acquired land for a disposal site. In 2025, we hope to obtain vehicles to collect waste so that [study village] can become cleaner.

Interviewer: Will that be funded by the Village Fund?

Participant: Yes, by the Village Fund.

Interviewer: What district-government support has been received for water and sanitation improvements in [study village] during the last two years?

Participant: The health office has also assisted, including guidance in locating water sources.

Interviewer: Has a new water source been found?

Participant: A source was found with support from the health office, but its yield is limited during a season like this.

Interviewer: What support does the village still need so that climate-resilient water and sanitation can continue?

Participant: We hope to identify reliable water sources. [Study village] has a long coastline, so we also hope mangroves can be planted along the entire coast to reduce direct wave impacts. We also need to address waste. We have prepared a disposal site, and our next task is to obtain a vehicle to transport waste there.

Interviewer: Have you experienced difficulty coordinating or communicating with the district government?

Participant: No.

Interviewer: Okay.

Participant: District officials advised us about selecting a suitable disposal site at least two kilometers from settlements. The village government then identified the landowner, purchased the land, and continued coordinating with the district.

Interviewer: Is there a group that manages the water distributed to residents?

Participant: Each hamlet has a management team with a chair, treasurer, and operator.

Interviewer: Does the community participate in water management?

Participant: Yes. The community formed the group and manages it.

Interviewer: Does the village also have an STBM(Community Based Total Sanitation) team?

Participant: Yes.

Interviewer: Does the community participate in the STBM(Community Based Total Sanitation) team or its activities?

Participant: Yes. Community members participate in cooperation with the village midwife.

Interviewer: Are women, young people, children, older people, and persons with disabilities included in water-management and STBM(Community Based Total Sanitation) activities?

Participant: We involve everyone. During the mangrove-planting activity, even schoolchildren participated.

Interviewer: How do residents perceive climate hazards, particularly in relation to water and sanitation?

Participant: The climate hazards residents fear most are drought and water scarcity in [study village].

Interviewer: Why is drought feared most?

Participant: Drought makes everything difficult. Plants cannot grow, animals cannot drink, and many other problems arise.

Interviewer: Is there a key person who controls water or STBM(Community Based Total Sanitation) management?

Participant: What do you mean?

Interviewer: Is there one respected person whose views dominate the water group or STBM(Community Based Total Sanitation) team?

Participant: Village governance has levels. Each hamlet has a hamlet head, followed by RT leaders. Below the village head are hamlet teams and community members, with chairs, secretaries, and treasurers.

Interviewer: Is there any imbalance in roles and responsibilities, or does everyone contribute?

Participant: Everyone has an assigned role and is prepared to carry it out.

Interviewer: Do the water-management and STBM(Community Based Total Sanitation) groups ensure that persons with disabilities, women, older people, and children can access water and sanitation?

Participant: Yes, we ensure access. At public sanitation facilities, we provide specific signs and access arrangements for persons with disabilities where possible.

Interviewer: What specific challenges do persons with disabilities, women, or poor residents face when obtaining water or using toilets?

Participant: I do not think there is a specific barrier because we try to ensure everyone is accommodated. We also try to ensure that if one household receives water, neighboring households can receive it as well.

Interviewer: Is there traditional knowledge about protecting water sources from drought or anticipating tidal flooding?

Participant: Older people have always advised against leaving the mountains bare and have encouraged reforestation. Much of the area is now planted, although earlier deforestation may have contributed to current drought.

Interviewer: How aware is the community that climate change is occurring?

Participant: Residents appear aware. The entire community has supported tree-planting activities.

Interviewer: Do persons with disabilities or women face barriers when acting to address climate-change impacts?

Participant: No.

Interviewer: As village government, do you have concerns that affect your ability to address climate hazards?

Participant: No.

Interviewer: This is my final question. Is there anything else you would like to share about your experience with inclusive and climate-resilient water and STBM(Community Based Total Sanitation) ?

Participant: Our village was among the first in [study district] to receive STBM(Community Based Total Sanitation) information. Following the program, nearly all hamlets conducted waste cleanups. To keep the village clean, we have prepared a final disposal site and hope to obtain...

Participant: a dump truck next year to collect waste. Alhamdulillah, residents are reducing littering and open defecation, and that is the main change they have experienced so far.

Interviewer: Thank you very much. That concludes my questions. I apologize for any inappropriate wording during the interview. Plan will provide 100,000 rupiah in phone-credit reimbursement because we used Zoom. I will now take my leave because I have another interview. Thank you. I hope I can visit the village another time. I will close the interview.
