# Supplementary material for: Implementing a climate-resilient and inclusive WASH guidance in Indonesia: A mixed-methods case study
Source: PLoS One. 2026 Sep 28;21(9):e0359266. doi: 10.1371/journal.pone.0359266 (PMC13618932; doi:10.1371/journal.pone.0359266)
Supplement: Supplementary file 6 [file pone.0359266.s006.zip › S6 Appendix Transcripts/KII-28_Deidentified_English.docx]

Interviewer, Participant

Interviewer: Hello. Assalamualaikum.

Participant: Waalaikumsalam warahmatullahi wabarakatuh. How are you?

Interviewer: Can you hear me clearly?

Participant: Yes, clearly.

Interviewer: Thank you. Before we begin, could you confirm your full name?

Participant: My name is [participant].

Interviewer: Do you work in the village-government office?

Participant: I am a farmer.

Interviewer: You participated with [named local actor] in climate-resilient water and STBM(Community Based Total Sanitation) activities?

Participant: I participated as a community leader.

Interviewer: Thank you. Before we begin, I will read the consent information. My name is [interviewer], and I am a researcher for Plan's evaluation study. This study aims to identify factors that influence climate-change responses in the implementation of inclusive water, sanitation, and hygiene, or STBM(Community Based Total Sanitation) , programs in Indonesia. It will improve our understanding of factors that enable or hinder climate-resilient and inclusive STBM(Community Based Total Sanitation) services and decision-making in village program planning. Today, we will discuss community experiences with inclusive and climate-resilient water, sanitation, and hygiene, including waste management, food hygiene, and wastewater management. Please keep these components in mind. We will use the terms extreme weather events and climate hazards interchangeably. Examples include floods, landslides, drought, and sea-level rise. Inclusive STBM(Community Based Total Sanitation) facilities are accessible to everyone. The interview will last approximately one hour and will be recorded. The recording will be stored securely, transcribed, retained for one year, and then destroyed. Participation is voluntary. You may decline, skip questions, or stop at any time without consequence. Your identity and information will remain confidential. Please tell us if you need any accommodation. Honest responses will help us improve program recommendations, and your answers will not affect whether the village receives a grant or another program. This is the consent form, which you have already completed.

Interviewer: Thank you. To begin, how long have you lived in this village, and what role have you held?

Participant: I have lived here since birth.

Participant: I am [a middle-aged lifelong resident and former village institutional representative].

Participant: I am approximately fifty years old and have lived here for about fifty years.

Interviewer: You described yourself as a community leader. Please explain your role.

Participant: I previously served in village representative and community-empowerment institutions.

Interviewer: So, you have participated in climate-resilient water and sanitation activities?

Participant: Yes.

Interviewer: Please describe the Plan activities you attended and your role in them.

Participant: Early Plan activities involved collecting information about children, including identifying prospective sponsors and local organizers. More recently, I participated with Plan and the STBM(Community Based Total Sanitation) team in field activities concerning drought hazards and tidal flooding, which has many impacts in our area.

Interviewer: So, you participated directly in the activities?

Participant: Yes.

Interviewer: You mentioned drought and tidal flooding. Have water or sanitation facilities, such as toilets, been modified so they are less affected by drought or sea-level rise?

Participant: During the current season, [study village] has experienced severe drought. Household wells have dried up, and residents must purchase water from outside for drinking and cooking. During tidal flooding, waste is carried into yards and settlements, which creates environmental and health risks. Residents respond through mutual cooperation to clean the waste. If it is left in place, it threatens health because waste collects in drains and around houses.

Interviewer: What type of toilet or drainage infrastructure could withstand tidal flooding and remain usable?

Participant: The drainage system is still inadequate. Besides community cleanups, we hope the government can help construct better drainage. However, even good drainage will not solve the problem if residents continue dumping waste indiscriminately. Waste thrown into rivers reaches the sea and returns to the settlement during tidal flooding. [Study village] does not yet have a final disposal site, so household waste is often discarded carelessly. That is the most critical problem.

Interviewer: What type of water facility would reduce the impacts of drought?

Participant: [Study village] has several hamlets, and many experience serious water shortages. We hope Plan can help us obtain drilled wells. Shallow wells dry up, but drilled wells at several locations have continued producing water during this prolonged dry season.

Interviewer: Could drilled wells be accessed easily by persons with disabilities, older people, children, and women?

Participant: A drilled well uses a pump to lift the water.

Interviewer: Okay.

Participant: From the pump, water can be distributed by pipe to residents' homes.

Interviewer: Tidal flooding and drought occur regularly. What measures has the community taken to address them?

Participant: During prolonged drought, residents can do little except accept the situation and buy water from outside, either bottled water or tanker water. For tidal flooding, we clean drains through mutual cooperation after the water recedes because large amounts of waste remain.

Interviewer: The condition has existed for decades, yet it seems residents have accepted that they must simply buy water when shortages occur.

Participant: That is correct. We cannot do much without assistance from the government or Plan. We especially hope for attention to clean water.

Interviewer: Okay.

Participant: For tidal flooding, the only action available to us is mutual cooperation to clean drains and remove flood debris.

Interviewer: Does [study village] have a water-management group and an STBM(Community Based Total Sanitation) group?

Participant: Yes. There is a water-management group because a clean-water system operates in [study village]. It is still functioning, although supply becomes limited during drought.

Interviewer: Can young people, older people, and women participate in the water-management group?

Participant: Yes, they participate.

Interviewer: What forms does their participation take?

Participant: They can become members of the management group. Everyone also participates in mutual-cooperation activities, which have been especially common recently.

Interviewer: Does the community also participate in the STBM(Community Based Total Sanitation) group?

Participant: Yes.

Interviewer: How does the community respond to drought and tidal flooding, particularly their impacts on water and sanitation?

Participant: What do you mean?

Interviewer: How do residents perceive these recurring conditions?

Participant: Drought is considered normal here. October and November are usually the peak months, and residents prepare to purchase water. It occurs every year, and we have no other solution. For tidal flooding, people have also largely accepted the situation. The only action we can take is mutual cooperation to remove waste and clear blocked drains.

Interviewer: When a water crisis occurs, does the water-management group respond quickly?

Participant: Yes. It reports the situation to the local government. In October and November, two or three donated water tankers may arrive each day. Tidal flooding also affects sanitation.

Participant: The STBM(Community Based Total Sanitation) team quickly asks residents to conduct mutual-cooperation work. It monitors and encourages the community not to remain passive.

Interviewer: What government support has [study village] received for water and sanitation?

Participant: The regional government provides clean-water tanker assistance each year. It has also supported drilled wells or irrigation for dry agricultural land. However, I do not think there has been specific government assistance for tidal flooding beyond community drain cleaning. During the current drought, tankers arrive daily.

Interviewer: During water shortages, do older men and older women bear similar workloads, or do older women carry a greater burden?

Participant: The burden is shared. It is not assigned exclusively to women or men.

Interviewer: Okay.

Participant: Both participate.

Interviewer: Within households, who usually makes decisions about modifying water or sanitation facilities, men or women?

Participant: Usually men.

Interviewer: Why?

Participant: In my household and in many others, men generally make those decisions.

Interviewer: Does male decision-making help ensure a quicker response when water or sanitation problems occur?

Participant: Men generally decide whether to order or purchase water and contact the village government about shortages. Men also take the lead in sanitation-related mutual-cooperation work, although women participate as well.

Interviewer: Have you attended Plan activities concerning gender equality and inclusion?

Participant: Gender equality?

Interviewer: Yes. Have you attended that component?

Participant: I do not think so. I only attended the meeting concerning tidal flooding and climate issues.

Interviewer: Between women and men in the household, who bears the greater workload during drought or tidal flooding?

Participant: As I explained...

Interviewer: For example, does the wife carry more water?

Participant: To be honest, women seem to have the larger role.

Interviewer: When water must be ordered or residents must wait for supply, do women or men make the decision?

.

Participant: From what I observe, women often report that the household water has run out and ask what should be done.

Interviewer: Do older people, persons with disabilities, women, or children face particular challenges during water shortages or tidal flooding?

Participant: Yes. During drought, there is simply no water.

Participant: Older people may no longer have the strength to collect water from another place.

Participant: Persons with disabilities face even greater difficulty, and children cannot collect water independently. Older people and persons with disabilities face the greatest challenges.

Interviewer: What is done so that older people, persons with disabilities, children, and women can obtain water safely and comfortably?

Participant: We hope government water assistance can be delivered in a way that is easy for everyone to access. At present, water may be delivered to a common point and then carried home, which remains difficult for women, older people, and persons with disabilities. Direct household delivery would be easier.

Interviewer: Is there local or traditional knowledge from earlier generations about reducing drought or tidal-flood risk?

Participant: What do you mean?

Interviewer: Did older generations teach any method for reducing those risks?

Participant: No such knowledge exists. During drought, residents have always relied on requesting water assistance from the regional government.

Interviewer: Okay.

Participant: During tidal flooding, the only response has been mutual cooperation.

Interviewer: On a scale from one to five, where one means no effect and five means a very severe effect, how serious are drought and tidal flooding?

Participant: All right.

Interviewer: How severe are they?

Participant: Very severe.

Interviewer: So, five. Did tidal flooding affect your own hamlet?

Participant: Not my hamlet.

Interviewer: Please describe your personal experience with water shortages.

Participant: In the past, we dug a well in a garden and carried water from there. Both men and women did that work. Alhamdulillah, my hamlet now has piped clean water, and supply remains available even during a prolonged dry season.

Interviewer: Is water available every day?

Participant: Yes, in the morning and evening.

Interviewer: So, your hamlet does not face multi-day rotational supply like some other hamlets?

Interviewer: Do you have concerns that affect your actions to reduce drought or tidal-flood damage?

Participant: Yes. Continued drought is unsustainable because water becomes insufficient even for drinking, much less bathing. Residents cannot be expected to buy water every year indefinitely. Through this interview, I ask Plan to communicate with the government so that [study village] receives a lasting solution. We also need government assistance to reduce tidal-flood impacts on settlements. Tidal flooding brings waste and creates health risks. We continue carrying out Friday cleanups, but sanitation and protective infrastructure still need improvement.

Interviewer: Thank you. Is there anything else you would like to share about your experience with climate-resilient water and STBM(Community Based Total Sanitation) ?

Participant: I think that is enough.

Interviewer: Thank you for your time. Plan will provide 100,000 rupiah in phone-credit reimbursement.

Participant: You are welcome. Thank you.

Interviewer: Assalamualaikum warahmatullahi wabarakatuh.

Participant: Waalaikumsalam warahmatullahi wabarakatuh.
