# Supplementary material for: Implementing a climate-resilient and inclusive WASH guidance in Indonesia: A mixed-methods case study
Source: PLoS One. 2026 Sep 28;21(9):e0359266. doi: 10.1371/journal.pone.0359266 (PMC13618932; doi:10.1371/journal.pone.0359266)
Supplement: Supplementary file 6 [file pone.0359266.s006.zip › S6 Appendix Transcripts/KII-29_Deidentified_English.docx]

Interviewer, Participant

Interviewer: Hello. Assalamualaikum.

Participant: Waalaikumsalam warahmatullahi wabarakatuh. My name is [participant].

Participant: Yes, simply [participant].

Interviewer: What is your current occupation?

Participant: I am [a local hamlet leader].

Interviewer: A hamlet head. Which hamlet?

Participant: [local hamlet].

Interviewer: Is [local hamlet] currently experiencing drought or flooding?

Participant: At present, it can be described as drought.

Interviewer: Before we continue, I will read the consent information. My name is [interviewer], and I am a researcher for Plan's evaluation study. This study aims to identify factors that influence climate-change responses in the implementation of inclusive water, sanitation, and hygiene programs in Indonesia. It will improve our understanding of factors that enable or hinder climate-resilient and inclusive STBM(Community Based Total Sanitation) services and decision-making in village program planning. Today, we will discuss community experiences with inclusive and climate-resilient water, sanitation, and hygiene, including waste management, food hygiene, and wastewater management. Please keep these components in mind. We will use the terms extreme weather events and climate hazards interchangeably. Examples include floods, landslides, drought, and sea-level rise. Inclusive facilities are accessible to everyone. The interview will last approximately one hour and will be recorded. The recording will be stored securely, transcribed, retained for one year, and then destroyed. Participation is voluntary. You may decline, skip questions, or stop at any time without consequence. Your information will remain confidential. Please tell us if you need accommodation. Honest responses will help us improve program recommendations, and your answers will not affect whether the village receives a grant or another program. I sent the consent link by WhatsApp. If it has not been completed, a family member may help you complete it after the interview.

Participant: Do I need to complete it before the interview?

Interviewer: No. It can be completed after the interview. We can begin now.

Interviewer: How long have you lived in this village, and what is your role?

Participant: I am [a lifelong resident and local hamlet leader] in [study village].

Interviewer: Have you participated in Plan activities concerning climate-resilient water and STBM(Community Based Total Sanitation) , in addition to the five STBM(Community Based Total Sanitation) pillars?

Participant: I participated in both the five-pillar STBM(Community Based Total Sanitation) activities and the water-related STBM(Community Based Total Sanitation) activities.

Interviewer: What climate-resilient water and STBM(Community Based Total Sanitation) activities do you remember?

Participant: The climate-resilience activity was held in the village, while the five-pillar STBM(Community Based Total Sanitation) activity was held at the district level.

Interviewer: What village activities did you attend?

Participant: We first gathered at the village office with the STBM(Community Based Total Sanitation) team and community members. We received information about preventing and responding to natural hazards. The STBM(Community Based Total Sanitation) team then shared this information with residents. In our area, heavy rain sometimes brings runoff from the mountains into the village.

Interviewer: What did you do during the activity, and what was your role?

Participant: Like the others, I listened and joined the team in field visits. We observed locations vulnerable to erosion and places where heavy rainwater could enter the settlement.

Interviewer: So, the main hazard is flooding rather than household wells drying up?

Participant: Alhamdulillah, household wells have not completely dried up. Their water level declines, but flooding during the rainy season is caused by runoff from the mountain.

Interviewer: What toilet modifications have been made, particularly at your home, to prevent floodwater entering yards and settlements?

Participant: At my own home, the toilet and waste-disposal system have not been inundated. We advise residents in vulnerable areas to raise their toilets so floodwater does not enter them.

Interviewer: So, toilets have been raised?

Participant: Yes, some have.

Interviewer: Has raising the toilets effectively protected them from flooding?

Participant: To some extent. Residents understand the information provided through STBM(Community Based Total Sanitation) training and village field activities.

Interviewer: Were these modifications made during Plan's program or earlier because of previous flooding?

Participant: Some residents had already acted on their own before Plan arrived because they had experienced flooding at several locations. Others made changes after STBM(Community Based Total Sanitation) facilitators and the village government provided guidance.

Interviewer: During flooding or drought, are handwashing facilities still usable?

Participant: Most residents have outdoor taps for handwashing. When flowing water is available, they use those taps.

Interviewer: How are they used during a disaster?

Participant: What we call flooding here is not a large destructive flood. It is runoff from the mountain. Because village development has created more walls and channels, water now flows directly toward the river instead of spreading widely. A few minutes after the rain stops, the water usually disappears.

Interviewer: Are there measures to prevent water from overflowing whenever heavy rain occurs?

Participant: The government generally responds promptly.

Participant: Before the rainy season, residents conduct mutual-cooperation work to clean drains and other channels.

Interviewer: Does the village have an STBM(Community Based Total Sanitation) team?

Participant: Yes.

Interviewer: Can children, women, and persons with disabilities participate in the STBM(Community Based Total Sanitation) team?

Participant: Women are very involved. Children participate to a more limited extent, although junior-high-school students have gained some understanding through STBM(Community Based Total Sanitation) activities.

Interviewer: What about persons with disabilities?

Participant: They are also included whenever we hold meetings.

Interviewer: How quickly does the STBM(Community Based Total Sanitation) team respond to flooding?

Participant: When heavy rain begins, whether during the day or night, we can generally predict when conditions may become serious. Team members become alert, circulate through the village, and check vulnerable locations.

Participant: We coordinate with the village government and other STBM(Community Based Total Sanitation) participants.

Interviewer: Does the STBM(Community Based Total Sanitation) management team make decisions alone, or is the community involved?

Participant: Decisions about what?

Interviewer: Decisions concerning responses to drought or flooding.

Participant: Mutual cooperation remains strong in our community. The STBM(Community Based Total Sanitation) team and residents decide together how to respond, and the team encourages people to take action.

Interviewer: What government support has been received to maintain clean-water and sanitation conditions?

Participant: The government has improved environmental drainage and the main channel that carries water to the river, although the work is not yet complete. Before the rainy season, sediment is removed so water can flow smoothly. For clean water, many households use privately drilled or dug wells. Their protective structures are generally elevated.

Interviewer: So, the wells are high enough to prevent floodwater from entering. During flooding or water shortages in [local hamlet], do older men or older women bear the greater workload?

Participant: Men may undertake somewhat more of the physical response, but women also play an important role. Women often provide the encouragement that leads men to make decisions quickly.

Interviewer: Who usually decides whether to repair or modify water and sanitation facilities, women or men?

Participant: Both.

Interviewer: Both?

Participant: Monitoring results show that ideas and decisions are generally shared. Household sanitation and domestic work are also increasingly regarded as shared responsibilities.

Interviewer: Have you attended Plan activities on gender equality and inclusion?

Participant: Gender equality?

Interviewer: Yes, gender equality and inclusion.

Participant: We attended related training at the district level.

Participant: That is the activity I remember.

Interviewer: What did you learn about gender equality or inclusion?

Participant: Gender equality means balancing roles and listening to everyone during deliberation before making decisions. In the past, parents, particularly fathers, often made decisions without allowing children or mothers to express their opinions. After learning about gender equality, residents have begun to understand the importance of shared views, including the views of children.

Interviewer: In your own household, who carries the greater workload, you or your wife?

Participant: In my household, there are several women and children. Work is generally shared. Tasks that I would normally do can also be handled by other family members, and responsibilities are arranged around the children's school schedules.

Interviewer: During flooding or water shortages, do men or women have more work?

Participant: I do.

Interviewer: Why?

Participant: I take the initiative.

Interviewer: What specific challenges do persons with disabilities, pregnant or postpartum women, poor residents, and children face during floods or water shortages?

Participant: In our immediate area, pregnant women have not reported serious difficulty because families help one another. [Some community members with disabilities received or required household support, including an accessibility improvement to a bathroom.]

Interviewer: Is there local wisdom from earlier generations for reducing disaster risk?

Participant: In the past, households prepared simple fire-prevention equipment, although modern conditions have changed those practices. For flooding, there was little household preparation because village spatial planning and flood prevention were generally managed by government.

Interviewer: Do you remember any traditional method for addressing floods or water shortages?

Participant: During water shortages, residents commonly ask neighbors with taps to share water for one or several days.

Interviewer: Are floods or water shortages in this village severely harmful?

Participant: Not especially.

Interviewer: On a scale from one to five, where five means very severe and one means little or no impact, what score would you give?

Participant: Three. The impact is moderate.

Interviewer: Please describe a personal experience of a disaster and how you maintained clean water and sanitation.

Interviewer: Is there an experience you can share?

Participant: During flooding, water can sometimes enter dug wells, depending on conditions. At my home, I understand how rainwater flows and prepare in advance. People throughout the village make similar preparations.

Participant: The main concern is maintaining clean water.

Interviewer: So, the score is three because neighbors or relatives can still help. Do you have concerns that affect your ability to prevent or anticipate floods and water shortages?

Participant: Water shortages have not become an overwhelming burden because residents have not lacked water completely. Flooding creates somewhat greater concern. We have proposed to the village and district governments that runoff from the mountain be slowed, perhaps through a small retention structure or stepped channel, so that it does not flow directly into the settlement. That remains unfinished work for us.

Interviewer: Thank you. Is there anything else you would like to share?

Participant: We are very grateful for STBM(Community Based Total Sanitation) . It has provided knowledge and motivation that we can apply personally, within our households, and in the wider community. I am concerned that the STBM(Community Based Total Sanitation) program may end this year. Even when local groups have been established, they may become inactive without follow-up, visits, or renewed encouragement. I hope the program continues through regular visits, further training, and stronger local management, including collaboration with the subdistrict team.

Interviewer: Thank you for your time. Plan will provide 100,000 rupiah in phone-credit reimbursement to the number I contacted through WhatsApp. I will report that the interview has been completed. Thank you for the information and your time. Assalamualaikum warahmatullahi wabarakatuh.
