# Supplementary material for: Implementing a climate-resilient and inclusive WASH guidance in Indonesia: A mixed-methods case study
Source: PLoS One. 2026 Sep 28;21(9):e0359266. doi: 10.1371/journal.pone.0359266 (PMC13618932; doi:10.1371/journal.pone.0359266)
Supplement: Supplementary file 6 [file pone.0359266.s006.zip › S6 Appendix Transcripts/KII-30_Deidentified_English.docx]

Interviewer, Participant

Interviewer: Could you provide your full name?

Participant: [participant].

Interviewer: Could you provide your current age?

Participant: I am [a middle-aged participant].

Interviewer: So, you are in your forties. What are your daily activities?

Participant: I undertake [several local livelihoods].

Interviewer: So, you are a carpenter. Do you also farm?

Participant: Yes.

Interviewer: Which do you do more often, farming or carpentry?

Participant: It depends on the season.

Interviewer: So, you work as both a carpenter and farmer?

Participant: During the rainy season, I farm more, but I also sometimes provide massage therapy.

Interviewer: Which did you begin first: farming, massage therapy, or carpentry?

Participant: I began them around the same time, although therapy came first.

Interviewer: Before we continue, I will read the consent information. My name is [interviewer], and I am a researcher for Plan's evaluation study. This study aims to identify factors that influence climate-change responses in the implementation of inclusive water, sanitation, and hygiene programs in Indonesia. It will improve our understanding of factors that enable or hinder climate-resilient and inclusive STBM(Community Based Total Sanitation) services and decision-making in village program planning. Today, we will discuss community experiences with inclusive and climate-resilient water, sanitation, and hygiene, including waste management, food hygiene, and wastewater management. Please keep these components in mind. We will use the terms extreme weather events and climate hazards interchangeably. Examples include floods, landslides, drought, and sea-level rise. Inclusive facilities are accessible to everyone. The interview will last approximately one hour and will be recorded. The recording will be stored securely, transcribed, retained for one year, and then destroyed. Participation is voluntary. You may decline, skip questions, or stop at any time without consequence. Your information will remain confidential. Please tell us if you need any accommodation. Honest responses will help us improve program recommendations, and your answers will not affect whether the village receives a grant or another program. May we begin? How long have you lived in this village, and what is your daily role?

Participant: I am [a long-term resident of the study locality].

Interviewer: Where are you originally from?

Participant: I am originally from [place of origin].

Interviewer: Please describe your daily role in this village.

Participant: My daily activities vary. I undertake [several local livelihoods].

Interviewer: Have you participated in climate-resilient STBM(Community Based Total Sanitation) activities?

Participant: Yes, in the past.

Interviewer: What activities did you attend?

Participant: I attended frequently. I once served in a leadership role during an activity at [training location], together with a colleague. We discussed waste and inclusive toilets. Many women asked what an inclusive toilet should be like. We explained that it should be environmentally appropriate and usable by everyone, including persons with disabilities and older people. For example, the access path should be suitable, handrails should be provided, and tactile guiding blocks should assist people with visual disabilities without creating steep access.

Interviewer: Have you participated in village mapping, such as identifying alleys and other locations?

Participant: Yes, I did, in [comparison village], if I remember correctly.

Interviewer: What was your role in that Plan activity?

Participant: Roles rotated. During one period, I served in the leadership group.

Interviewer: What was your impression of Plan's climate-resilient STBM(Community Based Total Sanitation) program?

Participant: For persons with disabilities, it was very good. Without such activities, the community would not know what an accessible toilet should look like. We benefited greatly from that information.

Interviewer: Does flooding occur here?

Participant: Flooding occurs in [study locality], but it is not as severe as in some other places.

Interviewer: Does floodwater reach this area?

Participant: No, Alhamdulillah.

Interviewer: What about drought?

Participant: During the current season, we have begun deepening wells.

Interviewer: Where is the well?

Participant: It is in front of the house. We covered it, but InshaAllah we will reopen it on Saturday.

Interviewer: Why?

Participant: Because of drought.

Interviewer: So, the main problem is drought rather than flooding. Does this occur every year?

Participant: Drought occurs every year in this area, but this is the first year my own household well has dried up.

Interviewer: Is the well used only by your household or shared with neighbors?

Participant: It is used by my household, but this year's drought is the most severe.

Interviewer: Your toilet has already been modified?

Participant: Yes. I modified the bathroom myself several months ago.

Interviewer: What modifications did you make?

Participant: In many places, there is only one tap. I installed three taps and a shower.

Interviewer: Did Plan activities inspire you to make those modifications?

Participant: Yes, I was inspired by Plan.

Interviewer: Besides the shower, what else did you install?

Participant: I adapted and installed the shower myself rather than purchasing a complete system.

Interviewer: May we photograph it later?

Participant: Certainly.

Interviewer: Did you add other features to make the bathroom easier to use, such as handrails?

Participant: A handrail was installed during an earlier documentation activity, and part of it remains. A bathroom should have a handrail.

Interviewer: It did not have one before?

Participant: No.

Interviewer: So, you added it after participating in the activity. Aside from the bathroom modifications, you are now deepening the well. Is the bathroom otherwise easier to use?

Participant: Alhamdulillah.

Interviewer: The water shortage began only this year, while the bathroom itself has no other problem?

Participant: Correct.

Interviewer: When water is limited, do you continue washing hands as usual or conserve water?

Participant: We conserve water, including for bathing, although hygiene remains important.

Interviewer: Besides your well, have other wells dried up?

Participant: Many neighboring wells have dried up, almost all of them.

Interviewer: What are your neighbors doing to address the drought?

Participant: They are deepening their wells.

Interviewer: Is the work conducted through mutual cooperation? This is an ordinary dug well, not a drilled well. How much deeper will it be?

Participant: Approximately one meter. It is an ordinary dug well. We reopen and deepen it, then cover it again.

Interviewer: Is there another water source besides the well?

Participant: We use well water for household needs and buy bottled water for drinking.

Interviewer: So, there is no piped supply from PAM(Regional Water Utility) . Does the village have an STBM(Community Based Total Sanitation) team?

Participant: Yes. Teams from the village and subdistrict have visited.

Interviewer: Do you participate in STBM(Community Based Total Sanitation) activities?

Participant: InshaAllah, yes.

Interviewer: Are people like you, women, and older residents involved in village and subdistrict STBM(Community Based Total Sanitation) activities?

Participant: Village understanding is still limited, although some persons with disabilities participate.

Interviewer: How have you personally participated in the STBM(Community Based Total Sanitation) team?

Participant: I was most active when I was still working in the district capital.

Interviewer: Where did you work?

Participant: At [a local workplace].

Interviewer: Since moving to the village, you have been less involved. What activities did you attend at [local hotel]?

Participant: Plan activities, mainly discussions.

Interviewer: So, you participate less frequently since moving here?

Participant: I attend when I am invited.

Interviewer: Are you able to express your views during STBM(Community Based Total Sanitation) activities?

Participant: Yes, InshaAllah.

Interviewer: Have you discussed the present water shortage only within your household, or have you informed the STBM(Community Based Total Sanitation) team or village government?

Participant: Even when we report problems to the village, the response is sometimes limited, although occasionally the issue receives attention.

Interviewer: So, the repairs are funded privately, without a village-government response to the water crisis?

Participant: We requested assistance in the past but did not receive it, although some other people did receive assistance.

Interviewer: Has tanker water been delivered here?

Participant: No.

Interviewer: During this water shortage, do you or your wife bear the greater workload?

Participant: At present, I am more involved. The water must be rationed, the pump can no longer draw water, I repair the equipment, and I will enter the well tomorrow.

Interviewer: Who decides whether to repair or deepen the well, you, your wife, or both of you?

Participant: I usually decide, although we discuss it together.

Interviewer: You and your wife discuss whether to deepen the well. Can persons with disabilities living with their families make similar decisions about improving their water supply?

Participant: In my experience, they can express a decision, but they rarely perform the work themselves.

Interviewer: So, they may be unable to implement the decision independently.

Interviewer: Because they cannot perform the physical work, another family member must act. Have you attended Plan activities concerning gender equality, disability, and social inclusion?

Participant: I was away in [another locality] at that time, so I did not attend.

Interviewer: What is the greatest challenge you personally face during this drought?

Participant: When there is no water, we must search elsewhere. Sometimes we bathe at [relatives' homes].

Interviewer: So, you can find alternative solutions. Do neighbors support you when you repair the well?

Participant: Yes.

Interviewer: So, the community helps through mutual cooperation. Have you heard of local knowledge from earlier generations about addressing drought?

Participant: In the past, when rain did not fall for a long time, people sometimes held a communal prayer for rain after Friday prayers.

Interviewer: Is that still practiced?

Participant: Not now.

Interviewer: So, such a practice existed when rain failed. Where did you learn to deepen wells and modify bathrooms?

Participant: I learned partly from attending meetings. [A relative also dug wells.]

Interviewer: On a scale from one to five, where one means no impact and five means a severe impact, how much does drought affect your ability to use water and sanitation?

Participant: It has a very serious impact.

Interviewer: What impact are you experiencing now?

Participant: Water is difficult to obtain.

Interviewer: So, many daily activities become difficult?

Participant: Even when I return home, I keep thinking about how to make the pump work.

Interviewer: Are you worried that the water problem cannot be repaired?

Participant: Yes. My main concern is obtaining enough money for the repairs.

Interviewer: Do you have any other concerns?

Participant: No, only the funding.

Interviewer: Is there any other experience related to the dry well that you would like to describe, including how difficult or frustrating it is?

Participant: It is extremely difficult. Washing dishes is hard, and washing clothes requires much more water. We must wait until a small amount of water accumulates before using it for laundry.

Interviewer: Is there anything else you would like to say about your STBM(Community Based Total Sanitation) involvement? You attend village STBM(Community Based Total Sanitation) activities when invited. Do you also participate in village planning?

Participant: I do not participate in village planning.

Interviewer: So, you have participated only in STBM(Community Based Total Sanitation) activities at the village level.
